# 1,2‐ or 1,3‐Hydride Shifts: What Controls Guaiane Biosynthesis?

**DOI:** 10.1002/chem.202101371

**Published:** 2021-05-26

**Authors:** Houchao Xu, Bernd Goldfuss, Jeroen S. Dickschat

**Affiliations:** ^1^ Kekulé-Institute for Organic Chemistry and Biochemistry University of Bonn Gerhard-Domagk-Straße 1 53121 Bonn Germany; ^2^ Institute for Organic Chemistry University of Cologne Greinstraße 4 50939 Cologne Germany

**Keywords:** carbocations, DFT calculations, hydride shifts, isotopes, terpenes

## Abstract

A systematic computational study addressing the entire chemical space of guaianes in conjunction with an analysis of all known compounds shows that 1,3‐hydride shifts are rare events in guaiane biosynthesis. As demonstrated here, 1,3‐hydride shifts towards guaianes can only be realized for two stereochemically well defined out of numerous possible stereoisomeric skeletons. One example is given by the mechanism of guaia‐4(15)‐en‐11‐ol synthase from California poplar, an enzyme that yields guaianes with unusual stereochemical properties. The general results from DFT calculations were experimentally verified through isotopic‐labeling experiments with guaia‐4(15)‐en‐11‐ol synthase.

## Graphical abstract

**A systematic exploration** of the reachable chemical space for guaianes is provided by DFT calculations that show that 1,3‐hydride migrations are exceptional events in guaiane biosynthesis. They are only possible for stereochemically distinct skeletons and open the way to unusual guaianes. The relevance of a 1,3‐hydride shift for guai‐4(15)‐en‐11‐ol synthase from poplar was shown experimentally by isotopic labeling.

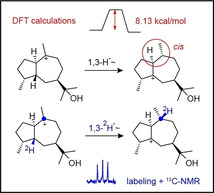

During the past two decades many terpene synthases (TPSs) have been characterized, mainly from plants,[^1^, ^2^] bacteria^[3]^ and fungi.[^4^, ^5^] These remarkable enzymes convert acyclic, achiral polyisoprenoid diphosphates into structurally complex, often polycyclic, chiral and enantiomerically enriched terpenes. These transformations involve just a single enzyme catalyzed reaction and proceed through cationic cascade reactions inside a hydrophobic cavity of the TPS. Because of their transient nature the cationic intermediates along the cascade cannot be observed spectroscopically, but especially isotopic labeling experiments[^6^, ^7^] and DFT or QM/MM calculations[^8^, ^9^, ^10^, ^11^, ^12^, ^13^] have helped to develop a deep mechanistic understanding of TPS catalysis. Also structure based site‐directed mutagenesis can give valuable insights,[^14^, ^15^, ^16^] especially if an enzyme variant leads to an aberrant product formed by deprotonation of a cationic intermediate, giving indirect evidence for its existence. In some cases terpene cyclizations proceed through a neutral (deprotonated) intermediate that can be reactivated by reprotonation for further downstream cyclization steps; these neutral intermediates can often be observed as minor products, as they can leak from the enzyme's active site. It is, however, difficult to distinguish in these cases between true intermediates and shunt products, because instead of a deprotonation‐reprotonation sequence a direct intramolecular or water/enzyme mediated proton transfer could bypass such a hypothetical neutral “intermediate”. While keeping this in mind, for simplification we will no longer differentiate here between neutral “intermediate” and “shunt product”, or only where it is relevant.

Germacrene A (**1**) and hedycaryol (**2**) belong to the most important intermediates of sesquiterpene biosynthesis and numerous compounds derive from them,^[17]^ likely because their fairly strained ten‐membered ring is sufficiently reactive for further protonation induced cyclizations (Scheme 1).

**Scheme 1:**
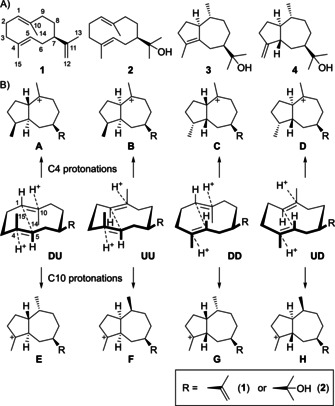
Biosynthesis of guaiane sesquiterpenes. A) Structures of germacrene A (**1**) and of the PtTPS5 products **2**–**4**. B) Possible cyclization reactions from **1** and **2** to different stereoisomers of the guaiane skeleton.

In almost all cases the resulting structures can be explained by reprotonation of a double bond with a well explainable face selectivity, that is, attack of the proton from the enzyme exposed face and not at the inner face of the macrocycle. Starting from different ring conformations (**DU**, **UU**, **DD** and **UD**, referring to Me14 and Me15 down=D or up=U) the reprotonation at C4 leads to the four stereoisomeric intermediates **A**–**D**, while the four intermediates **E**–**H** can be reached through reprotonations at C10 (plus their enantiomers from the antipodes of **1** and **2**). As a result of the *E*‐configured double bonds in **1** and **2** the C4 reprotonations always lead to a *trans* orientation of Me15 and H5, while for C10 reprotonations Me14 and H1 are always *trans*. We have recently reported about the oomycete infection induced PtTPS5 from *Populus trichocarpa* (California poplar) that converts farnesyl diphosphate (FPP) into (1*S*,7*R*,10*R*)‐guaia‐4‐en‐11‐ol (**3**) and (1*S*,5*S*,7*R*,10*R*)‐guaia‐4(15)‐en‐11‐ol (**4**), besides minor amounts of **2**.^[18]^ The double bond positioning in **3** and **4** indicates a cationic precursor with the charge residing at C4, which could be reached directly through cyclization of **2** upon C10 protonation, but none of the intermediates **E**–**H** fulfills the stereochemical requirements of the observed products.

A systematic analysis of the reachable chemical space revealed that such a situation is very rare among guaiane sesquiterpenes. For this purpose, the possible structures of guaiadienes and guaienols were identified as the three different deprotonation products of each of the intermediates **A**–**H** (Schemes S1 and S2 in the Supporting Information). Further compounds can be reached through 1,2‐ or 1,3‐hydride migration or their combinations and different deprotonation events (Schemes S3–S14). The systematics of this approach is summarized for the **D** series in Scheme 2A which includes the precursors for **3** (**D4** or **D5**) and **4** (**D5**). In some cases the same compounds can be formed through alternative pathways, such as **D3** and its deprotonation products can hypothetically arise from **D1** by two sequential 1,2‐hydride shifts or one 1,3‐hydride migration (but in other cases the order of steps is relevant, for example, starting from **D** the sequence of 1,2‐ plus 1,3‐hydride migration leads to **D3**, while the reverse order of 1,3‐ plus 1,2‐hydride transfer leads to another stereoisomer **D5**). This analysis also turned out that some compounds can be obtained from different initial cyclization products, for example, a 1,2‐hydride shift from **B** or a 1,3‐hydride shift from **F** both lead to **B1**=**F4** (Scheme 2B). All structures are summarized together with the information about their potential precursors **A**–**H** in Figures S1–S9.

**Scheme 2:**
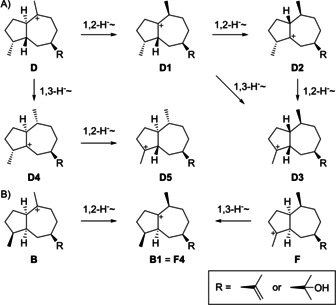
Exploring the reachable chemical space of guaiane sesquiterpenes. A) Cationic intermediates from the initially formed bicyclic intermediate, here exemplified for the **D** series. B) Some cationic intermediates can be reached from different starting points.

These considerations have so far neglected whether the proposed hydride migrations can indeed be realized or not. While it seems rationale to assume that 1,2‐hydride shifts may be possible in every case, some of the 1,3‐hydride shifts that were taken into account might be prevented by steric constraints, that is, the hydride to be shifted may point away from the empty p orbital at the cationic center, so that no significant orbital overlap can be achieved. To gain deeper insights DFT calculations for all eight series starting from **A**–**H**, with both substituents of an isopropenyl or a hydroxyisopropyl group, were carried out for all (corresponding) hydride migrations as in Scheme 2A (Figures S10–S25). As expected, low to moderate transition state (TS) barriers between 0.76 kcal/mol (**H‐TS1** in Figure S25) and 11.14 kcal/mol (**H‐TS3** in Figure S16) were obtained for all 1,2‐hydride shifts, with an average TS barrier of 5.13 kcal/mol. In contrast, several 1,3‐hydride shifts could not be realized, including those from **E1** to **E3** and the corresponding intermediates in the **F**, **G** and **H** series (Figures S18–S25), while for the **A**–**D** series high TS barriers were found for this step (Figures S10–S17). Because this step can be substituted by two sequential 1,2‐hydride shifts in all cases, 1,3‐hydride shifts are, if possible at all, likely not relevant here. Furthermore, hydride shifts could not be realized for the steps from **B** to **B4** and from **C** to **C4** (Figures S12–S15), while the corresponding steps showed high TS barriers in the **E**–**H** series (Figures S18–S25). Only for the **A** and **D** series this step with TS barriers between 7.27 and 9.72 kcal/mol is feasible (Figures S10, S11, S16 and S17). These 1,3‐hydride shifts, where possible, open the path towards guaiane stereoisomers that cannot be reached through another sequence of hydride shifts. Going back with these insights to the known natural products and their possible mechanisms of formations (cf. precursor cations and their color code in Figures S1–S9), it becomes clear that the PtTPS5 product **4** is the only known guaiane that must be generated with participation of a 1,3‐hydride shift, at least if the so far discussed simple mechanistic models apply, whereas in many other cases an optional 1,3‐hydride shift can be substituted by two energetically more feasible 1,2‐hydride transfers. The only other known compounds for which 1,3‐hydride shifts could be relevant are **3**, and it is logical to assume a common biosynthetic mechanism for **3** and **4** by PtTPS5, and (1*S*,7*R*,10*R*)‐guai‐4‐en‐11‐ol from *Bulnesia sarmientoi* (Figure S6).^[19]^ In fact, for the latter compound only future experimental work will give clarification, as it can be formed from **A** through 1,3‐hydride shift (9.72 kcal/mol, Figure S10) and deprotonation, or from **D** through two sequential 1,2‐hydride shifts (highest TS is 8.54 kcal/mol, Figure S17) and deprotonation.

Are mechanistic alternatives to the so far considered path A of Scheme 3 for the biosynthesis of **3** and **4** possible? The cyclization of FPP and capture with water could lead to protonated hedycaryol (**2**‐H^+^), with subsequent direct intramolecular proton transfer to C10 from the inner sphere, which can directly result in cyclization to **D5** (path B). In this case, the observed PtTPS5 product **2** would be identified as a shunt product rather than an intermediate towards **3** and **4**. Or if (2*E*,6*Z*)‐FPP would be the substrate of PtTPS5, this could be cyclized to **5**, a stereoisomer of **2**, that could be followed by outer sphere protonation to induce direct cyclization to **D5** (path C). This hypothesis is unlikely, because normal (2*E*,6*E*)‐FPP is efficiently converted into **3** and **4** by PtTPS5, and there is no good mechanistic explanation for a 6*E*/6*Z* double bond isomerization in FPP.

**Scheme 3:**
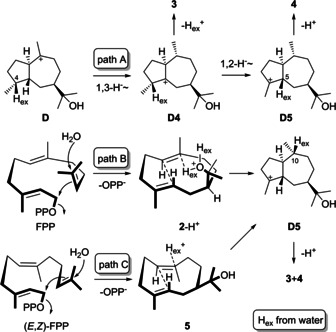
Biosynthetic hypotheses for the PtTPS5 products **3** and **4**.

To distinguish between path A and path B isotopic labeling experiments were performed (Scheme 4 and Figure 1). While path A must operate with proton incorporation from water at C4 with subsequent migration to C5 in **4** or loss by deprotonation in **3**, path B should give proton incorporation at C10 of both products. Incubation of (7‐^13^C)FPP^[20]^ in deuterium oxide gave strongly enhanced singlets for C10 of **3** and **4** in the ^13^C NMR spectrum, with a small upfield shift for **4** indicating deuterium incorporation two positions away (Figure 1A). The same experiment with (6‐^13^C)FPP^[20]^ gave singlets for C1 of **3** and **4**, again with an upfield shift for **4**, in line with deuterium incorporation at a neighboring position (Figure 1B), while (2‐^13^C)FPP^[20]^ in ^2^H_2_O gave a singlet for C4 of **3** and an upfield shifted triplet for **4**, giving direct evidence for deuterium incorporation at C5 (Figure 1C), in line with path A and conflicting path B. The 1,3‐hydride shift in the biosynthesis of **3** and **4** was demonstrated by incubation of (2‐^2^H)DMAPP^[21]^ and (3‐^13^C)IPP^[22]^ with isopentenyl diphosphate isomerase from *Escherichia coli* ^[23]^ and FPP synthase (FPPS) from *Streptomyces coelicolor*.^[24]^ This will yield a mixture of isotopomers of [3,7,11‐^13^C_3_,2,6,10‐^2^H_3_]FPP in which each terpene unit carries either a ^13^C‐ or a ^2^H‐labeling, but not both simultaneously. Further conversion with PtTPS5 gave corresponding mixtures of isotopomers of **3** and **4** (all eight FPP isotopomers in this mixture and their conversion by PtTPS5 are shown in Scheme S15). For the relevant carbon C10 that only gives a strong signal in the ^13^CNMR if it is ^13^C‐labeled itself, either upfield shifted triplet signals for a ^1^ *J*_C,D_ coupling with deuterium or doublets for a ^3^ *J*_C,C_ coupling with ^13^C (C4) were observed, depending on whether the third unit of FPP was derived from (2‐^2^H)DMAPP or (3‐^13^C)IPP. The triplet coupling with deuterium unequivocally established the 1,3‐hydride shift in the biosynthesis of **3** and **4** (the triplets are not explainable by two sequential 1,2‐hydride shifts, because then the deuterium and the ^13^C‐labelings must be incorporated into the same terpene unit, which is not possible from the precursors used).

**Scheme 4:**
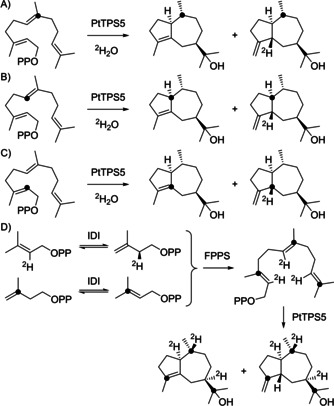
Labeling experiments on the cyclization mechanism of PtTPS5.

**Figure 1:**
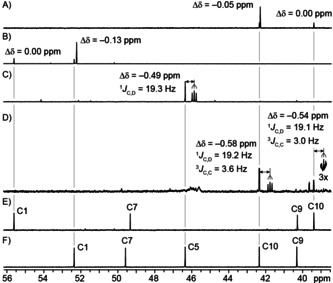
Labeling experiments on the cyclization mechanism of PtTPS5. Partial ^13^C NMR spectra for labeled **3** and **4** obtained from A) (7‐^13^C)FPP, B) (6‐^13^C)FPP or C) (2‐^13^C)FPP in D_2_O, D) obtained from [3,7,11‐^13^C_3_,2,6,10‐^2^H_3_]FPP, and for E) unlabeled **3** and F) unlabeled **4**.

After having established path A experimentally and computationally, a possible mechanism for the protonation‐induced cyclization of **2** by PtTPS5 was investigated in more detail. Here the question is what could be the source of the proton to induce the second cyclization? For a similar step by the fungal myrothec‐15(17)‐en‐7‐ol synthase from *Myrothecium gramineum* (MgMS) recently a proton transfer mediated through two water molecules to induce further cyclization events was suggested based on DFT calculations (Scheme 5A).^[25]^ Such a mechanism could also be of interest for catalysis by PtTPS5 (Scheme 5B). To investigate this hypothesis DFT calculations were started from protonated hedycaryol (Figure 2). Water‐mediated protonation at C4 required bridging by two molecules of water, leading through D‐TS0* with a TS barrier of 5.80 kcal/mol to D* (one molecule of water was not sufficient to realize this step). The 1,3‐hydride transfer can also be assisted by the water network, but the barrier for D‐TS5* (11.40 kcal/mol) is not better than without water (8.13 kcal/mol, Figure S17). However, the presence of water can explain a very smooth deprotonation of D4* through D‐TS6* to the product 3* that is with 0.25 kcal/mol nearly barrierless. Instead of water, also active site residues or diphosphate could be involved in mediating proton transfers or act as a base in the final deprotonation.

**Scheme 5:**
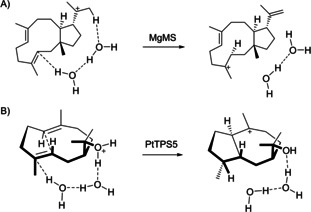
Water‐mediated proton transfer in the terpene cyclizations A) to myrothec‐15(17)‐en‐7‐ol by MgMS, and B) to **3** and **4** by PtTPS5.

**Figure 2:**
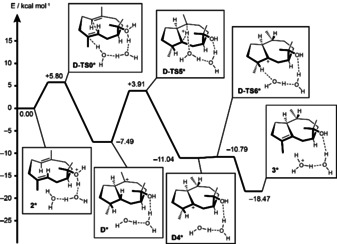
Energy profile (Gibbs energies, 298 K, mPW1PW91/6‐311+G(d,p)//B97D3/6‐31g (d,p)) for the PtTPS5 mechanism including a water‐mediated proton transfer.

In conclusion, we have shown that many guaiane skeletons can easily be reached by 1,2‐hydride migrations. It is surprising that many theoretically possible structures have not been discovered from natural sources. This could mean that some structures are privileged in nature, but there is also another possible explanation: today countless studies rely only on GC/MS‐based compound identification, even without the use of reference standards. It is well known that the stereoisomers of terpenes can have similar mass spectra and retention indices, and if one compound has been reported hundreds of times, it may be tempting to claim to have found the same “privileged structure”, when in fact it is one of the missing compounds. In contrast to 1,2‐hydride migrations, 1,3‐hydride shifts are exceptional events in guaiane biosynthesis. A deep analysis of the eight stereoisomeric series **A**–**H** demonstrated that, in many cases, 1,3‐hydride shifts are sterically impossible or they are associated with high barriers, making their participation very unlikely. However, in these cases, 1,3‐hydride transfers cannot fully be excluded because relevant barriers could be lowered by the enzyme. For a very few cases, 1,3‐hydride shifts must be considered as there is no other obvious solution to the formation of the observed skeletons; this includes the PtTPS5 products **3** and **4**. Here, the barriers for the 1,3‐hydride shifts are comparably low and seem to be realizable, as verified in this study experimentally through isotopic labeling. Furthermore, our DFT calculations show that a conceptually interesting water‐mediated proton transfer could be involved in the terpene cyclization to **3** and **4** by triggering the second cyclization event, but for deeper insights QM/MM calculations based on a crystal structure would be needed. Systematic explorations of the reachable chemical space of guaiane sesquiterpenes have demonstrated that for some known compounds such as (1*S*,4*S*,5*S*,7*S*)‐guai‐9‐en‐11‐ol from *B. sarmientoi* (Figure S1)^[19]^ and guaia‐5,11‐diene from *Cymbastela hooperi* (Figure S9)^[26]^ only one biosynthetic mechanism is plausible, while for most of the known compounds mechanistic alternatives can apply. In none of these cases has the cyclization mechanism been studied to distinguish between these alternatives; this opens up an interesting playground for future terpene biosynthesis work.

## Conflict of interest

The authors declare no conflict of interest.

## Supporting information

As a service to our authors and readers, this journal provides supporting information supplied by the authors. Such materials are peer reviewed and may be re‐organized for online delivery, but are not copy‐edited or typeset. Technical support issues arising from supporting information (other than missing files) should be addressed to the authors.

Supplementary
