# Supplementary material for: 1,2‐ or 1,3‐Hydride Shifts: What Controls Guaiane Biosynthesis?
Source: Chemistry. 2021 May 26;27(38):9758–62. doi: 10.1002/chem.202101371 (PMC8362104; doi:10.1002/chem.202101371)
Supplement: Supplementary file 1 — Supplementary [file CHEM-27-9758-s001.pdf]

Chemistry–A European Journal

Supporting Information

1,2- or 1,3-Hydride Shifts: What Controls Guaiane Biosynthesis?

Houchao Xu, Bernd Goldfuss,* and Jeroen S. Dickschat*

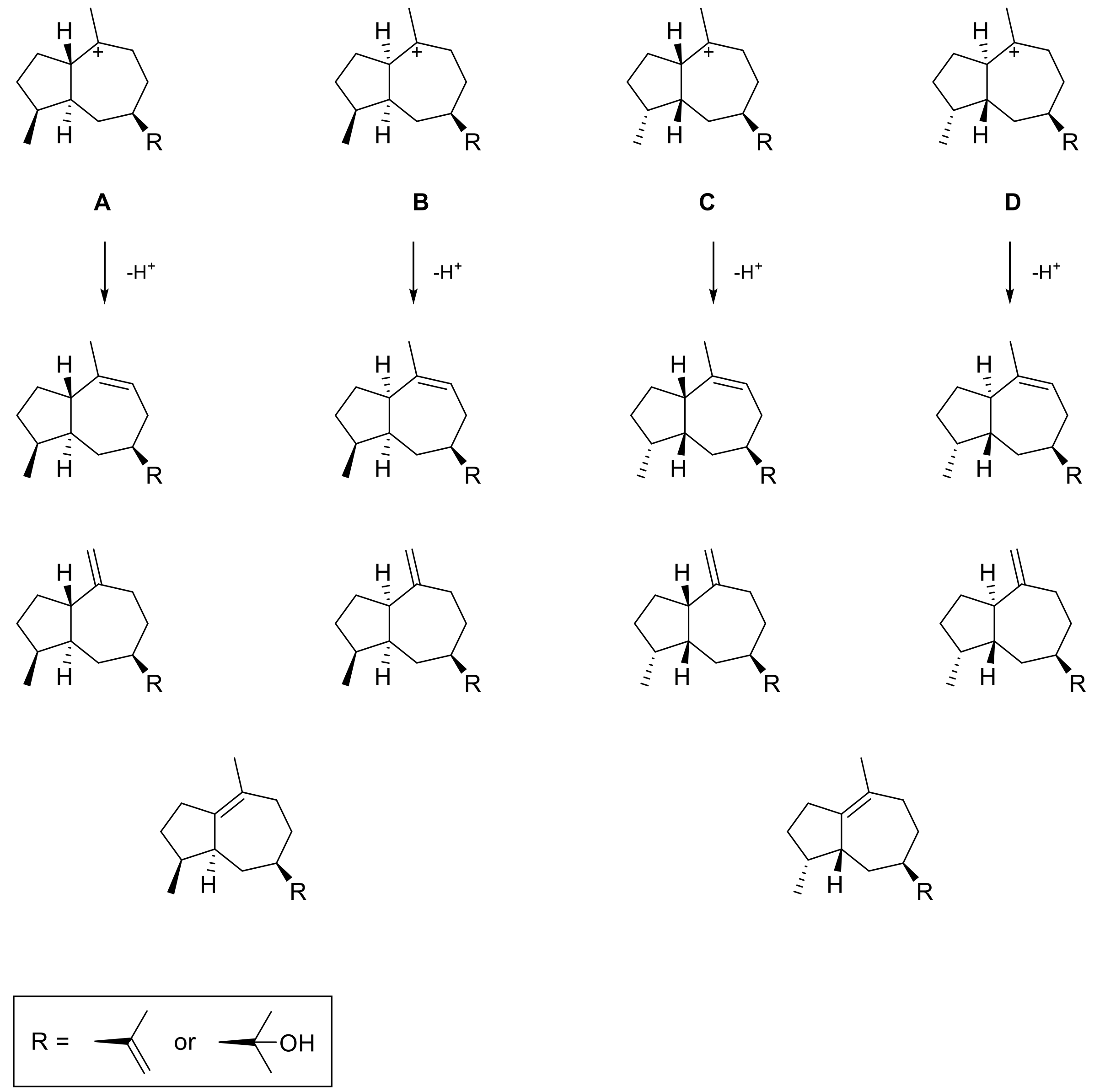

Scheme S1. Guaiane sesquiterpenes that are accessible from intermediates **A – D** through deprotonation.

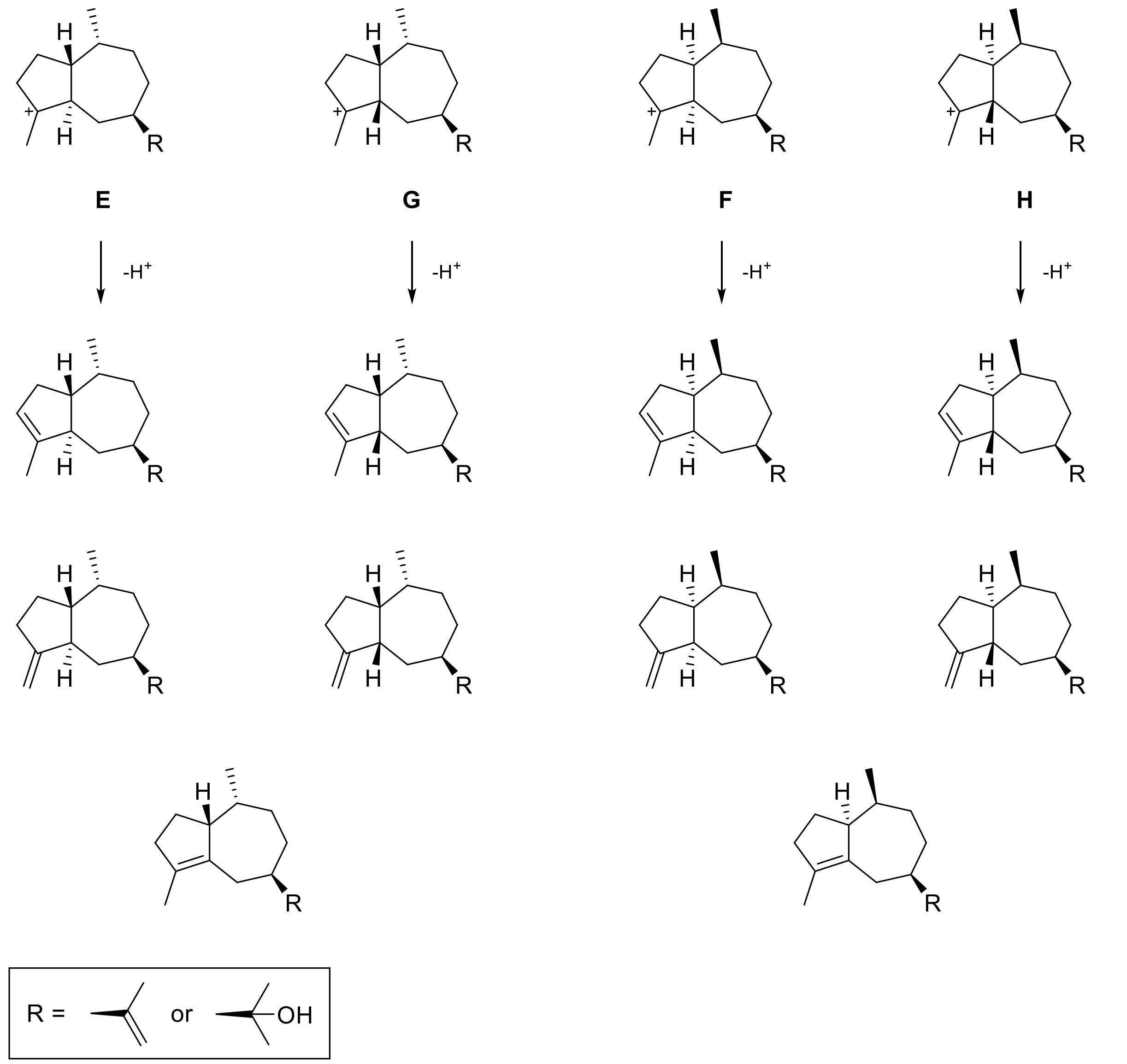

Scheme S2. Guaiane sesquiterpenes that are accessible from intermediates **E – H** through deprotonation.

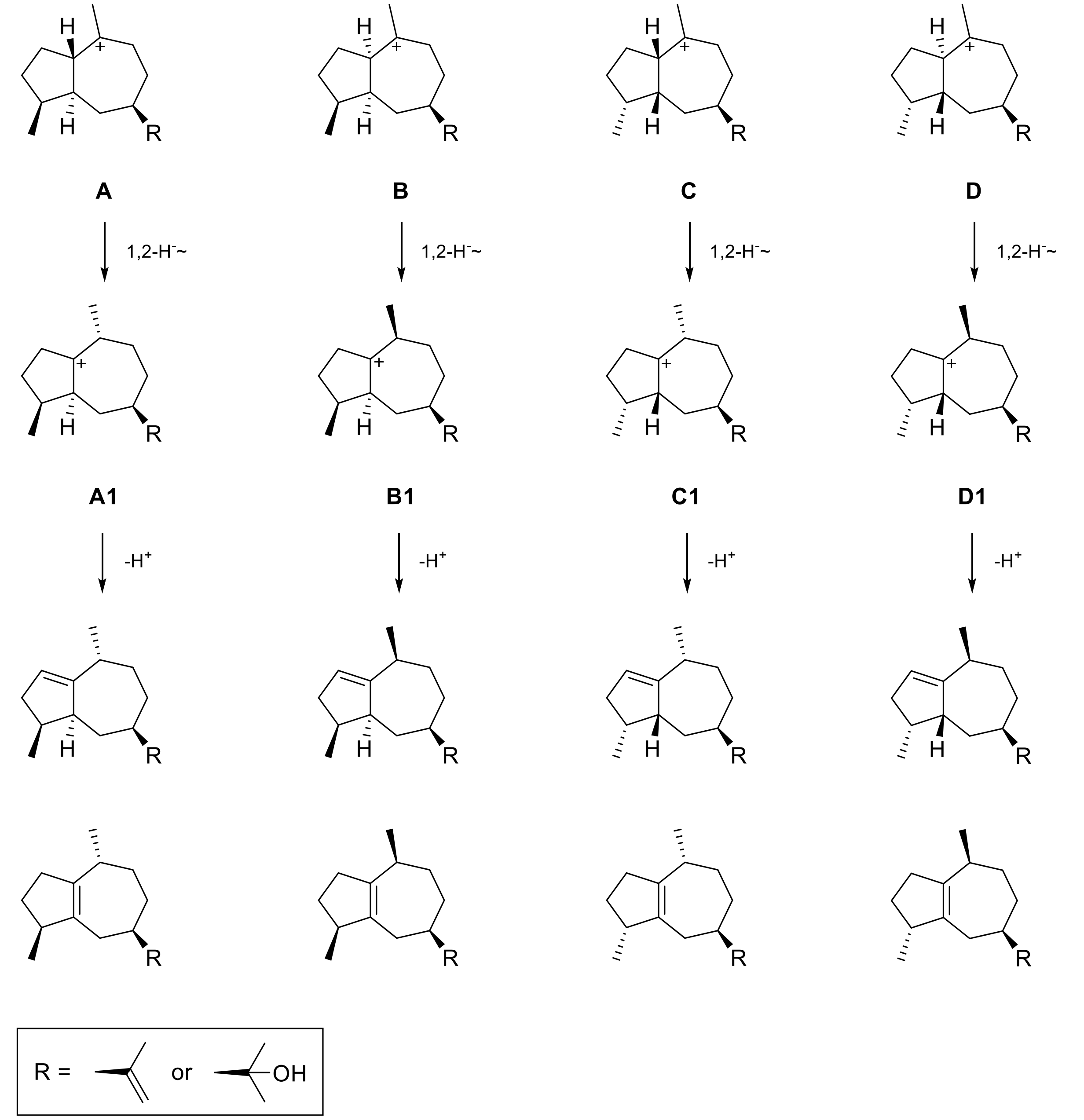

Scheme S3. Guaiane sesquiterpenes that are accessible from intermediates **A – D** through one 1,2-hydride shift and deprotonation.

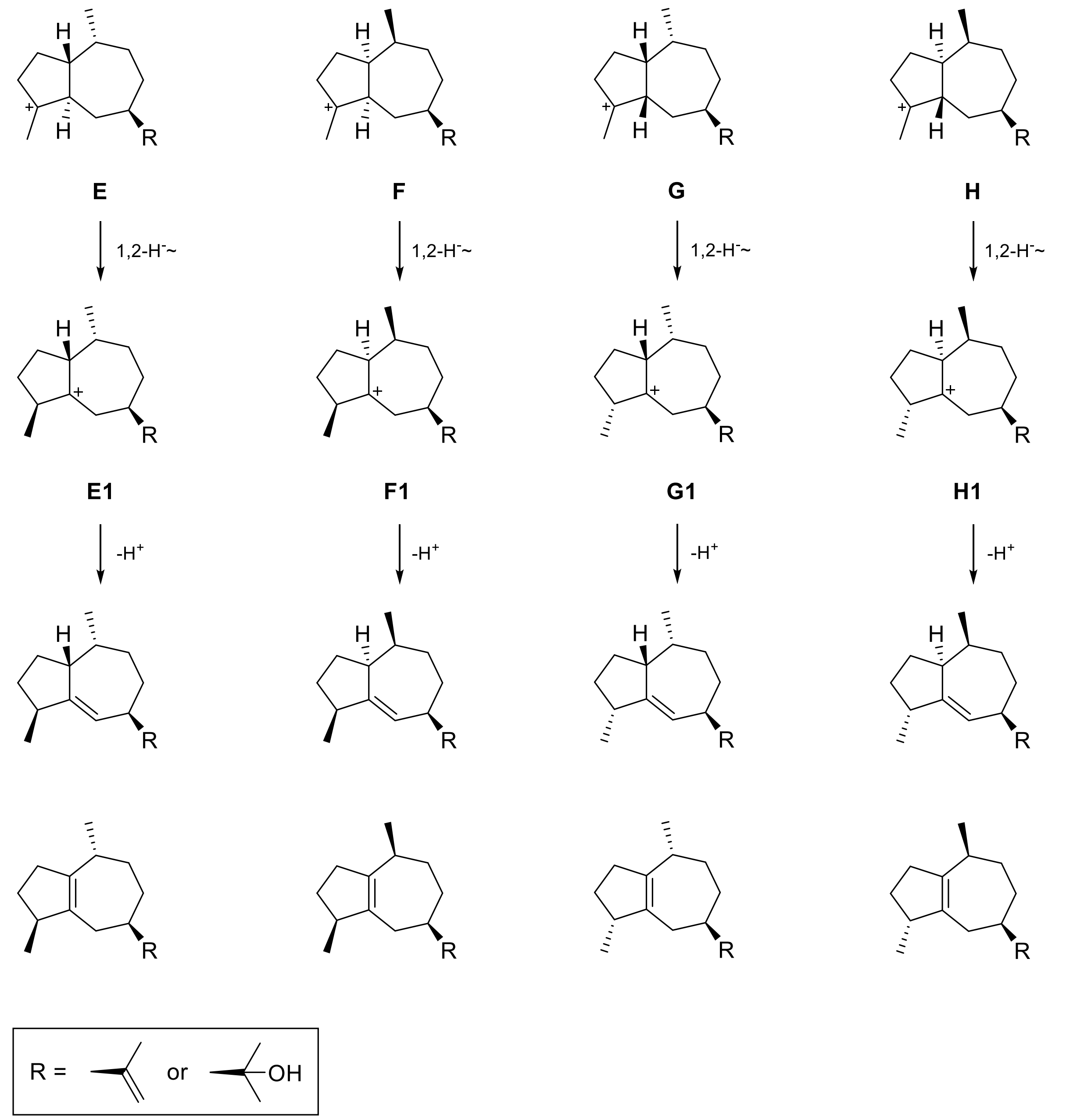

Scheme S4. Guaiane sesquiterpenes that are accessible from intermediates **E – H** through one 1,2-hydride shift and deprotonation.

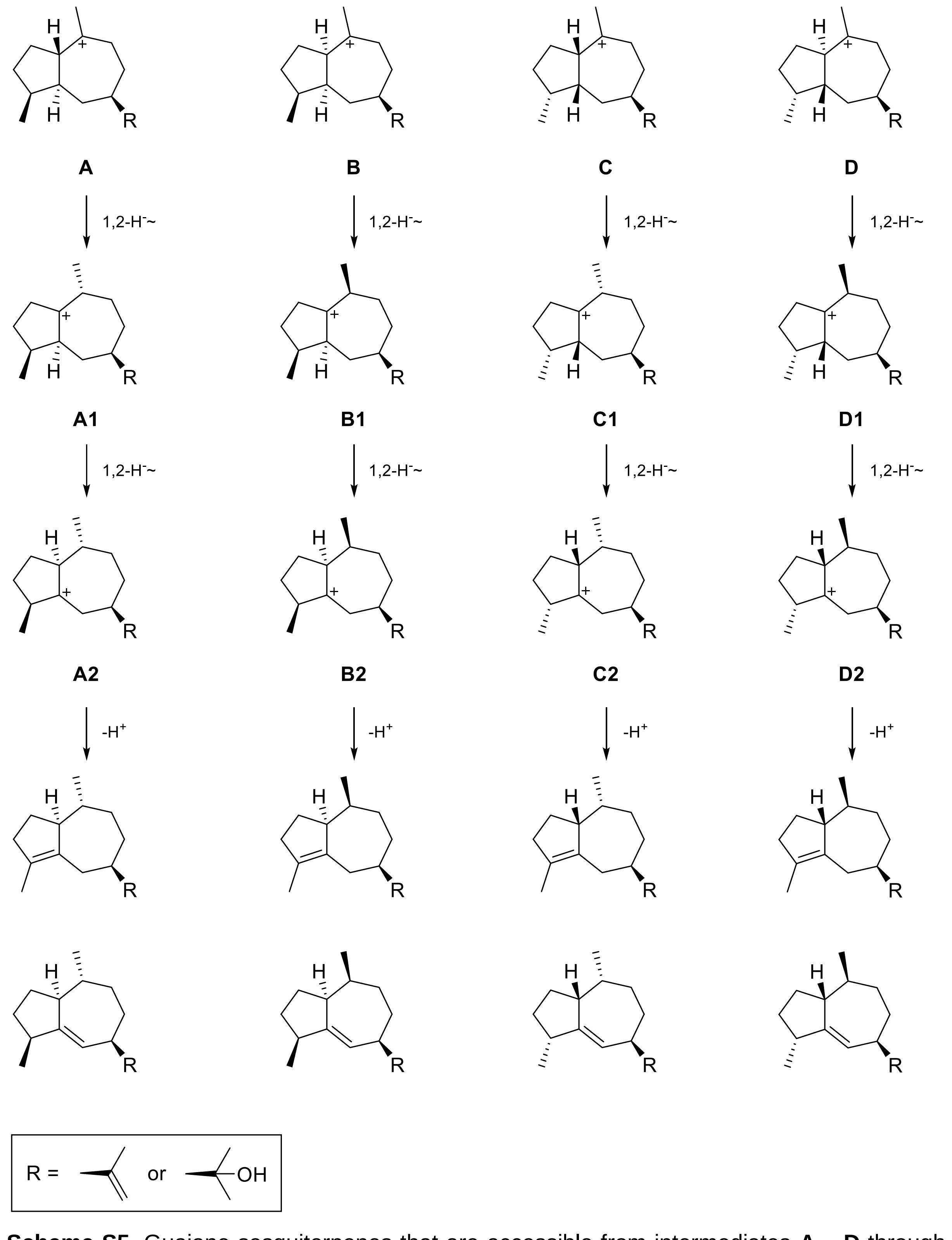

Scheme S5. Guaiane sesquiterpenes that are accessible from intermediates **A – D** through two sequential 1,2-hydride shifts and deprotonation.

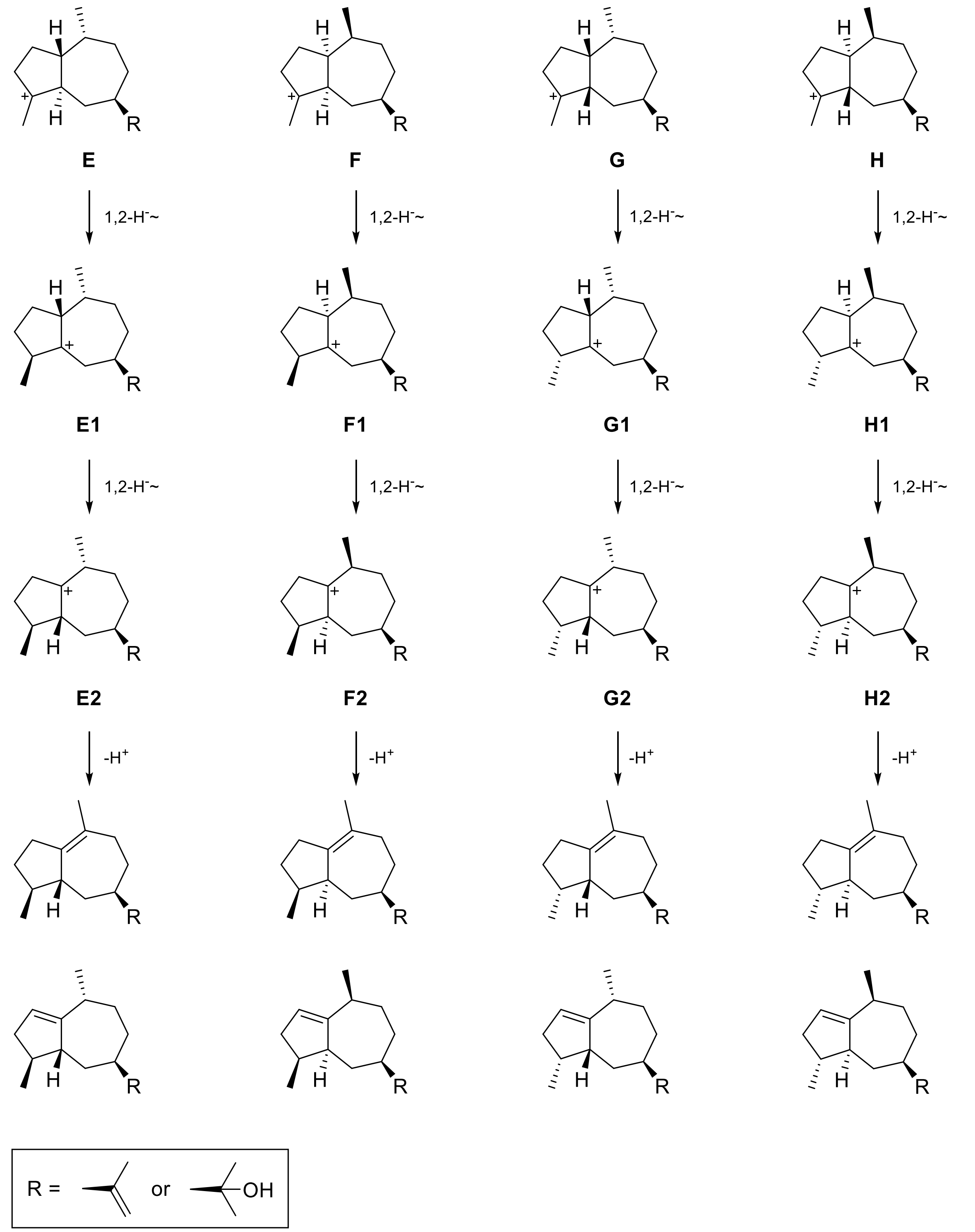

Scheme S6. Guaiane sesquiterpenes that are accessible from intermediates **E – H** through two sequential 1,2-hydride shifts and deprotonation.

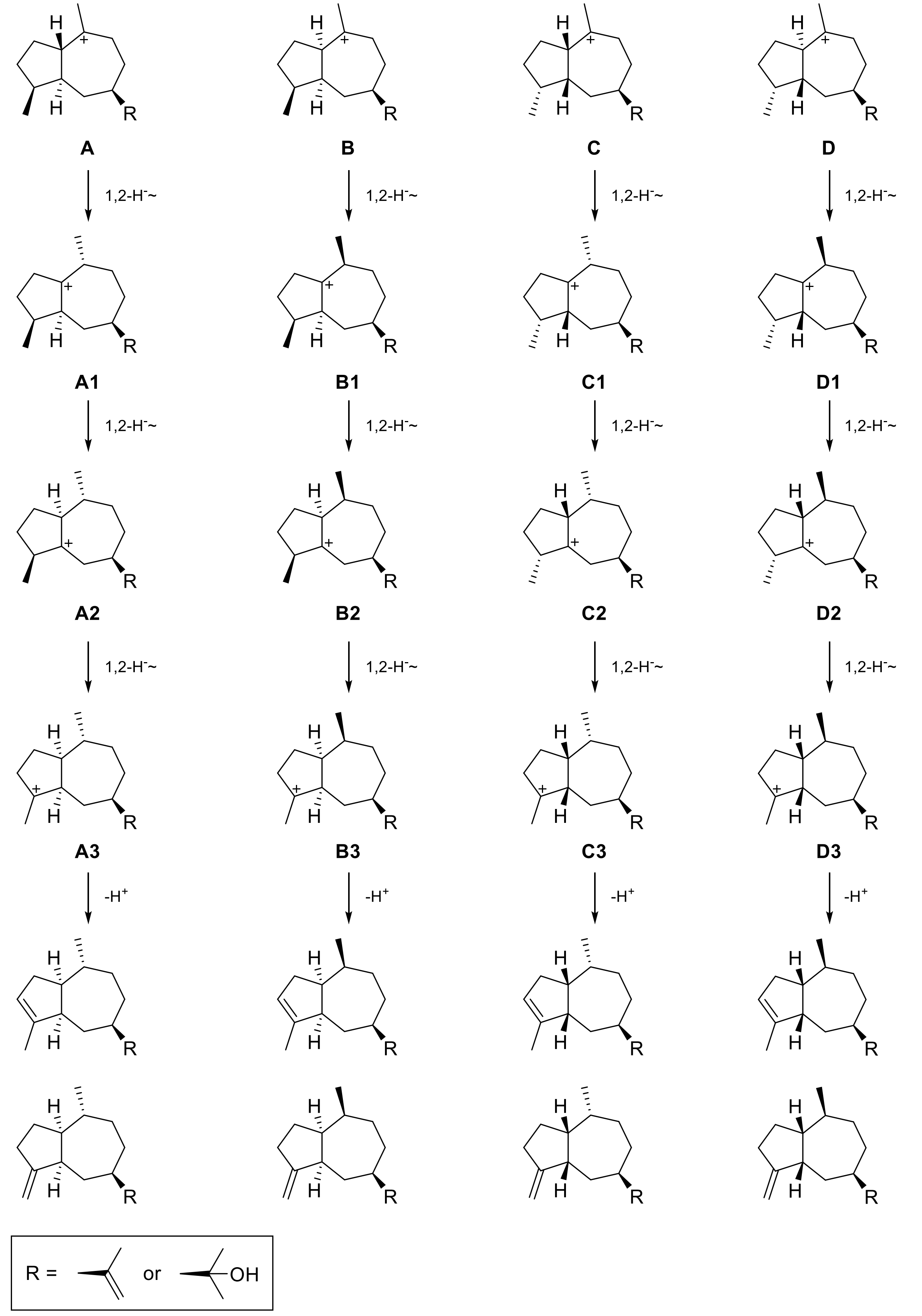

Scheme S7. Guaiane sesquiterpenes that are accessible from intermediates **A – D** through three sequential 1,2-hydride shifts and deprotonation.

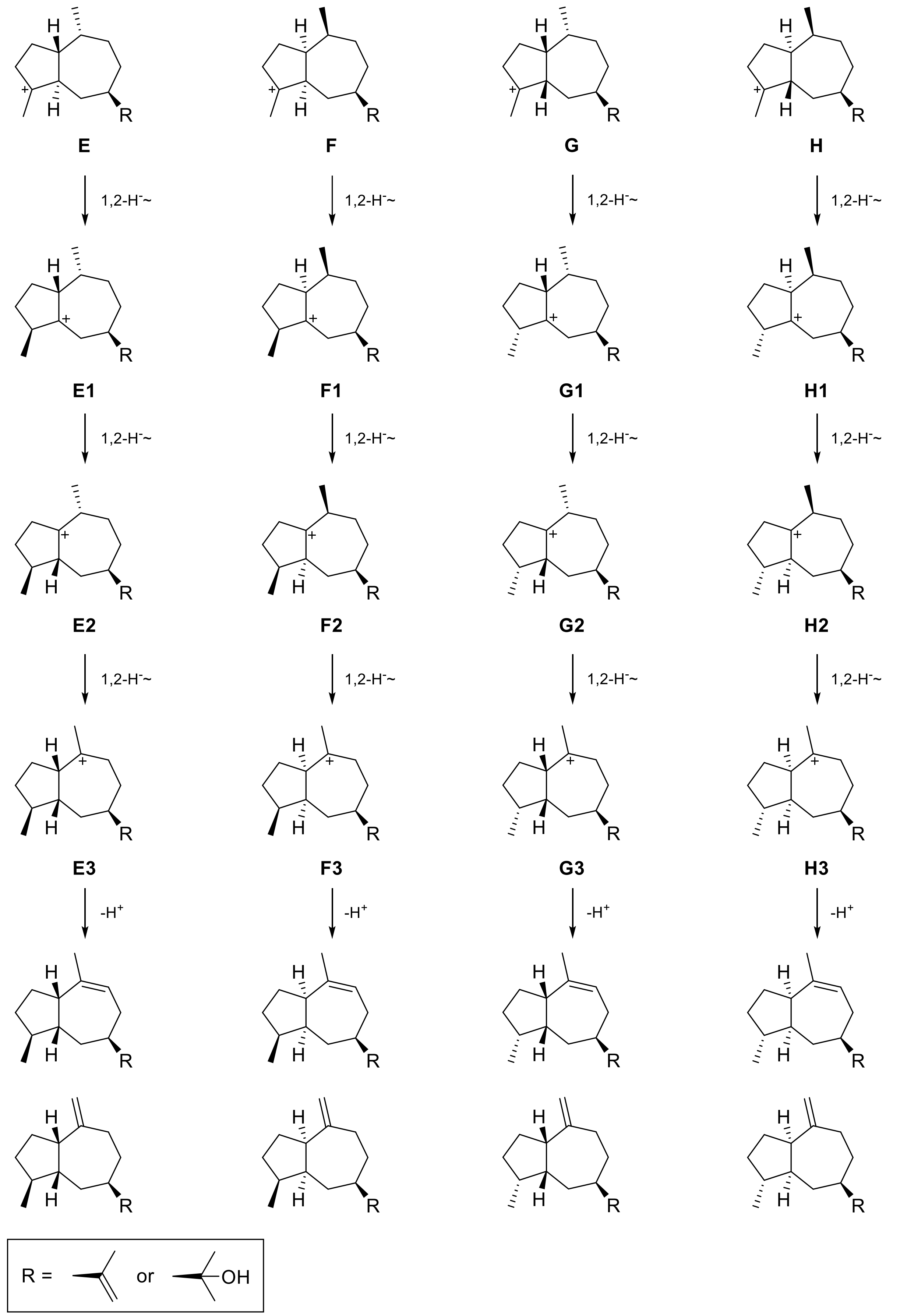

Scheme S8. Guaiane sesquiterpenes that are accessible from intermediates **E – H** through three sequential 1,2-hydride shifts and deprotonation.

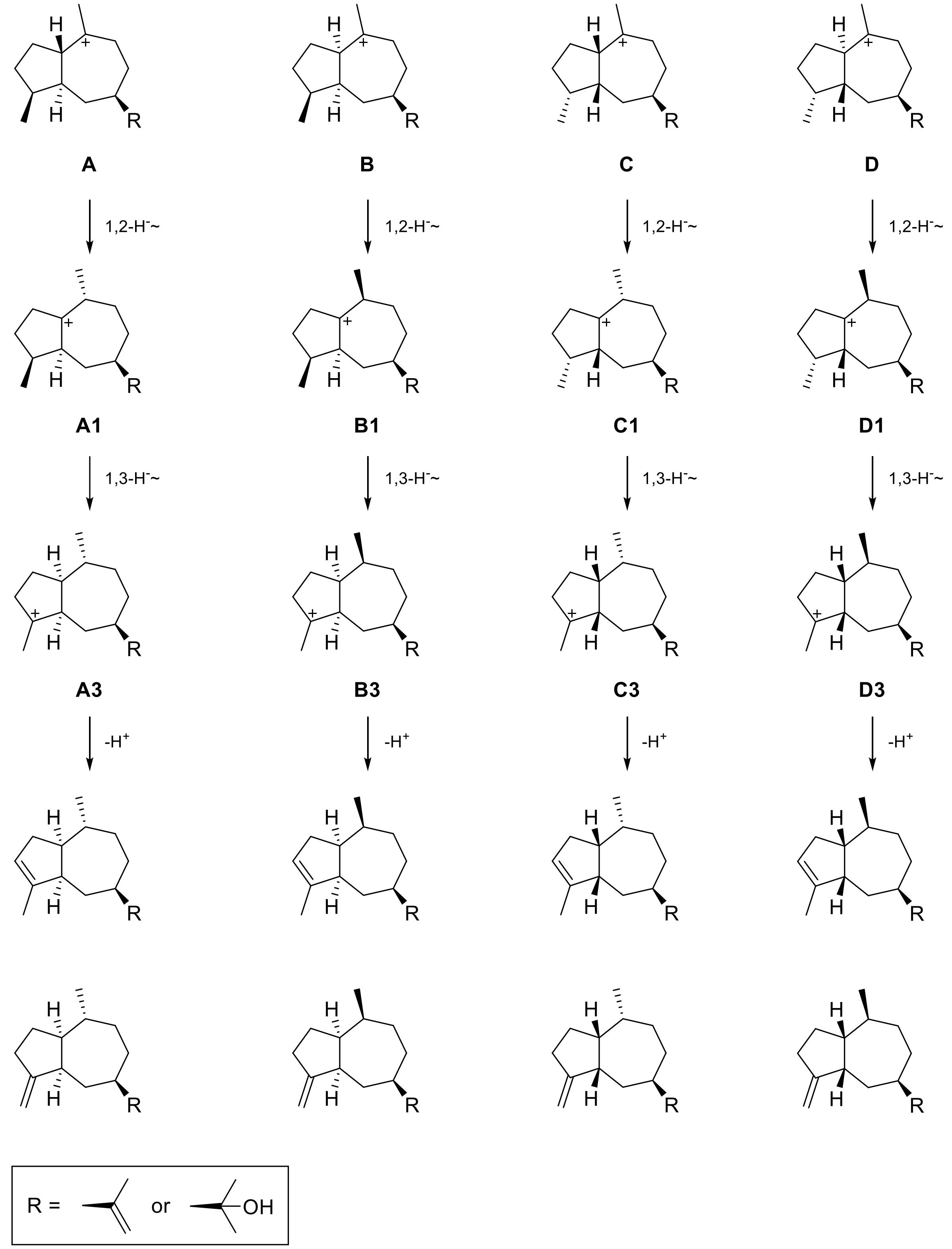

Scheme S9. Guaiane sesquiterpenes that are accessible from intermediates **A – D** through one 1,2-hydride shift, followed by one 1,3-hydride shift and deprotonation. These reactions result from all intermediates **A – D** in the same compounds as in Scheme S7.

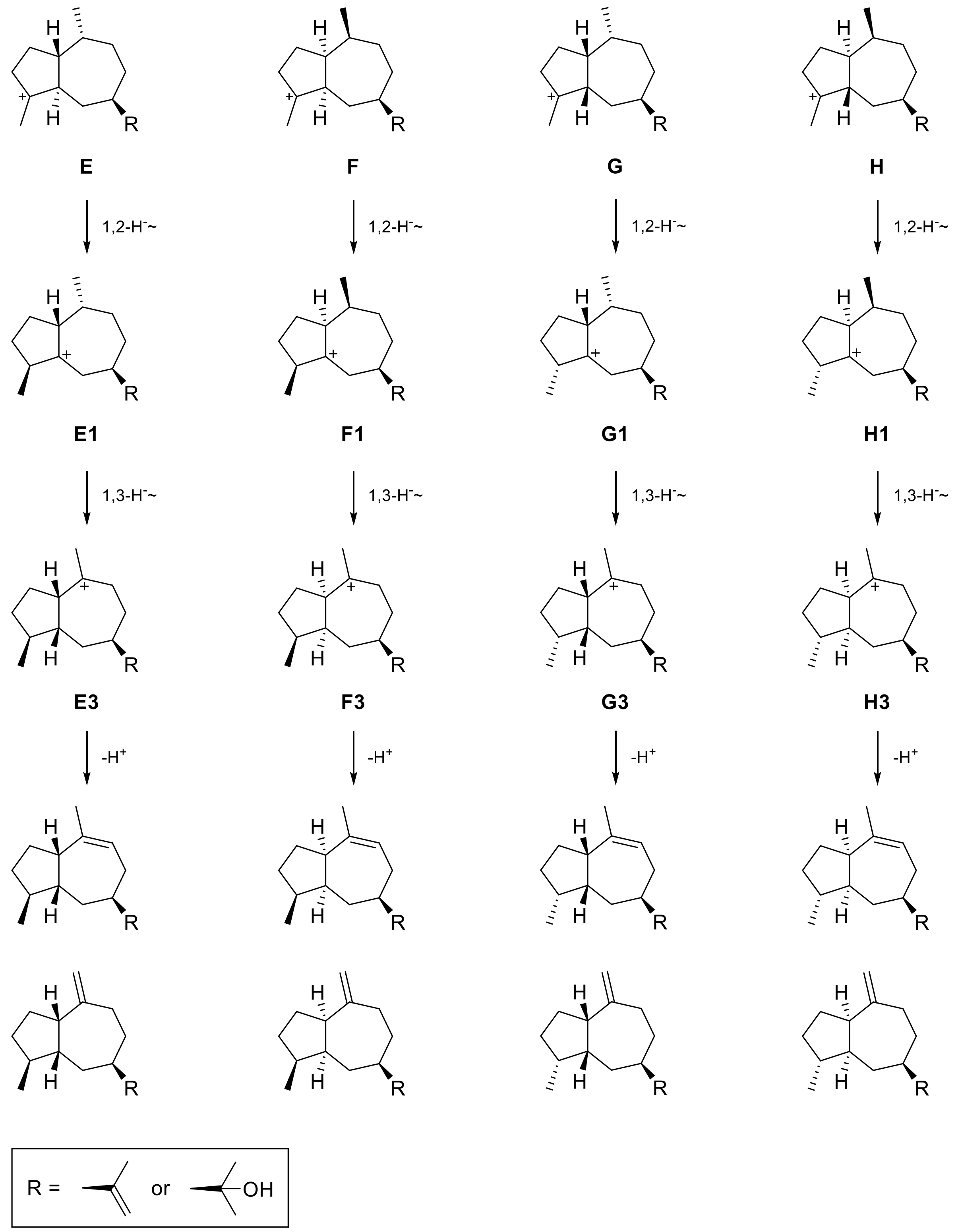

Scheme S10. Guaiane sesquiterpenes that are accessible from intermediates **E – H** through one 1,2-hydride shift, followed by one 1,3-hydride shift and deprotonation. These reactions result from all intermediates **E – H** in the same compounds as in Scheme S8.

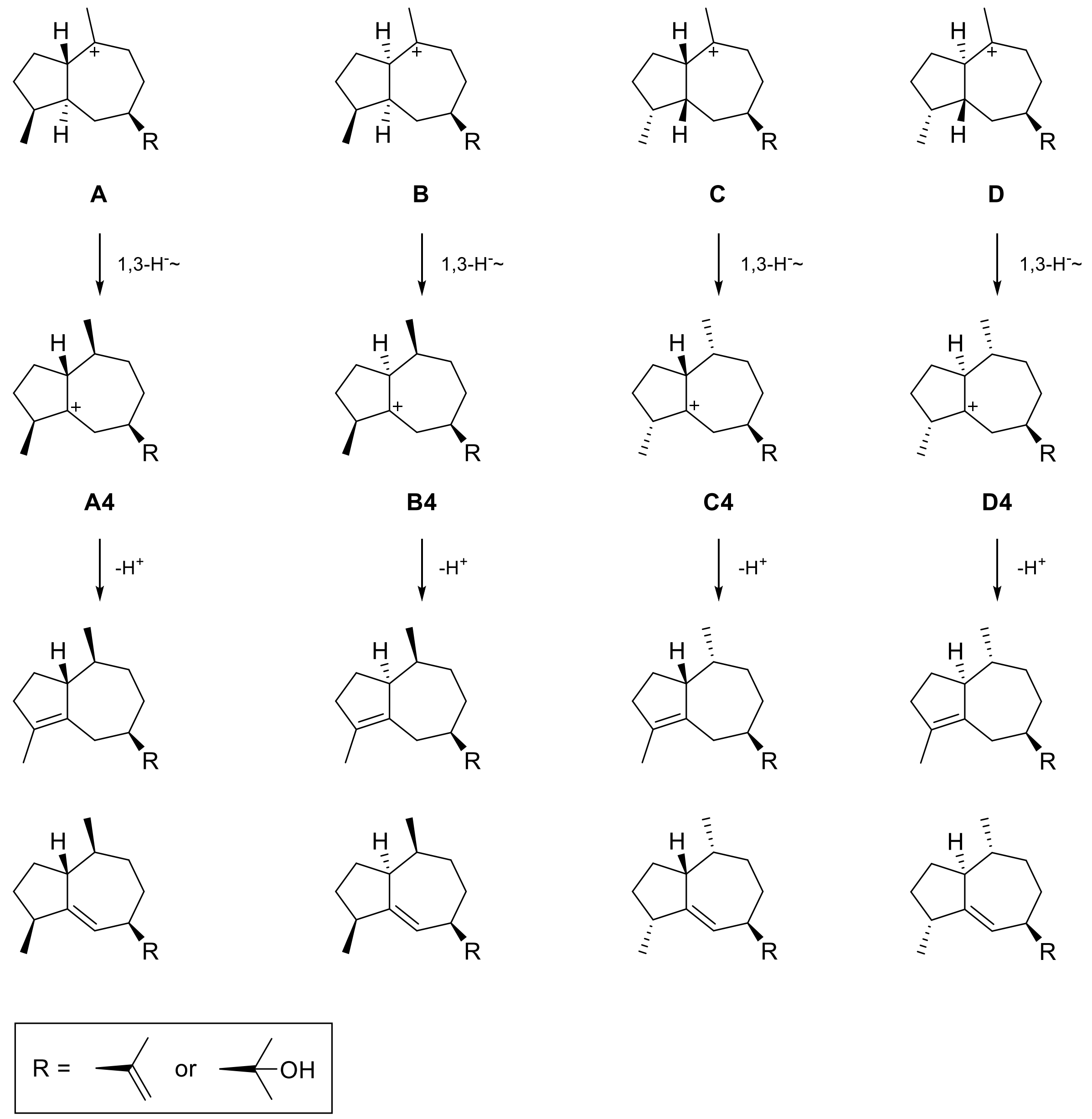

Scheme S11. Guaiane sesquiterpenes that are accessible from intermediates **A – D** through one 1,3-hydride shifts and deprotonation.

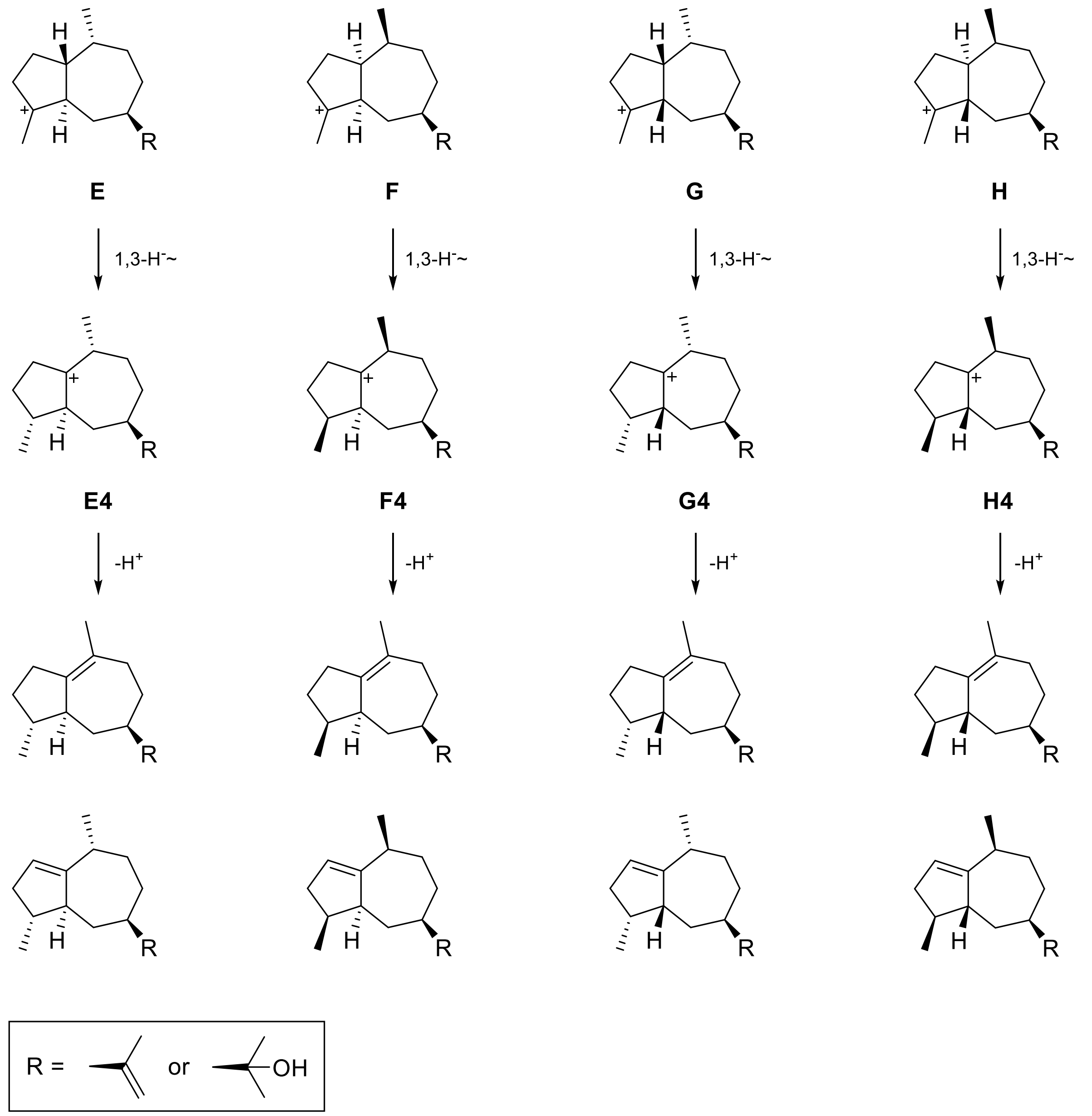

Scheme S12. Guaiane sesquiterpenes that are accessible from intermediates **E – H** through one 1,3-hydride shifts and deprotonation.

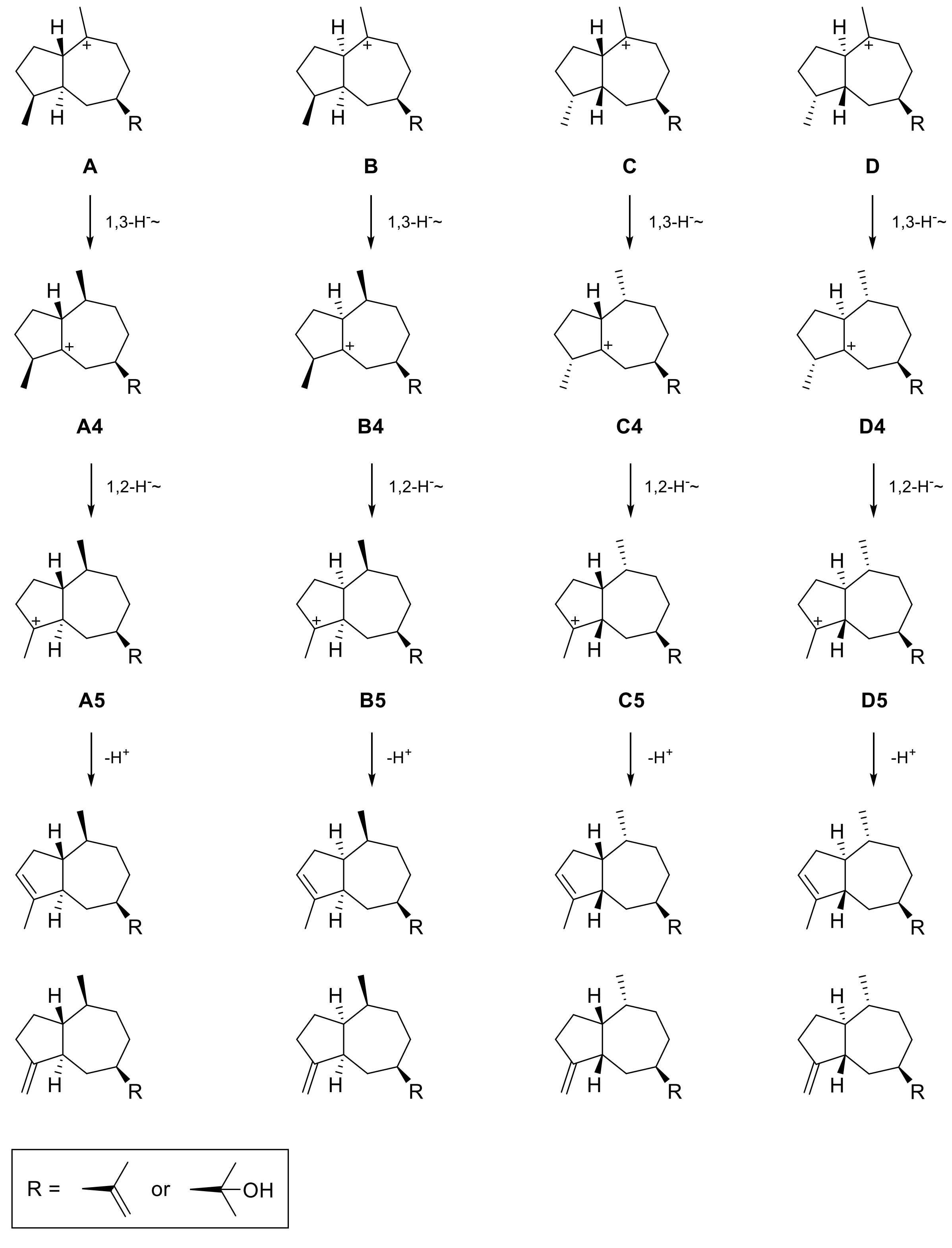

Scheme S13. Guaiane sesquiterpenes that are accessible from intermediates **A – D** through one 1,3-hydride shift, followed by one 1,2-hydride shift and deprotonation.

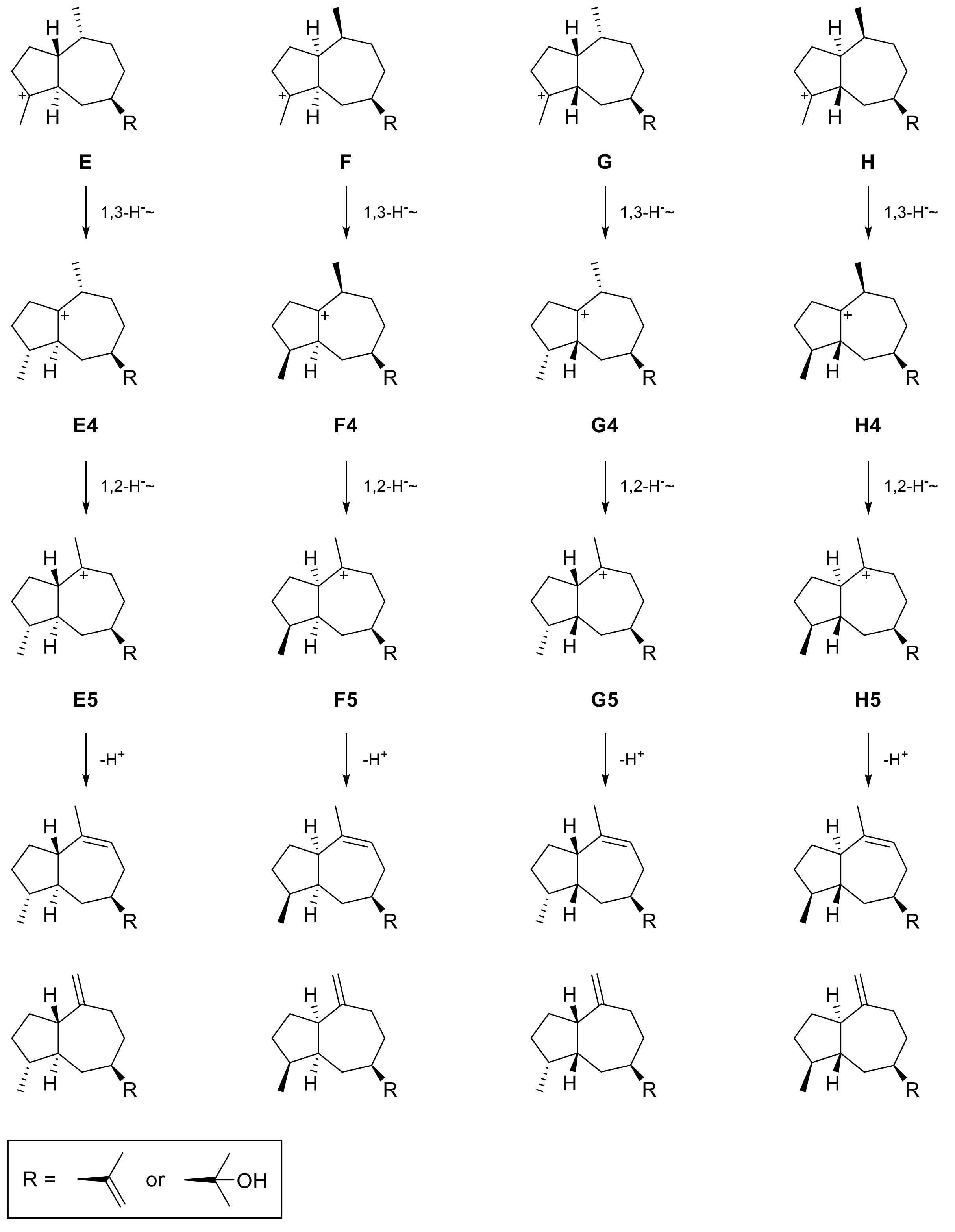

Scheme S14. Guaiane sesquiterpenes that are accessible from intermediates **E – H** through one 1,3-hydride shift, followed by one 1,2-hydride shift and deprotonation.

A)

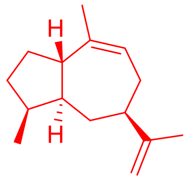

(1*S*,4*S*,5*S*,7*S*)-
guaia-9,11-diene

unknown

A (Scheme S1)

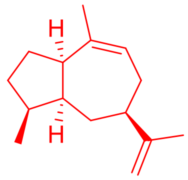

(1*R*,4*S*,5*S*,7*S*)-
guaia-9,11-diene

unknown

B (Scheme S1), **F** (Scheme S8)
F (Scheme S10)
F (Scheme S14)

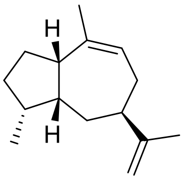

(1*S*,4*R*,5*R*,7*S*)-
guaia-9,11-diene

Peucedanum tauricum [1]

single report

C (Scheme S1), **G** (Scheme S8)
G (Scheme S10)
G (Scheme S14)

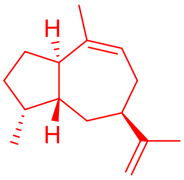

(1*R*,4*R*,5*R*,7*S*)-
guaia-9,11-diene

unknown

D (Scheme S1)

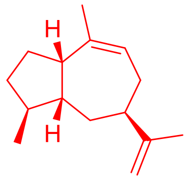

(1*S*,4*S*,5*R*,7*S*)-
guaia-9,11-diene

unknown

E (Scheme S8)
E (Scheme S10)

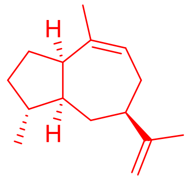

(1*R*,4*R*,5*S*,7*S*)-
guaia-9,11-diene

unknown

H (Scheme S8)
H (Scheme S10)

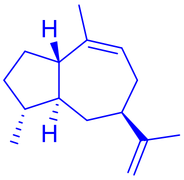

(1*S*,4*R*,5*S*,7*S*)-
guaia-9,11-diene

synthetic [2]

E (Scheme S14)

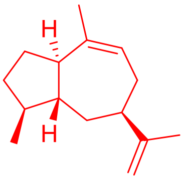

(1*R*,4*S*,5*R*,7*S*)-
guaia-9,11-diene

unknown

H (Scheme S14)

B)

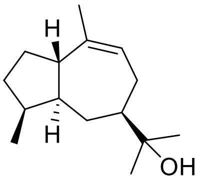

(1*S*,4*S*,5*S*,7*S*)-
guai-9-en-11-ol

Bulnesia sarmientoi [3]

single report

A (Scheme S1)

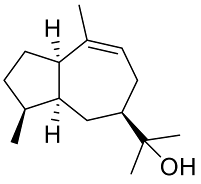

(1*R*,4*S*,5*S*,7*S*)-
guai-9-en-11-ol

Galbanum [4]

few reports

B (Scheme S1), **F** (Scheme S8)
F (Scheme S10)
F (Scheme S14)

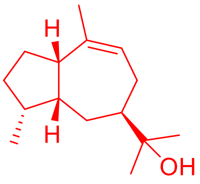

(1*S*,4*R*,5*R*,7*S*)-
guai-9-en-11-ol

unknown

C (Scheme S1), **G** (Scheme S8)
G (Scheme S10)
G (Scheme S14)

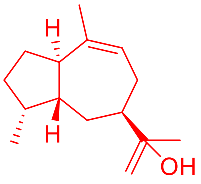

(1*R*,4*R*,5*R*,7*S*)-
guai-9-en-11-ol

unknown

D (Scheme S1)

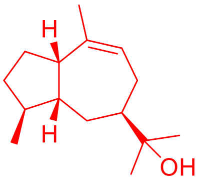

(1*S*,4*S*,5*R*,7*S*)-
guai-9-en-11-ol

unknown

E (Scheme S8)
E (Scheme S10)

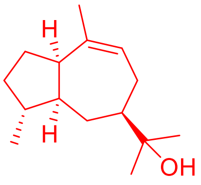

(1*R*,4*R*,5*S*,7*S*)-
guai-9-en-11-ol

unknown

H (Scheme S8)
H (Scheme S10)

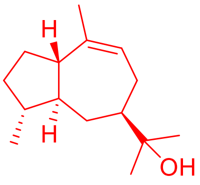

(1*S*,4*R*,5*S*,7*S*)-
guai-9-en-11-ol

unknown

E (Scheme S14)

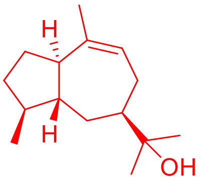

(1*R*,4*S*,5*R*,7*S*)-
guai-9-en-11-ol

unknown

H (Scheme S14)

Figure S1. Structures of all stereoisomers of guaia-9,11-diene and of guai-9-en-11-ol. Known natural products are shown in black, compounds only reported from synthetic work are in blue, and unknown compounds are in red. For each stereoisomer only one enantiomer is shown, representing the enantiomeric series typically isolated from plants (7*S* configuration). For each compound the potential precursor cation(s) **A** – **H** with reference to the relevant Scheme are given (colour code: black = possible, red = impossible, blue = possible through high barrier according to DFT calculations).

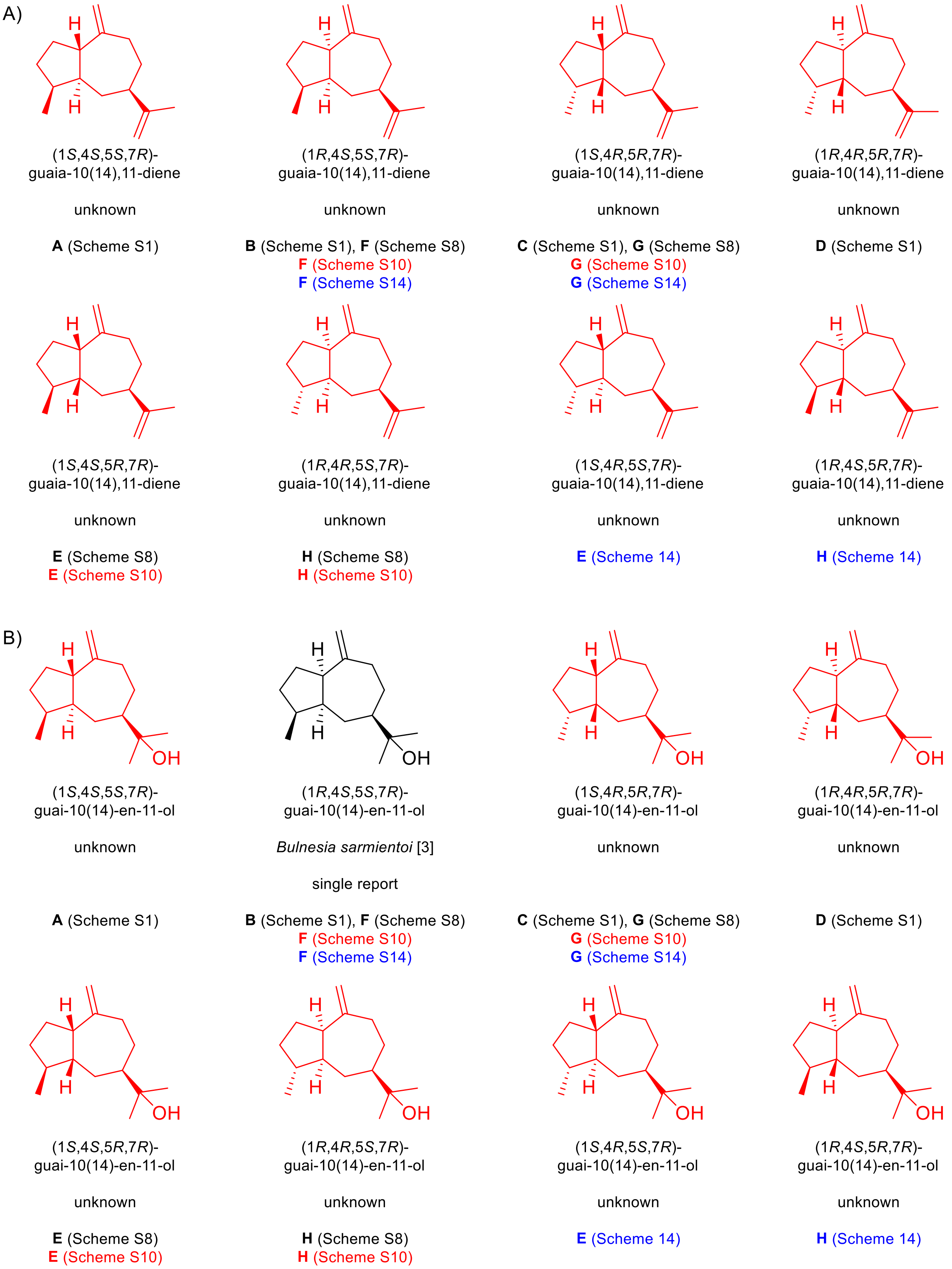

Figure S2. Structures of all stereoisomers of guaia-10(14),11-diene and of guai-10(14)-en-11-ol. Known natural products are shown in black and unknown compounds are in red. For each stereoisomer only one enantiomer is shown, representing the enantiomeric series typically isolated from plants (7*R* configuration). For each compound the potential precursor cation(s) **A** – **H** with reference to the relevant Scheme are given (colour code: black = possible, red = impossible, blue = possible through high barrier according to DFT calculations).

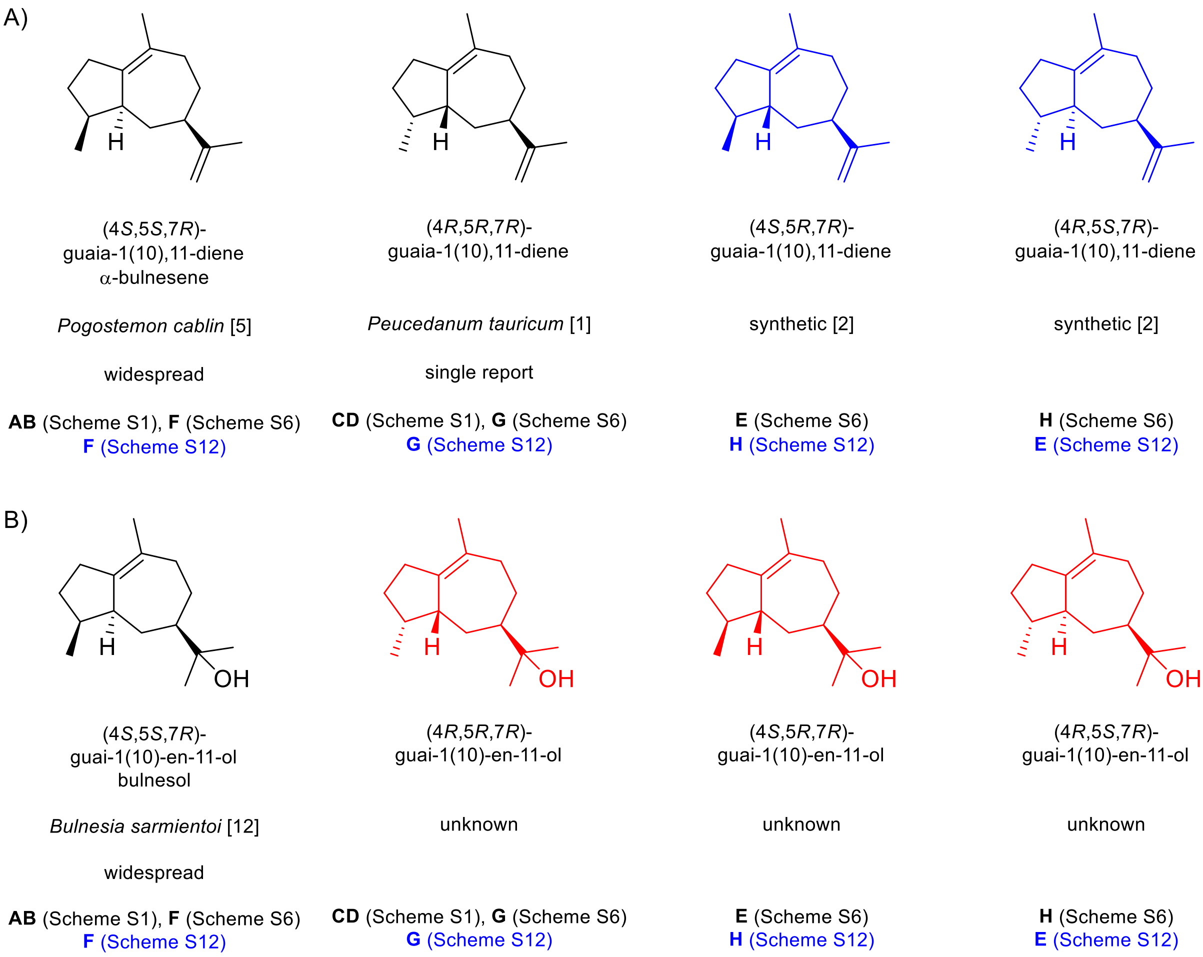

Figure S3. Structures of all stereoisomers of guaia-1(10),11-diene and of guai-1(10)-en-11-ol. Known natural products are shown in black, compounds only reported from synthetic work are in blue, and unknown compounds are in red. For each stereoisomer only one enantiomer is shown, representing the enantiomeric series typically isolated from plants (7*R* configuration). For each compound the potential precursor cation(s) **A** – **H** with reference to the relevant Scheme are given (colour code: black = possible, red = impossible, blue = possible through high barrier according to DFT calculations).

A)

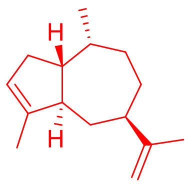

(1*R*,5*R*,7*R*,10*R*)-guaia-3,11-diene

unknown

E (Scheme S2)

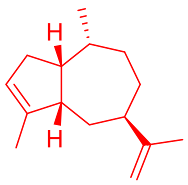

(1*R*,5*S*,7*R*,10*R*)-guaia-3,11-diene

unknown

G (Scheme S2), **C** (Scheme S7)
C (Scheme S9)
C (Scheme S13)

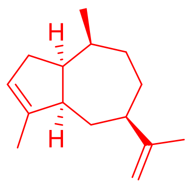

(1*S*,5*R*,7*R*,10*S*)-guaia-3,11-diene

unknown

F (Scheme S2), **B** (Scheme S7)
B (Scheme S9)
B (Scheme S13)

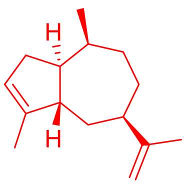

(1*S*,5*S*,7*R*,10*S*)-guaia-3,11-diene

unknown

H (Scheme S2)

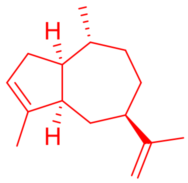

(1*S*,5*R*,7*R*,10*R*)-guaia-3,11-diene

unknown

A (Scheme S7)
A (Scheme S9)

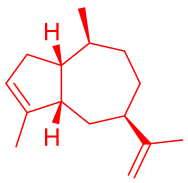

(1*R*,5*S*,7*R*,10*S*)-guaia-3,11-diene

unknown

D (Scheme S7)
D (Scheme S9)

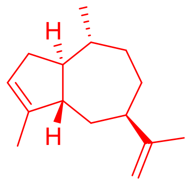

(1*S*,5*S*,7*R*,10*S*)-guaia-3,11-diene

unknown

D (Scheme S13)

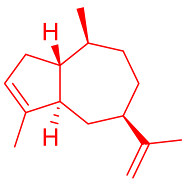

(1*R*,5*R*,7*R*,10*S*)-guaia-3,11-diene

unknown

A (Scheme S13)

B)

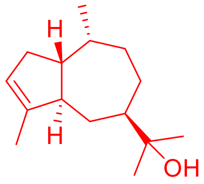

(1*R*,5*R*,7*R*,10*R*)-guai-3-en-11-ol

unknown

E (Scheme S2)

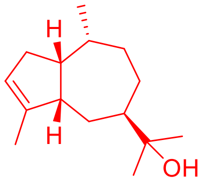

(1*R*,5*S*,7*R*,10*R*)-guai-3-en-11-ol

unknown

G (Scheme S2), **C** (Scheme S7)
C (Scheme S9)
C (Scheme S13)

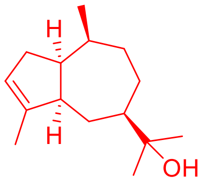

(1*S*,5*R*,7*R*,10*S*)-guai-3-en-11-ol

unknown

F (Scheme S2), **B** (Scheme S7)
B (Scheme S9)
B (Scheme S13)

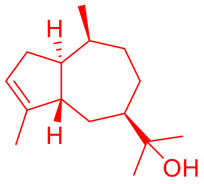

(1*S*,5*S*,7*R*,10*S*)-guai-3-en-11-ol

unknown

H (Scheme S2)

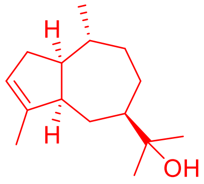

(1*S*,5*R*,7*R*,10*R*)-guai-3-en-11-ol

unknown

A (Scheme S7)
A (Scheme S9)

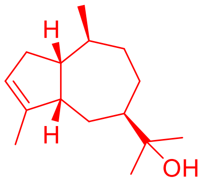

(1*R*,5*S*,7*R*,10*S*)-guai-3-en-11-ol

unknown

D (Scheme S7)
D (Scheme S9)

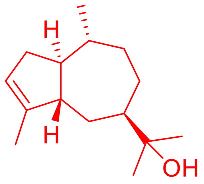

(1*S*,5*S*,7*R*,10*S*)-guai-3-en-11-ol

unknown

D (Scheme S13)

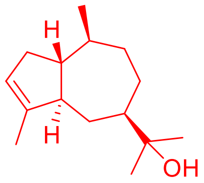

(1*R*,5*R*,7*R*,10*S*)-guai-3-en-11-ol

unknown

A (Scheme S13)

Figure S4. Structures of all stereoisomers of guaia-3,11-diene and of guai-3-en-11-ol. No natural products from this group have been reported so far. For each stereoisomer only one enantiomer is shown, representing the enantiomeric series typically isolated from plants (7*R* configuration). For each compound the potential precursor cation(s) **A** – **H** with reference to the relevant Scheme are given (colour code: black = possible, red = impossible, blue = possible through high barrier according to DFT calculations).

A)

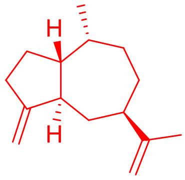

(1*R*,5*R*,7*R*,10*R*)-
guaia-4(15),11-diene

unknown

E (Scheme S2)

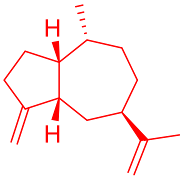

(1*R*,5*S*,7*R*,10*R*)-
guaia-4(15),11-diene

unknown

G (Scheme S2), **C** (Scheme S7)
C (Scheme S9)
C (Scheme S13)

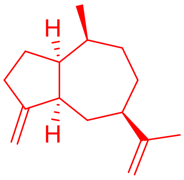

(1*S*,5*R*,7*R*,10*S*)-
guaia-4(15),11-diene

unknown

F (Scheme S2), **B** (Scheme S7)
B (Scheme S9)
B (Scheme S13)

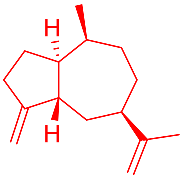

(1*S*,5*S*,7*R*,10*S*)-
guaia-4(15),11-diene

unknown

H (Scheme S2)

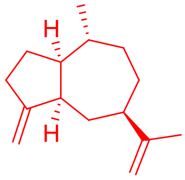

(1*S*,5*R*,7*R*,10*R*)-
guaia-4(15),11-diene

unknown

A (Scheme S7)
A (Scheme S9)

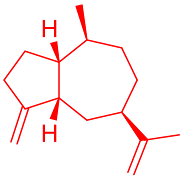

(1*R*,5*S*,7*R*,10*S*)-
guaia-4(15),11-diene

unknown

D (Scheme S7)
D (Scheme S9)

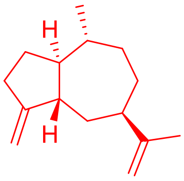

(1*S*,5*S*,7*R*,10*S*)-
guaia-4(15),11-diene

unknown

D (Scheme S13)

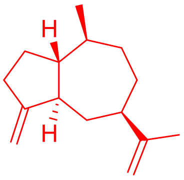

(1*R*,5*R*,7*R*,10*S*)-
guaia-4(15),11-diene

unknown

A (Scheme S13)

B)

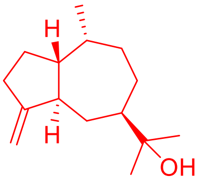

(1*R*,5*R*,7*R*,10*R*)-
guai-4(15)-en-11-ol

unknown

E (Scheme S2)

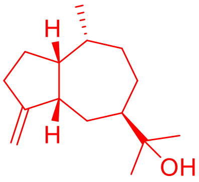

(1*R*,5*S*,7*R*,10*R*)-
guai-4(15)-en-11-ol

unknown

G (Scheme S2), **C** (Scheme S7)
C (Scheme S9)
C (Scheme S13)

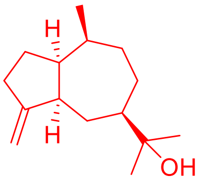

(1*S*,5*R*,7*R*,10*S*)-
guai-4(15)-en-11-ol

unknown

F (Scheme S2), **B** (Scheme S7)
B (Scheme S9)
B (Scheme S13)

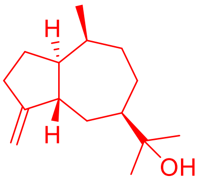

(1*S*,5*S*,7*R*,10*S*)-
guai-4(15)-en-11-ol

unknown

H (Scheme S2)

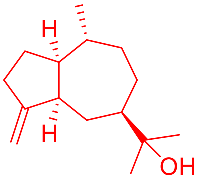

(1*S*,5*R*,7*R*,10*R*)-
guai-4(15)-en-11-ol

unknown

A (Scheme S7)
A (Scheme S9)

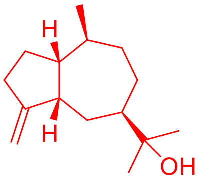

(1*R*,5*S*,7*R*,10*S*)-
guai-4(15)-en-11-ol

unknown

D (Scheme S7)
D (Scheme S9)

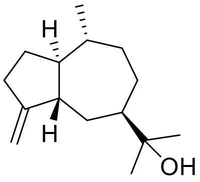

(1*S*,5*S*,7*R*,10*R*)-
guai-4(15)-en-11-ol

Populus trichocarpa [7]

single report

D (Scheme S13)

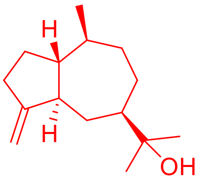

(1*R*,5*R*,7*R*,10*S*)-
guai-4(15)-en-11-ol

unknown

A (Scheme S13)

Figure S5. Structures of all stereoisomers of guaia-4(15),11-diene and of guai-4(15)-en-11-ol. Known natural products are shown in black and unknown compounds are in red. For each stereoisomer only one enantiomer is shown, representing the enantiomeric series typically isolated from plants (7*R* configuration). For each compound the potential precursor cation(s) **A** – **H** with reference to the relevant Scheme are given (colour code: black = possible, red = impossible, blue = possible through high barrier according to DFT calculations).

A)

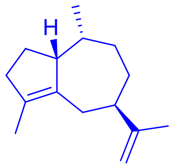

(1*S*,7*R*,10*S*)-
guaia-4,11-diene

synthetic [8]

EG (Scheme S2), **C** (Scheme S5)
C (Scheme S11)

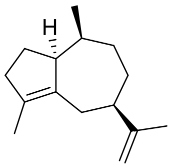

(1*R*,7*R*,10*R*)-
guaia-4,11-diene
aciphyllene

Lindera glauca [9]
structural revision [8]

widespread

FH (Scheme S2), **B** (Scheme S5)
B (Scheme S11)

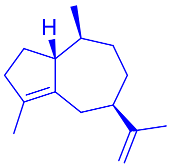

(1*S*,7*R*,10*R*)-
guaia-4,11-diene

synthetic [10]

D (Scheme S5), **A** (Scheme S11)

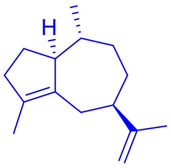

(1*R*,7*R*,10*S*)-
guaia-4,11-diene

synthetic [8]

A (Scheme S5), **D** (Scheme S11)

B)

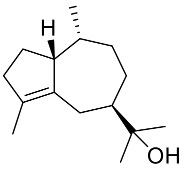

(1*S*,7*R*,10*S*)-
guai-4-en-11-ol

Bulnesia sarmientoi [3]

few reports

EG (Scheme S2), **C** (Scheme S5)
C (Scheme S11)

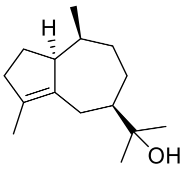

(1*R*,7*R*,10*R*)-
guai-4-en-11-ol

Pittosporum undulatum [11]

few reports

FH (Scheme S2), **B** (Scheme S5)
B (Scheme S11)

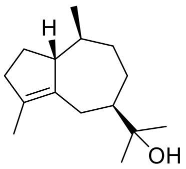

(1*S*,7*R*,10*R*)-
guai-4-en-11-ol

Bulnesia sarmientoi [3]

single report

D (Scheme S5), **A** (Scheme S11)

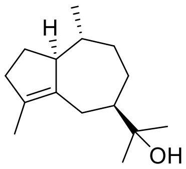

(1*R*,7*R*,10*S*)-
guai-4-en-11-ol

Bulnesia sarmientoi [3]
Populus trichocarpa [7]

few reports

A (Scheme S5), **D** (Scheme S11)

Figure S6. Structures of all stereoisomers of guaia-4,11-diene and of guai-4-en-11-ol. Known natural products are shown in black and compounds only reported from synthetic work are in blue. All stereoisomers from this group are known. For each stereoisomer only one enantiomer is shown, representing the enantiomeric series typically isolated from plants (7*R* configuration). For each compound the potential precursor cation(s) **A** – **H** with reference to the relevant Scheme are given (colour code: black = possible, red = impossible, blue = possible through high barrier according to DFT calculations).

A)

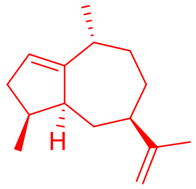

(4*S*,5*S*,7*R*,10*R*)-guaia-1,11-diene

unknown

A (Scheme S3)

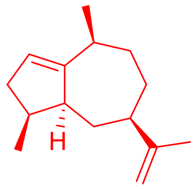

(4*S*,5*S*,7*R*,10*S*)-guaia-1,11-diene

unknown

B (Scheme S3), **F** (Scheme S6)
F (Scheme S12)

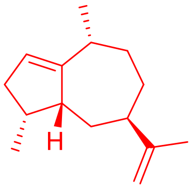

(4*R*,5*R*,7*R*,10*R*)-guaia-1,11-diene

unknown

C (Scheme S3), **G** (Scheme S6)
G (Scheme S12)

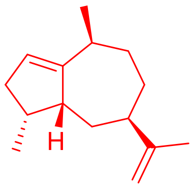

(4*R*,5*R*,7*R*,10*S*)-guaia-1,11-diene

unknown

D (Scheme S3)

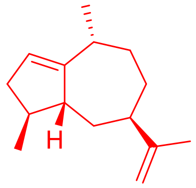

(4*S*,5*R*,7*R*,10*R*)-guaia-1,11-diene

unknown

E (Scheme S6)

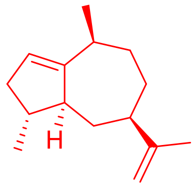

(4*R*,5*S*,7*R*,10*S*)-guaia-1,11-diene

unknown

H (Scheme S6)

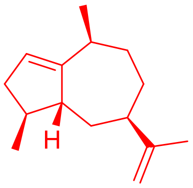

(4*S*,5*R*,7*R*,10*S*)-guaia-1,11-diene

unknown

H (Scheme S12)

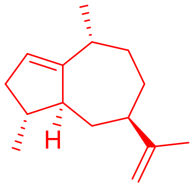

(4*R*,5*S*,7*R*,10*R*)-guaia-1,11-diene

unknown

E (Scheme S12)

B)

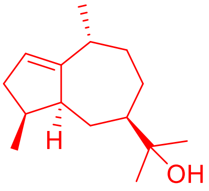

(4*S*,5*S*,7*R*,10*R*)-guai-1-en-11-ol

unknown

A (Scheme S3)

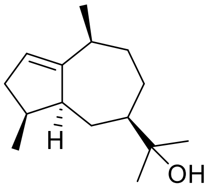

(4*S*,5*S*,7*R*,10*S*)-guai-1-en-11-ol

Hyptis suaveolens [12]

single report

B (Scheme S3), **F** (Scheme S6)
F (Scheme S12)

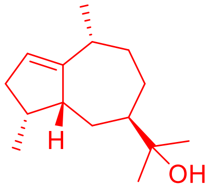

(4*R*,5*R*,7*R*,10*R*)-guai-1-en-11-ol

unknown

C (Scheme S3), **G** (Scheme S6)
G (Scheme S12)

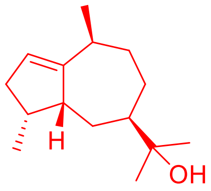

(4*R*,5*R*,7*R*,10*S*)-guai-1-en-11-ol

unknown

D (Scheme S3)

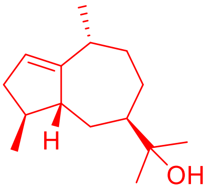

(4*S*,5*R*,7*R*,10*R*)-guai-1-en-11-ol

unknown

E (Scheme S6)

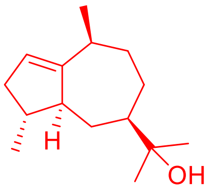

(4*R*,5*S*,7*R*,10*S*)-guai-1-en-11-ol

unknown

H (Scheme S6)

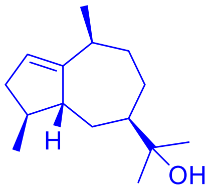

(4*S*,5*R*,7*R*,10*S*)-guai-1-en-11-ol

synthetic [13]

H (Scheme S12)

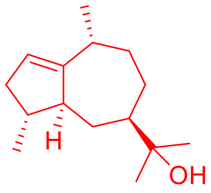

(4*R*,5*S*,7*R*,10*R*)-guai-1-en-11-ol

unknown

E (Scheme S12)

Figure S7. Structures of all stereoisomers of guaia-1,11-diene and of guai-1-en-11-ol. Known natural products are shown in black, compounds only reported from synthetic work are in blue, and unknown compounds are in red. For each stereoisomer only one enantiomer is shown, representing the enantiomeric series typically isolated from plants (7*R* configuration). For each compound the potential precursor cation(s) **A** – **H** with reference to the relevant Scheme are given (colour code: black = possible, red = impossible, blue = possible through high barrier according to DFT calculations).

A)

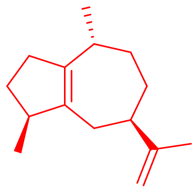

(4*S*,7*R*,10*R*)-
guaia-1(5),11-diene

unknown

A (Scheme S3)
E (Scheme S4)

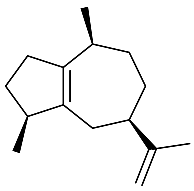

(4*S*,7*R*,10*S*)-
guaia-1(5),11-diene
α-guaiene

Pogostemon cablin [14]

widespread

B (Scheme S3)
F (Scheme S4)

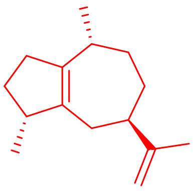

(4*R*,7*R*,10*R*)-
guaia-1(5),11-diene

unknown

C (Scheme S3)
G (Scheme S4)

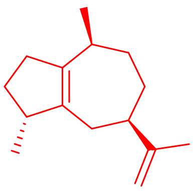

(4*S*,7*R*,10*S*)-
guaia-1(5),11-diene

unknown

D (Scheme S3)
H (Scheme S4)

B)

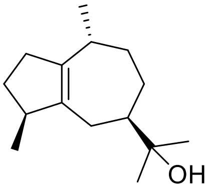

(4*S*,7*R*,10*R*)-
guai-1(5)-en-11-ol
10-*epi*-guaiol

Bulnesia sarmientoi [3]

single report

A (Scheme S3)
E (Scheme S4)

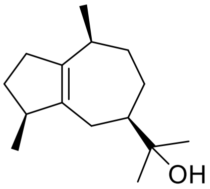

(4*S*,7*R*,10*S*)-
guai-1(5)-en-11-ol
guaiol

Guaiacum [15]

widespread

B (Scheme S3)
F (Scheme S4)

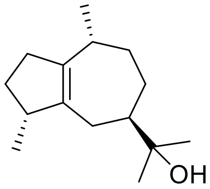

(4*R*,7*R*,10*R*)-
guai-1(5)-en-11-ol
4,10-*diepi*-guaiol

Boswellia occulta [16]

single report

C (Scheme S3)
G (Scheme S4)

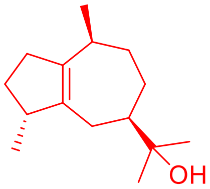

(4*S*,7*R*,10*S*)-
guai-1(5)-en-11-ol

unknown

D (Scheme S3)
H (Scheme S4)

Figure S8. Structures of all stereoisomers of guaia-1(10),11-diene and of guai-1(10)-en-11-ol. Known natural products are shown in black and unknown compounds are in red. For each stereoisomer only one enantiomer is shown, representing the enantiomeric series typically isolated from plants (7*R* configuration). For each compound the potential precursor cation(s) **A** – **H** with reference to the relevant Scheme are given (colour code: black = possible, red = impossible, blue = possible through high barrier according to DFT calculations).

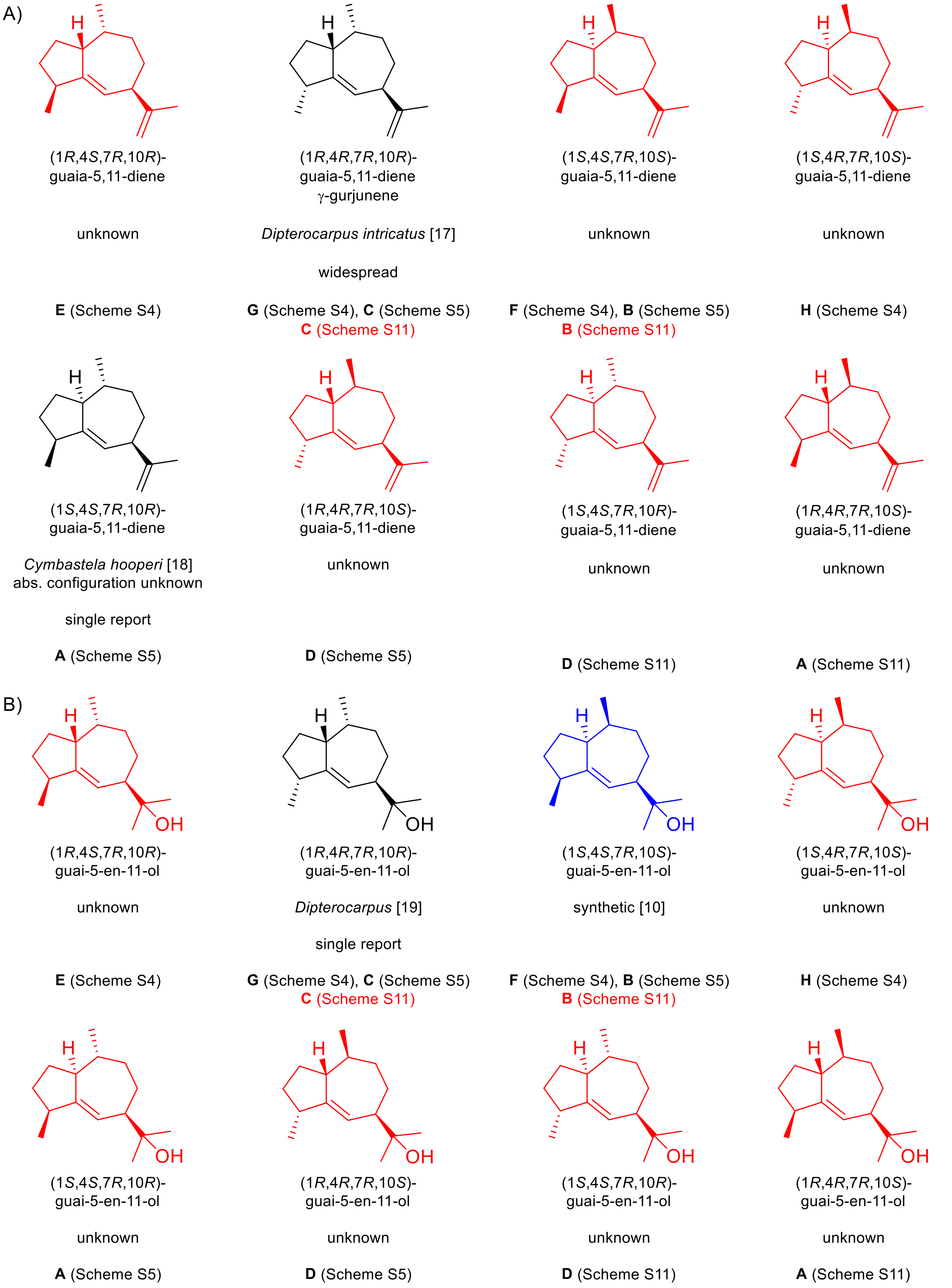

Figure S9. Structures of all stereoisomers of guaia-5,11-diene and of guai-5-en-11-ol. Known natural products are shown in black, compounds only reported from synthetic work are in blue, and unknown compounds are in red. For each stereoisomer only one enantiomer is shown, representing the enantiomeric series typically isolated from plants (7*R* configuration). For each compound the potential precursor cation(s) **A** – **H** with reference to the relevant Scheme are given (colour code: black = possible, red = impossible, blue = possible through high barrier according to DFT calculations).

A)

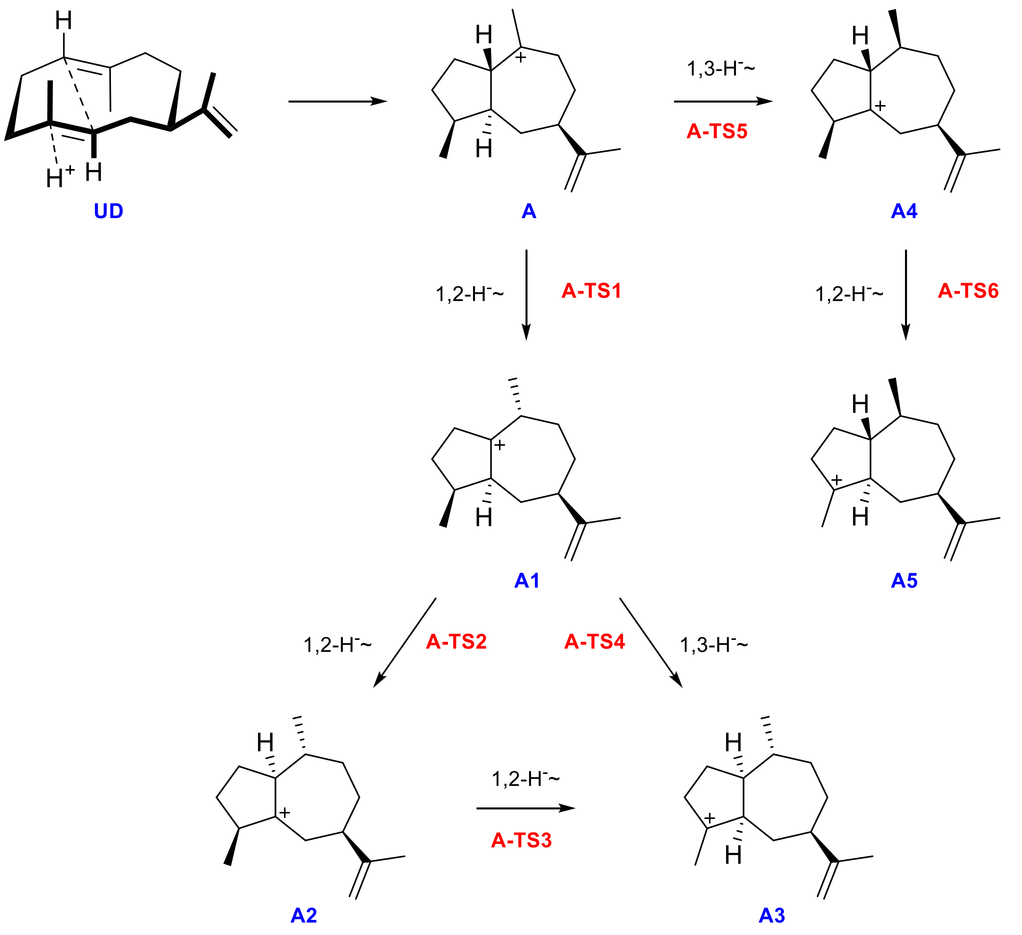

B)

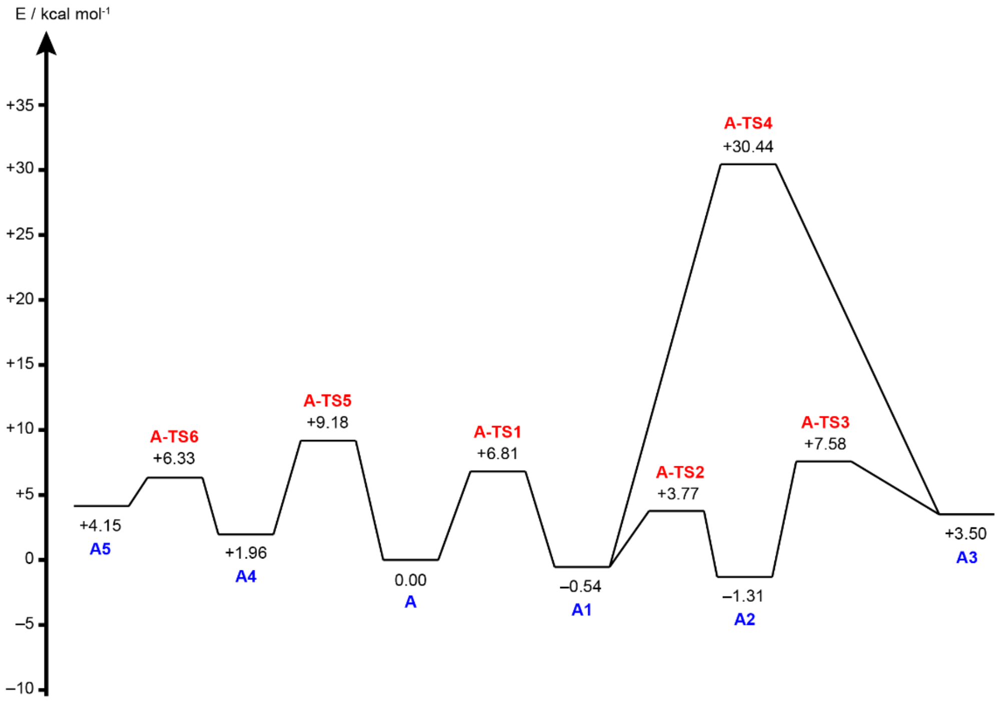

Figure S10. DFT calculations for **A** series from germacrene A. A) Hydride shifts starting from **A** (in **A**: H1 on top, H4 and H5 on bottom = TBB, pseudoenantiomeric to **D** series). B) Energy profile for the terpene biosynthetic steps (Gibbs energies, 298 K, mPW1PW91/6-311+G(d,p)//B97D3/6-31g(d,p)).

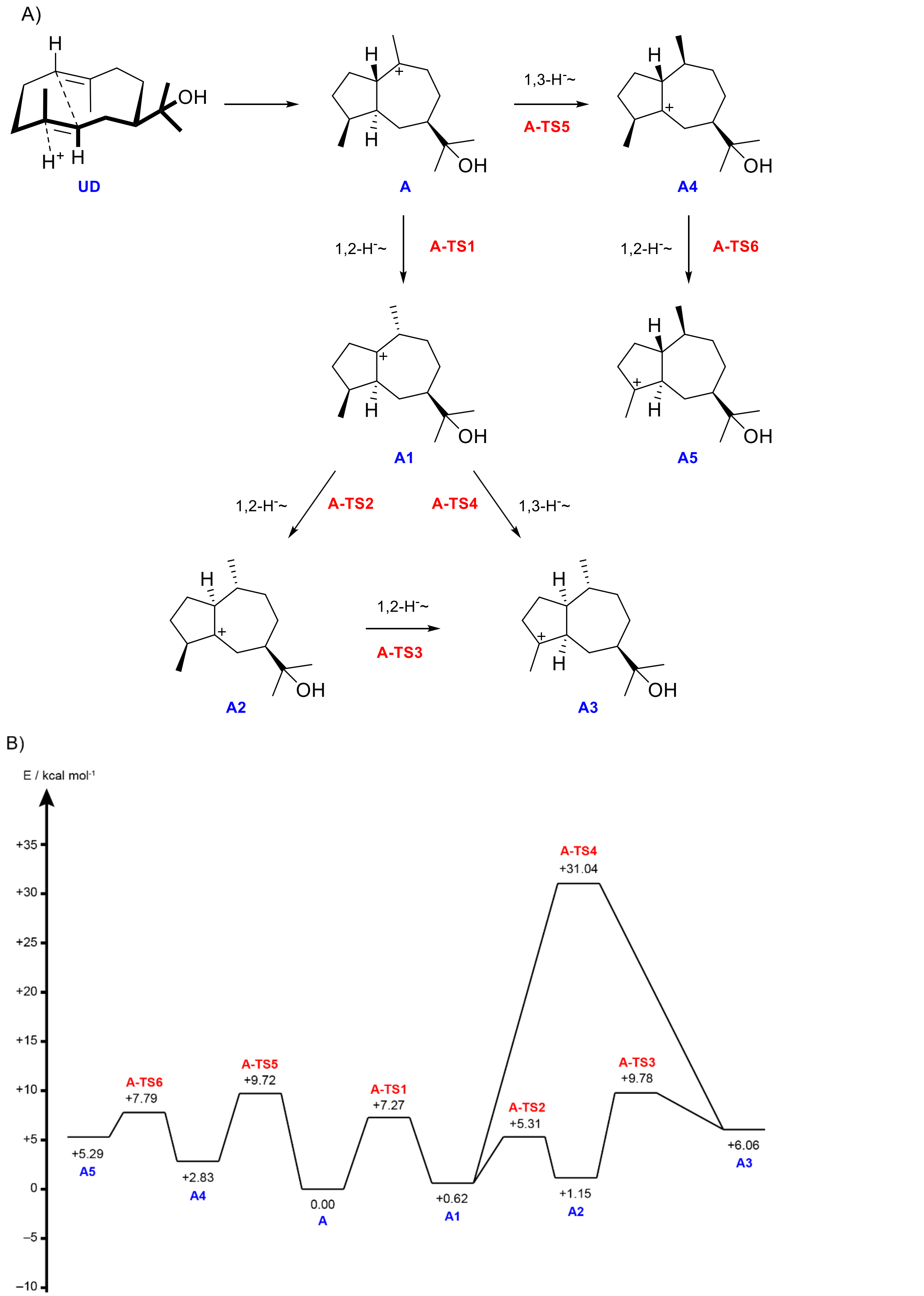

Figure S11. DFT calculations for **A** series from hedycaryol. A) Hydride shifts starting from **A** (in **A**: H1 on top, H4 and H5 on bottom = TBB, pseudoenantiomeric to **D** series). B) Energy profile for the terpene biosynthetic steps (Gibbs energies, 298 K, mPW1PW91/6-311+G(d,p)//B97D3/6-31g(d,p)).

A)

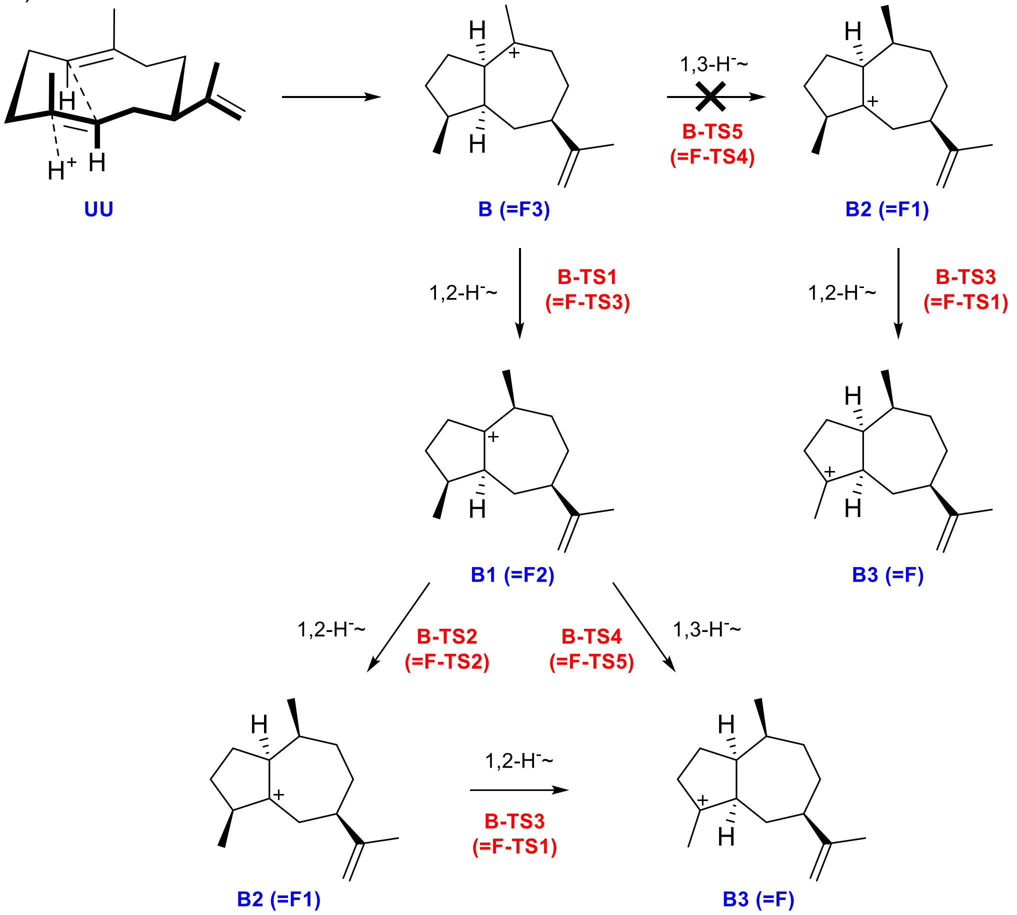

B)

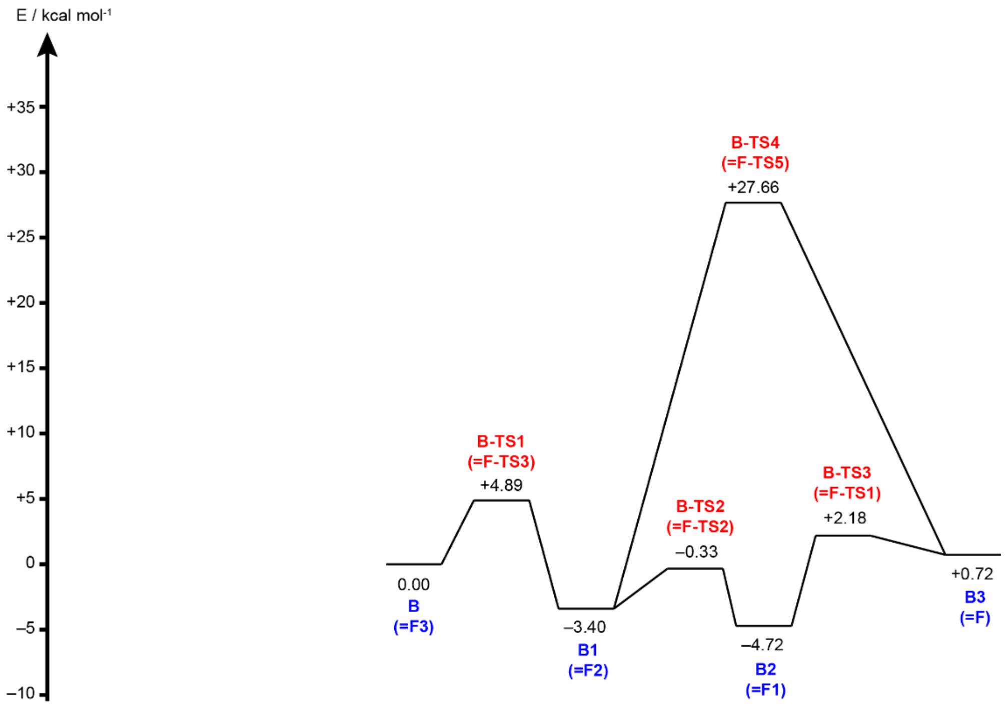

Figure S12. DFT calculations for **B** series from germacrene A. A) Hydride shifts starting from **B** (in **B**: H1, H4 and H5 on bottom = BBB, intermediates identical to those in **F** series, pseudoenantiomeric to those in **C** and **G** series). B) Energy profile for the terpene biosynthetic steps (Gibbs energies, 298 K, mPW1PW91/6-311+G(d,p)//B97D3/6-31g(d,p)).

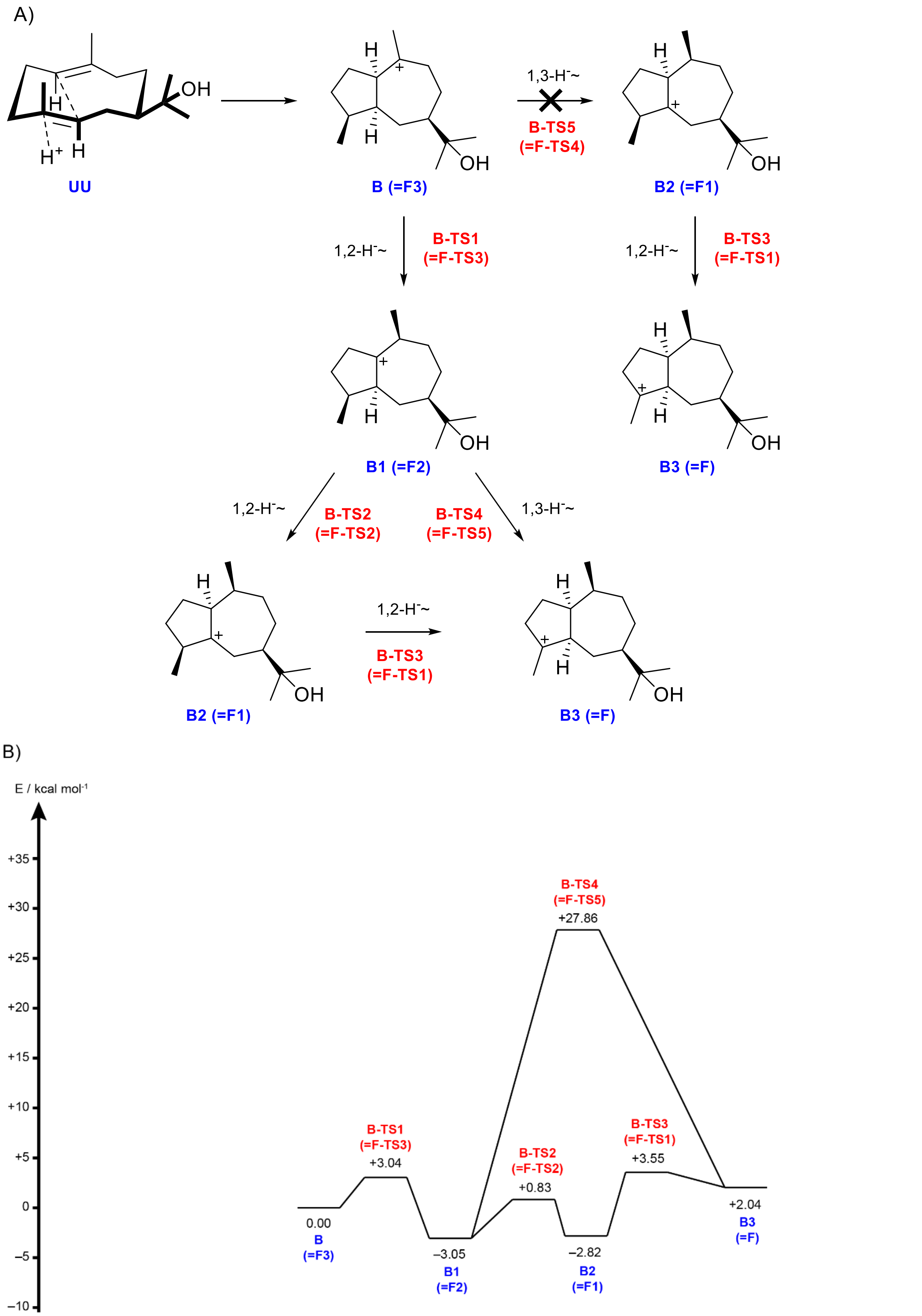

Figure S13. DFT calculations for **B** series from hedycaryol. A) Hydride shifts starting from **B** (in **B**: H1, H4 and H5 on bottom = BBB, intermediates identical to those in **F** series, pseudoenantiomeric to those in **C** and **G** series). B) Energy profile for the terpene biosynthetic steps (Gibbs energies, 298 K, mPW1PW91/6-311+G(d,p)//B97D3/6-31g(d,p)).

A)

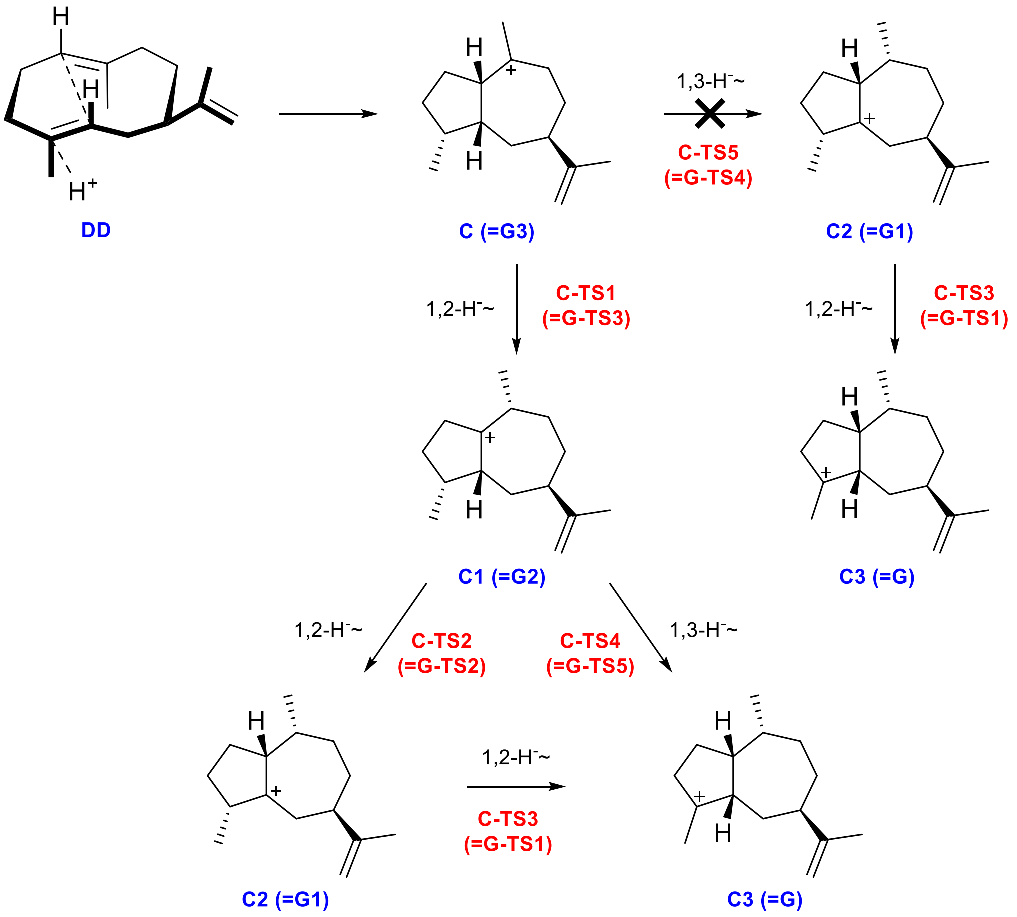

B)

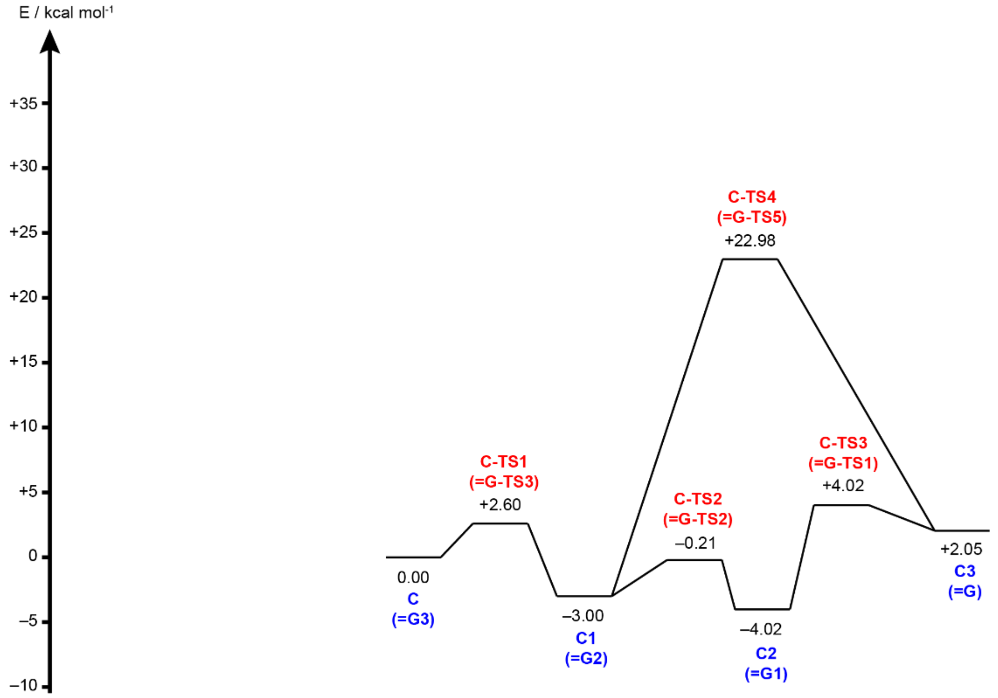

Figure S14. DFT calculations for **C** series from germacrene A. A) Hydride shifts starting from **C** (in **C**: H1, H4 and H5 on top = TTT, intermediates identical to those in **G** series, pseudoenantiomeric to those in **B** and **F** series). B) Energy profile for the terpene biosynthetic steps (Gibbs energies, 298 K, mPW1PW91/6-311+G(d,p)//B97D3/6-31g(d,p)).

A)

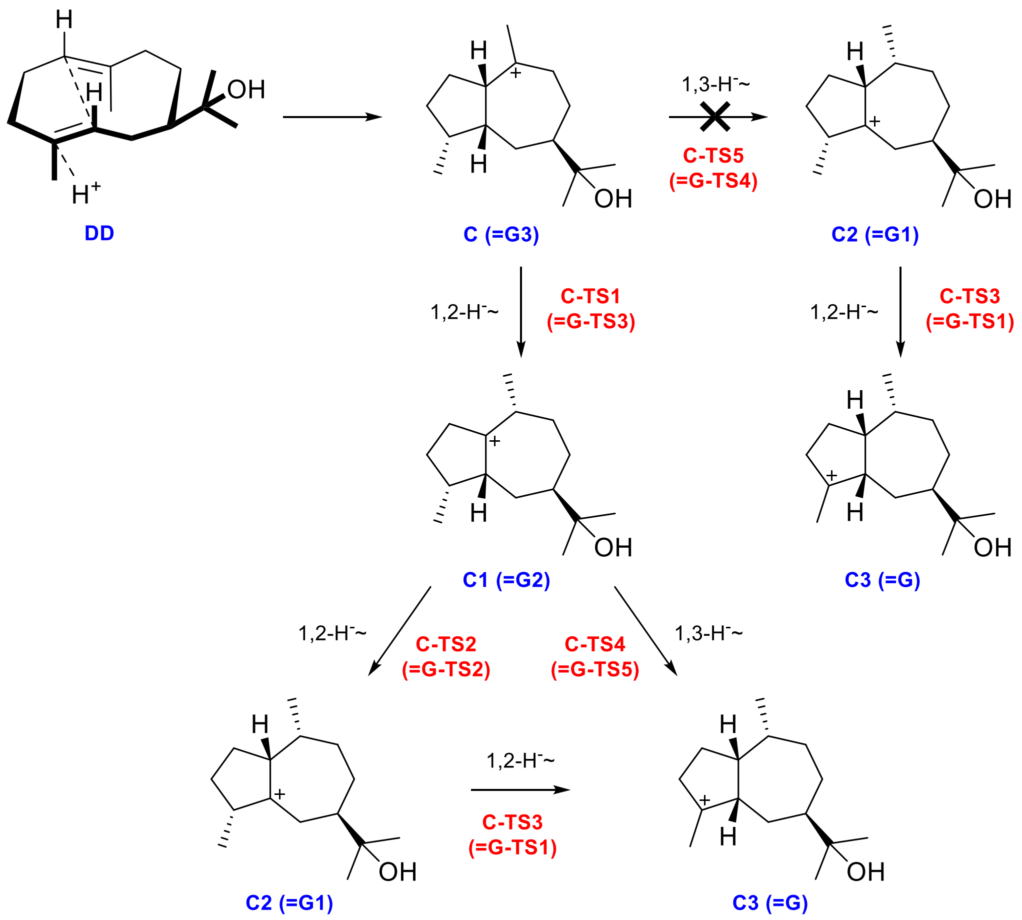

B)

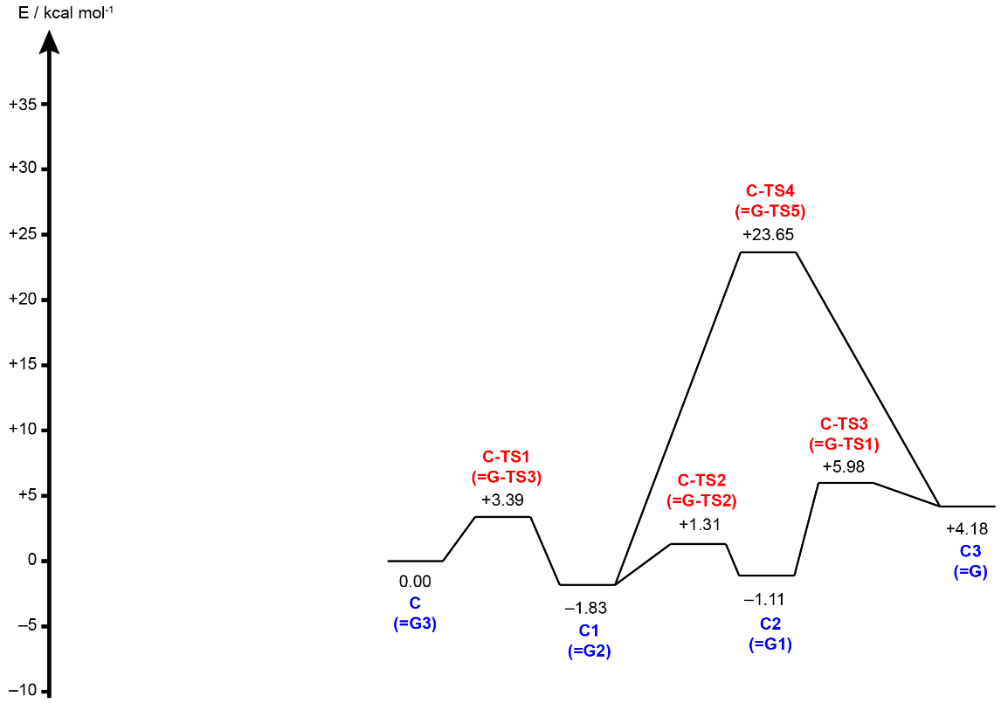

Figure S15. DFT calculations for **C** series from hedycaryol. A) Hydride shifts starting from **C** (in **C**: H1, H4 and H5 on top = TTT, intermediates dentical to those in **G** series, pseudoenantiomeric to those in **B** and **F** series). B) Energy profile for the terpene biosynthetic steps (Gibbs energies, 298 K, mPW1PW91/6-311+G(d,p)//B97D3/6-31g(d,p)).

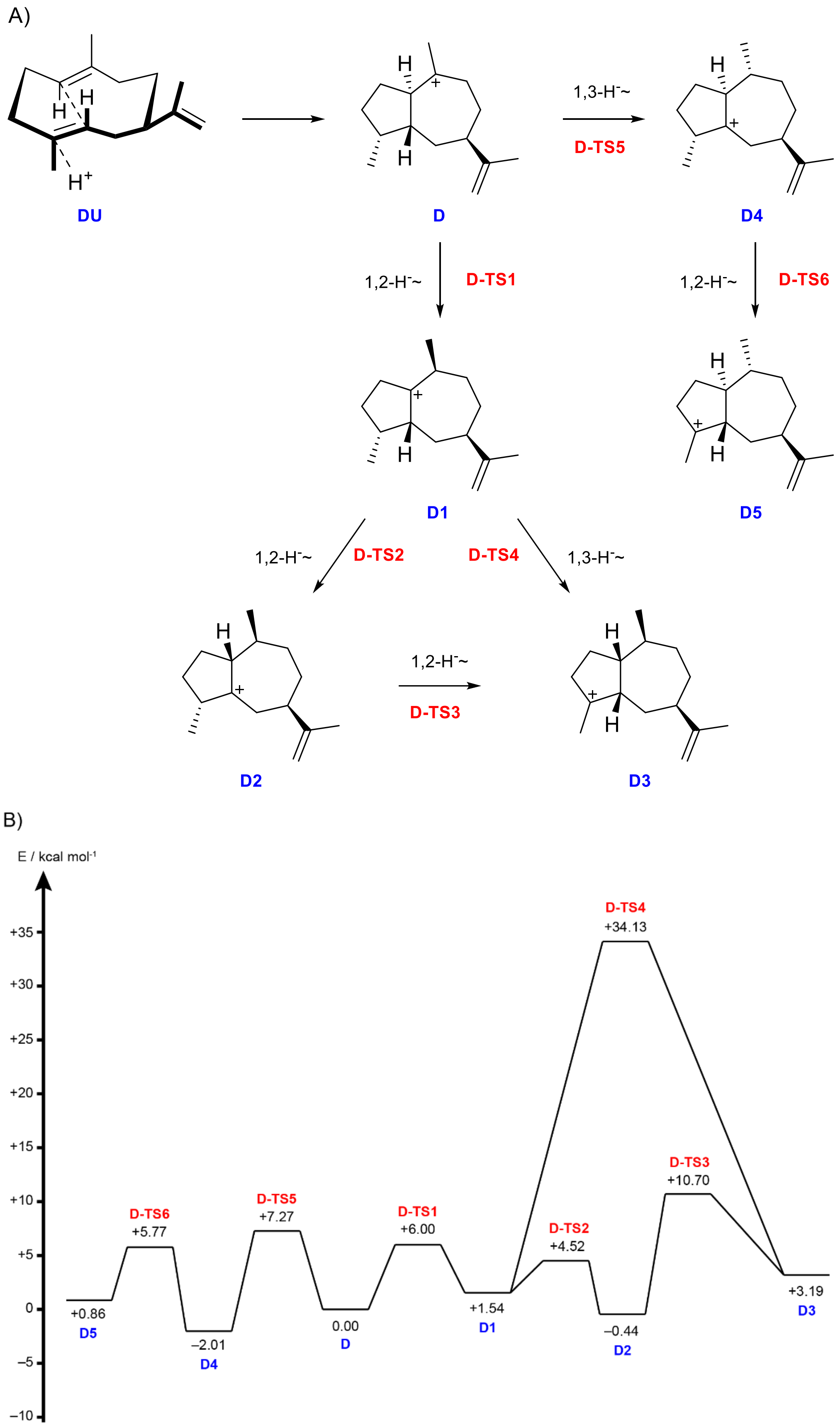

Figure S16. DFT calculations for **D** series from germacrene A. A) Hydride shifts starting from **D** (in **D**: H1 on bottom, H4 and H5 on top = BTT, pseudoenantiomeric to **A** series). B) Energy profile for the terpene biosynthetic steps (Gibbs energies, 298 K, mPW1PW91/6-311+G(d,p)//B97D3/6-31g(d,p)).

A)

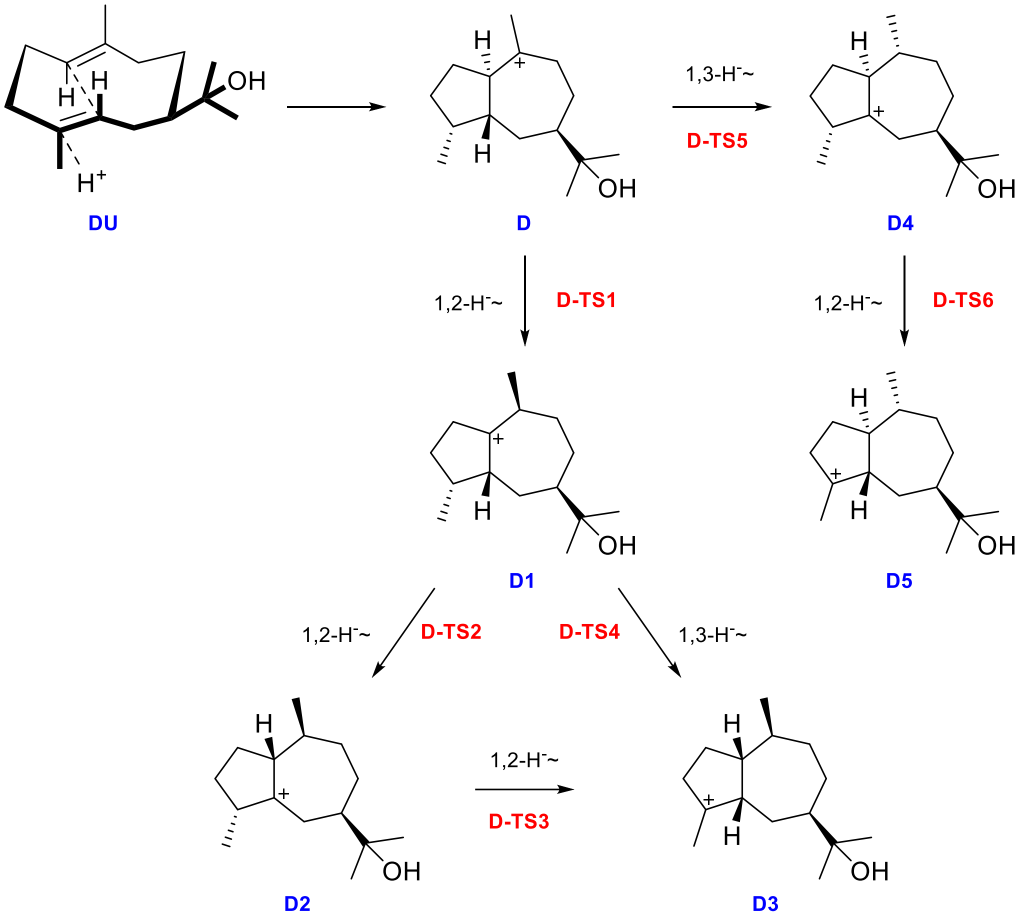

B)

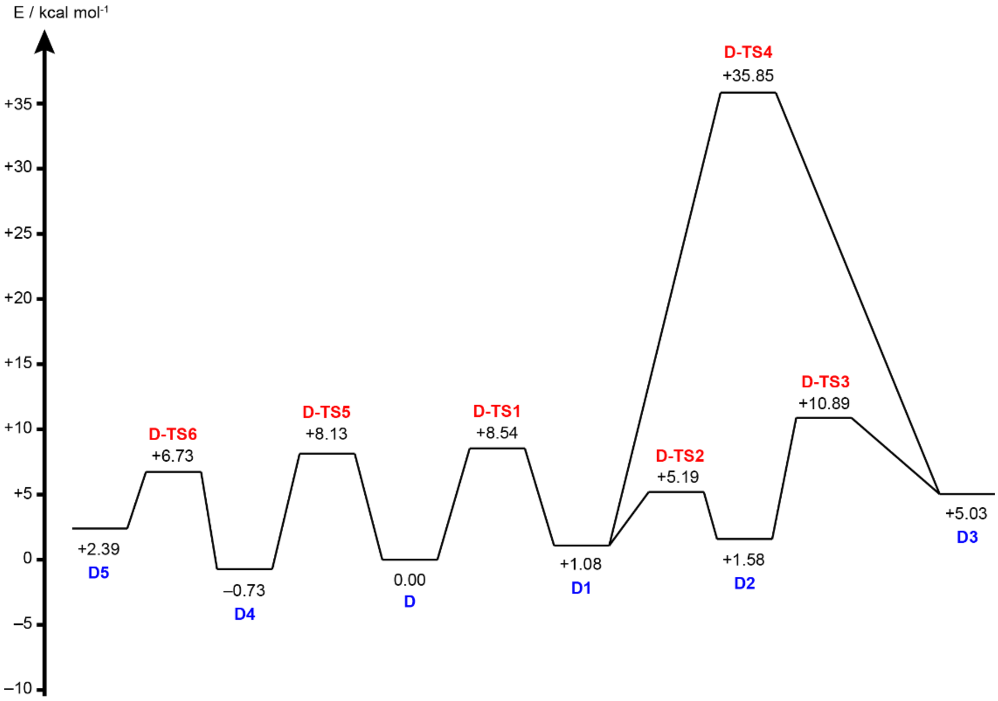

Figure S17. DFT calculations for **D** series from hedycaryol. A) Hydride shifts starting from **D** (in **D**: H1 on bottom, H4 and H5 on top = BTT, pseudoenantiomeric to **A** series). B) Energy profile for the terpene biosynthetic steps (Gibbs energies, 298 K, mPW1PW91/6-311+G(d,p)//B97D3/6-31g(d,p)).

A)

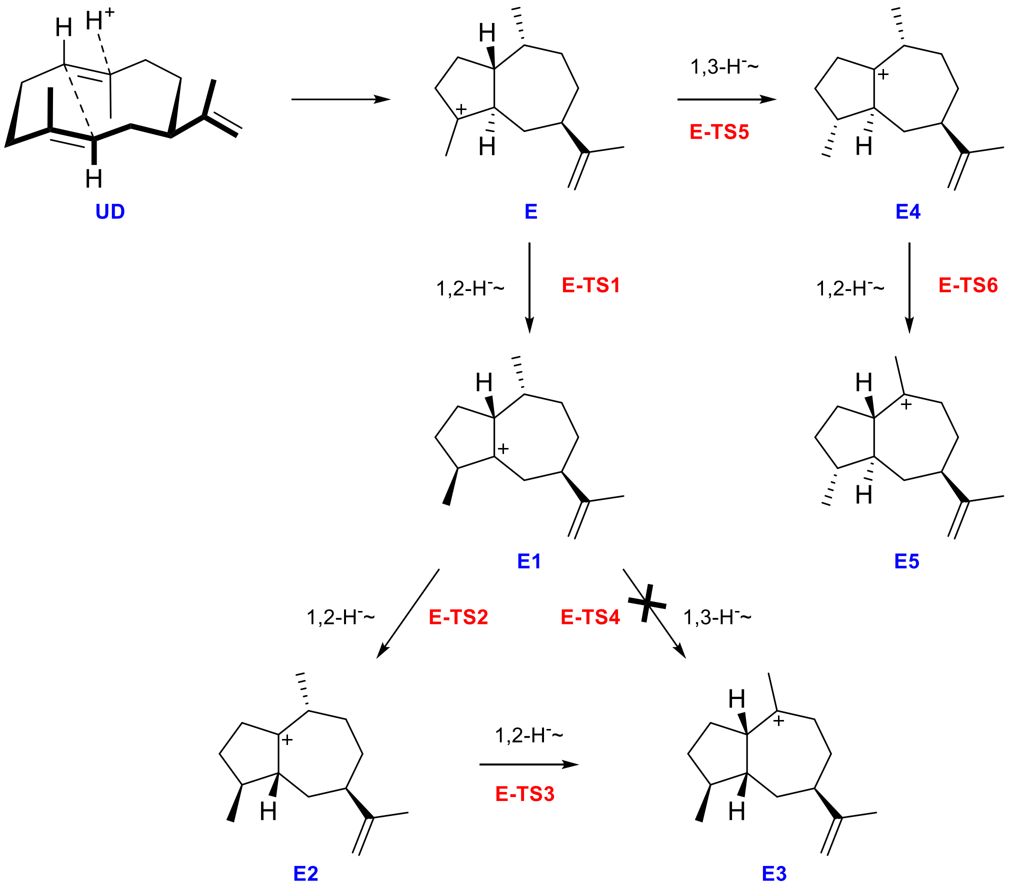

B)

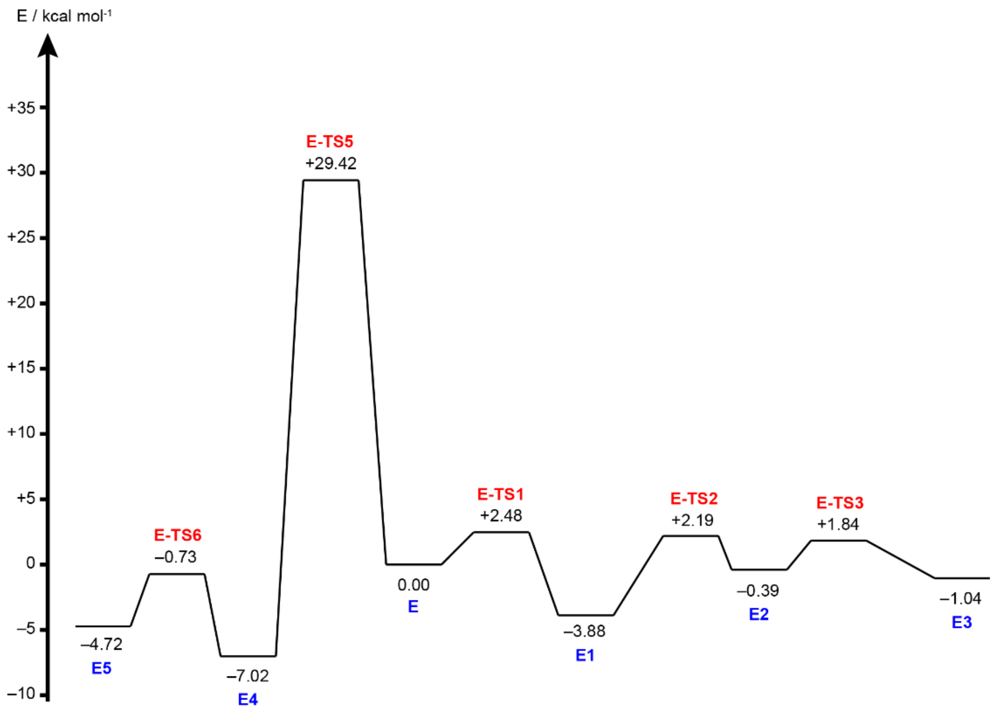

Figure S18. DFT calculations for **E** series from germacrene A. A) Hydride shifts starting from **E** (in **E3**: H1 on top, H4 on bottom, H5 on top = TBT, pseudoenantiomeric to **H** series). B) Energy profile for the terpene biosynthetic steps (Gibbs energies, 298 K, mPW1PW91/6-311+G(d,p)//B97D3/6-31g(d,p)).

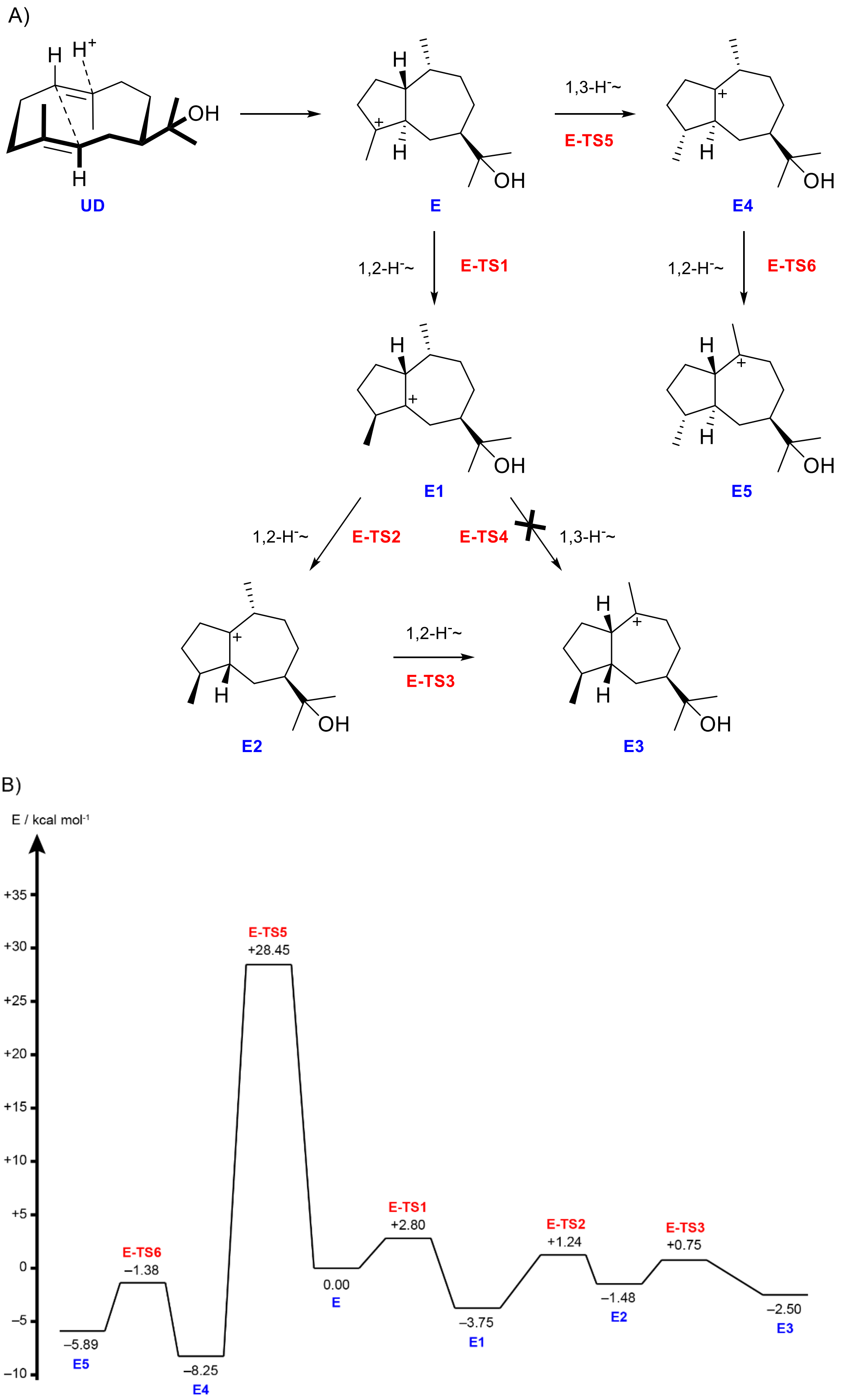

Figure S19. DFT calculations for **E** series from hedycaryol. A) Hydride shifts starting from **E** (in **E3**: H1 on top, H4 on bottom, H5 on top = TBT, pseudoenantiomeric to **H** series). B) Energy profile for the terpene biosynthetic steps (Gibbs energies, 298 K, mPW1PW91/6-311+G(d,p)//B97D3/6-31g(d,p)).

A)

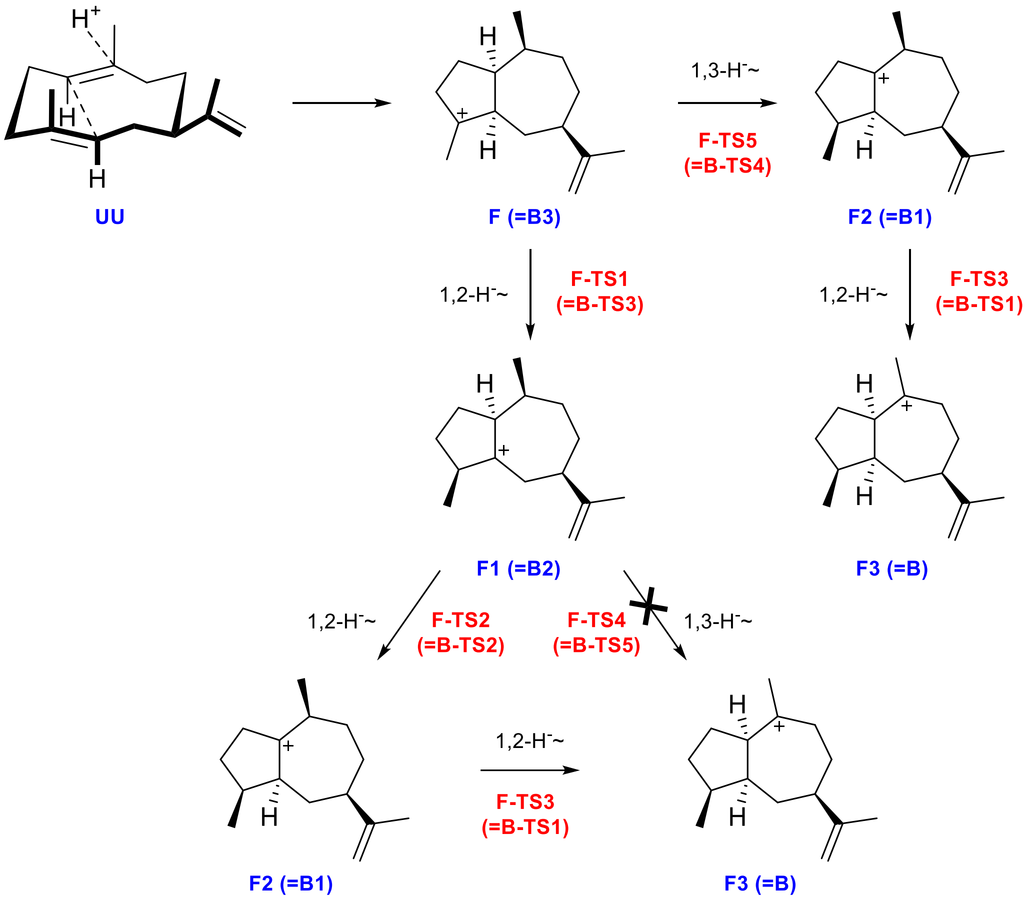

B)

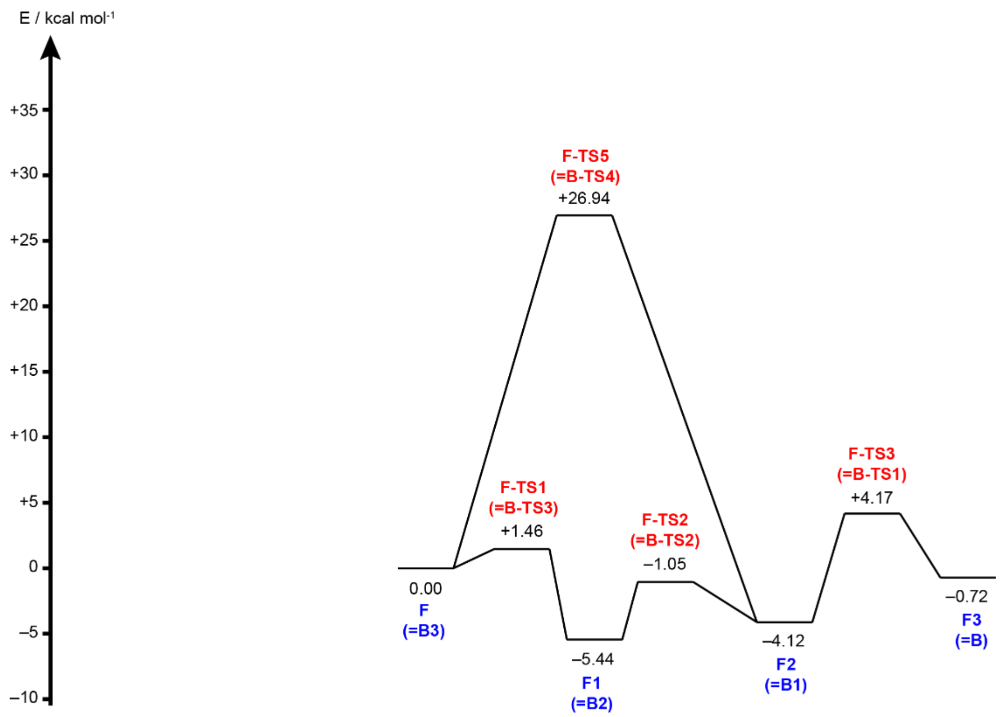

Figure S20. DFT calculations for **F** series from germacrene A. A) Hydride shifts starting from **F** (in **F3**: H1, H4 and H5 on bottom = BBB, intermediates identical to those in **B** series, pseudoenantiomeric to those in **C** and **G** series). B) Energy profile for the terpene biosynthetic steps (Gibbs energies, 298 K, mPW1PW91/6-311+G(d,p)//B97D3/6-31g(d,p)).

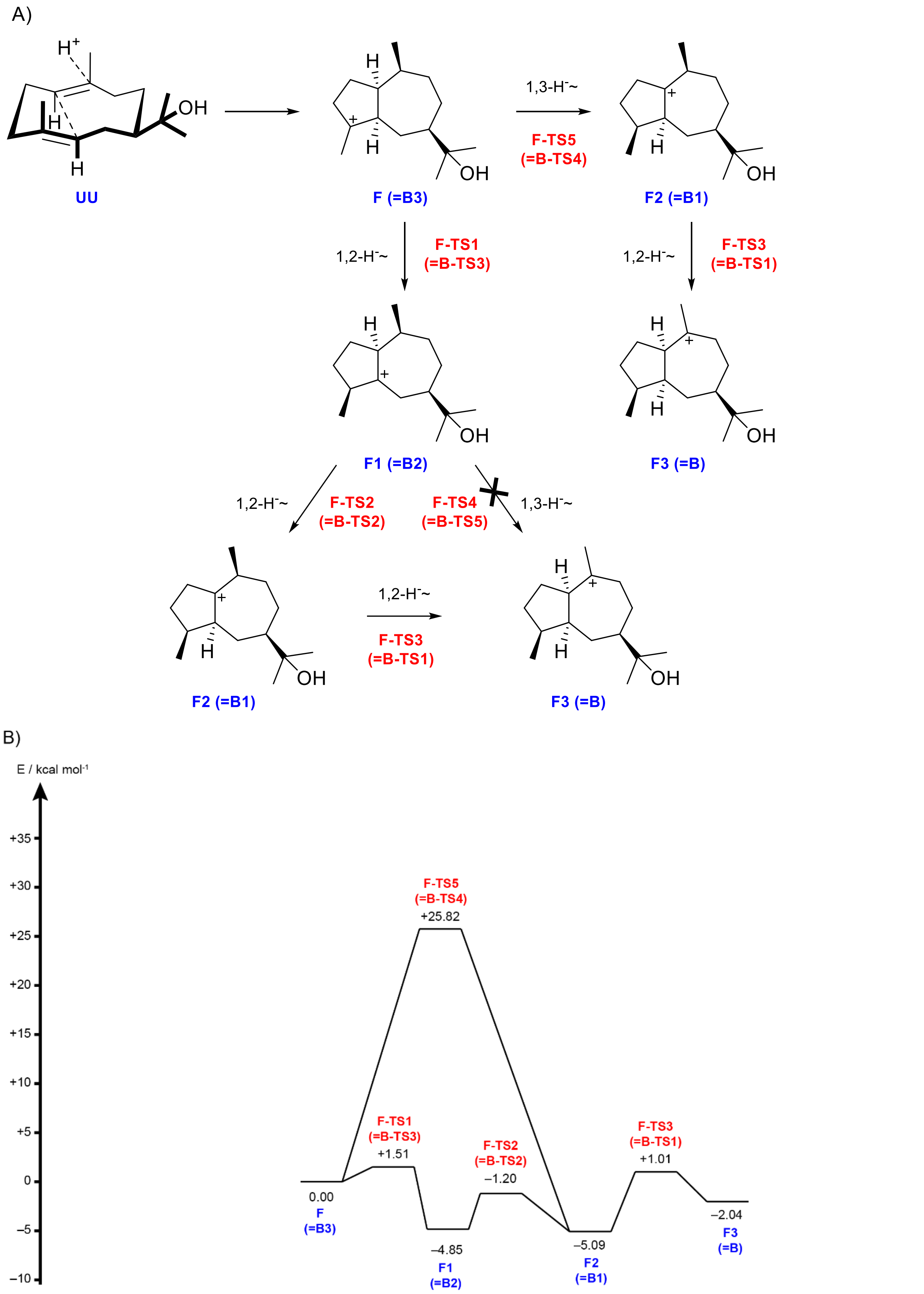

Figure S21. DFT calculations for **F** series from hedycaryol. A) Hydride shifts starting from **F** (in **F3**: H1, H4 and H5 on bottom = BBB, intermediates identical to those in **B** series, pseudoenantiomeric to those in **C** and **G** series). B) Energy profile for the terpene biosynthetic steps (Gibbs energies, 298 K, mPW1PW91/6-311+G(d,p)//B97D3/6-31g(d,p)).

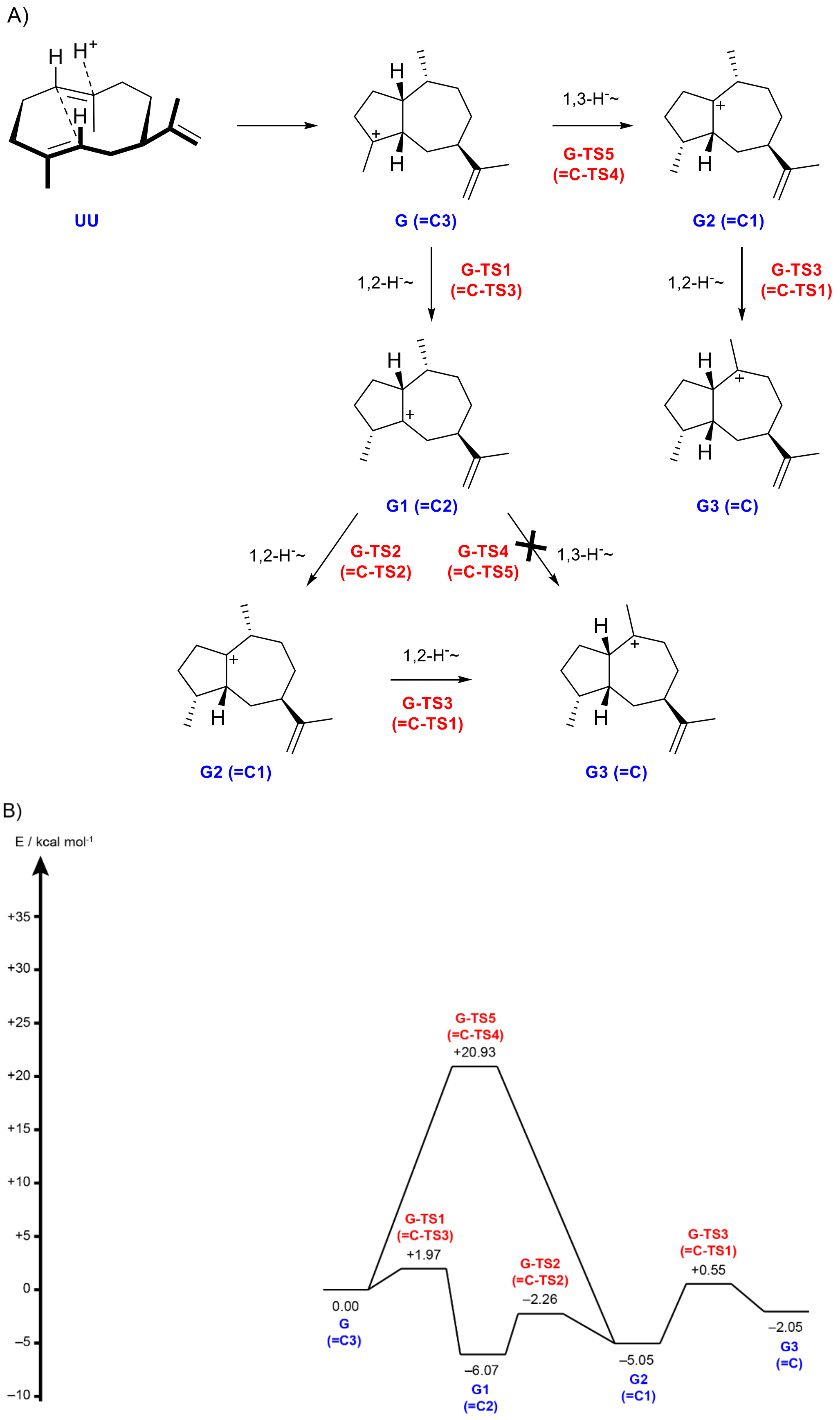

Figure S22. DFT calculations for **G** series from germacrene A. A) Hydride shifts starting from **G** (in **G3**: H1, H4 and H5 on top = TTT, intermediates identical to those in **C** series, pseudoenantiomeric to those in **B** and **F** series). B) Energy profile for the terpene biosynthetic steps (Gibbs energies, 298 K, mPW1PW91/6-311+G(d,p)//B97D3/6-31g(d,p)).

A)

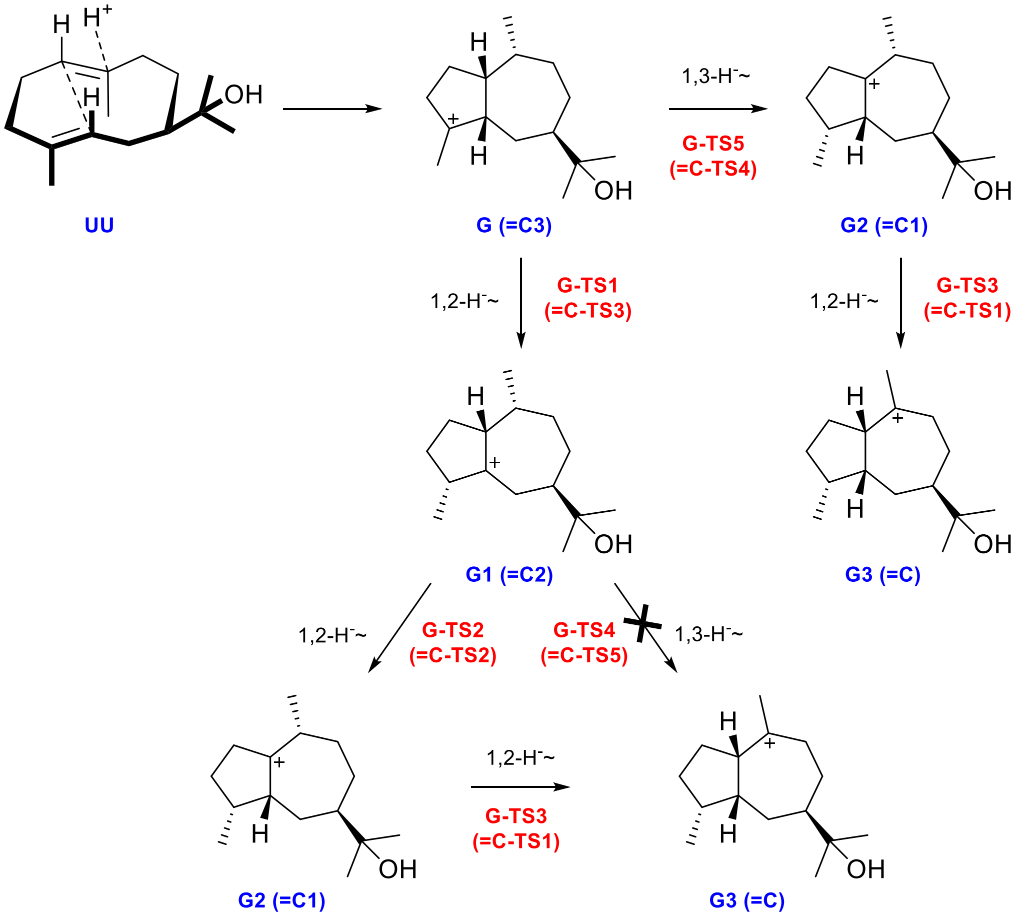

B)

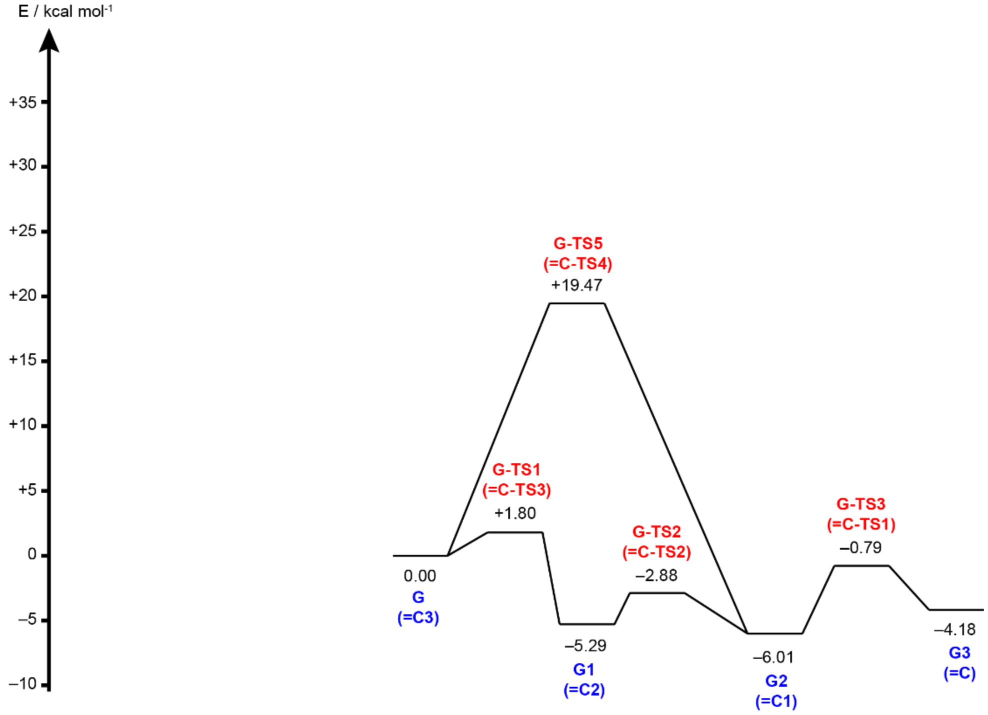

Figure S23. DFT calculations for **G** series from hedycaryol. A) Hydride shifts starting from **G** (in **G3**: H1, H4 and H5 on top = TTT, intermediates identical to those in **C** series, pseudoenantiomeric to those in **B** and **F** series). B) Energy profile for the terpene biosynthetic steps (Gibbs energies, 298 K, mPW1PW91/6-311+G(d,p)//B97D3/6-31g(d,p)).

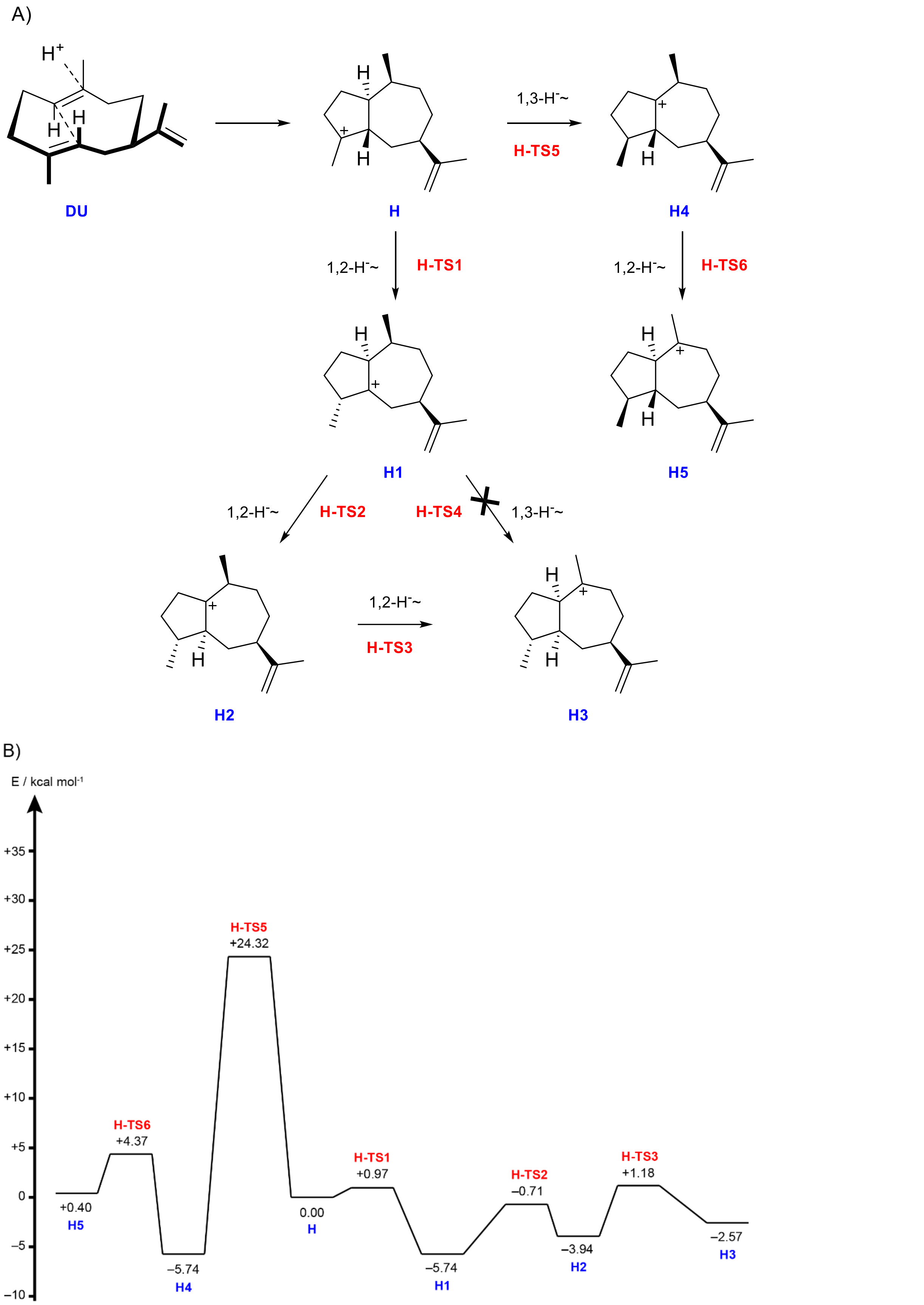

Figure S24. DFT calculations for **H** series from germacrene A. A) Hydride shifts starting from **H** (in **H3**: H1 on bottom, H4 on top, H5 on bottom = BTB, pseudoenantiomeric to **E** series). B) Energy profile for the terpene biosynthetic steps (Gibbs energies, 298 K, mPW1PW91/6-311+G(d,p)//B97D3/6-31g(d,p)).

A)

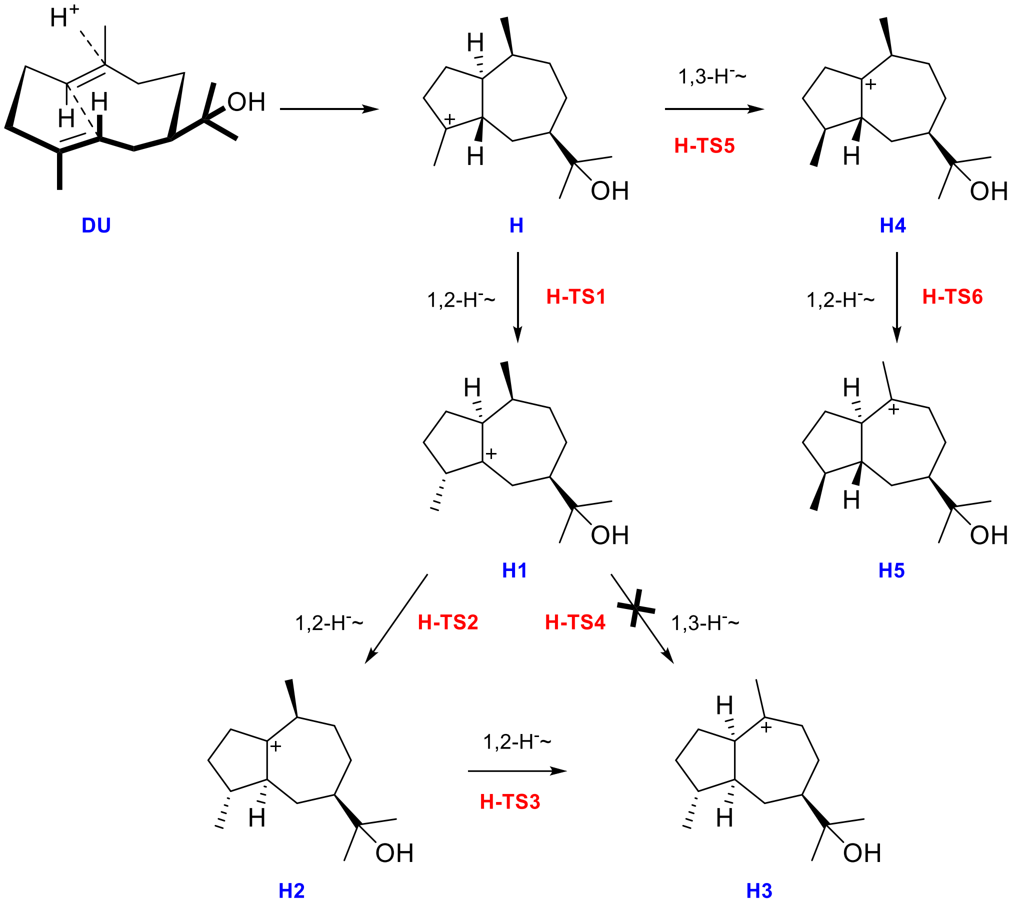

B)

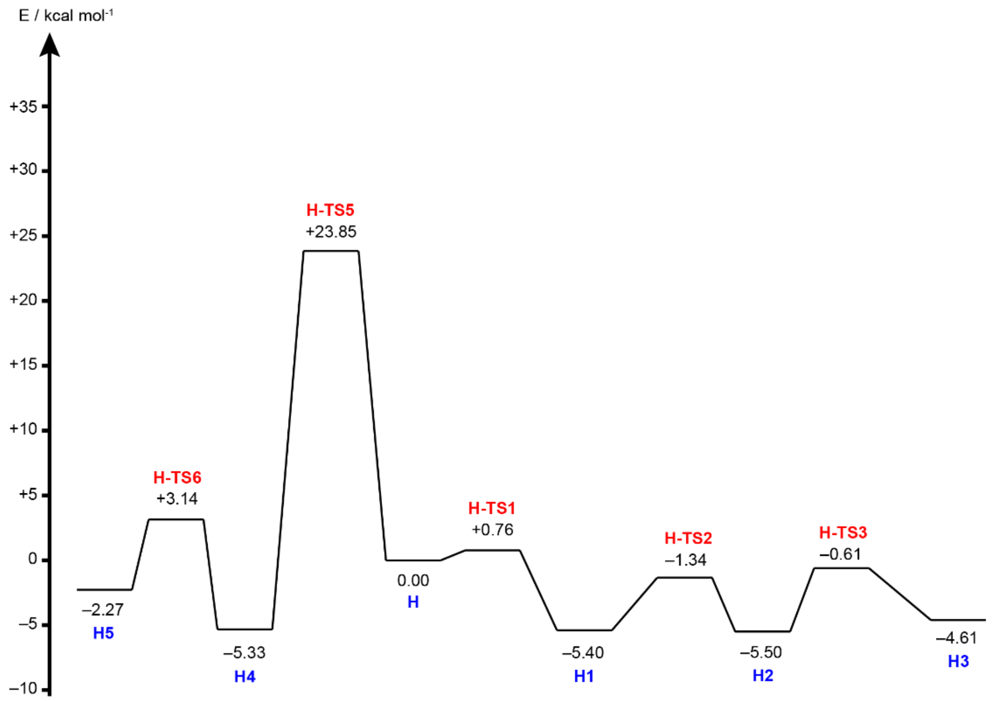

Figure S25. DFT calculations for **H** series from hedycaryol. A) Hydride shifts starting from **H** (in **H3**: H1 on bottom, H4 on top, H5 on bottom = BTB, pseudoenantiomeric to **E** series). B) Energy profile for the terpene biosynthetic steps (Gibbs energies, 298 K, mPW1PW91/6-311+G(d,p)//B97D3/6-31g(d,p)).

Experimental and computational part

Computational methods

All computed structures are geometry optimized without restrictions and are characterized as minima or as transition state structures by frequency analyses, also providing Gibbs-corrections, using the B97D3/6-31g(d,p) method with the density fitting approximation for s- and p-functions, including Grimme's empirical D3-dispersion correction[20] in Gaussian16.[21] For improved single point energies, the mPW1PW91 functional was applied with the 6-311+G(d,p) basis set without density fitting and the ultra-fine integration grid, as this method has shown to be very reliable for examining carbocation cyclisation and rearrangement reactions.[22-25] The Gibbs corrections include an entropic quasi-harmonic treatment with a frequency cut-off value of 100.0 wavenumbers, according to Grimme, using a mixture of RRHO and free-rotor vibrational entropies.[26,27]

Isotopic labeling experiments

Isotopic labeling experiments were conducted with the substrates and enzymes as listed in Table S1. Each reaction mixture contained substrates (1 mg each) in aqueous NH_4HCO_3 solution (1 mL; 25 mM), enzyme preparations (2 mL each) obtained as reported previously (for references cf. Table S1) and incubation buffer (3 – 7 mL to a total volume of 10 mL; 50 mM Tris, 10 mM $MgCl_2$, 20 vol-% glycerol, pH 8.2). After incubation with shaking at 28 °C overnight, the reaction mixtures were extracted with C_6D_6 (0.6 mL + 0.3 mL). The extracts were dried over $MgSO_4$ and directly analyzed by NMR.

Table S1. Isotopic labeling experiments with PtTPS5.

no.	substrates	enzymes	results shown in
1	(7-^{13}C)FPP[28] in ^2H_2O	PtTPS5	Figure 1 (main text)
2	(6-^{13}C)FPP[28] in ^2H_2O	PtTPS5	Figure 1 (main text)
3	(2-^{13}C)FPP[28] in ^2H_2O	PtTPS5	Figure 1 (main text)
4	(2-^{2}H)DMAPP[29] + (3-^{13}C)IPP[30]	IDI[31] + FPPS[32] + PtTPS5[7]	Figure 1 (main text)

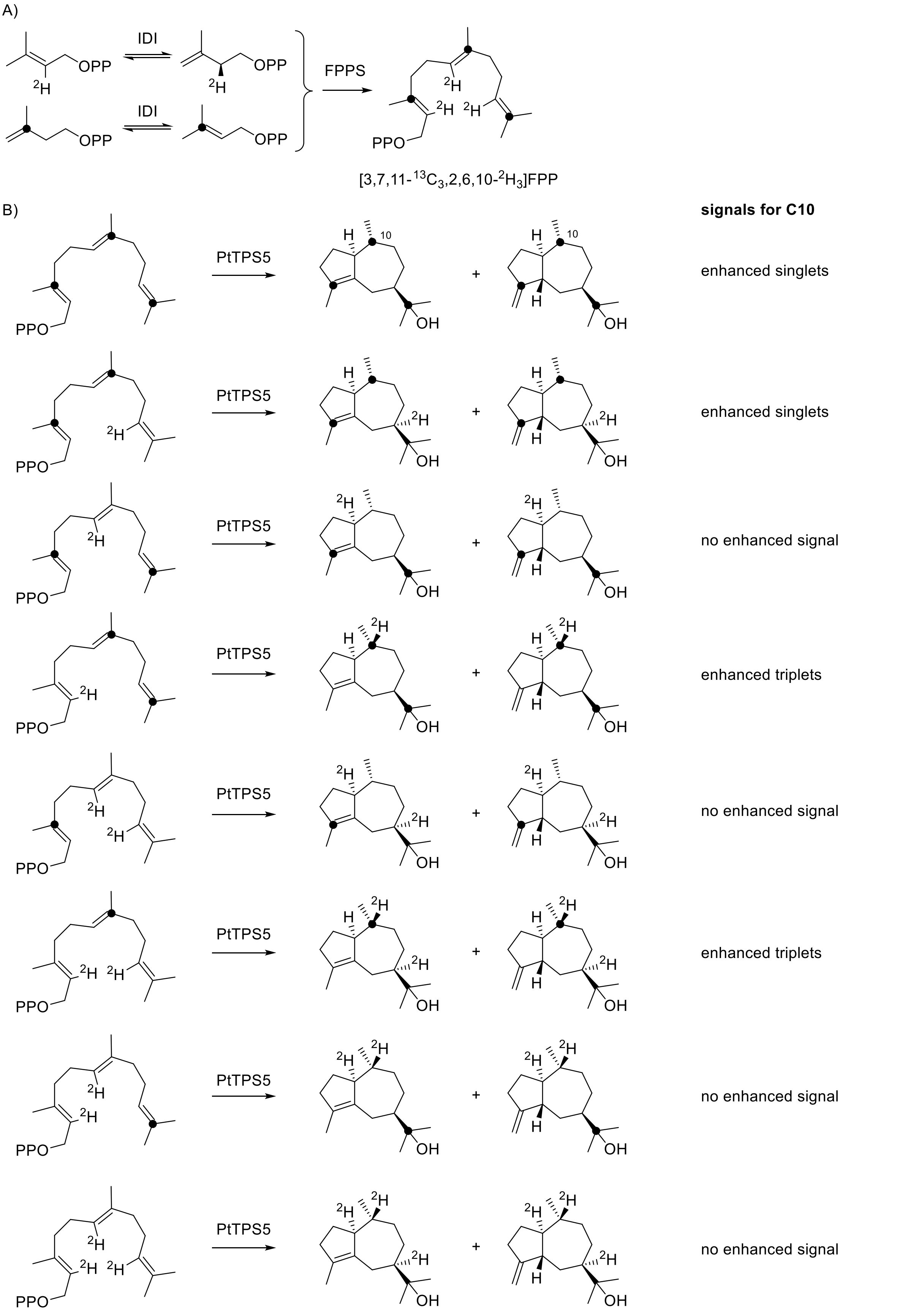

Scheme S15. A) Formation of [3,7,11-$^{13}C_3$,2,6,10-$^{2}H_3$]FPP from (2-^{2}H)DMAPP and (3-^{13}C)IPP (mixture of isotopomers). B) Conversion of each of the eight isotopomers in this mixture by PtTPS5 and expected signals for C10 in the ^{13}C-NMR.

Computational Results for Guaiadiene

Intermediate A (–586,3834435 Hartree)

C(1)	3.163	-0.907	0.430
H(2)	3.989	-1.604	0.246
H(3)	3.431	-0.312	1.317
C(4)	2.893	0.014	-0.777
H(5)	3.566	0.880	-0.818
H(6)	3.030	-0.531	-1.719
C(7)	1.403	0.422	-0.678
C(8)	1.040	1.711	-0.150
C(9)	1.990	2.517	0.660
H(10)	2.471	3.223	-0.045
H(11)	1.486	3.128	1.417
H(12)	2.797	1.929	1.107
C(13)	-0.312	2.279	-0.401
H(14)	-0.133	2.970	-1.255
H(15)	-0.594	2.940	0.432
C(16)	-1.463	1.318	-0.757
H(17)	-1.292	0.843	-1.735
H(18)	-2.361	1.940	-0.872
C(19)	-1.728	0.244	0.328
H(20)	-1.645	0.738	1.313
C(21)	-3.146	-0.312	0.210
C(22)	-4.017	-0.159	1.223
H(23)	-5.034	-0.549	1.161
H(24)	-3.744	0.357	2.145
C(25)	-3.526	-1.025	-1.069
H(26)	-2.914	-1.925	-1.235
H(27)	-3.389	-0.383	-1.953
H(28)	-4.575	-1.340	-1.048
C(29)	1.587	-2.780	-0.320
H(30)	2.404	-3.508	-0.233
H(31)	1.559	-2.440	-1.365
H(32)	0.649	-3.309	-0.115
C(33)	1.823	-1.628	0.669
C(34)	0.755	-0.492	0.557
H(35)	0.800	0.086	1.497
C(36)	-0.689	-0.903	0.286
H(37)	-0.747	-1.415	-0.685
H(38)	-0.965	-1.647	1.050
H(39)	0.808	0.157	-1.561
H(40)	1.765	-2.022	1.696

Intermediate A1 (–586,3842984 Hartree)

C(1)	-3.007	-1.363	-0.807
H(2)	-3.887	-1.920	-0.466
H(3)	-3.034	-1.330	-1.907
C(4)	-2.973	0.077	-0.252
H(5)	-3.541	0.849	-0.795
H(6)	-3.388	0.123	0.779
C(7)	-1.546	0.425	-0.080
C(8)	-1.052	1.730	0.375
C(9)	-2.117	2.829	0.512
H(10)	-2.976	2.508	1.115
H(11)	-1.678	3.706	1.001
H(12)	-2.485	3.147	-0.473
C(13)	0.225	2.212	-0.416
H(14)	0.284	3.292	-0.223
H(15)	0.037	2.100	-1.495
C(16)	1.558	1.565	-0.016
H(17)	1.682	1.652	1.075
H(18)	2.365	2.161	-0.464
C(19)	1.760	0.097	-0.449
H(20)	1.635	0.056	-1.547
C(21)	3.179	-0.379	-0.136
C(22)	4.017	-0.706	-1.134
H(23)	5.034	-1.045	-0.933
H(24)	3.720	-0.642	-2.182
C(25)	3.594	-0.468	1.317
H(26)	2.951	-1.157	1.887
H(27)	3.534	0.509	1.821
H(28)	4.625	-0.826	1.411
C(29)	-1.752	-2.547	1.107
H(30)	-2.462	-3.382	1.135
H(31)	-2.089	-1.798	1.841
H(32)	-0.779	-2.926	1.444
C(33)	-1.672	-1.972	-0.315
C(34)	-0.707	-0.737	-0.398
H(35)	-0.617	-0.533	-1.505
C(36)	0.716	-0.865	0.169
H(37)	0.677	-0.741	1.262
H(38)	1.041	-1.898	-0.017
H(39)	-1.313	-2.753	-0.999
H(40)	-0.666	1.467	1.391

Intermediate A2 (–586,3855325 Hartree)

C(1)	2.849	-1.469	0.858
H(2)	3.714	-2.083	0.582
H(3)	2.766	-1.480	1.955
C(4)	2.940	-0.020	0.345
H(5)	3.565	0.622	0.972
H(6)	3.346	0.018	-0.677
C(7)	1.453	0.447	0.335
C(8)	1.058	1.765	-0.394
C(9)	2.184	2.807	-0.253
H(10)	3.132	2.456	-0.676
H(11)	1.906	3.727	-0.783
H(12)	2.352	3.066	0.802
C(13)	-0.269	2.355	0.134
H(14)	-0.367	3.369	-0.279
H(15)	-0.184	2.484	1.227
C(16)	-1.563	1.598	-0.197
H(17)	-1.693	1.556	-1.291
H(18)	-2.409	2.185	0.190
C(19)	-1.686	0.175	0.383
H(20)	-1.407	0.195	1.449
C(21)	-3.088	-0.399	0.268
C(22)	-3.700	-0.911	1.355
H(23)	-4.711	-1.314	1.299
H(24)	-3.219	-0.922	2.335
C(25)	-3.743	-0.382	-1.093
H(26)	-3.100	-0.842	-1.863
H(27)	-3.942	0.646	-1.429
H(28)	-4.694	-0.926	-1.085
C(29)	1.752	-2.588	-1.210
H(30)	2.428	-3.445	-1.109
H(31)	2.218	-1.856	-1.882
H(32)	0.809	-2.929	-1.653
C(33)	1.534	-1.991	0.231
C(34)	0.686	-0.783	0.060
H(35)	1.182	0.587	1.417
C(36)	-0.711	-0.844	-0.345
H(37)	-0.730	-0.563	-1.418
H(38)	-1.091	-1.873	-0.283
H(39)	1.009	-2.764	0.815
H(40)	0.941	1.525	-1.467

Intermediate A3 (–586,3778713 Hartree)

C(1)	2.572	1.665	0.761
H(2)	2.598	2.341	1.631
H(3)	3.484	1.931	0.180
C(4)	2.521	0.148	1.035
H(5)	3.518	-0.282	1.175
H(6)	1.949	-0.047	1.954
C(7)	1.777	-0.437	-0.195
C(8)	1.174	-1.838	0.047
C(9)	2.282	-2.902	-0.056
H(10)	3.136	-2.672	0.596
H(11)	1.899	-3.889	0.234
H(12)	2.657	-2.977	-1.088
C(13)	-0.013	-2.166	-0.889
H(14)	-0.082	-3.260	-0.984
H(15)	0.185	-1.794	-1.909
C(16)	-1.375	-1.669	-0.371
H(17)	-1.478	-1.998	0.675
H(18)	-2.181	-2.170	-0.929
C(19)	-1.653	-0.159	-0.464
H(20)	-1.655	0.133	-1.528
C(21)	-3.002	0.230	0.133
C(22)	-3.812	1.073	-0.543
H(23)	-4.787	1.361	-0.151
H(24)	-3.536	1.475	-1.520
C(25)	-3.392	-0.333	1.481
H(26)	-2.592	-0.215	2.227
H(27)	-3.604	-1.410	1.416
H(28)	-4.290	0.160	1.870
C(29)	1.098	3.293	-0.641
H(30)	1.921	3.643	-1.293
H(31)	1.063	4.016	0.188
H(32)	0.171	3.319	-1.222
C(33)	1.459	1.942	-0.174
C(34)	0.804	0.708	-0.596
H(35)	2.497	-0.519	-1.031
C(36)	-0.575	0.743	0.232
H(37)	-0.386	0.427	1.266
H(38)	-0.968	1.766	0.263
H(39)	0.503	0.733	-1.654
H(40)	0.788	-1.858	1.083

Intermediate A4 (–586,3803208 Hartree)

C(1)	2.983	-0.918	0.558
H(2)	3.821	-1.626	0.536
H(3)	3.105	-0.287	1.451
C(4)	2.885	-0.054	-0.719
H(5)	3.591	0.784	-0.743
H(6)	3.079	-0.662	-1.617
C(7)	1.398	0.404	-0.733
C(8)	1.059	1.576	0.330
C(9)	2.233	2.566	0.471
H(10)	2.522	2.983	-0.505
H(11)	1.904	3.398	1.113
H(12)	3.115	2.117	0.943
C(13)	-0.227	2.328	-0.085
H(14)	0.026	3.035	-0.892
H(15)	-0.526	2.938	0.782
C(16)	-1.440	1.482	-0.541
H(17)	-1.343	1.205	-1.603
H(18)	-2.334	2.121	-0.477
C(19)	-1.678	0.206	0.300
H(20)	-1.409	0.394	1.352
C(21)	-3.119	-0.285	0.256
C(22)	-3.775	-0.519	1.409
H(23)	-4.819	-0.839	1.416
H(24)	-3.302	-0.378	2.385
C(25)	-3.766	-0.490	-1.096
H(26)	-3.213	-1.223	-1.710
H(27)	-3.800	0.446	-1.676
H(28)	-4.794	-0.862	-0.989
C(29)	1.643	-3.001	-0.193
H(30)	2.366	-3.654	0.315
H(31)	1.976	-2.854	-1.230
H(32)	0.664	-3.497	-0.199
C(33)	1.606	-1.642	0.599
C(34)	0.676	-0.741	-0.142
H(35)	0.894	1.122	1.323
C(36)	-0.763	-0.995	-0.226
H(37)	-1.026	-1.107	-1.294
H(38)	-1.040	-1.917	0.302
H(39)	1.029	0.733	-1.715
H(40)	1.226	-1.867	1.610

Intermediate A5 (–586,3768311 Hartree)

C(1)	2.994	1.431	0.342
H(2)	3.000	1.611	1.443
H(3)	3.823	2.042	-0.047
C(4)	2.995	-0.087	0.064
H(5)	3.297	-0.262	-0.980
H(6)	3.691	-0.630	0.711
C(7)	1.517	-0.487	0.276
C(8)	1.080	-1.818	-0.381
C(9)	2.170	-2.902	-0.286
H(10)	2.443	-3.093	0.762
H(11)	1.799	-3.846	-0.706
H(12)	3.082	-2.633	-0.835
C(13)	-0.242	-2.388	0.198
H(14)	0.008	-3.040	1.050
H(15)	-0.673	-3.051	-0.568
C(16)	-1.323	-1.402	0.678
H(17)	-1.054	-0.995	1.667
H(18)	-2.241	-1.985	0.840
C(19)	-1.643	-0.226	-0.279
H(20)	-1.463	-0.550	-1.318
C(21)	-3.101	0.220	-0.182
C(22)	-3.868	0.280	-1.288
H(23)	-4.916	0.578	-1.237
H(24)	-3.479	0.015	-2.273
C(25)	-3.644	0.586	1.182
H(26)	-3.109	1.445	1.619
H(27)	-3.539	-0.244	1.897
H(28)	-4.705	0.852	1.128
C(29)	1.279	3.312	-0.203
H(30)	2.136	3.989	-0.282
H(31)	0.721	3.573	0.718
H(32)	0.559	3.472	-1.018
C(33)	1.661	1.899	-0.075
C(34)	0.763	0.760	-0.298
H(35)	0.924	-1.597	-1.454
C(36)	-0.735	1.000	-0.001
H(37)	-0.828	1.293	1.057
H(38)	-1.092	1.848	-0.603
H(39)	1.322	-0.530	1.364
H(40)	0.860	0.664	-1.417

Transition State A-TS1 (–586,372586 Hartree)

C(1)	-3.045	-1.362	-0.668
H(2)	-3.856	-2.000	-0.299
H(3)	-3.207	-1.200	-1.744
C(4)	-3.013	0.003	0.058
H(5)	-3.699	0.746	-0.367
H(6)	-3.282	-0.116	1.118
C(7)	-1.557	0.444	-0.077
C(8)	-1.130	1.790	0.078
C(9)	-2.112	2.859	0.496
H(10)	-3.037	2.472	0.930
H(11)	-1.644	3.562	1.196
H(12)	-2.371	3.434	-0.408
C(13)	0.245	2.275	-0.339
H(14)	0.316	3.334	-0.059
H(15)	0.246	2.238	-1.443
C(16)	1.491	1.534	0.189
H(17)	1.422	1.448	1.286
H(18)	2.354	2.182	-0.016
C(19)	1.760	0.153	-0.443
H(20)	1.706	0.275	-1.539
C(21)	3.171	-0.331	-0.105
C(22)	4.079	-0.497	-1.081
H(23)	5.091	-0.839	-0.862
H(24)	3.845	-0.299	-2.128
C(25)	3.499	-0.608	1.346
H(26)	2.860	-1.400	1.767
H(27)	3.357	0.282	1.978
H(28)	4.540	-0.930	1.460
C(29)	-1.535	-2.622	0.965
H(30)	-2.279	-3.423	1.055
H(31)	-1.716	-1.912	1.787
H(32)	-0.547	-3.069	1.128
C(33)	-1.644	-1.958	-0.419
C(34)	-0.711	-0.717	-0.593
H(35)	-0.630	-0.511	-1.681
C(36)	0.710	-0.898	-0.030
H(37)	0.655	-0.954	1.069
H(38)	1.059	-1.884	-0.369
H(39)	-1.053	0.882	1.067
H(40)	-1.391	-2.703	-1.187

Transition State A-TS2 (–586,3774409 Hartree)

C(1)	2.908	-1.376	0.865
H(2)	3.787	-1.979	0.610
H(3)	2.857	-1.310	1.963
C(4)	2.968	0.047	0.269
H(5)	3.504	0.777	0.886
H(6)	3.454	0.056	-0.723
C(7)	1.519	0.422	0.017
C(8)	1.064	1.799	-0.424
C(9)	2.174	2.858	-0.288
H(10)	3.095	2.568	-0.807
H(11)	1.830	3.801	-0.729
H(12)	2.414	3.053	0.766
C(13)	-0.240	2.279	0.270
H(14)	-0.347	3.343	0.019
H(15)	-0.094	2.242	1.364
C(16)	-1.545	1.568	-0.120
H(17)	-1.652	1.586	-1.216
H(18)	-2.382	2.158	0.280
C(19)	-1.713	0.122	0.394
H(20)	-1.488	0.113	1.476
C(21)	-3.147	-0.376	0.220
C(22)	-3.865	-0.751	1.292
H(23)	-4.892	-1.101	1.192
H(24)	-3.456	-0.712	2.303
C(25)	-3.713	-0.422	-1.183
H(26)	-3.101	-1.046	-1.855
H(27)	-3.757	0.579	-1.637
H(28)	-4.728	-0.834	-1.184
C(29)	1.770	-2.714	-1.038
H(30)	2.428	-3.578	-0.889
H(31)	2.229	-2.063	-1.795
H(32)	0.814	-3.080	-1.432
C(33)	1.592	-1.978	0.313
C(34)	0.717	-0.754	0.067
H(35)	0.875	0.024	1.132
C(36)	-0.736	-0.880	-0.288
H(37)	-0.803	-0.761	-1.383
H(38)	-1.055	-1.905	-0.057
H(39)	1.111	-2.663	1.025
H(40)	0.832	1.669	-1.500

Transition State A-TS3 (–586,3713701 Hartree)

C(1)	-3.010	1.500	0.308
H(2)	-3.703	2.023	-0.368
H(3)	-3.272	1.839	1.324
C(4)	-3.014	-0.037	0.144
H(5)	-3.721	-0.532	0.817
H(6)	-3.307	-0.287	-0.887
C(7)	-1.545	-0.494	0.385
C(8)	-1.116	-1.798	-0.346
C(9)	-2.222	-2.862	-0.199
H(10)	-3.177	-2.549	-0.638
H(11)	-1.919	-3.787	-0.705
H(12)	-2.396	-3.103	0.860
C(13)	0.209	-2.373	0.210
H(14)	0.296	-3.409	-0.150
H(15)	0.118	-2.447	1.308
C(16)	1.517	-1.655	-0.149
H(17)	1.656	-1.676	-1.243
H(18)	2.350	-2.232	0.278
C(19)	1.648	-0.197	0.343
H(20)	1.361	-0.161	1.410
C(21)	3.081	0.318	0.233
C(22)	3.731	0.764	1.321
H(23)	4.755	1.133	1.260
H(24)	3.270	0.758	2.311
C(25)	3.725	0.301	-1.136
H(26)	3.115	0.827	-1.889
H(27)	3.855	-0.728	-1.505
H(28)	4.712	0.776	-1.117
C(29)	-1.227	3.298	-0.420
H(30)	-1.772	4.034	0.183
H(31)	-1.556	3.429	-1.465
H(32)	-0.152	3.502	-0.381
C(33)	-1.591	1.903	-0.029
C(34)	-0.731	0.764	0.017
H(35)	-1.396	-0.685	1.464
C(36)	0.699	0.755	-0.442
H(37)	0.665	0.452	-1.502
H(38)	1.100	1.778	-0.434
H(39)	-0.803	1.498	1.053
H(40)	-0.994	-1.572	-1.422

Transition State A-TS4 (–586,3349268 Hartree)

C(1)	-1.875	-1.834	1.123
H(2)	-1.172	-2.677	1.158
H(3)	-2.830	-2.164	1.547
C(4)	-1.358	-0.513	1.754
H(5)	-2.050	-0.099	2.497
H(6)	-0.361	-0.563	2.213
C(7)	-1.330	0.322	0.468
C(8)	-1.313	1.827	0.394
C(9)	-2.400	2.423	-0.523
H(10)	-3.408	2.209	-0.144
H(11)	-2.281	3.511	-0.577
H(12)	-2.315	2.028	-1.546
C(13)	0.116	2.248	-0.082
H(14)	0.250	3.282	0.271
H(15)	0.114	2.296	-1.182
C(16)	1.319	1.389	0.384
H(17)	1.188	1.075	1.433
H(18)	2.203	2.042	0.386
C(19)	1.681	0.170	-0.519
H(20)	1.661	0.535	-1.561
C(21)	3.098	-0.331	-0.237
C(22)	3.997	-0.421	-1.231
H(23)	5.010	-0.784	-1.051
H(24)	3.755	-0.132	-2.256
C(25)	3.443	-0.713	1.187
H(26)	2.757	-1.472	1.595
H(27)	3.391	0.155	1.862
H(28)	4.459	-1.121	1.251
C(29)	-2.931	-1.858	-1.359
H(30)	-3.918	-2.109	-0.957
H(31)	-2.453	-2.784	-1.721
H(32)	-3.034	-1.187	-2.220
C(33)	-2.012	-1.302	-0.309
C(34)	-0.743	-0.520	-0.641
H(35)	-0.855	-0.015	-1.611
C(36)	0.680	-1.019	-0.441
H(37)	0.785	-1.535	0.524
H(38)	0.938	-1.753	-1.217
H(39)	-2.587	-0.146	0.071
H(40)	-1.491	2.188	1.421

Transition State A-TS5 (–586,3688174 Hartree)

C(1)	-3.185	-0.838	-0.108
H(2)	-3.870	-1.656	0.143
H(3)	-3.704	-0.223	-0.853
C(4)	-2.799	-0.013	1.146
H(5)	-3.471	0.837	1.319
H(6)	-2.835	-0.639	2.048
C(7)	-1.344	0.428	0.930
C(8)	-1.040	1.442	-0.157
C(9)	-2.157	2.187	-0.876
H(10)	-2.530	2.945	-0.171
H(11)	-1.772	2.703	-1.762
H(12)	-3.000	1.560	-1.167
C(13)	0.246	2.251	0.021
H(14)	-0.022	3.049	0.735
H(15)	0.477	2.748	-0.933
C(16)	1.479	1.479	0.517
H(17)	1.364	1.184	1.570
H(18)	2.338	2.162	0.482
C(19)	1.770	0.247	-0.362
H(20)	1.696	0.567	-1.418
C(21)	3.169	-0.326	-0.147
C(22)	3.969	-0.556	-1.201
H(23)	4.969	-0.971	-1.075
H(24)	3.657	-0.332	-2.223
C(25)	3.603	-0.615	1.273
H(26)	2.899	-1.280	1.799
H(27)	3.669	0.307	1.871
H(28)	4.587	-1.095	1.294
C(29)	-1.556	-2.811	-0.130
H(30)	-2.377	-3.471	-0.437
H(31)	-1.511	-2.818	0.967
H(32)	-0.624	-3.233	-0.520
C(33)	-1.851	-1.392	-0.678
C(34)	-0.748	-0.449	-0.156
H(35)	-0.718	0.552	-1.059
C(36)	0.714	-0.873	-0.160
H(37)	0.882	-1.366	0.811
H(38)	0.866	-1.628	-0.940
H(39)	-0.738	0.563	1.829
H(40)	-1.851	-1.440	-1.777

Transition State A-TS6 (–586,3733579 Hartree)

C(1)	3.107	-1.332	-0.196
H(2)	3.319	-1.590	-1.250
H(3)	3.871	-1.826	0.416
C(4)	2.990	0.190	-0.002
H(5)	3.174	0.431	1.057
H(6)	3.712	0.755	-0.599
C(7)	1.517	0.540	-0.355
C(8)	0.972	1.811	0.350
C(9)	2.028	2.935	0.378
H(10)	2.357	3.188	-0.640
H(11)	1.592	3.841	0.818
H(12)	2.913	2.677	0.972
C(13)	-0.324	2.378	-0.277
H(14)	-0.049	2.995	-1.148
H(15)	-0.752	3.074	0.460
C(16)	-1.410	1.382	-0.718
H(17)	-1.179	0.983	-1.720
H(18)	-2.349	1.940	-0.832
C(19)	-1.642	0.198	0.254
H(20)	-1.408	0.523	1.281
C(21)	-3.090	-0.289	0.248
C(22)	-3.808	-0.301	1.384
H(23)	-4.846	-0.633	1.396
H(24)	-3.388	0.032	2.334
C(25)	-3.674	-0.739	-1.074
H(26)	-3.137	-1.607	-1.492
H(27)	-3.622	0.056	-1.833
H(28)	-4.725	-1.030	-0.964
C(29)	1.396	-3.293	0.212
H(30)	2.139	-3.795	0.843
H(31)	1.465	-3.743	-0.792
H(32)	0.389	-3.488	0.592
C(33)	1.711	-1.840	0.057
C(34)	0.774	-0.768	-0.046
H(35)	0.762	1.523	1.397
C(36)	-0.714	-0.989	-0.102
H(37)	-0.908	-1.285	-1.149
H(38)	-0.985	-1.867	0.503
H(39)	1.424	0.661	-1.454
H(40)	1.239	-1.110	1.127

Intermediate B = F3 (–586,3774821 Hartree)

C(1)	-3.235	-0.995	-0.745
H(2)	-4.132	-1.451	-0.308
H(3)	-3.325	-1.064	-1.840
C(4)	-3.059	0.476	-0.328
H(5)	-3.748	1.161	-0.838
H(6)	-3.233	0.587	0.750
C(7)	-1.601	0.803	-0.707
C(8)	-0.801	1.699	0.103
C(9)	-1.110	1.961	1.528
H(10)	-1.490	1.073	2.048
H(11)	-0.273	2.406	2.075
H(12)	-1.945	2.688	1.540
C(13)	0.412	2.304	-0.472
H(14)	0.543	3.323	-0.079
H(15)	0.382	2.326	-1.568
C(16)	1.679	1.479	-0.008
H(17)	1.756	1.515	1.088
H(18)	2.543	2.024	-0.412
C(19)	1.703	0.022	-0.502
H(20)	1.627	0.041	-1.604
C(21)	3.057	-0.589	-0.137
C(22)	3.980	-0.801	-1.093
H(23)	4.954	-1.229	-0.856
H(24)	3.791	-0.559	-2.140
C(25)	3.317	-0.926	1.315
H(26)	4.328	-1.324	1.452
H(27)	2.608	-1.680	1.692
H(28)	3.217	-0.046	1.969
C(29)	-1.960	-2.023	1.222
H(30)	-1.032	-2.505	1.552
H(31)	-2.782	-2.725	1.415
H(32)	-2.129	-1.147	1.864
C(33)	-1.931	-1.668	-0.273
C(34)	-0.806	-0.680	-0.702
H(35)	-0.598	-0.824	-1.772
C(36)	0.524	-0.824	0.048
H(37)	0.397	-0.641	1.126
H(38)	0.797	-1.886	-0.045
H(39)	-1.528	1.118	-1.757
H(40)	-1.747	-2.597	-0.839

Intermediate B1 = F2 (–586,3828966 Hartree)

C(1)	-3.209	-1.063	-0.806
H(2)	-4.137	-1.480	-0.400
H(3)	-3.242	-1.166	-1.901
C(4)	-3.022	0.425	-0.437
H(5)	-3.523	1.174	-1.069
H(6)	-3.407	0.626	0.586
C(7)	-1.560	0.647	-0.323
C(8)	-0.967	1.971	-0.087
C(9)	-0.958	2.116	1.488
H(10)	-0.426	1.296	1.982
H(11)	-0.434	3.053	1.712
H(12)	-1.976	2.175	1.886
C(13)	0.429	2.235	-0.711
H(14)	0.604	3.313	-0.574
H(15)	0.357	2.072	-1.799
C(16)	1.632	1.465	-0.146
H(17)	1.679	1.588	0.946
H(18)	2.540	1.944	-0.540
C(19)	1.694	-0.034	-0.512
H(20)	1.590	-0.113	-1.609
C(21)	3.050	-0.637	-0.140
C(22)	3.856	-1.130	-1.095
H(23)	4.827	-1.562	-0.851
H(24)	3.578	-1.114	-2.150
C(25)	3.440	-0.655	1.323
H(26)	4.421	-1.123	1.463
H(27)	2.715	-1.214	1.936
H(28)	3.493	0.361	1.744
C(29)	-2.070	-2.122	1.248
H(30)	-1.145	-2.568	1.633
H(31)	-2.872	-2.860	1.375
H(32)	-2.311	-1.256	1.883
C(33)	-1.939	-1.738	-0.234
C(34)	-0.852	-0.630	-0.456
H(35)	-0.746	-0.558	-1.581
C(36)	0.553	-0.863	0.122
H(37)	0.520	-0.703	1.210
H(38)	0.774	-1.929	-0.027
H(39)	-1.666	-2.630	-0.814
H(40)	-1.671	2.737	-0.452

Intermediate B2 = F1 (–586,3850088 Hartree)

C(1)	-3.043	-1.203	-0.903
H(2)	-3.996	-1.626	-0.565
H(3)	-2.916	-1.465	-1.964
C(4)	-2.948	0.323	-0.714
H(5)	-3.418	0.893	-1.523
H(6)	-3.420	0.640	0.227
C(7)	-1.419	0.586	-0.644
C(8)	-0.937	1.965	-0.111
C(9)	-1.125	2.090	1.412
H(10)	-0.497	1.384	1.975
H(11)	-0.851	3.100	1.743
H(12)	-2.168	1.917	1.707
C(13)	0.485	2.336	-0.584
H(14)	0.665	3.383	-0.300
H(15)	0.509	2.310	-1.687
C(16)	1.649	1.493	-0.039
H(17)	1.689	1.566	1.059
H(18)	2.586	1.937	-0.405
C(19)	1.653	0.006	-0.459
H(20)	1.434	-0.059	-1.538
C(21)	2.988	-0.676	-0.205
C(22)	3.600	-1.358	-1.194
H(23)	4.561	-1.847	-1.035
H(24)	3.170	-1.430	-2.195
C(25)	3.575	-0.570	1.183
H(26)	4.474	-1.187	1.285
H(27)	2.857	-0.894	1.956
H(28)	3.846	0.468	1.426
C(29)	-2.191	-1.934	1.436
H(30)	-1.332	-2.316	1.999
H(31)	-3.009	-2.663	1.493
H(32)	-2.530	-0.997	1.894
C(33)	-1.841	-1.746	-0.090
C(34)	-0.830	-0.660	-0.126
H(35)	-1.043	0.502	-1.701
C(36)	0.557	-0.844	0.292
H(37)	0.589	-0.526	1.355
H(38)	0.816	-1.914	0.297
H(39)	-1.431	-2.705	-0.443
H(40)	-1.620	2.688	-0.586

Intermediate B3 = F (–586,3763345 Hartree)

C(1)	-3.200	-0.788	-0.219
H(2)	-3.571	-0.488	0.784
H(3)	-3.961	-1.504	-0.574
C(4)	-2.897	0.424	-1.125
H(5)	-3.093	0.150	-2.171
H(6)	-3.531	1.286	-0.887
C(7)	-1.385	0.728	-0.920
C(8)	-1.087	1.827	0.132
C(9)	-1.482	1.451	1.570
H(10)	-0.998	0.526	1.922
H(11)	-1.183	2.243	2.268
H(12)	-2.569	1.324	1.676
C(13)	0.354	2.378	0.020
H(14)	0.409	3.288	0.637
H(15)	0.516	2.704	-1.021
C(16)	1.509	1.455	0.434
H(17)	1.424	1.197	1.502
H(18)	2.442	2.029	0.335
C(19)	1.658	0.157	-0.396
H(20)	1.506	0.403	-1.461
C(21)	3.058	-0.440	-0.259
C(22)	3.824	-0.636	-1.348
H(23)	4.829	-1.050	-1.267
H(24)	3.479	-0.375	-2.350
C(25)	3.541	-0.791	1.131
H(26)	4.544	-1.232	1.103
H(27)	2.874	-1.514	1.628
H(28)	3.581	0.096	1.780
C(29)	-1.742	-2.683	0.806
H(30)	-0.942	-3.318	0.398
H(31)	-2.674	-3.241	0.936
H(32)	-1.378	-2.384	1.808
C(33)	-1.904	-1.444	0.030
C(34)	-0.806	-0.693	-0.575
H(35)	-0.944	1.062	-1.869
C(36)	0.608	-0.912	0.012
H(37)	0.537	-0.945	1.110
H(38)	0.967	-1.901	-0.306
H(39)	-0.823	-1.218	-1.573
H(40)	-1.739	2.666	-0.167

Transition State B-TS1 = F-TS3 (–586,369684 Hartree)

C(1)	-3.172	-1.169	-0.340
H(2)	-3.908	-1.823	0.142
H(3)	-3.599	-0.835	-1.299
C(4)	-2.833	0.065	0.520
H(5)	-3.618	0.828	0.558
H(6)	-2.612	-0.229	1.561
C(7)	-1.533	0.538	-0.100
C(8)	-1.026	1.866	-0.066
C(9)	-1.695	2.955	0.727
H(10)	-1.010	3.190	1.558
H(11)	-1.793	3.874	0.135
H(12)	-2.662	2.677	1.154
C(13)	0.293	2.211	-0.731
H(14)	0.404	3.303	-0.721
H(15)	0.276	1.903	-1.788
C(16)	1.501	1.535	-0.037
H(17)	1.385	1.598	1.057
H(18)	2.395	2.121	-0.291
C(19)	1.733	0.073	-0.477
H(20)	1.775	0.072	-1.581
C(21)	3.082	-0.435	0.031
C(22)	4.055	-0.750	-0.841
H(23)	5.027	-1.112	-0.503
H(24)	3.916	-0.658	-1.920
C(25)	3.280	-0.567	1.526
H(26)	4.295	-0.909	1.759
H(27)	2.578	-1.288	1.971
H(28)	3.125	0.390	2.048
C(29)	-1.494	-2.832	0.603
H(30)	-0.531	-3.340	0.475
H(31)	-2.269	-3.608	0.641
H(32)	-1.479	-2.332	1.582
C(33)	-1.804	-1.861	-0.552
C(34)	-0.788	-0.660	-0.702
H(35)	-0.632	-0.471	-1.779
C(36)	0.600	-0.888	-0.054
H(37)	0.493	-0.884	1.041
H(38)	0.916	-1.903	-0.330
H(39)	-1.861	1.392	-1.021
H(40)	-1.805	-2.437	-1.488

Transition State B-TS2 = F-TS2 (–586,378001 Hartree)

C(1)	-3.121	-1.107	-0.876
H(2)	-4.066	-1.543	-0.533
H(3)	-3.065	-1.248	-1.966
C(4)	-3.001	0.397	-0.545
H(5)	-3.428	1.071	-1.299
H(6)	-3.489	0.646	0.414
C(7)	-1.521	0.629	-0.322
C(8)	-0.947	2.007	-0.107
C(9)	-1.011	2.296	1.418
H(10)	-0.404	1.589	1.996
H(11)	-0.626	3.307	1.604
H(12)	-2.041	2.253	1.795
C(13)	0.457	2.250	-0.712
H(14)	0.661	3.323	-0.582
H(15)	0.405	2.080	-1.800
C(16)	1.636	1.458	-0.125
H(17)	1.678	1.591	0.967
H(18)	2.561	1.901	-0.518
C(19)	1.663	-0.047	-0.472
H(20)	1.483	-0.150	-1.558
C(21)	3.026	-0.669	-0.173
C(22)	3.738	-1.243	-1.158
H(23)	4.716	-1.685	-0.968
H(24)	3.374	-1.281	-2.187
C(25)	3.532	-0.606	1.252
H(26)	4.498	-1.114	1.350
H(27)	2.835	-1.084	1.959
H(28)	3.663	0.432	1.591
C(29)	-2.157	-2.208	1.261
H(30)	-1.256	-2.620	1.732
H(31)	-2.924	-2.992	1.256
H(32)	-2.522	-1.378	1.881
C(33)	-1.888	-1.755	-0.195
C(34)	-0.865	-0.623	-0.151
H(35)	-0.915	-0.039	-1.333
C(36)	0.561	-0.869	0.255
H(37)	0.612	-0.669	1.339
H(38)	0.770	-1.939	0.123
H(39)	-1.497	-2.610	-0.764
H(40)	-1.635	2.709	-0.603

Transition State B-TS3 = F-TS1 (–586,3740048 Hartree)

C(1)	-3.239	-0.777	-0.266
H(2)	-3.656	-0.479	0.712
H(3)	-3.971	-1.458	-0.720
C(4)	-2.857	0.442	-1.139
H(5)	-3.020	0.198	-2.198
H(6)	-3.467	1.322	-0.909
C(7)	-1.341	0.708	-0.885
C(8)	-1.034	1.892	0.091
C(9)	-1.454	1.611	1.544
H(10)	-0.906	0.768	1.992
H(11)	-1.250	2.489	2.171
H(12)	-2.529	1.399	1.627
C(13)	0.419	2.397	-0.039
H(14)	0.499	3.323	0.549
H(15)	0.593	2.686	-1.089
C(16)	1.544	1.449	0.405
H(17)	1.448	1.218	1.479
H(18)	2.495	1.990	0.297
C(19)	1.660	0.128	-0.398
H(20)	1.498	0.351	-1.466
C(21)	3.053	-0.491	-0.266
C(22)	3.799	-0.721	-1.359
H(23)	4.796	-1.157	-1.284
H(24)	3.448	-0.471	-2.362
C(25)	3.548	-0.820	1.125
H(26)	4.545	-1.274	1.094
H(27)	2.880	-1.525	1.648
H(28)	3.608	0.080	1.756
C(29)	-1.826	-2.748	0.783
H(30)	-0.826	-3.192	0.740
H(31)	-2.574	-3.470	0.432
H(32)	-2.053	-2.534	1.840
C(33)	-1.927	-1.451	0.047
C(34)	-0.834	-0.614	-0.320
H(35)	-0.822	0.935	-1.828
C(36)	0.604	-0.916	0.041
H(37)	0.636	-1.033	1.137
H(38)	0.881	-1.901	-0.366
H(39)	-1.268	-1.502	-1.159
H(40)	-1.674	2.709	-0.282

Transition State B-TS4 = F-TS5 (–586,3334032 Hartree)

C(1)	-2.015	-1.414	1.355
H(2)	-1.330	-2.183	1.738
H(3)	-3.005	-1.589	1.792
C(4)	-1.531	0.050	1.529
H(5)	-2.272	0.678	2.035
H(6)	-0.572	0.176	2.050
C(7)	-1.394	0.390	0.037
C(8)	-1.350	1.770	-0.571
C(9)	-1.910	2.853	0.364
H(10)	-1.303	2.950	1.275
H(11)	-1.898	3.823	-0.148
H(12)	-2.947	2.644	0.657
C(13)	0.145	2.021	-1.004
H(14)	0.271	3.113	-1.003
H(15)	0.268	1.696	-2.047
C(16)	1.271	1.382	-0.151
H(17)	1.032	1.440	0.923
H(18)	2.166	2.007	-0.281
C(19)	1.690	-0.065	-0.553
H(20)	1.778	-0.071	-1.653
C(21)	3.061	-0.428	0.018
C(22)	4.049	-0.839	-0.795
H(23)	5.031	-1.108	-0.406
H(24)	3.912	-0.914	-1.875
C(25)	3.261	-0.303	1.514
H(26)	4.256	-0.653	1.810
H(27)	2.522	-0.889	2.086
H(28)	3.162	0.741	1.849
C(29)	-2.873	-2.319	-1.035
H(30)	-2.903	-1.991	-2.081
H(31)	-3.890	-2.436	-0.646
H(32)	-2.376	-3.305	-1.017
C(33)	-2.033	-1.418	-0.176
C(34)	-0.733	-0.780	-0.658
H(35)	-0.757	-0.645	-1.749
C(36)	0.658	-1.167	-0.177
H(37)	0.667	-1.332	0.908
H(38)	0.966	-2.115	-0.638
H(39)	-2.621	-0.205	-0.265
H(40)	-1.946	1.760	-1.499

Intermediate C = G3 (–586,3733589 Hartree)

C(1)	1.588	-1.720	0.287
C(2)	0.445	-0.768	-0.137
H(3)	-0.091	-1.225	-0.978
C(4)	-0.567	-0.379	0.946
H(5)	-0.897	-1.310	1.432
H(6)	-0.066	0.191	1.744
C(7)	-1.829	0.403	0.469
H(8)	-2.170	0.997	1.333
C(9)	-2.994	-0.498	0.062
C(10)	-4.210	-0.304	0.603
H(11)	-4.393	0.476	1.344
H(12)	-5.062	-0.925	0.325
C(13)	-2.752	-1.589	-0.958
H(14)	-2.044	-2.346	-0.582
H(15)	-3.683	-2.110	-1.208
H(16)	-2.333	-1.198	-1.899
C(17)	-1.516	1.412	-0.663
H(18)	-1.340	0.888	-1.614
H(19)	-2.393	2.051	-0.830
C(20)	-0.320	2.325	-0.320
H(21)	-0.532	2.969	0.548
H(22)	-0.155	3.026	-1.167
C(23)	1.002	1.667	-0.160
C(24)	1.283	0.469	-0.922
H(25)	0.749	0.539	-1.881
C(26)	2.718	-0.070	-1.101
H(27)	3.098	0.178	-2.100
H(28)	3.413	0.377	-0.379
C(29)	2.612	-1.589	-0.857
H(30)	3.583	-2.039	-0.613
H(31)	2.231	-2.090	-1.761
C(32)	1.924	2.262	0.833
H(33)	2.956	1.914	0.775
C(34)	2.196	-1.438	1.669
H(35)	1.455	-1.529	2.472
H(36)	2.996	-2.160	1.875
H(37)	2.643	-0.436	1.736
H(38)	1.882	3.361	0.771
H(39)	1.524	2.025	1.837
H(40)	1.151	-2.733	0.303

Intermediate C1 = G2 (–586,3781336 Hartree)

C(1)	1.276	-2.007	0.068
C(2)	0.430	-0.688	-0.007
H(3)	-0.047	-0.733	-1.028
C(4)	-0.681	-0.466	1.034
H(5)	-1.059	-1.457	1.319
H(6)	-0.218	-0.046	1.938
C(7)	-1.878	0.420	0.596
H(8)	-2.320	0.805	1.528
C(9)	-2.969	-0.366	-0.129
C(10)	-4.244	-0.303	0.288
H(11)	-4.534	0.290	1.157
H(12)	-5.041	-0.841	-0.227
C(13)	-2.577	-1.204	-1.329
H(14)	-1.900	-2.029	-1.046
H(15)	-3.454	-1.663	-1.799
H(16)	-2.068	-0.612	-2.108
C(17)	-1.441	1.656	-0.231
H(18)	-1.289	1.379	-1.288
H(19)	-2.282	2.362	-0.238
C(20)	-0.177	2.357	0.299
H(21)	-0.126	2.298	1.397
H(22)	-0.219	3.429	0.061
C(23)	1.147	1.842	-0.307
C(24)	1.381	0.382	-0.329
C(25)	2.665	-0.211	-0.774
H(26)	3.154	0.330	-1.596
H(27)	3.330	-0.055	0.105
C(28)	2.397	-1.721	-0.958
H(29)	3.297	-2.324	-0.797
H(30)	2.042	-1.907	-1.983
C(31)	2.383	2.626	0.230
H(32)	3.301	2.404	-0.325
C(33)	1.805	-2.287	1.484
H(34)	0.987	-2.448	2.196
H(35)	2.425	-3.193	1.480
H(36)	2.423	-1.463	1.871
H(37)	2.180	3.698	0.137
H(38)	2.543	2.398	1.292
H(39)	0.648	-2.850	-0.252
H(40)	1.119	2.054	-1.405

Intermediate C2 = G1 (–586,3797634 Hartree)

C(1)	1.207	-1.866	0.333
C(2)	0.489	-0.569	0.310
C(3)	-0.603	-0.207	1.206
H(4)	-0.936	-1.060	1.809
H(5)	-0.164	0.540	1.895
C(6)	-1.864	0.522	0.560
H(7)	-2.382	0.942	1.435
C(8)	-2.774	-0.508	-0.079
C(9)	-4.001	-0.739	0.432
H(10)	-4.366	-0.200	1.307
H(11)	-4.683	-1.457	-0.024
C(12)	-2.259	-1.271	-1.274
H(13)	-1.331	-1.821	-1.031
H(14)	-2.986	-2.010	-1.626
H(15)	-2.017	-0.610	-2.121
C(16)	-1.500	1.709	-0.374
H(17)	-1.413	1.378	-1.419
H(18)	-2.366	2.386	-0.366
C(19)	-0.235	2.475	0.044
H(20)	-0.218	2.581	1.141
H(21)	-0.287	3.503	-0.340
C(22)	1.114	1.864	-0.443
C(23)	1.058	0.329	-0.711
C(24)	2.329	-0.392	-1.239
H(25)	2.519	-0.161	-2.293
H(26)	3.211	-0.071	-0.668
C(27)	2.023	-1.882	-0.984
H(28)	2.922	-2.504	-0.907
H(29)	1.399	-2.291	-1.794
C(30)	2.254	2.243	0.518
H(31)	3.243	1.984	0.122
C(32)	2.134	-1.802	1.606
H(33)	1.544	-1.772	2.529
H(34)	2.748	-2.711	1.604
H(35)	2.802	-0.933	1.581
H(36)	2.245	3.327	0.691
H(37)	2.136	1.754	1.497
H(38)	0.526	-2.718	0.478
H(39)	1.342	2.300	-1.429
H(40)	0.279	0.180	-1.502

Intermediate C3 = G (–586,3700929 Hartree)

C(1)	-1.722	-1.418	-0.299
C(2)	-0.494	-0.712	0.047
C(3)	0.580	-0.524	-1.037
H(4)	0.903	-1.509	-1.407
H(5)	0.094	-0.022	-1.888
C(6)	1.845	0.288	-0.652
H(7)	2.302	0.525	-1.627
C(8)	2.880	-0.516	0.132
C(9)	4.153	-0.591	-0.297
H(10)	4.476	-0.101	-1.218
H(11)	4.914	-1.137	0.262
C(12)	2.451	-1.199	1.411
H(13)	1.769	-2.048	1.211
H(14)	3.309	-1.616	1.952
H(15)	1.928	-0.515	2.096
C(16)	1.555	1.659	0.029
H(17)	1.564	1.556	1.124
H(18)	2.405	2.317	-0.198
C(19)	0.251	2.329	-0.439
H(20)	0.170	2.218	-1.533
H(21)	0.320	3.412	-0.263
C(22)	-1.069	1.819	0.230
C(23)	-0.919	0.472	0.978
C(24)	-2.218	-0.066	1.641
H(25)	-1.971	-0.609	2.564
H(26)	-2.919	0.733	1.908
C(27)	-2.819	-1.053	0.617
H(28)	-3.584	-0.577	-0.033
H(29)	-3.335	-1.942	1.019
C(30)	-2.222	1.841	-0.786
H(31)	-3.204	1.682	-0.318
C(32)	-1.857	-2.359	-1.421
H(33)	-0.999	-3.048	-1.463
H(34)	-2.802	-2.912	-1.420
H(35)	-1.781	-1.773	-2.357
H(36)	-2.260	2.812	-1.296
H(37)	-2.090	1.081	-1.575
H(38)	-1.325	2.536	1.030
H(39)	-0.149	0.601	1.747
H(40)	-0.115	-1.492	0.772

Transition State C-TS1 = G-TS3 (–586,369213 Hartree)

C(1)	1.306	-2.012	-0.001
C(2)	0.397	-0.745	-0.156
H(3)	-0.087	-0.844	-1.142
C(4)	-0.676	-0.557	0.932
H(5)	-1.079	-1.547	1.187
H(6)	-0.182	-0.194	1.847
C(7)	-1.857	0.383	0.575
H(8)	-2.247	0.768	1.531
C(9)	-3.017	-0.328	-0.123
C(10)	-4.267	-0.203	0.354
H(11)	-4.484	0.382	1.250
H(12)	-5.114	-0.685	-0.134
C(13)	-2.727	-1.160	-1.355
H(14)	-2.084	-2.024	-1.120
H(15)	-3.652	-1.555	-1.791
H(16)	-2.217	-0.582	-2.142
C(17)	-1.404	1.616	-0.238
H(18)	-1.210	1.342	-1.289
H(19)	-2.237	2.330	-0.274
C(20)	-0.171	2.341	0.337
H(21)	-0.216	2.354	1.441
H(22)	-0.171	3.396	0.031
C(23)	1.192	1.771	-0.012
C(24)	1.417	0.393	-0.282
H(25)	1.094	1.222	-1.240
C(26)	2.797	-0.191	-0.582
H(27)	3.334	0.346	-1.373
H(28)	3.401	-0.092	0.335
C(29)	2.514	-1.672	-0.900
H(30)	3.386	-2.307	-0.706
H(31)	2.247	-1.781	-1.962
C(32)	2.340	2.750	0.034
H(33)	3.302	2.330	-0.268
C(34)	1.723	-2.307	1.450
H(35)	0.861	-2.555	2.081
H(36)	2.407	-3.164	1.475
H(37)	2.242	-1.458	1.922
H(38)	2.122	3.645	-0.561
H(39)	2.426	3.082	1.081
H(40)	0.756	-2.881	-0.390

Transition State C-TS2 = G-TS2 (–586,3736981 Hartree)

C(1)	1.161	-1.955	0.193
C(2)	0.505	-0.582	0.298
H(3)	0.210	-0.227	-0.931
C(4)	-0.663	-0.303	1.204
H(5)	-1.035	-1.258	1.597
H(6)	-0.265	0.262	2.060
C(7)	-1.868	0.492	0.592
H(8)	-2.391	0.915	1.463
C(9)	-2.849	-0.421	-0.136
C(10)	-4.143	-0.454	0.225
H(11)	-4.522	0.146	1.054
H(12)	-4.866	-1.078	-0.301
C(13)	-2.326	-1.271	-1.274
H(14)	-1.606	-2.028	-0.919
H(15)	-3.136	-1.816	-1.772
H(16)	-1.817	-0.673	-2.049
C(17)	-1.438	1.688	-0.301
H(18)	-1.285	1.361	-1.344
H(19)	-2.293	2.374	-0.350
C(20)	-0.183	2.432	0.188
H(21)	-0.140	2.436	1.289
H(22)	-0.236	3.489	-0.109
C(23)	1.143	1.885	-0.389
C(24)	1.292	0.382	-0.393
C(25)	2.469	-0.311	-1.056
H(26)	2.699	0.095	-2.049
H(27)	3.343	-0.109	-0.416
C(28)	2.095	-1.810	-1.035
H(29)	2.971	-2.465	-0.978
H(30)	1.545	-2.066	-1.953
C(31)	2.367	2.483	0.357
H(32)	3.314	2.263	-0.149
C(33)	1.917	-2.228	1.515
H(34)	1.235	-2.263	2.373
H(35)	2.425	-3.197	1.446
H(36)	2.677	-1.459	1.712
H(37)	2.251	3.573	0.398
H(38)	2.421	2.110	1.389
H(39)	0.404	-2.740	0.053
H(40)	1.218	2.199	-1.445

Transition State C-TS3 = G-TS1 (–586,3669516 Hartree)

C(1)	-1.654	-1.481	-0.306
C(2)	-0.565	-0.575	-0.158
C(3)	0.597	-0.467	-1.106
H(4)	0.891	-1.462	-1.470
H(5)	0.166	0.046	-1.984
C(6)	1.866	0.310	-0.654
H(7)	2.384	0.514	-1.605
C(8)	2.813	-0.547	0.182
C(9)	4.077	-0.760	-0.225
H(10)	4.456	-0.337	-1.157
H(11)	4.775	-1.354	0.365
C(12)	2.303	-1.138	1.478
H(13)	1.465	-1.841	1.313
H(14)	3.087	-1.704	1.995
H(15)	1.940	-0.368	2.176
C(16)	1.587	1.703	-0.004
H(17)	1.611	1.631	1.093
H(18)	2.437	2.350	-0.261
C(19)	0.279	2.373	-0.464
H(20)	0.174	2.250	-1.555
H(21)	0.353	3.458	-0.304
C(22)	-1.024	1.883	0.241
C(23)	-0.883	0.471	0.895
C(24)	-2.149	-0.080	1.610
H(25)	-1.855	-0.588	2.539
H(26)	-2.853	0.715	1.882
C(27)	-2.772	-1.111	0.638
H(28)	-3.553	-0.661	-0.000
H(29)	-3.229	-1.983	1.124
C(30)	-2.230	2.000	-0.704
H(31)	-3.184	1.801	-0.199
C(32)	-1.790	-2.544	-1.350
H(33)	-0.835	-2.836	-1.800
H(34)	-2.302	-3.429	-0.955
H(35)	-2.423	-2.131	-2.152
H(36)	-2.286	3.016	-1.115
H(37)	-2.143	1.315	-1.563
H(38)	-0.558	-1.706	0.488
H(39)	-1.206	2.544	1.106
H(40)	-0.047	0.536	1.603

Transition State C-TS4 = G-TS5 (–586,3367343 Hartree)

C(1)	1.943	-1.473	-0.240
C(2)	0.627	-0.787	-0.584
H(3)	0.568	-0.618	-1.667
C(4)	-0.736	-1.160	-0.011
H(5)	-1.234	-1.818	-0.736
H(6)	-0.651	-1.747	0.914
C(7)	-1.622	0.099	0.280
H(8)	-1.419	0.403	1.321
C(9)	-3.096	-0.300	0.213
C(10)	-3.811	-0.460	1.339
H(11)	-3.376	-0.290	2.326
H(12)	-4.859	-0.761	1.311
C(13)	-3.701	-0.511	-1.158
H(14)	-3.112	-1.213	-1.771
H(15)	-4.721	-0.905	-1.087
H(16)	-3.746	0.432	-1.725
C(17)	-1.314	1.342	-0.608
H(18)	-1.208	1.060	-1.668
H(19)	-2.200	1.991	-0.563
C(20)	-0.121	2.215	-0.162
H(21)	-0.176	2.393	0.924
H(22)	-0.199	3.204	-0.638
C(23)	1.331	1.723	-0.515
C(24)	1.392	0.343	0.069
C(25)	1.638	-0.030	1.539
H(26)	2.424	0.573	2.005
H(27)	0.720	0.085	2.130
C(28)	2.080	-1.499	1.288
H(29)	1.429	-2.265	1.729
H(30)	3.109	-1.694	1.613
C(31)	2.408	2.690	-0.012
H(32)	3.416	2.327	-0.252
C(33)	2.676	-2.377	-1.189
H(34)	2.608	-2.034	-2.229
H(35)	3.725	-2.517	-0.906
H(36)	2.166	-3.354	-1.133
H(37)	2.284	3.671	-0.486
H(38)	2.341	2.838	1.074
H(39)	2.560	-0.270	-0.362
H(40)	1.381	1.634	-1.613

Intermediate D (–586,3794497 Hartree)

C(1)	-3.164	-0.800	0.496
H(2)	-3.348	-0.176	1.384
H(3)	-4.034	-1.459	0.378
C(4)	-2.927	0.080	-0.746
H(5)	-3.142	-0.478	-1.666
H(6)	-3.560	0.976	-0.770
C(7)	-1.413	0.426	-0.751
C(8)	-0.987	1.719	-0.259
C(9)	-1.812	2.505	0.690
H(10)	-2.629	1.951	1.159
H(11)	-1.191	3.010	1.443
H(12)	-2.260	3.320	0.088
C(13)	0.339	2.266	-0.627
H(14)	0.399	3.329	-0.363
H(15)	0.510	2.147	-1.708
C(16)	1.500	1.497	0.110
H(17)	1.346	1.539	1.199
H(18)	2.401	2.085	-0.110
C(19)	1.706	0.055	-0.394
H(20)	1.611	0.090	-1.493
C(21)	3.122	-0.437	-0.083
C(22)	3.894	-0.939	-1.062
H(23)	4.895	-1.318	-0.857
H(24)	3.551	-0.990	-2.097
C(25)	3.601	-0.340	1.350
H(26)	2.894	-0.796	2.060
H(27)	3.725	0.709	1.663
H(28)	4.568	-0.838	1.477
C(29)	-1.747	-2.776	-0.297
H(30)	-2.615	-3.434	-0.155
H(31)	-0.848	-3.376	-0.113
H(32)	-1.736	-2.467	-1.352
C(33)	-1.850	-1.584	0.667
C(34)	-0.741	-0.499	0.446
H(35)	-0.709	0.101	1.371
C(36)	0.670	-0.987	0.127
H(37)	1.048	-1.444	1.055
H(38)	0.628	-1.793	-0.616
H(39)	-0.901	0.155	-1.682
H(40)	-1.742	-1.951	1.700

Intermediate D1 (–586,3769981 Hartree)

C(1)	2.431	-1.650	-1.031
H(2)	2.084	-1.782	-2.066
H(3)	3.341	-2.248	-0.903
C(4)	2.673	-0.150	-0.751
H(5)	3.434	0.002	0.043
H(6)	3.043	0.476	-1.581
C(7)	1.413	0.366	-0.175
C(8)	1.202	1.721	0.341
C(9)	2.381	2.693	0.207
H(10)	2.597	2.912	-0.848
H(11)	2.134	3.642	0.698
H(12)	3.295	2.305	0.675
C(13)	-0.164	2.405	-0.085
H(14)	0.091	3.072	-0.921
H(15)	-0.454	3.047	0.757
C(16)	-1.335	1.512	-0.513
H(17)	-1.094	1.032	-1.475
H(18)	-2.182	2.177	-0.738
C(19)	-1.826	0.457	0.508
H(20)	-2.164	1.003	1.403
C(21)	-3.031	-0.299	-0.053
C(22)	-4.252	-0.129	0.480
H(23)	-5.123	-0.654	0.088
H(24)	-4.421	0.541	1.325
C(25)	-2.798	-1.237	-1.219
H(26)	-2.081	-2.036	-0.966
H(27)	-2.397	-0.711	-2.101
H(28)	-3.728	-1.725	-1.529
C(29)	1.827	-2.381	1.370
H(30)	2.450	-3.283	1.325
H(31)	1.002	-2.581	2.066
H(32)	2.439	-1.575	1.802
C(33)	1.308	-2.031	-0.032
C(34)	0.436	-0.722	-0.037
H(35)	0.004	-0.710	-1.074
C(36)	-0.702	-0.528	0.972
H(37)	-1.147	-1.508	1.192
H(38)	-0.266	-0.180	1.921
H(39)	0.701	-2.863	-0.411
H(40)	1.052	1.472	1.421

Intermediate D2 (–586,3801467 Hartree)

C(1)	2.950	-1.456	-0.796
H(2)	2.947	-1.557	-1.892
H(3)	3.809	-2.018	-0.410
C(4)	2.957	0.027	-0.383
H(5)	3.313	0.134	0.651
H(6)	3.600	0.651	-1.012
C(7)	1.462	0.458	-0.470
C(8)	0.999	1.636	0.464
C(9)	2.119	2.685	0.607
H(10)	2.405	3.090	-0.373
H(11)	1.755	3.519	1.220
H(12)	3.016	2.287	1.093
C(13)	-0.291	2.343	-0.006
H(14)	-0.026	3.072	-0.789
H(15)	-0.652	2.935	0.849
C(16)	-1.448	1.471	-0.534
H(17)	-1.296	1.238	-1.601
H(18)	-2.367	2.072	-0.491
C(19)	-1.671	0.155	0.245
H(20)	-1.406	0.307	1.303
C(21)	-3.106	-0.350	0.192
C(22)	-3.765	-0.645	1.329
H(23)	-4.796	-0.999	1.313
H(24)	-3.302	-0.524	2.310
C(25)	-3.736	-0.515	-1.170
H(26)	-3.152	-1.195	-1.814
H(27)	-3.794	0.444	-1.707
H(28)	-4.749	-0.925	-1.097
C(29)	1.691	-2.294	1.314
H(30)	2.435	-3.091	1.431
H(31)	0.728	-2.644	1.702
H(32)	2.018	-1.427	1.901
C(33)	1.608	-1.970	-0.228
C(34)	0.697	-0.808	-0.333
H(35)	1.250	0.779	-1.518
C(36)	-0.754	-0.977	-0.315
H(37)	-0.993	-1.074	-1.401
H(38)	-1.018	-1.963	0.102
H(39)	1.195	-2.866	-0.719
H(40)	0.823	1.193	1.459

Intermediate D3 (–586,374366 Hartree)

C(1)	-2.644	1.679	0.506
H(2)	-3.448	1.915	-0.226
H(3)	-2.819	2.376	1.341
C(4)	-2.630	0.170	0.826
H(5)	-2.203	0.003	1.827
H(6)	-3.637	-0.259	0.825
C(7)	-1.708	-0.443	-0.260
C(8)	-1.111	-1.810	0.126
C(9)	-2.218	-2.878	0.197
H(10)	-2.695	-3.014	-0.785
H(11)	-1.801	-3.846	0.501
H(12)	-3.000	-2.613	0.919
C(13)	0.021	-2.279	-0.812
H(14)	-0.391	-2.464	-1.816
H(15)	0.353	-3.260	-0.440
C(16)	1.261	-1.353	-0.930
H(17)	1.194	-0.716	-1.826
H(18)	2.149	-1.980	-1.099
C(19)	1.525	-0.490	0.315
H(20)	1.351	-1.110	1.206
C(21)	2.934	0.071	0.404
C(22)	3.585	0.071	1.587
H(23)	4.598	0.463	1.680
H(24)	3.132	-0.344	2.489
C(25)	3.548	0.647	-0.851
H(26)	2.880	1.380	-1.332
H(27)	3.733	-0.139	-1.598
H(28)	4.500	1.146	-0.641
C(29)	-0.936	3.293	-0.619
H(30)	-1.737	3.795	-1.190
H(31)	-0.015	3.294	-1.209
H(32)	-0.807	3.915	0.283
C(33)	-1.394	1.942	-0.243
C(34)	-0.694	0.701	-0.547
H(35)	-2.295	-0.574	-1.188
C(36)	0.557	0.764	0.459
H(37)	1.117	1.680	0.237
H(38)	0.209	0.842	1.496
H(39)	-0.249	0.705	-1.552
H(40)	-0.692	-1.707	1.143

Intermediate D4 (–586,3826523 Hartree)

C(1)	3.065	-0.945	-0.385
H(2)	3.345	-0.265	-1.202
H(3)	3.865	-1.687	-0.283
C(4)	2.796	-0.157	0.912
H(5)	2.834	-0.824	1.785
H(6)	3.507	0.653	1.097
C(7)	1.341	0.332	0.735
C(8)	1.140	1.610	-0.284
C(9)	2.324	2.577	-0.116
H(10)	3.279	2.145	-0.434
H(11)	2.135	3.454	-0.749
H(12)	2.416	2.917	0.924
C(13)	-0.205	2.306	-0.002
H(14)	-0.189	3.255	-0.559
H(15)	-0.242	2.581	1.065
C(16)	-1.487	1.552	-0.392
H(17)	-1.500	1.387	-1.483
H(18)	-2.341	2.206	-0.169
C(19)	-1.706	0.207	0.334
H(20)	-1.480	0.345	1.405
C(21)	-3.147	-0.299	0.235
C(22)	-3.811	-0.667	1.344
H(23)	-4.832	-1.046	1.295
H(24)	-3.362	-0.594	2.337
C(25)	-3.774	-0.372	-1.139
H(26)	-3.171	-0.966	-1.845
H(27)	-3.879	0.628	-1.587
H(28)	-4.772	-0.824	-1.096
C(29)	1.555	-3.021	-0.026
H(30)	2.359	-3.652	-0.421
H(31)	0.592	-3.485	-0.267
H(32)	1.663	-2.966	1.064
C(33)	1.694	-1.606	-0.694
C(34)	0.689	-0.705	-0.054
H(35)	1.144	1.252	-1.330
C(36)	-0.761	-0.884	-0.213
H(37)	-0.981	-1.127	-1.268
H(38)	-0.969	-1.846	0.307
H(39)	0.813	0.623	1.652
H(40)	1.482	-1.739	-1.767

Intermediate D5 (–586,3780835 Hartree)

C(1)	-2.762	1.579	0.600
H(2)	-3.627	2.218	0.374
H(3)	-2.485	1.809	1.655
C(4)	-2.926	0.049	0.434
H(5)	-3.499	-0.397	1.253
H(6)	-3.462	-0.159	-0.505
C(7)	-1.467	-0.468	0.351
C(8)	-1.293	-1.806	-0.399
C(9)	-2.263	-2.877	0.130
H(10)	-3.314	-2.595	-0.014
H(11)	-2.105	-3.831	-0.390
H(12)	-2.103	-3.054	1.204
C(13)	0.153	-2.346	-0.340
H(14)	0.198	-3.230	-0.994
H(15)	0.345	-2.719	0.681
C(16)	1.306	-1.398	-0.725
H(17)	1.138	-0.955	-1.722
H(18)	2.209	-2.015	-0.831
C(19)	1.604	-0.303	0.326
H(20)	1.356	-0.714	1.317
C(21)	3.057	0.159	0.362
C(22)	3.663	0.388	1.546
H(23)	4.699	0.723	1.600
H(24)	3.149	0.224	2.495
C(25)	3.767	0.378	-0.955
H(26)	3.177	0.999	-1.647
H(27)	3.942	-0.577	-1.473
H(28)	4.738	0.865	-0.809
C(29)	-1.254	3.224	-0.740
H(30)	-2.105	3.552	-1.362
H(31)	-0.327	3.272	-1.319
H(32)	-1.218	3.964	0.080
C(33)	-1.551	1.893	-0.187
C(34)	-0.708	0.707	-0.329
H(35)	-1.542	-1.609	-1.460
C(36)	0.764	1.002	0.155
H(37)	1.245	1.667	-0.574
H(38)	0.740	1.538	1.114
H(39)	-1.081	-0.582	1.381
H(40)	-0.611	0.520	-1.421

Transition State D-TS1 (–586,3698867 Hartree)

C(1)	2.465	-1.677	-0.944
H(2)	2.146	-1.834	-1.985
H(3)	3.356	-2.293	-0.772
C(4)	2.751	-0.172	-0.712
H(5)	3.551	-0.029	0.029
H(6)	3.064	0.367	-1.618
C(7)	1.426	0.385	-0.193
C(8)	1.188	1.763	0.057
C(9)	2.304	2.765	-0.107
H(10)	2.196	3.208	-1.110
H(11)	2.208	3.582	0.620
H(12)	3.305	2.329	-0.041
C(13)	-0.184	2.340	0.375
H(14)	-0.150	3.375	0.002
H(15)	-0.289	2.444	1.468
C(16)	-1.393	1.604	-0.232
H(17)	-1.159	1.325	-1.271
H(18)	-2.227	2.317	-0.297
C(19)	-1.870	0.376	0.573
H(20)	-2.279	0.759	1.523
C(21)	-3.015	-0.333	-0.151
C(22)	-4.274	-0.221	0.306
H(23)	-5.109	-0.703	-0.202
H(24)	-4.510	0.354	1.203
C(25)	-2.697	-1.150	-1.386
H(26)	-2.045	-2.007	-1.150
H(27)	-2.182	-0.557	-2.159
H(28)	-3.610	-1.553	-1.840
C(29)	1.796	-2.264	1.458
H(30)	2.480	-3.122	1.477
H(31)	0.966	-2.485	2.140
H(32)	2.346	-1.405	1.875
C(33)	1.306	-2.007	0.023
C(34)	0.405	-0.742	-0.105
H(35)	-0.045	-0.773	-1.115
C(36)	-0.703	-0.578	0.950
H(37)	-1.115	-1.570	1.177
H(38)	-0.240	-0.233	1.890
H(39)	1.568	0.865	1.007
H(40)	0.742	-2.885	-0.322

Transition State D-TS2 (–586,3722506 Hartree)

C(1)	2.975	-1.187	-0.873
H(2)	2.918	-1.092	-1.969
H(3)	3.898	-1.727	-0.635
C(4)	2.922	0.217	-0.233
H(5)	3.398	0.232	0.763
H(6)	3.403	1.006	-0.822
C(7)	1.444	0.467	0.006
C(8)	0.853	1.786	0.472
C(9)	1.906	2.896	0.627
H(10)	2.350	3.170	-0.339
H(11)	1.424	3.794	1.034
H(12)	2.712	2.613	1.315
C(13)	-0.323	2.277	-0.422
H(14)	0.097	2.750	-1.324
H(15)	-0.814	3.080	0.145
C(16)	-1.378	1.227	-0.836
H(17)	-1.076	0.709	-1.765
H(18)	-2.294	1.769	-1.106
C(19)	-1.699	0.186	0.264
H(20)	-1.594	0.674	1.245
C(21)	-3.130	-0.351	0.182
C(22)	-3.908	-0.366	1.277
H(23)	-4.928	-0.751	1.245
H(24)	-3.557	0.013	2.239
C(25)	-3.620	-0.855	-1.158
H(26)	-2.968	-1.636	-1.581
H(27)	-3.657	-0.046	-1.904
H(28)	-4.627	-1.277	-1.076
C(29)	1.958	-2.665	0.990
H(30)	2.718	-3.438	0.825
H(31)	1.049	-3.158	1.355
H(32)	2.326	-1.991	1.776
C(33)	1.711	-1.912	-0.340
C(34)	0.739	-0.766	-0.071
H(35)	0.864	0.065	-1.116
C(36)	-0.714	-1.018	0.218
H(37)	-1.060	-1.764	-0.515
H(38)	-0.737	-1.548	1.186
H(39)	1.294	-2.618	-1.072
H(40)	0.439	1.562	1.471

Transition State D-TS3 (–586,3623927 Hartree)

C(1)	3.143	-1.307	-0.063
H(2)	3.644	-1.684	-0.970
H(3)	3.709	-1.728	0.782
C(4)	2.998	0.228	-0.006
H(5)	3.159	0.555	1.030
H(6)	3.737	0.746	-0.627
C(7)	1.534	0.576	-0.427
C(8)	0.938	1.778	0.361
C(9)	1.968	2.921	0.488
H(10)	2.322	3.239	-0.504
H(11)	1.493	3.789	0.962
H(12)	2.841	2.657	1.095
C(13)	-0.342	2.362	-0.277
H(14)	-0.051	2.996	-1.130
H(15)	-0.787	3.041	0.466
C(16)	-1.413	1.364	-0.747
H(17)	-1.161	0.967	-1.744
H(18)	-2.356	1.912	-0.876
C(19)	-1.628	0.189	0.235
H(20)	-1.362	0.517	1.252
C(21)	-3.075	-0.299	0.282
C(22)	-3.731	-0.354	1.455
H(23)	-4.768	-0.684	1.511
H(24)	-3.258	-0.059	2.394
C(25)	-3.733	-0.700	-1.020
H(26)	-3.229	-1.558	-1.495
H(27)	-3.712	0.119	-1.755
H(28)	-4.779	-0.985	-0.864
C(29)	1.409	-3.251	0.291
H(30)	2.159	-3.922	-0.145
H(31)	0.407	-3.554	-0.027
H(32)	1.465	-3.371	1.386
C(33)	1.727	-1.821	-0.005
C(34)	0.782	-0.772	-0.239
H(35)	1.496	0.844	-1.498
C(36)	-0.723	-1.014	-0.138
H(37)	-1.077	-1.387	-1.112
H(38)	-0.899	-1.826	0.581
H(39)	1.170	-1.443	-1.230
H(40)	0.706	1.419	1.380

Transition State D-TS4 (–586,3250531 Hartree)

C(1)	2.048	-1.808	1.029
H(2)	3.049	-2.058	1.398
H(3)	1.422	-2.708	1.094
C(4)	1.442	-0.550	1.704
H(5)	0.484	-0.711	2.215
H(6)	2.129	-0.080	2.419
C(7)	1.245	0.301	0.443
C(8)	1.018	1.796	0.418
C(9)	2.313	2.622	0.259
H(10)	2.754	2.456	-0.735
H(11)	2.092	3.691	0.355
H(12)	3.056	2.356	1.022
C(13)	-0.048	2.163	-0.642
H(14)	0.414	2.037	-1.634
H(15)	-0.229	3.244	-0.549
C(16)	-1.429	1.425	-0.595
H(17)	-1.781	1.320	-1.631
H(18)	-2.154	2.087	-0.100
C(19)	-1.561	0.055	0.134
H(20)	-1.222	0.216	1.172
C(21)	-3.023	-0.386	0.227
C(22)	-3.560	-0.696	1.419
H(23)	-4.593	-1.033	1.508
H(24)	-2.988	-0.617	2.346
C(25)	-3.822	-0.468	-1.056
H(26)	-3.304	-1.049	-1.834
H(27)	-4.004	0.532	-1.480
H(28)	-4.798	-0.936	-0.883
C(29)	2.964	-1.751	-1.496
H(30)	3.988	-1.918	-1.147
H(31)	2.963	-1.081	-2.365
H(32)	2.545	-2.717	-1.829
C(33)	2.059	-1.267	-0.401
C(34)	0.720	-0.579	-0.665
H(35)	0.782	-0.051	-1.624
C(36)	-0.691	-1.108	-0.441
H(37)	-1.099	-1.478	-1.391
H(38)	-0.723	-1.949	0.266
H(39)	2.536	-0.051	-0.047
H(40)	0.631	1.995	1.438

Transition State D-TS5 (–586,3678619 Hartree)

C(1)	3.183	-0.864	-0.048
H(2)	3.781	-0.242	-0.726
H(3)	3.808	-1.732	0.197
C(4)	2.774	-0.085	1.228
H(5)	2.774	-0.748	2.104
H(6)	3.454	0.745	1.456
C(7)	1.333	0.389	0.999
C(8)	1.053	1.455	-0.050
C(9)	2.195	2.188	-0.735
H(10)	3.064	1.567	-0.956
H(11)	1.854	2.675	-1.655
H(12)	2.514	2.974	-0.033
C(13)	-0.240	2.268	0.127
H(14)	-0.134	3.198	-0.447
H(15)	-0.301	2.544	1.191
C(16)	-1.538	1.556	-0.299
H(17)	-1.545	1.439	-1.396
H(18)	-2.382	2.213	-0.049
C(19)	-1.731	0.186	0.378
H(20)	-1.551	0.311	1.459
C(21)	-3.147	-0.367	0.212
C(22)	-3.821	-0.816	1.284
H(23)	-4.826	-1.227	1.191
H(24)	-3.396	-0.782	2.289
C(25)	-3.742	-0.389	-1.179
H(26)	-3.099	-0.916	-1.903
H(27)	-3.887	0.629	-1.572
H(28)	-4.718	-0.887	-1.181
C(29)	1.536	-2.800	-0.430
H(30)	2.360	-3.418	-0.806
H(31)	0.614	-3.136	-0.918
H(32)	1.448	-2.979	0.651
C(33)	1.862	-1.320	-0.735
C(34)	0.751	-0.420	-0.141
H(35)	0.720	0.591	-0.991
C(36)	-0.708	-0.848	-0.155
H(37)	-0.976	-1.160	-1.175
H(38)	-0.760	-1.749	0.474
H(39)	0.708	0.495	1.889
H(40)	1.912	-1.205	-1.828

Transition State D-TS6 (–586,3702504 Hartree)

C(1)	-2.988	1.332	-0.018
H(2)	-3.429	1.154	-1.012
H(3)	-3.579	2.144	0.430
C(4)	-2.901	0.065	0.866
H(5)	-3.031	0.351	1.919
H(6)	-3.684	-0.660	0.629
C(7)	-1.460	-0.495	0.658
C(8)	-1.342	-1.616	-0.427
C(9)	-2.288	-2.792	-0.119
H(10)	-3.347	-2.515	-0.167
H(11)	-2.132	-3.598	-0.847
H(12)	-2.086	-3.202	0.881
C(13)	0.091	-2.169	-0.599
H(14)	0.047	-2.902	-1.418
H(15)	0.350	-2.747	0.304
C(16)	1.240	-1.187	-0.891
H(17)	0.992	-0.536	-1.750
H(18)	2.103	-1.784	-1.216
C(19)	1.676	-0.339	0.329
H(20)	1.535	-0.957	1.231
C(21)	3.149	0.070	0.281
C(22)	3.949	-0.148	1.338
H(23)	5.000	0.141	1.325
H(24)	3.585	-0.633	2.246
C(25)	3.659	0.714	-0.991
H(26)	3.074	1.603	-1.279
H(27)	3.605	0.020	-1.843
H(28)	4.703	1.030	-0.883
C(29)	-1.188	3.177	-0.577
H(30)	-1.844	3.529	-1.383
H(31)	-0.139	3.285	-0.874
H(32)	-1.360	3.833	0.291
C(33)	-1.552	1.790	-0.152
C(34)	-0.666	0.759	0.271
H(35)	-1.655	-1.164	-1.391
C(36)	0.811	0.934	0.498
H(37)	1.193	1.740	-0.144
H(38)	0.910	1.312	1.531
H(39)	-1.043	-0.890	1.598
H(40)	-0.916	0.883	-0.985

Intermediate E (–586,3746648 Hartree)

C(1)	3.186	-1.016	-0.464
H(2)	3.159	-1.392	-1.514
H(3)	4.108	-1.442	-0.041
C(4)	3.002	0.516	-0.442
H(5)	3.392	0.915	0.506
H(6)	3.536	1.014	-1.258
C(7)	1.469	0.697	-0.529
C(8)	0.946	2.067	-0.040
C(9)	1.058	2.247	1.485
H(10)	0.823	3.284	1.756
H(11)	0.345	1.615	2.036
H(12)	2.066	2.035	1.869
C(13)	-0.489	2.395	-0.557
H(14)	-0.407	3.008	-1.468
H(15)	-0.982	3.036	0.190
C(16)	-1.431	1.221	-0.892
H(17)	-1.125	0.740	-1.836
H(18)	-2.419	1.659	-1.095
C(19)	-1.596	0.133	0.200
H(20)	-1.478	0.602	1.190
C(21)	-2.982	-0.512	0.165
C(22)	-3.734	-0.561	1.282
H(23)	-4.731	-1.001	1.276
H(24)	-3.385	-0.146	2.230
C(25)	-3.470	-1.086	-1.147
H(26)	-2.820	-1.897	-1.510
H(27)	-3.490	-0.324	-1.940
H(28)	-4.482	-1.495	-1.048
C(29)	1.766	-2.990	0.461
H(30)	2.707	-3.540	0.563
H(31)	1.198	-3.441	-0.378
H(32)	1.121	-3.125	1.340
C(33)	1.954	-1.571	0.126
C(34)	0.928	-0.535	0.267
H(35)	1.058	-0.282	1.355
C(36)	-0.539	-0.996	0.078
H(37)	-0.617	-1.459	-0.918
H(38)	-0.775	-1.777	0.816
H(39)	1.170	0.555	-1.583
H(40)	1.624	2.811	-0.492

Intermediate E1 (–586,3808457 Hartree)

C(1)	-3.232	0.930	-0.434
H(2)	-3.214	1.360	-1.448
H(3)	-4.174	1.227	0.040
C(4)	-3.003	-0.589	-0.467
H(5)	-3.369	-1.051	0.462
H(6)	-3.503	-1.091	-1.303
C(7)	-1.459	-0.740	-0.551
C(8)	-0.862	-2.064	0.029
C(9)	-0.919	-2.062	1.570
H(10)	-0.570	-3.026	1.960
H(11)	-0.287	-1.281	2.016
H(12)	-1.942	-1.915	1.942
C(13)	0.534	-2.424	-0.546
H(14)	0.398	-3.020	-1.461
H(15)	1.032	-3.087	0.178
C(16)	1.489	-1.264	-0.892
H(17)	1.191	-0.789	-1.842
H(18)	2.481	-1.697	-1.080
C(19)	1.625	-0.177	0.200
H(20)	1.536	-0.653	1.188
C(21)	2.964	0.552	0.163
C(22)	3.725	0.628	1.271
H(23)	4.687	1.140	1.264
H(24)	3.419	0.168	2.212
C(25)	3.379	1.182	-1.145
H(26)	2.634	1.914	-1.505
H(27)	3.480	0.430	-1.942
H(28)	4.334	1.710	-1.052
C(29)	-1.744	2.967	0.346
H(30)	-2.655	3.491	0.657
H(31)	-1.488	3.306	-0.667
H(32)	-0.938	3.264	1.027
C(33)	-2.001	1.456	0.354
C(34)	-0.907	0.527	-0.008
C(35)	0.502	0.893	0.097
H(36)	0.639	1.428	-0.878
H(37)	0.642	1.701	0.834
H(38)	-1.165	-0.688	-1.625
H(39)	-1.562	-2.842	-0.316
H(40)	-2.153	1.125	1.418

Intermediate E2 (–586,3752933 Hartree)

C(1)	-3.241	0.956	-0.272
H(2)	-3.271	1.280	-1.325
H(3)	-4.182	1.262	0.199
C(4)	-3.012	-0.568	-0.205
H(5)	-3.356	-0.964	0.778
H(6)	-3.513	-1.202	-0.952
C(7)	-1.544	-0.764	-0.158
C(8)	-0.902	-2.083	-0.209
C(9)	-0.713	-2.467	1.316
H(10)	-0.248	-3.460	1.324
H(11)	-0.062	-1.772	1.852
H(12)	-1.677	-2.531	1.834
C(13)	0.421	-2.198	-1.037
H(14)	0.134	-2.499	-2.055
H(15)	0.973	-3.045	-0.606
C(16)	1.361	-0.981	-1.134
H(17)	0.983	-0.259	-1.876
H(18)	2.306	-1.351	-1.556
C(19)	1.658	-0.276	0.207
H(20)	1.775	-1.062	0.969
C(21)	2.969	0.508	0.175
C(22)	3.887	0.340	1.142
H(23)	4.820	0.904	1.146
H(24)	3.739	-0.370	1.957
C(25)	3.175	1.475	-0.971
H(26)	2.326	2.167	-1.092
H(27)	3.281	0.943	-1.930
H(28)	4.078	2.079	-0.826
C(29)	-1.692	2.997	0.082
H(30)	-2.543	3.634	0.355
H(31)	-1.510	3.122	-0.995
H(32)	-0.815	3.371	0.625
C(33)	-1.992	1.537	0.423
C(34)	-0.872	0.531	-0.003
C(35)	0.506	0.676	0.667
H(36)	0.811	1.716	0.491
H(37)	0.362	0.597	1.755
H(38)	-0.733	0.716	-1.110
H(39)	-1.624	-2.814	-0.604
H(40)	-2.111	1.436	1.518

Intermediate E3 (–586,3763253 Hartree)

C(1)	-2.951	1.178	0.268
H(2)	-3.415	1.909	-0.410
H(3)	-3.428	1.308	1.248
C(4)	-3.146	-0.261	-0.296
H(5)	-3.578	-0.944	0.447
H(6)	-3.837	-0.262	-1.148
C(7)	-1.765	-0.761	-0.789
C(8)	-1.014	-1.692	0.036
C(9)	-1.350	-1.935	1.456
H(10)	-0.522	-2.369	2.027
H(11)	-1.762	-1.050	1.956
H(12)	-2.172	-2.678	1.453
C(13)	0.173	-2.354	-0.529
H(14)	0.178	-2.299	-1.623
H(15)	0.209	-3.409	-0.216
C(16)	1.479	-1.663	0.053
H(17)	2.289	-1.985	-0.614
H(18)	1.688	-2.079	1.046
C(19)	1.417	-0.121	0.145
H(20)	0.882	0.129	1.077
C(21)	2.837	0.424	0.287
C(22)	3.290	0.827	1.488
H(23)	4.304	1.205	1.617
H(24)	2.659	0.797	2.379
C(25)	3.699	0.479	-0.956
H(26)	3.306	1.202	-1.689
H(27)	3.748	-0.492	-1.473
H(28)	4.724	0.779	-0.712
C(29)	-1.042	2.917	0.280
H(30)	-1.543	3.465	1.089
H(31)	-1.346	3.375	-0.672
H(32)	0.039	3.059	0.403
C(33)	-1.430	1.434	0.328
C(34)	-0.876	0.630	-0.863
C(35)	0.643	0.532	-1.028
H(36)	0.886	0.035	-1.980
H(37)	0.993	1.568	-1.140
H(38)	-1.282	1.072	-1.790
H(39)	-1.763	-1.139	-1.823
H(40)	-1.031	1.009	1.266

Intermediate E4 (–586,3858585 Hartree)

C(1)	-3.082	-1.394	-0.113
H(2)	-3.917	-1.878	0.404
C(3)	-3.015	0.122	0.158
C(4)	-1.585	0.491	0.083
C(5)	-1.068	1.862	0.185
H(6)	-0.620	1.829	1.210
C(7)	-2.130	2.973	0.145
H(8)	-1.662	3.934	0.387
H(9)	-2.939	2.806	0.867
H(10)	-2.571	3.060	-0.857
C(11)	0.153	2.141	-0.768
C(12)	1.513	1.600	-0.304
H(13)	2.290	2.075	-0.920
C(14)	1.705	0.072	-0.397
H(15)	1.533	-0.221	-1.449
C(16)	0.691	-0.719	0.465
H(17)	1.016	-1.767	0.505
H(18)	0.693	-0.353	1.504
C(19)	-0.758	-0.716	-0.055
C(20)	-1.699	-1.904	0.343
C(21)	-1.290	-3.253	-0.251
H(22)	-0.301	-3.572	0.099
H(23)	-1.269	-3.210	-1.350
H(24)	-2.008	-4.030	0.042
H(25)	-1.684	-1.971	1.446
H(26)	-0.718	-0.751	-1.182
C(27)	3.136	-0.335	-0.042
C(28)	3.640	-0.020	1.350
H(29)	4.654	-0.408	1.498
H(30)	3.670	1.065	1.537
H(31)	2.999	-0.458	2.131
C(32)	3.906	-0.958	-0.950
H(33)	1.693	1.937	0.730
H(34)	0.214	3.235	-0.838
H(35)	-0.094	1.780	-1.778
H(36)	-3.670	0.784	-0.427
H(37)	-3.280	0.344	1.218
H(38)	-3.209	-1.570	-1.193
H(39)	3.546	-1.181	-1.956
H(40)	4.927	-1.262	-0.717

Intermediate E5 (–586,3821911 Hartree)

C(1)	-3.268	-0.928	-0.031
H(2)	-4.058	-1.141	0.700
H(3)	-3.634	-1.247	-1.017
C(4)	-2.951	0.598	-0.075
H(5)	-3.056	1.001	-1.091
H(6)	-3.569	1.205	0.596
C(7)	-1.419	0.641	0.370
C(8)	-0.807	1.899	-0.048
C(9)	-1.165	3.160	0.643
H(10)	-0.270	3.771	0.835
H(11)	-1.769	3.761	-0.063
H(12)	-1.738	3.013	1.563
C(13)	0.208	1.919	-1.097
C(14)	1.519	1.339	-0.371
C(15)	1.667	-0.195	-0.441
H(16)	1.585	-0.474	-1.506
C(17)	3.086	-0.533	0.019
C(18)	4.049	-0.764	-0.895
H(19)	5.073	-0.992	-0.598
H(20)	3.840	-0.747	-1.967
C(21)	3.371	-0.567	1.504
H(22)	2.862	-1.413	1.990
H(23)	3.025	0.344	2.015
H(24)	4.445	-0.670	1.698
C(25)	-1.860	-3.075	-0.307
H(26)	-2.693	-3.706	0.030
H(27)	-0.929	-3.585	-0.024
H(28)	-1.901	-3.023	-1.405
C(29)	-1.952	-1.679	0.313
C(30)	-0.850	-0.691	-0.145
H(31)	-0.871	-0.684	-1.253
C(32)	0.579	-0.979	0.330
H(33)	0.648	-0.769	1.410
H(34)	0.796	-2.050	0.209
H(35)	-1.878	-1.775	1.412
H(36)	-0.021	1.243	-1.930
H(37)	0.410	2.930	-1.468
H(38)	1.565	1.690	0.672
H(39)	2.357	1.819	-0.895
H(40)	-1.449	0.632	1.477

Transition State E-TS1 (–586,3707139 Hartree)

C(1)	-3.294	0.869	-0.349
H(2)	-3.514	1.344	-1.323
H(3)	-4.132	1.115	0.314
C(4)	-2.996	-0.639	-0.500
H(5)	-3.345	-1.173	0.394
H(6)	-3.510	-1.080	-1.360
C(7)	-1.449	-0.765	-0.624
C(8)	-0.872	-2.034	0.062
C(9)	-0.966	-1.951	1.599
H(10)	-0.631	-2.895	2.046
H(11)	-0.332	-1.159	2.024
H(12)	-1.994	-1.782	1.951
C(13)	0.548	-2.420	-0.439
H(14)	0.446	-3.067	-1.323
H(15)	1.022	-3.040	0.336
C(16)	1.511	-1.277	-0.819
H(17)	1.246	-0.868	-1.809
H(18)	2.509	-1.720	-0.944
C(19)	1.612	-0.113	0.198
H(20)	1.468	-0.512	1.213
C(21)	2.987	0.559	0.173
C(22)	3.719	0.640	1.297
H(23)	4.705	1.105	1.300
H(24)	3.364	0.235	2.247
C(25)	3.484	1.107	-1.147
H(26)	2.826	1.899	-1.544
H(27)	3.531	0.325	-1.920
H(28)	4.486	1.538	-1.044
C(29)	-1.876	2.927	0.471
H(30)	-2.724	3.241	1.090
H(31)	-1.916	3.514	-0.461
H(32)	-0.935	3.173	0.975
C(33)	-1.989	1.486	0.087
C(34)	-0.916	0.566	-0.094
H(35)	-1.472	0.671	1.078
C(36)	0.535	0.970	-0.059
H(37)	0.706	1.397	-1.065
H(38)	0.681	1.807	0.638
H(39)	-1.165	-0.773	-1.693
H(40)	-1.546	-2.850	-0.247

Transition State E-TS2 (–586,3711711 Hartree)

C(1)	3.279	-0.919	-0.226
H(2)	3.329	-1.240	-1.279
H(3)	4.214	-1.223	0.257
C(4)	3.027	0.599	-0.155
H(5)	3.379	1.022	0.803
H(6)	3.492	1.189	-0.955
C(7)	1.518	0.741	-0.125
C(8)	0.847	2.089	-0.214
C(9)	0.677	2.593	1.249
H(10)	0.260	3.607	1.223
H(11)	0.001	1.956	1.831
H(12)	1.640	2.641	1.774
C(13)	-0.462	2.166	-1.063
H(14)	-0.190	2.482	-2.081
H(15)	-1.061	2.984	-0.639
C(16)	-1.348	0.910	-1.172
H(17)	-0.928	0.196	-1.904
H(18)	-2.303	1.230	-1.610
C(19)	-1.612	0.191	0.172
H(20)	-1.608	0.945	0.972
C(21)	-2.975	-0.503	0.222
C(22)	-3.810	-0.271	1.249
H(23)	-4.785	-0.755	1.310
H(24)	-3.551	0.415	2.058
C(25)	-3.337	-1.439	-0.910
H(26)	-2.612	-2.262	-1.025
H(27)	-3.365	-0.914	-1.877
H(28)	-4.322	-1.892	-0.749
C(29)	1.725	-2.986	0.009
H(30)	2.597	-3.624	0.196
H(31)	1.499	-3.034	-1.066
H(32)	0.879	-3.412	0.562
C(33)	2.033	-1.550	0.442
C(34)	0.930	-0.515	0.199
C(35)	-0.509	-0.852	0.477
H(36)	-0.723	-1.783	-0.071
H(37)	-0.545	-1.147	1.541
H(38)	1.065	-0.148	-1.055
H(39)	1.578	2.761	-0.689
H(40)	2.167	-1.523	1.542

Transition State E-TS3 (–586,371728 Hartree)

C(1)	2.994	-1.001	0.598
H(2)	3.717	-0.983	-0.234
H(3)	3.480	-1.491	1.449
C(4)	2.533	0.429	0.927
H(5)	2.063	0.464	1.926
H(6)	3.327	1.186	0.920
C(7)	1.423	0.693	-0.079
C(8)	0.795	1.950	-0.284
C(9)	1.162	3.168	0.518
H(10)	1.238	4.056	-0.122
H(11)	0.321	3.345	1.208
H(12)	2.071	3.057	1.115
C(13)	-0.413	2.031	-1.189
H(14)	-0.239	1.452	-2.105
H(15)	-0.592	3.072	-1.486
C(16)	-1.651	1.453	-0.449
H(17)	-2.461	1.410	-1.191
H(18)	-1.984	2.146	0.337
C(19)	-1.416	0.049	0.165
H(20)	-0.842	0.181	1.101
C(21)	-2.744	-0.593	0.563
C(22)	-3.070	-0.734	1.859
H(23)	-4.015	-1.183	2.164
H(24)	-2.402	-0.404	2.658
C(25)	-3.661	-1.057	-0.548
H(26)	-3.185	-1.827	-1.174
H(27)	-3.944	-0.236	-1.224
H(28)	-4.586	-1.484	-0.143
C(29)	1.903	-3.030	-0.570
H(30)	2.408	-3.754	0.083
H(31)	2.525	-2.893	-1.467
H(32)	0.950	-3.477	-0.883
C(33)	1.696	-1.701	0.158
C(34)	0.956	-0.630	-0.706
C(35)	-0.584	-0.850	-0.783
H(36)	-0.941	-0.733	-1.817
H(37)	-0.763	-1.902	-0.521
H(38)	1.387	-0.690	-1.723
H(39)	1.854	1.515	-0.999
H(40)	1.076	-1.874	1.057

Transition State E-TS5 (–586,3277742 Hartree)

C(1)	-3.053	-1.415	-0.274
H(2)	-3.642	-1.870	0.531
H(3)	-3.286	-1.935	-1.214
C(4)	-3.180	0.134	-0.318
H(5)	-3.485	0.550	-1.288
H(6)	-3.845	0.530	0.458
C(7)	-1.696	0.436	-0.038
C(8)	-1.214	1.739	0.568
C(9)	-1.015	2.708	-0.629
H(10)	-0.779	3.703	-0.230
H(11)	-0.194	2.396	-1.284
H(12)	-1.927	2.799	-1.233
C(13)	0.011	1.645	1.522
H(14)	-0.365	1.551	2.552
H(15)	0.530	2.613	1.476
C(16)	1.028	0.518	1.278
H(17)	0.624	-0.444	1.632
H(18)	1.886	0.719	1.935
C(19)	1.539	0.408	-0.178
H(20)	1.586	1.435	-0.573
C(21)	2.963	-0.149	-0.245
C(22)	3.926	0.521	-0.898
H(23)	4.945	0.139	-0.960
H(24)	3.729	1.476	-1.390
C(25)	3.237	-1.468	0.446
H(26)	2.617	-2.287	0.046
H(27)	3.029	-1.413	1.526
H(28)	4.284	-1.769	0.326
C(29)	-0.826	-2.591	0.647
H(30)	-1.341	-2.937	1.550
H(31)	0.226	-2.386	0.865
H(32)	-0.854	-3.406	-0.097
C(33)	-1.542	-1.446	-0.006
C(34)	-0.899	-0.461	-0.982
H(35)	-1.381	-0.520	-1.967
C(36)	0.634	-0.420	-1.139
H(37)	0.972	-1.467	-1.134
H(38)	0.841	-0.067	-2.158
H(39)	-1.590	-0.474	0.949
H(40)	-2.060	2.121	1.159

Transition State E-TS6 (–586,375837 Hartree)

C(1)	-3.120	-1.266	0.115
H(2)	-3.453	-1.287	-0.935
H(3)	-3.869	-1.799	0.712
C(4)	-1.709	-1.872	0.229
C(5)	-0.774	-0.723	-0.249
H(6)	-0.732	-0.744	-1.358
C(7)	-1.959	3.065	0.110
H(8)	-2.820	2.855	0.750
H(9)	-2.331	3.421	-0.864
H(10)	-1.371	3.884	0.544
C(11)	-1.083	1.866	-0.159
C(12)	-1.544	0.548	0.094
C(13)	-2.947	0.198	0.579
H(14)	-2.988	0.251	1.679
H(15)	-3.706	0.886	0.187
C(16)	0.240	2.128	-0.856
C(17)	1.516	1.549	-0.200
H(18)	1.515	1.804	0.872
H(19)	2.372	2.077	-0.641
C(20)	1.734	0.033	-0.398
H(21)	1.662	-0.168	-1.481
C(22)	3.138	-0.374	0.049
C(23)	3.496	-0.214	1.511
H(24)	3.409	0.833	1.843
H(25)	4.526	-0.536	1.701
H(26)	2.837	-0.809	2.163
C(27)	4.016	-0.860	-0.845
C(28)	0.663	-0.831	0.301
H(29)	0.641	-0.613	1.383
H(30)	0.965	-1.883	0.210
C(31)	-1.523	-3.180	-0.541
H(32)	-2.206	-3.952	-0.163
H(33)	-0.501	-3.569	-0.445
H(34)	-1.734	-3.039	-1.612
H(35)	-0.872	1.237	1.003
H(36)	-1.491	-2.048	1.299
H(37)	0.350	3.215	-0.953
H(38)	0.142	1.721	-1.878
H(39)	5.023	-1.157	-0.549
H(40)	3.761	-0.977	-1.900

Intermediate H (–586,3774102 Hartree)

C(1)	-3.088	-1.160	-0.546
H(2)	-4.025	-1.589	-0.162
H(3)	-2.964	-1.604	-1.561
C(4)	-2.968	0.376	-0.621
H(5)	-3.453	0.792	-1.511
H(6)	-3.449	0.822	0.262
C(7)	-1.444	0.625	-0.598
C(8)	-1.012	2.054	-0.193
C(9)	-1.246	2.360	1.295
H(10)	-2.291	2.207	1.600
H(11)	-0.609	1.748	1.954
H(12)	-0.998	3.407	1.509
C(13)	0.435	2.367	-0.635
H(14)	0.636	3.426	-0.412
H(15)	0.497	2.267	-1.732
C(16)	1.553	1.522	-0.002
H(17)	1.555	1.640	1.094
H(18)	2.510	1.939	-0.348
C(19)	1.538	0.016	-0.366
H(20)	1.216	-0.086	-1.415
C(21)	2.927	-0.613	-0.256
C(22)	3.447	-1.292	-1.296
H(23)	4.442	-1.736	-1.245
H(24)	2.907	-1.407	-2.238
C(25)	3.682	-0.449	1.044
H(26)	3.125	-0.861	1.901
H(27)	3.863	0.612	1.273
H(28)	4.653	-0.956	1.007
C(29)	-1.664	-2.993	0.643
H(30)	-1.031	-3.037	1.539
H(31)	-1.085	-3.506	-0.151
H(32)	-2.596	-3.551	0.779
C(33)	-1.881	-1.613	0.179
C(34)	-0.935	-0.515	0.342
H(35)	-1.319	-0.210	1.361
C(36)	0.575	-0.832	0.515
H(37)	0.858	-0.695	1.567
H(38)	0.756	-1.890	0.281
H(39)	-1.047	0.412	-1.607
H(40)	-1.665	2.730	-0.772

Intermediate H1 (–586,3865564 Hartree)

C(1)	-3.167	-1.090	-0.437
H(2)	-4.114	-1.442	-0.014
H(3)	-3.073	-1.509	-1.451
C(4)	-3.020	0.439	-0.456
H(5)	-3.524	0.922	-1.300
H(6)	-3.422	0.880	0.470
C(7)	-1.485	0.652	-0.505
C(8)	-0.939	2.075	-0.187
C(9)	-1.027	2.397	1.316
H(10)	-2.051	2.286	1.697
H(11)	-0.373	1.758	1.925
H(12)	-0.720	3.435	1.495
C(13)	0.457	2.342	-0.787
H(14)	0.676	3.412	-0.650
H(15)	0.411	2.178	-1.877
C(16)	1.632	1.539	-0.209
H(17)	1.742	1.746	0.867
H(18)	2.555	1.904	-0.682
C(19)	1.570	0.013	-0.438
H(20)	1.275	-0.177	-1.484
C(21)	2.904	-0.675	-0.191
C(22)	3.426	-1.498	-1.123
H(23)	4.383	-1.996	-0.968
H(24)	2.924	-1.681	-2.075
C(25)	3.591	-0.414	1.128
H(26)	2.924	-0.612	1.984
H(27)	3.903	0.637	1.216
H(28)	4.481	-1.042	1.248
C(29)	-1.581	-3.037	0.379
H(30)	-0.819	-3.298	1.123
H(31)	-1.213	-3.320	-0.616
H(32)	-2.474	-3.636	0.593
C(33)	-1.950	-1.549	0.412
C(34)	-0.900	-0.531	0.153
C(35)	0.512	-0.709	0.480
H(36)	0.631	-0.287	1.501
H(37)	0.751	-1.778	0.581
H(38)	-1.171	0.414	-1.559
H(39)	-1.638	2.749	-0.709
H(40)	-2.200	-1.270	1.471

Intermediate H2 (–586,3836923 Hartree)

C(1)	-3.333	-0.904	-0.009
H(2)	-4.184	-1.221	0.604
H(3)	-3.573	-1.126	-1.061
C(4)	-3.021	0.601	0.146
H(5)	-3.664	1.324	-0.376
H(6)	-3.083	0.870	1.225
C(7)	-1.577	0.739	-0.153
C(8)	-0.922	2.031	-0.399
C(9)	-0.750	2.680	1.036
H(10)	-1.720	2.930	1.476
H(11)	-0.199	2.027	1.721
H(12)	-0.172	3.602	0.896
C(13)	0.400	2.013	-1.203
H(14)	0.619	3.068	-1.428
H(15)	0.211	1.528	-2.175
C(16)	1.638	1.388	-0.538
H(17)	1.778	1.815	0.467
H(18)	2.517	1.697	-1.120
C(19)	1.641	-0.153	-0.462
H(20)	1.484	-0.539	-1.486
C(21)	2.985	-0.695	0.028
C(22)	3.653	-1.605	-0.702
H(23)	4.605	-2.016	-0.365
H(24)	3.276	-1.964	-1.662
C(25)	3.518	-0.186	1.350
H(26)	2.789	-0.306	2.166
H(27)	3.764	0.886	1.301
H(28)	4.430	-0.721	1.639
C(29)	-1.841	-3.011	-0.132
H(30)	-0.880	-3.448	0.168
H(31)	-1.903	-3.040	-1.230
H(32)	-2.634	-3.657	0.266
C(33)	-2.004	-1.582	0.387
C(34)	-0.932	-0.581	-0.145
C(35)	0.506	-0.727	0.414
H(36)	0.532	-0.296	1.426
H(37)	0.675	-1.806	0.533
H(38)	-0.868	-0.740	-1.259
H(39)	-1.650	2.680	-0.916
H(40)	-1.920	-1.583	1.490

Intermediate H3 (–586,3815038 Hartree)

C(1)	3.316	0.837	-0.204
H(2)	4.191	1.124	0.392
H(3)	3.482	1.199	-1.232
C(4)	3.071	-0.679	-0.199
H(5)	3.752	-1.241	-0.851
H(6)	3.201	-1.069	0.820
C(7)	1.613	-0.851	-0.681
C(8)	0.737	-1.813	-0.047
C(9)	0.957	-2.261	1.349
H(10)	1.773	-3.008	1.321
H(11)	1.329	-1.452	1.992
H(12)	0.078	-2.740	1.791
C(13)	-0.467	-2.280	-0.753
H(14)	-0.646	-3.341	-0.520
H(15)	-0.384	-2.145	-1.839
C(16)	-1.729	-1.487	-0.226
H(17)	-1.875	-1.704	0.842
H(18)	-2.586	-1.912	-0.765
C(19)	-1.662	0.032	-0.462
H(20)	-1.513	0.198	-1.543
C(21)	-3.009	0.648	-0.077
C(22)	-3.845	1.093	-1.032
H(23)	-4.810	1.532	-0.781
H(24)	-3.589	1.039	-2.092
C(25)	-3.361	0.722	1.393
H(26)	-2.637	1.332	1.956
H(27)	-3.372	-0.271	1.867
H(28)	-4.352	1.167	1.538
C(29)	1.861	2.935	0.167
H(30)	0.930	3.319	0.602
H(31)	1.887	3.212	-0.896
H(32)	2.695	3.446	0.668
C(33)	2.004	1.415	0.343
C(34)	0.885	0.639	-0.395
C(35)	-0.485	0.708	0.291
H(36)	-0.425	0.337	1.327
H(37)	-0.714	1.781	0.372
H(38)	0.772	1.035	-1.416
H(39)	1.556	-0.966	-1.772
H(40)	1.934	1.172	1.420

Intermediate H4 (–586,3838988 Hartree)

C(1)	-3.047	-1.060	-0.542
H(2)	-3.763	-1.053	0.295
H(3)	-3.502	-1.621	-1.366
C(4)	-2.694	0.389	-0.931
H(5)	-2.280	0.417	-1.966
H(6)	-3.500	1.138	-0.932
C(7)	-1.539	0.769	-0.089
C(8)	-1.000	2.134	-0.032
C(9)	-0.658	2.649	1.384
H(10)	-1.568	2.726	1.992
H(11)	0.058	2.012	1.911
H(12)	-0.220	3.651	1.296
C(13)	0.267	2.146	-1.000
H(14)	0.481	3.217	-1.130
H(15)	-0.037	1.765	-1.988
C(16)	1.521	1.419	-0.511
H(17)	1.863	1.857	0.439
H(18)	2.314	1.633	-1.244
C(19)	1.399	-0.117	-0.367
H(20)	0.918	-0.508	-1.283
C(21)	2.772	-0.776	-0.256
C(22)	3.122	-1.761	-1.103
H(23)	4.095	-2.248	-1.037
H(24)	2.455	-2.103	-1.896
C(25)	3.700	-0.296	0.839
H(26)	3.231	-0.363	1.833
H(27)	3.985	0.758	0.696
H(28)	4.621	-0.889	0.865
C(29)	-1.813	-2.874	0.821
H(30)	-2.386	-2.644	1.731
H(31)	-0.829	-3.255	1.123
H(32)	-2.331	-3.686	0.295
C(33)	-1.697	-1.644	-0.080
C(34)	-0.997	-0.418	0.597
H(35)	-1.495	-0.294	1.597
C(36)	0.525	-0.536	0.841
H(37)	0.830	0.024	1.735
H(38)	0.711	-1.594	1.071
H(39)	-1.094	-1.903	-0.970
H(40)	-1.731	2.816	-0.493

Intermediate H5 (–586,3767655 Hartree)

C(1)	-3.347	-0.574	-0.199
H(2)	-3.855	-0.837	0.740
H(3)	-4.079	-0.675	-1.011
C(4)	-2.834	0.898	-0.103
H(5)	-3.305	1.589	-0.810
H(6)	-2.968	1.303	0.909
C(7)	-1.274	0.744	-0.425
C(8)	-0.543	1.881	0.123
C(9)	-0.508	3.177	-0.587
H(10)	-1.022	3.169	-1.553
H(11)	-0.944	3.954	0.066
H(12)	0.542	3.491	-0.718
C(13)	0.198	1.716	1.378
C(14)	1.558	0.918	1.067
H(15)	1.817	0.374	1.985
H(16)	2.327	1.684	0.903
C(17)	1.554	-0.018	-0.164
H(18)	1.346	0.620	-1.044
C(19)	2.976	-0.558	-0.340
C(20)	3.833	0.075	-1.163
H(21)	4.861	-0.266	-1.287
H(22)	3.531	0.945	-1.749
C(23)	3.386	-1.779	0.454
H(24)	2.892	-2.687	0.076
H(25)	3.118	-1.693	1.518
H(26)	4.468	-1.942	0.392
C(27)	-2.245	-2.884	0.206
H(28)	-2.387	-2.815	1.294
H(29)	-1.352	-3.492	0.014
H(30)	-3.107	-3.421	-0.211
C(31)	-2.115	-1.492	-0.418
C(32)	-0.946	-0.647	0.140
H(33)	-1.091	-0.612	1.236
C(34)	0.482	-1.135	-0.152
H(35)	0.744	-1.898	0.594
H(36)	0.513	-1.634	-1.134
H(37)	-1.949	-1.607	-1.505
H(38)	-0.385	1.095	2.072
H(39)	0.448	2.670	1.854
H(40)	-1.204	0.765	-1.528

Transition State H-TS1 (–586,3758569 Hartree)

C(1)	-3.172	-1.056	-0.529
H(2)	-4.094	-1.392	-0.038
H(3)	-3.161	-1.510	-1.538
C(4)	-2.977	0.473	-0.601
H(5)	-3.464	0.924	-1.472
H(6)	-3.408	0.940	0.297
C(7)	-1.441	0.679	-0.616
C(8)	-0.946	2.059	-0.116
C(9)	-1.106	2.236	1.404
H(10)	-2.146	2.106	1.732
H(11)	-0.483	1.533	1.978
H(12)	-0.799	3.246	1.703
C(13)	0.481	2.380	-0.615
H(14)	0.697	3.430	-0.365
H(15)	0.487	2.319	-1.716
C(16)	1.627	1.517	-0.061
H(17)	1.705	1.642	1.030
H(18)	2.566	1.909	-0.477
C(19)	1.564	0.007	-0.402
H(20)	1.260	-0.105	-1.456
C(21)	2.927	-0.666	-0.245
C(22)	3.483	-1.319	-1.281
H(23)	4.459	-1.797	-1.192
H(24)	2.992	-1.383	-2.253
C(25)	3.613	-0.562	1.099
H(26)	2.988	-0.961	1.915
H(27)	3.836	0.483	1.359
H(28)	4.557	-1.118	1.106
C(29)	-1.758	-2.965	0.624
H(30)	-0.877	-3.110	1.257
H(31)	-1.642	-3.603	-0.268
H(32)	-2.657	-3.309	1.150
C(33)	-1.917	-1.559	0.142
C(34)	-0.906	-0.552	0.109
H(35)	-1.658	-0.655	1.156
C(36)	0.541	-0.772	0.485
H(37)	0.701	-0.469	1.531
H(38)	0.768	-1.846	0.432
H(39)	-1.084	0.538	-1.656
H(40)	-1.614	2.791	-0.602

Transition State H-TS2 (–586,3785486 Hartree)

C(1)	-3.228	-1.098	-0.243
H(2)	-4.147	-1.450	0.238
H(3)	-3.246	-1.449	-1.288
C(4)	-3.078	0.436	-0.214
H(5)	-3.573	0.970	-1.034
H(6)	-3.467	0.860	0.729
C(7)	-1.583	0.673	-0.169
C(8)	-0.988	2.053	-0.303
C(9)	-0.923	2.668	1.123
H(10)	-1.917	2.726	1.584
H(11)	-0.268	2.094	1.789
H(12)	-0.525	3.687	1.049
C(13)	0.360	2.133	-1.060
H(14)	0.572	3.205	-1.187
H(15)	0.216	1.730	-2.077
C(16)	1.584	1.468	-0.413
H(17)	1.722	1.840	0.613
H(18)	2.472	1.794	-0.974
C(19)	1.577	-0.076	-0.415
H(20)	1.309	-0.416	-1.433
C(21)	2.957	-0.647	-0.091
C(22)	3.580	-1.448	-0.971
H(23)	4.567	-1.860	-0.763
H(24)	3.133	-1.709	-1.932
C(25)	3.578	-0.273	1.236
H(26)	2.933	-0.547	2.087
H(27)	3.755	0.810	1.311
H(28)	4.541	-0.778	1.377
C(29)	-1.543	-3.049	0.025
H(30)	-0.685	-3.427	0.595
H(31)	-1.291	-3.082	-1.044
H(32)	-2.379	-3.738	0.195
C(33)	-1.950	-1.634	0.449
C(34)	-0.919	-0.529	0.209
C(35)	0.532	-0.692	0.560
H(36)	0.658	-0.245	1.562
H(37)	0.745	-1.763	0.666
H(38)	-1.056	-0.207	-1.064
H(39)	-1.715	2.645	-0.881
H(40)	-2.099	-1.611	1.548

Transition State H-TS3 (–586,375523 Hartree)

C(1)	-3.167	-1.012	0.416
H(2)	-3.827	-1.502	1.142
H(3)	-3.727	-0.916	-0.529
C(4)	-2.701	0.379	0.887
H(5)	-3.461	1.168	0.837
H(6)	-2.344	0.333	1.932
C(7)	-1.479	0.628	0.026
C(8)	-0.927	1.892	-0.314
C(9)	-1.467	3.181	0.246
H(10)	-2.408	3.076	0.792
H(11)	-0.704	3.553	0.949
H(12)	-1.569	3.941	-0.538
C(13)	0.345	1.972	-1.137
H(14)	0.504	3.020	-1.421
H(15)	0.233	1.401	-2.072
C(16)	1.574	1.427	-0.363
H(17)	1.538	1.776	0.681
H(18)	2.470	1.878	-0.811
C(19)	1.723	-0.111	-0.417
H(20)	1.702	-0.399	-1.484
C(21)	3.076	-0.544	0.146
C(22)	3.979	-1.140	-0.652
H(23)	4.951	-1.456	-0.273
H(24)	3.780	-1.331	-1.708
C(25)	3.358	-0.282	1.610
H(26)	2.642	-0.802	2.266
H(27)	3.294	0.789	1.857
H(28)	4.363	-0.624	1.884
C(29)	-1.972	-3.002	-0.729
H(30)	-1.000	-3.494	-0.871
H(31)	-2.357	-2.717	-1.719
H(32)	-2.660	-3.744	-0.302
C(33)	-1.850	-1.781	0.186
C(34)	-0.839	-0.709	-0.340
C(35)	0.571	-0.864	0.283
H(36)	0.520	-0.579	1.347
H(37)	0.806	-1.939	0.264
H(38)	-0.750	-0.792	-1.439
H(39)	-1.870	1.273	-1.044
H(40)	-1.479	-2.112	1.172

Transition State H-TS5 (–586,3386579 Hartree)

C(1)	-2.979	-1.577	0.192
H(2)	-3.214	-2.107	1.126
H(3)	-3.522	-2.063	-0.628
C(4)	-3.186	-0.040	0.229
H(5)	-3.853	0.322	-0.563
H(6)	-3.538	0.357	1.191
C(7)	-1.717	0.349	-0.008
C(8)	-1.317	1.691	-0.588
C(9)	-1.350	2.684	0.606
H(10)	-2.334	2.711	1.090
H(11)	-0.596	2.439	1.364
H(12)	-1.136	3.690	0.223
C(13)	-0.001	1.755	-1.392
H(14)	0.051	2.771	-1.809
H(15)	-0.088	1.075	-2.257
C(16)	1.304	1.454	-0.644
H(17)	1.418	2.124	0.222
H(18)	2.134	1.692	-1.325
C(19)	1.453	-0.021	-0.207
H(20)	1.096	-0.633	-1.054
C(21)	2.917	-0.397	0.019
C(22)	3.504	-1.330	-0.749
H(23)	4.548	-1.609	-0.608
H(24)	2.969	-1.835	-1.556
C(25)	3.664	0.324	1.119
H(26)	3.204	0.158	2.106
H(27)	3.671	1.412	0.954
H(28)	4.705	-0.013	1.178
C(29)	-0.695	-2.690	-0.623
H(30)	-0.790	-3.498	0.124
H(31)	0.372	-2.494	-0.754
H(32)	-1.140	-3.046	-1.559
C(33)	-1.463	-1.546	-0.030
C(34)	-0.891	-0.515	0.941
H(35)	-1.355	-0.591	1.934
C(36)	0.627	-0.365	1.061
H(37)	0.823	0.398	1.829
H(38)	1.002	-1.312	1.480
H(39)	-1.522	-0.567	-0.994
H(40)	-2.129	1.969	-1.280

Transition State H-TS6 (–586,370443 Hartree)

C(1)	2.990	-1.255	0.440
H(2)	3.597	-1.395	-0.468
H(3)	3.510	-1.756	1.265
C(4)	2.789	0.253	0.707
H(5)	2.686	0.452	1.785
H(6)	3.612	0.886	0.347
C(7)	1.487	0.590	-0.014
C(8)	0.967	1.897	-0.187
C(9)	1.679	3.111	0.349
H(10)	2.446	2.884	1.095
H(11)	2.169	3.602	-0.507
H(12)	0.962	3.831	0.762
C(13)	-0.284	2.102	-1.020
C(14)	-1.578	1.576	-0.344
H(15)	-2.370	1.630	-1.102
H(16)	-1.879	2.251	0.470
C(17)	-1.480	0.129	0.209
H(18)	-0.964	0.161	1.190
C(19)	-2.876	-0.420	0.505
C(20)	-3.304	-0.541	1.773
H(21)	-4.302	-0.915	2.003
H(22)	-2.675	-0.269	2.623
C(23)	-3.742	-0.808	-0.675
H(24)	-3.297	-1.635	-1.250
H(25)	-3.881	0.025	-1.381
H(26)	-4.735	-1.131	-0.343
C(27)	1.549	-3.172	-0.494
H(28)	2.045	-3.121	-1.474
H(29)	0.526	-3.539	-0.650
H(30)	2.076	-3.920	0.112
C(31)	1.572	-1.808	0.198
C(32)	0.883	-0.673	-0.614
H(33)	1.318	-0.698	-1.636
C(34)	-0.667	-0.792	-0.730
H(35)	-0.983	-0.631	-1.769
H(36)	-0.918	-1.835	-0.498
H(37)	1.046	-1.884	1.169
H(38)	0.632	1.112	0.843
H(39)	-0.137	1.592	-1.984
H(40)	-0.394	3.173	-1.228

Computational Results for Guaienol

Intermediate A (–662,8435522 Hartree)

C(1)	-3.443	-0.922	-0.414
H(2)	-3.687	-0.383	-1.342
H(3)	-4.273	-1.609	-0.209
C(4)	-2.097	-1.654	-0.571
C(5)	-1.032	-0.510	-0.501
H(6)	-1.053	0.008	-1.477
C(7)	-2.272	2.489	-0.813
H(8)	-3.061	1.873	-1.255
H(9)	-1.753	3.072	-1.582
H(10)	-2.780	3.217	-0.151
C(11)	-1.337	1.730	0.059
C(12)	-1.714	0.474	0.656
H(13)	-1.141	0.266	1.568
C(14)	-3.205	0.072	0.741
H(15)	-3.368	-0.415	1.712
H(16)	-3.880	0.937	0.711
C(17)	0.012	2.305	0.303
H(18)	-0.185	3.059	1.099
H(19)	0.319	2.906	-0.567
C(20)	1.140	1.362	0.754
H(21)	0.922	0.944	1.749
H(22)	2.043	1.974	0.860
C(23)	1.442	0.233	-0.254
H(24)	1.386	0.668	-1.272
C(25)	2.925	-0.266	-0.095
C(26)	3.215	-0.836	1.302
H(27)	3.050	-0.084	2.084
H(28)	4.266	-1.145	1.355
H(29)	2.590	-1.713	1.515
C(30)	3.292	-1.295	-1.180
H(31)	3.041	-0.924	-2.186
H(32)	4.372	-1.486	-1.140
H(33)	2.782	-2.256	-1.035
O(34)	3.785	0.885	-0.216
H(35)	3.872	1.092	-1.160
C(36)	0.407	-0.909	-0.179
H(37)	0.442	-1.370	0.819
H(38)	0.673	-1.697	-0.898
C(39)	-1.887	-2.741	0.493
H(40)	-2.703	-3.473	0.431
H(41)	-1.885	-2.337	1.515
H(42)	-0.945	-3.282	0.345
H(43)	-2.011	-2.110	-1.570

Intermediate A1 (–662,8425705 Hartree)

C(1)	-3.236	-1.413	-0.843
H(2)	-3.219	-1.402	-1.943
H(3)	-4.116	-1.984	-0.527
C(4)	-1.907	-1.980	-0.287
C(5)	-0.969	-0.724	-0.358
H(6)	-0.850	-0.535	-1.464
C(7)	-2.500	2.822	0.427
H(8)	-2.828	3.113	-0.580
H(9)	-2.104	3.718	0.919
H(10)	-3.379	2.493	0.996
C(11)	-1.405	1.747	0.361
C(12)	-1.847	0.423	-0.090
C(13)	-3.258	0.038	-0.316
H(14)	-3.717	0.093	0.696
H(15)	-3.820	0.785	-0.898
C(16)	-0.099	2.244	-0.372
H(17)	-0.066	3.324	-0.177
H(18)	-0.240	2.128	-1.458
C(19)	1.221	1.617	0.089
H(20)	1.281	1.691	1.187
H(21)	2.046	2.225	-0.303
C(22)	1.473	0.163	-0.345
H(23)	1.366	0.121	-1.448
C(24)	2.959	-0.269	-0.054
C(25)	3.287	-0.295	1.446
H(26)	3.171	0.698	1.898
H(27)	4.330	-0.603	1.585
H(28)	2.647	-1.007	1.983
C(29)	3.286	-1.631	-0.696
H(30)	3.007	-1.647	-1.762
H(31)	4.364	-1.812	-0.618
H(32)	2.776	-2.467	-0.200
O(33)	3.829	0.735	-0.612
H(34)	3.868	0.594	-1.572
C(35)	0.439	-0.816	0.252
H(36)	0.367	-0.672	1.341
H(37)	0.769	-1.851	0.098
C(38)	-2.030	-2.527	1.143
H(39)	-2.722	-3.379	1.161
H(40)	-2.413	-1.772	1.847
H(41)	-1.064	-2.876	1.527
H(42)	-1.504	-2.766	-0.940
H(43)	-1.061	1.510	1.399

Intermediate A2 (–662,8417198 Hartree)

C(1)	-3.040	-1.497	-0.945
H(2)	-2.891	-1.498	-2.035
H(3)	-3.903	-2.136	-0.724
C(4)	-1.752	-1.987	-0.241
C(5)	-0.947	-0.761	-0.014
H(6)	-1.398	0.583	-1.396
C(7)	-2.535	2.798	0.193
H(8)	-2.650	3.045	-0.873
H(9)	-2.304	3.727	0.728
H(10)	-3.500	2.433	0.565
C(11)	-1.401	1.780	0.406
C(12)	-1.728	0.448	-0.328
C(13)	-3.200	-0.054	-0.431
H(14)	-3.668	-0.030	0.564
H(15)	-3.800	0.577	-1.093
C(16)	-0.055	2.387	-0.053
H(17)	0.007	3.403	0.363
H(18)	-0.087	2.514	-1.149
C(19)	1.232	1.648	0.345
H(20)	1.301	1.607	1.444
H(21)	2.090	2.239	0.000
C(22)	1.397	0.226	-0.224
H(23)	1.139	0.240	-1.299
C(24)	2.887	-0.294	-0.149
C(25)	3.402	-0.383	1.294
H(26)	3.395	0.600	1.777
H(27)	4.436	-0.749	1.289
H(28)	2.800	-1.079	1.894
C(29)	3.061	-1.635	-0.885
H(30)	2.648	-1.586	-1.904
H(31)	4.131	-1.866	-0.955
H(32)	2.582	-2.474	-0.365
O(33)	3.694	0.706	-0.787
H(34)	3.629	0.583	-1.749
C(35)	0.423	-0.784	0.489
H(36)	0.355	-0.446	1.544
H(37)	0.804	-1.811	0.510
C(38)	-2.042	-2.579	1.191
H(39)	-2.697	-3.447	1.053
H(40)	-2.559	-1.853	1.830
H(41)	-1.122	-2.902	1.690
H(42)	-1.174	-2.752	-0.787
H(43)	-1.339	1.551	1.487

Intermediate A3 (–662,8338887 Hartree)

C(1)	2.790	1.758	0.678
H(2)	3.681	2.064	0.084
H(3)	2.782	2.456	1.531
C(4)	1.659	1.949	-0.256
C(5)	1.057	0.674	-0.619
H(6)	2.792	-0.493	-1.050
C(7)	2.681	-2.854	-0.024
H(8)	3.015	-2.946	-1.068
H(9)	2.346	-3.845	0.309
H(10)	3.552	-2.577	0.585
C(11)	1.541	-1.826	0.098
C(12)	2.086	-0.412	-0.203
C(13)	2.827	0.247	0.992
H(14)	2.288	0.049	1.930
H(15)	3.848	-0.125	1.119
C(16)	0.329	-2.217	-0.781
H(17)	0.288	-3.314	-0.832
H(18)	0.485	-1.880	-1.821
C(19)	-1.033	-1.740	-0.241
H(20)	-1.092	-2.020	0.823
H(21)	-1.836	-2.293	-0.742
C(22)	-1.359	-0.243	-0.387
H(23)	-1.387	0.003	-1.465
C(24)	-2.810	0.113	0.158
C(25)	-2.949	-0.090	1.673
H(26)	-2.749	-1.130	1.955
H(27)	-3.974	0.151	1.979
H(28)	-2.263	0.566	2.225
C(29)	-3.226	1.544	-0.240
H(30)	-3.075	1.719	-1.317
H(31)	-4.290	1.681	-0.013
H(32)	-2.675	2.316	0.314
O(33)	-3.718	-0.823	-0.438
H(34)	-3.871	-0.556	-1.360
C(35)	-0.299	0.677	0.266
H(36)	-0.046	0.347	1.281
H(37)	-0.690	1.698	0.342
C(38)	1.237	3.263	-0.777
H(39)	1.998	3.569	-1.521
H(40)	1.252	4.043	-0.004
H(41)	0.270	3.232	-1.291
H(42)	0.721	0.646	-1.665
H(43)	1.197	-1.834	1.148

Intermediate A4 (–662,8390381 Hartree)

C(1)	3.236	0.916	-0.605
H(2)	3.324	0.293	-1.507
H(3)	4.068	1.629	-0.612
C(4)	1.859	1.627	-0.580
C(5)	0.963	0.717	0.187
C(6)	2.510	-2.565	-0.529
H(7)	3.379	-2.111	-1.017
H(8)	2.164	-3.385	-1.174
H(9)	2.825	-2.997	0.429
C(10)	1.341	-1.584	-0.341
C(11)	1.720	-0.421	0.732
H(12)	1.381	-0.778	1.713
C(13)	3.196	0.041	0.663
H(14)	3.422	0.642	1.556
H(15)	3.905	-0.793	0.651
C(16)	0.066	-2.331	0.095
H(17)	0.343	-3.064	0.869
H(18)	-0.270	-2.911	-0.778
C(19)	-1.113	-1.490	0.621
H(20)	-0.956	-1.235	1.681
H(21)	-2.013	-2.114	0.582
C(22)	-1.388	-0.198	-0.172
H(23)	-1.154	-0.362	-1.239
C(24)	-2.911	0.227	-0.131
C(25)	-3.424	0.449	1.298
H(26)	-3.309	-0.453	1.910
H(27)	-4.491	0.697	1.262
H(28)	-2.903	1.282	1.788
C(29)	-3.193	1.455	-1.014
H(30)	-2.764	1.333	-2.021
H(31)	-4.278	1.581	-1.113
H(32)	-2.796	2.383	-0.583
O(33)	-3.636	-0.905	-0.633
H(34)	-3.592	-0.890	-1.603
C(35)	-0.474	0.977	0.342
H(36)	-0.680	1.066	1.424
H(37)	-0.742	1.921	-0.144
C(38)	1.913	2.982	0.216
H(39)	2.608	3.642	-0.315
H(40)	2.288	2.832	1.236
H(41)	0.933	3.471	0.267
H(42)	1.432	1.861	-1.570
H(43)	1.152	-1.109	-1.320

Intermediate A5 (–662,8351146 Hartree)

C(1)	3.317	1.374	0.293
H(2)	4.132	1.986	-0.122
H(3)	3.383	1.525	1.398
C(4)	1.971	1.873	-0.035
C(5)	1.047	0.755	-0.256
C(6)	2.397	-2.933	-0.366
H(7)	3.288	-2.673	-0.951
H(8)	1.992	-3.866	-0.780
H(9)	2.712	-3.143	0.667
C(10)	1.322	-1.832	-0.400
C(11)	1.810	-0.519	0.256
H(12)	1.667	-0.578	1.351
C(13)	3.280	-0.135	-0.022
H(14)	3.999	-0.705	0.574
H(15)	3.525	-0.288	-1.085
C(16)	0.017	-2.381	0.234
H(17)	0.292	-3.048	1.067
H(18)	-0.468	-3.025	-0.516
C(19)	-1.010	-1.369	0.771
H(20)	-0.663	-0.959	1.733
H(21)	-1.931	-1.924	0.988
C(22)	-1.359	-0.199	-0.178
H(23)	-1.205	-0.528	-1.224
C(24)	-2.884	0.195	-0.085
C(25)	-3.286	0.678	1.315
H(26)	-3.052	-0.073	2.079
H(27)	-4.367	0.860	1.340
H(28)	-2.777	1.616	1.573
C(29)	-3.270	1.239	-1.152
H(30)	-2.948	0.921	-2.156
H(31)	-4.361	1.356	-1.161
H(32)	-2.835	2.227	-0.951
O(33)	-3.652	-1.000	-0.303
H(34)	-3.580	-1.235	-1.243
C(35)	-0.442	1.011	0.077
H(36)	-0.506	1.297	1.140
H(37)	-0.778	1.879	-0.506
C(38)	1.602	3.294	-0.081
H(39)	2.462	3.969	-0.116
H(40)	1.029	3.505	0.845
H(41)	0.892	3.505	-0.894
H(42)	1.119	0.688	-1.379
H(43)	1.123	-1.593	-1.463

Transition State A-TS1 (–662,8319706 Hartree)

C(1)	-3.293	-1.412	-0.686
H(2)	-3.408	-1.312	-1.776
H(3)	-4.112	-2.041	-0.319
C(4)	-1.898	-1.972	-0.343
C(5)	-0.975	-0.726	-0.540
H(6)	-0.869	-0.563	-1.633
C(7)	-2.476	2.863	0.364
H(8)	-2.680	3.411	-0.570
H(9)	-2.066	3.590	1.075
H(10)	-3.424	2.475	0.745
C(11)	-1.453	1.798	0.042
C(12)	-1.854	0.443	-0.105
H(13)	-1.419	0.926	1.057
C(14)	-3.312	-0.009	-0.037
H(15)	-3.646	-0.069	1.010
H(16)	-3.974	0.704	-0.544
C(17)	-0.060	2.297	-0.294
H(18)	-0.013	3.352	0.005
H(19)	-0.007	2.281	-1.398
C(20)	1.158	1.554	0.285
H(21)	1.027	1.452	1.375
H(22)	2.033	2.198	0.134
C(23)	1.468	0.187	-0.346
H(24)	1.418	0.307	-1.447
C(25)	2.951	-0.243	-0.038
C(26)	3.213	-0.442	1.462
H(27)	3.041	0.483	2.024
H(28)	4.259	-0.732	1.612
H(29)	2.575	-1.234	1.876
C(30)	3.344	-1.508	-0.824
H(31)	3.111	-1.403	-1.895
H(32)	4.423	-1.673	-0.718
H(33)	2.836	-2.409	-0.454
O(34)	3.815	0.843	-0.430
H(35)	3.909	0.813	-1.396
C(36)	0.435	-0.876	0.060
H(37)	0.356	-0.908	1.158
H(38)	0.782	-1.870	-0.249
C(39)	-1.842	-2.558	1.079
H(40)	-2.582	-3.361	1.183
H(41)	-2.064	-1.806	1.853
H(42)	-0.858	-2.984	1.308
H(43)	-1.600	-2.752	-1.058

Transition State A-TS2 (–662,8350866 Hartree)

C(1)	-3.121	-1.414	-0.919
H(2)	-3.020	-1.355	-2.014
H(3)	-3.996	-2.036	-0.700
C(4)	-1.819	-1.979	-0.301
C(5)	-0.984	-0.735	-0.023
H(6)	-1.119	0.045	-1.102
C(7)	-2.535	2.847	0.210
H(8)	-2.723	3.021	-0.858
H(9)	-2.235	3.804	0.653
H(10)	-3.478	2.547	0.684
C(11)	-1.413	1.815	0.423
C(12)	-1.814	0.423	-0.026
C(13)	-3.242	0.012	-0.338
H(14)	-3.774	0.017	0.630
H(15)	-3.765	0.723	-0.988
C(16)	-0.081	2.313	-0.202
H(17)	-0.002	3.377	0.058
H(18)	-0.173	2.279	-1.302
C(19)	1.209	1.617	0.251
H(20)	1.249	1.621	1.353
H(21)	2.063	2.215	-0.092
C(22)	1.427	0.183	-0.265
H(23)	1.223	0.172	-1.355
C(24)	2.931	-0.267	-0.116
C(25)	3.389	-0.319	1.349
H(26)	3.310	0.666	1.824
H(27)	4.440	-0.628	1.390
H(28)	2.799	-1.042	1.928
C(29)	3.193	-1.617	-0.812
H(30)	2.817	-1.613	-1.848
H(31)	4.274	-1.799	-0.838
H(32)	2.733	-2.464	-0.287
O(33)	3.746	0.746	-0.732
H(34)	3.703	0.623	-1.694
C(35)	0.456	-0.829	0.394
H(36)	0.474	-0.681	1.488
H(37)	0.781	-1.859	0.207
C(38)	-2.044	-2.707	1.048
H(39)	-2.674	-3.589	0.877
H(40)	-2.555	-2.060	1.775
H(41)	-1.100	-3.046	1.493
H(42)	-1.288	-2.660	-0.982
H(43)	-1.240	1.703	1.512

Transition State A-TS3 (–662,8279646 Hartree)

C(1)	3.242	1.529	-0.372
H(2)	3.456	1.883	-1.394
H(3)	3.950	2.061	0.281
C(4)	1.831	1.902	0.025
C(5)	0.993	0.745	0.013
H(6)	1.617	-0.691	-1.465
C(7)	2.566	-2.851	0.145
H(8)	2.684	-3.087	-0.923
H(9)	2.307	-3.782	0.665
H(10)	3.538	-2.522	0.530
C(11)	1.450	-1.807	0.357
C(12)	1.815	-0.495	-0.394
C(13)	3.284	-0.009	-0.223
H(14)	3.633	-0.261	0.790
H(15)	3.967	-0.483	-0.934
C(16)	0.105	-2.405	-0.122
H(17)	0.055	-3.438	0.253
H(18)	0.140	-2.490	-1.222
C(19)	-1.192	-1.702	0.300
H(20)	-1.261	-1.703	1.400
H(21)	-2.042	-2.292	-0.065
C(22)	-1.371	-0.260	-0.210
H(23)	-1.109	-0.235	-1.287
C(24)	-2.868	0.218	-0.128
C(25)	-3.385	0.278	1.317
H(26)	-3.351	-0.712	1.786
H(27)	-4.428	0.615	1.318
H(28)	-2.799	0.979	1.926
C(29)	-3.065	1.575	-0.835
H(30)	-2.677	1.546	-1.865
H(31)	-4.136	1.805	-0.878
H(32)	-2.575	2.408	-0.312
O(33)	-3.689	-0.766	-0.778
H(34)	-3.547	-0.692	-1.736
C(35)	-0.416	0.706	0.536
H(36)	-0.322	0.383	1.587
H(37)	-0.815	1.727	0.580
C(38)	1.455	3.289	0.435
H(39)	1.972	4.036	-0.179
H(40)	1.809	3.420	1.471
H(41)	0.377	3.476	0.425
H(42)	1.009	1.486	-1.022
H(43)	1.386	-1.583	1.438

Transition State A-TS4 (–662,7940837 Hartree)

C(1)	-2.200	-1.761	1.201
H(2)	-3.170	-2.074	1.604
H(3)	-1.495	-2.597	1.310
C(4)	-2.282	-1.310	-0.262
C(5)	-1.006	-0.539	-0.588
H(6)	-1.083	-0.088	-1.587
C(7)	-2.678	2.392	-0.708
H(8)	-2.548	1.939	-1.701
H(9)	-2.560	3.476	-0.820
H(10)	-3.701	2.195	-0.360
C(11)	-1.628	1.858	0.288
C(12)	-1.642	0.359	0.449
C(13)	-1.713	-0.405	1.778
H(14)	-0.735	-0.422	2.276
H(15)	-2.437	0.045	2.469
C(16)	-0.180	2.256	-0.148
H(17)	-0.058	3.305	0.160
H(18)	-0.138	2.252	-1.249
C(19)	0.996	1.415	0.409
H(20)	0.806	1.141	1.459
H(21)	1.886	2.057	0.427
C(22)	1.402	0.165	-0.421
H(23)	1.409	0.478	-1.483
C(24)	2.883	-0.262	-0.106
C(25)	3.104	-0.612	1.374
H(26)	2.891	0.247	2.021
H(27)	4.152	-0.894	1.528
H(28)	2.477	-1.458	1.686
C(29)	3.336	-1.430	-1.003
H(30)	3.137	-1.219	-2.066
H(31)	4.415	-1.581	-0.878
H(32)	2.836	-2.373	-0.744
O(33)	3.734	0.877	-0.343
H(34)	3.818	0.988	-1.304
C(35)	0.411	-1.024	-0.315
H(36)	0.484	-1.486	0.679
H(37)	0.670	-1.804	-1.043
C(38)	-3.161	-1.926	-1.314
H(39)	-4.162	-2.158	-0.935
H(40)	-2.669	-2.870	-1.606
H(41)	-3.232	-1.303	-2.213
H(42)	-2.875	-0.135	0.032
H(43)	-1.852	2.276	1.284

Transition State A-TS5 (–662,8280636 Hartree)

C(1)	3.474	0.822	-0.134
H(2)	3.969	0.227	-0.913
H(3)	4.169	1.632	0.119
C(4)	2.125	1.393	-0.647
C(5)	1.037	0.436	-0.120
H(6)	0.982	-0.548	-1.054
C(7)	2.427	-2.181	-0.956
H(8)	3.258	-1.546	-1.264
H(9)	2.015	-2.683	-1.839
H(10)	2.825	-2.951	-0.278
C(11)	1.329	-1.455	-0.191
C(12)	1.663	-0.473	0.919
H(13)	1.084	-0.638	1.830
C(14)	3.124	-0.039	1.106
H(15)	3.188	0.561	2.024
H(16)	3.799	-0.894	1.235
C(17)	0.045	-2.265	0.004
H(18)	0.331	-3.082	0.688
H(19)	-0.218	-2.734	-0.955
C(20)	-1.162	-1.497	0.560
H(21)	-1.000	-1.222	1.613
H(22)	-2.034	-2.160	0.539
C(23)	-1.479	-0.248	-0.278
H(24)	-1.435	-0.544	-1.346
C(25)	-2.947	0.262	-0.047
C(26)	-3.238	0.600	1.423
H(27)	-3.114	-0.280	2.065
H(28)	-4.276	0.938	1.516
H(29)	-2.587	1.404	1.791
C(30)	-3.274	1.463	-0.951
H(31)	-3.031	1.251	-2.004
H(32)	-4.347	1.684	-0.883
H(33)	-2.731	2.369	-0.652
O(34)	-3.827	-0.835	-0.356
H(35)	-3.906	-0.891	-1.322
C(36)	-0.425	0.865	-0.084
H(37)	-0.564	1.340	0.900
H(38)	-0.569	1.640	-0.845
C(39)	1.851	2.796	-0.047
H(40)	2.664	3.463	-0.356
H(41)	1.838	2.767	1.052
H(42)	0.909	3.233	-0.395
H(43)	2.091	1.475	-1.743

Transition State A-TS6 (–662,8311363 Hartree)

C(1)	3.343	1.290	-0.032
H(2)	3.790	1.267	-1.039
H(3)	3.936	2.025	0.534
C(4)	1.920	1.788	-0.102
C(5)	1.007	0.752	0.255
C(6)	2.492	-2.728	-0.618
H(7)	3.399	-2.340	-1.097
H(8)	2.119	-3.547	-1.247
H(9)	2.769	-3.159	0.355
C(10)	1.395	-1.661	-0.457
C(11)	1.762	-0.545	0.580
H(12)	1.424	-0.886	1.571
C(13)	3.256	-0.093	0.661
H(14)	3.545	0.012	1.714
H(15)	3.944	-0.815	0.213
C(16)	0.056	-2.367	-0.116
H(17)	0.277	-3.202	0.567
H(18)	-0.312	-2.829	-1.045
C(19)	-1.076	-1.537	0.517
H(20)	-0.874	-1.377	1.588
H(21)	-1.991	-2.139	0.465
C(22)	-1.366	-0.173	-0.148
H(23)	-1.130	-0.243	-1.228
C(24)	-2.892	0.204	-0.077
C(25)	-3.396	0.343	1.367
H(26)	-3.231	-0.579	1.937
H(27)	-4.473	0.545	1.357
H(28)	-2.901	1.175	1.886
C(29)	-3.204	1.480	-0.884
H(30)	-2.808	1.411	-1.909
H(31)	-4.292	1.608	-0.945
H(32)	-2.794	2.387	-0.420
O(33)	-3.638	-0.895	-0.628
H(34)	-3.512	-0.889	-1.591
C(35)	-0.474	0.938	0.458
H(36)	-0.612	0.945	1.553
H(37)	-0.774	1.932	0.104
C(38)	1.592	3.202	-0.456
H(39)	2.258	3.579	-1.242
H(40)	1.778	3.809	0.445
H(41)	0.548	3.352	-0.749
H(42)	1.249	0.919	-0.985
H(43)	1.281	-1.163	-1.442

Intermediate B = F3 (–662,8370059 Hartree)

C(1)	-3.437	-1.129	-0.745
H(2)	-4.342	-1.573	-0.312
H(3)	-3.465	-1.307	-1.832
C(4)	-3.332	0.381	-0.466
H(5)	-3.579	0.587	0.584
H(6)	-4.008	0.988	-1.081
C(7)	-1.862	0.728	-0.784
C(8)	-1.151	1.721	-0.002
C(9)	-1.560	2.079	1.376
H(10)	-0.769	2.584	1.939
H(11)	-1.966	1.231	1.939
H(12)	-2.400	2.793	1.268
C(13)	0.065	2.347	-0.547
H(14)	0.138	3.390	-0.207
H(15)	0.099	2.304	-1.642
C(16)	1.316	1.583	0.042
H(17)	2.195	2.131	-0.319
H(18)	1.308	1.665	1.138
C(19)	1.425	0.114	-0.390
H(20)	1.354	0.084	-1.495
C(21)	2.855	-0.442	-0.037
C(22)	3.092	-0.545	1.477
H(23)	3.032	0.438	1.960
H(24)	4.097	-0.943	1.661
H(25)	2.364	-1.218	1.949
C(26)	3.106	-1.799	-0.718
H(27)	4.150	-2.095	-0.556
H(28)	2.924	-1.739	-1.802
H(29)	2.471	-2.598	-0.314
O(30)	3.831	0.512	-0.510
H(31)	3.950	0.369	-1.463
C(32)	0.276	-0.749	0.186
H(33)	0.101	-0.482	1.239
H(34)	0.580	-1.806	0.197
C(35)	-1.029	-0.722	-0.617
C(36)	-2.140	-1.704	-0.144
C(37)	-2.233	-1.912	1.375
H(38)	-1.308	-2.325	1.795
H(39)	-2.460	-0.984	1.917
H(40)	-3.041	-2.621	1.600
H(41)	-0.778	-0.955	-1.661
H(42)	-1.735	0.967	-1.849
H(43)	-1.898	-2.677	-0.605

Intermediate B1 = F2 (–662,841873 Hartree)

C(1)	-2.813	-1.351	0.896
H(2)	-2.167	-1.706	1.713
H(3)	-3.811	-1.772	1.062
C(4)	-2.822	0.194	0.863
H(5)	-2.705	0.709	1.829
H(6)	-3.777	0.588	0.455
C(7)	-1.793	0.592	-0.135
C(8)	-1.448	1.985	-0.473
C(9)	-1.734	3.013	0.644
H(10)	-1.559	4.024	0.262
H(11)	-1.066	2.855	1.501
H(12)	-2.770	2.964	0.998
C(13)	-0.056	2.165	-1.146
H(14)	0.066	3.246	-1.303
H(15)	-0.060	1.711	-2.150
C(16)	1.130	1.625	-0.332
H(17)	2.055	2.098	-0.684
H(18)	1.018	1.918	0.724
C(19)	1.332	0.098	-0.435
H(20)	1.411	-0.157	-1.510
C(21)	2.707	-0.351	0.203
C(22)	2.794	-0.056	1.707
H(23)	2.701	1.017	1.910
H(24)	3.770	-0.383	2.084
H(25)	2.017	-0.594	2.266
C(26)	2.992	-1.839	-0.073
H(27)	4.023	-2.069	0.223
H(28)	2.873	-2.080	-1.141
H(29)	2.331	-2.504	0.501
O(30)	3.737	0.451	-0.398
H(31)	3.914	0.098	-1.285
C(32)	0.154	-0.695	0.134
H(33)	-0.030	-0.416	1.183
H(34)	0.350	-1.774	0.139
C(35)	-1.200	-0.591	-0.746
C(36)	-2.210	-1.746	-0.473
C(37)	-1.664	-3.173	-0.558
H(38)	-1.149	-3.352	-1.511
H(39)	-0.969	-3.402	0.262
H(40)	-2.490	-3.892	-0.486
H(41)	-0.878	-0.476	-1.789
H(42)	-2.997	-1.635	-1.240
H(43)	-2.205	2.163	-1.278

Intermediate B2 = F1 (–662,8414939 Hartree)

C(1)	-3.217	-1.282	-0.994
H(2)	-4.163	-1.753	-0.705
H(3)	-3.023	-1.535	-2.047
C(4)	-3.204	0.246	-0.799
H(5)	-3.739	0.539	0.117
H(6)	-3.656	0.796	-1.631
C(7)	-1.695	0.579	-0.647
C(8)	-1.297	1.984	-0.117
C(9)	-1.581	2.126	1.390
H(10)	-1.368	3.152	1.715
H(11)	-0.959	1.459	2.003
H(12)	-2.632	1.916	1.627
C(13)	0.138	2.398	-0.511
H(14)	0.264	3.453	-0.228
H(15)	0.224	2.366	-1.611
C(16)	1.296	1.598	0.105
H(17)	2.238	2.071	-0.200
H(18)	1.258	1.667	1.203
C(19)	1.378	0.115	-0.314
H(20)	1.190	0.045	-1.402
C(21)	2.819	-0.491	-0.090
C(22)	3.238	-0.472	1.387
H(23)	3.263	0.552	1.777
H(24)	4.244	-0.895	1.486
H(25)	2.557	-1.072	2.005
C(26)	2.943	-1.909	-0.679
H(27)	3.998	-2.211	-0.659
H(28)	2.592	-1.940	-1.723
H(29)	2.380	-2.660	-0.109
O(30)	3.741	0.380	-0.763
H(31)	3.712	0.179	-1.713
C(32)	0.296	-0.756	0.404
H(33)	0.237	-0.388	1.450
H(34)	0.569	-1.816	0.478
C(35)	-1.074	-0.637	-0.101
C(36)	-2.032	-1.768	-0.121
C(37)	-2.447	-1.959	1.390
H(38)	-1.603	-2.304	1.997
H(39)	-2.845	-1.033	1.824
H(40)	-3.238	-2.718	1.410
H(41)	-1.261	0.493	-1.683
H(42)	-1.562	-2.709	-0.445
H(43)	-1.978	2.671	-0.645

Intermediate B3 = F (–662,833760 Hartree)

C(1)	3.457	0.816	-0.265
H(2)	3.883	0.468	0.700
H(3)	4.187	1.564	-0.617
C(4)	3.129	-0.350	-1.222
H(5)	3.787	-1.212	-1.064
H(6)	3.269	-0.015	-2.260
C(7)	1.633	-0.692	-0.962
C(8)	1.405	-1.845	0.047
C(9)	1.876	-1.534	1.478
H(10)	1.615	-2.358	2.154
H(11)	1.408	-0.629	1.896
H(12)	2.966	-1.407	1.533
C(13)	-0.035	-2.405	-0.011
H(14)	-0.050	-3.341	0.570
H(15)	-0.249	-2.692	-1.054
C(16)	-1.171	-1.509	0.503
H(17)	-2.104	-2.084	0.447
H(18)	-1.013	-1.279	1.569
C(19)	-1.381	-0.193	-0.276
H(20)	-1.256	-0.401	-1.357
C(21)	-2.858	0.345	-0.123
C(22)	-3.202	0.707	1.329
H(23)	-3.095	-0.162	1.988
H(24)	-4.242	1.048	1.385
H(25)	-2.557	1.515	1.700
C(26)	-3.127	1.538	-1.063
H(27)	-4.197	1.778	-1.038
H(28)	-2.851	1.295	-2.101
H(29)	-2.582	2.445	-0.771
O(30)	-3.757	-0.723	-0.465
H(31)	-3.731	-0.840	-1.429
C(32)	-0.342	0.871	0.139
H(33)	-0.224	0.841	1.232
H(34)	-0.702	1.880	-0.101
C(35)	1.050	0.702	-0.516
C(36)	2.165	1.434	0.081
C(37)	2.023	2.621	0.939
H(38)	1.224	3.287	0.583
H(39)	1.669	2.263	1.925
H(40)	2.960	3.166	1.087
H(41)	1.152	-0.984	-1.905
H(42)	1.018	1.274	-1.486
H(43)	2.049	-2.661	-0.329

Transition State B-TS1 = F-TS3 (–662,8321571 Hartree)

C(1)	-2.846	-1.400	0.854
H(2)	-2.216	-1.689	1.709
H(3)	-3.816	-1.894	0.981
C(4)	-2.982	0.140	0.794
H(5)	-2.908	0.635	1.772
H(6)	-3.952	0.433	0.363
C(7)	-1.842	0.577	-0.122
C(8)	-1.396	1.912	-0.295
C(9)	-2.050	3.064	0.419
H(10)	-2.146	3.933	-0.244
H(11)	-1.372	3.360	1.235
H(12)	-3.024	2.821	0.854
C(13)	-0.096	2.180	-1.028
H(14)	0.004	3.263	-1.174
H(15)	-0.103	1.721	-2.028
C(16)	1.096	1.622	-0.208
H(17)	2.001	2.164	-0.506
H(18)	0.936	1.845	0.859
C(19)	1.370	0.116	-0.406
H(20)	1.460	-0.052	-1.498
C(21)	2.775	-0.270	0.188
C(22)	2.862	-0.063	1.707
H(23)	2.707	0.989	1.977
H(24)	3.860	-0.351	2.057
H(25)	2.123	-0.679	2.237
C(26)	3.162	-1.716	-0.175
H(27)	4.212	-1.883	0.095
H(28)	3.047	-1.903	-1.254
H(29)	2.561	-2.459	0.365
O(30)	3.750	0.636	-0.369
H(31)	3.945	0.342	-1.273
C(32)	0.234	-0.799	0.093
H(33)	0.069	-0.651	1.172
H(34)	0.538	-1.844	-0.032
C(35)	-1.111	-0.636	-0.677
C(36)	-2.149	-1.782	-0.468
C(37)	-1.604	-3.211	-0.511
H(38)	-1.038	-3.400	-1.434
H(39)	-0.950	-3.426	0.344
H(40)	-2.433	-3.930	-0.477
H(41)	-0.869	-0.511	-1.745
H(42)	-2.287	1.339	-1.106
H(43)	-2.890	-1.672	-1.281

Transition State B-TS2 = F-TS2 (–662,8356786 Hartree)

C(1)	-3.302	-1.203	-0.948
H(2)	-4.240	-1.680	-0.645
H(3)	-3.186	-1.354	-2.032
C(4)	-3.269	0.309	-0.633
H(5)	-3.813	0.545	0.298
H(6)	-3.690	0.952	-1.416
C(7)	-1.811	0.614	-0.345
C(8)	-1.316	2.022	-0.122
C(9)	-1.484	2.331	1.391
H(10)	-1.162	3.363	1.578
H(11)	-0.875	1.667	2.016
H(12)	-2.530	2.241	1.710
C(13)	0.109	2.320	-0.647
H(14)	0.262	3.400	-0.506
H(15)	0.123	2.151	-1.737
C(16)	1.281	1.572	0.004
H(17)	2.213	2.049	-0.326
H(18)	1.246	1.694	1.098
C(19)	1.392	0.077	-0.349
H(20)	1.240	-0.031	-1.443
C(21)	2.843	-0.477	-0.073
C(22)	3.228	-0.403	1.412
H(23)	3.212	0.632	1.775
H(24)	4.246	-0.788	1.543
H(25)	2.554	-1.009	2.032
C(26)	3.018	-1.913	-0.605
H(27)	4.081	-2.181	-0.557
H(28)	2.687	-1.996	-1.652
H(29)	2.469	-2.657	-0.014
O(30)	3.772	0.390	-0.747
H(31)	3.754	0.171	-1.693
C(32)	0.310	-0.783	0.348
H(33)	0.306	-0.553	1.427
H(34)	0.545	-1.850	0.258
C(35)	-1.107	-0.603	-0.121
C(36)	-2.073	-1.782	-0.200
C(37)	-2.391	-2.227	1.249
H(38)	-1.496	-2.587	1.771
H(39)	-2.825	-1.405	1.836
H(40)	-3.119	-3.046	1.220
H(41)	-1.134	-0.020	-1.316
H(42)	-1.615	-2.626	-0.736
H(43)	-2.008	2.682	-0.669

Transition State B-TS3 = F-TS1 (–662,8313482 Hartree)

C(1)	3.491	0.808	-0.298
H(2)	3.945	0.459	0.645
H(3)	4.197	1.525	-0.736
C(4)	3.094	-0.363	-1.228
H(5)	3.730	-1.243	-1.081
H(6)	3.205	-0.050	-2.276
C(7)	1.597	-0.675	-0.926
C(8)	1.362	-1.906	0.013
C(9)	1.861	-1.682	1.451
H(10)	1.690	-2.584	2.051
H(11)	1.338	-0.859	1.961
H(12)	2.938	-1.472	1.484
C(13)	-0.091	-2.423	-0.051
H(14)	-0.127	-3.370	0.509
H(15)	-0.324	-2.679	-1.099
C(16)	-1.194	-1.501	0.490
H(17)	-2.144	-2.047	0.442
H(18)	-1.014	-1.283	1.556
C(19)	-1.387	-0.175	-0.280
H(20)	-1.259	-0.373	-1.362
C(21)	-2.857	0.373	-0.129
C(22)	-3.223	0.687	1.330
H(23)	-3.130	-0.203	1.963
H(24)	-4.264	1.027	1.378
H(25)	-2.587	1.483	1.740
C(26)	-3.103	1.604	-1.023
H(27)	-4.176	1.835	-1.022
H(28)	-2.792	1.414	-2.062
H(29)	-2.580	2.503	-0.668
O(30)	-3.760	-0.676	-0.519
H(31)	-3.713	-0.767	-1.485
C(32)	-0.344	0.881	0.137
H(33)	-0.338	0.973	1.236
H(34)	-0.630	1.876	-0.235
C(35)	1.086	0.610	-0.283
C(36)	2.180	1.443	0.094
C(37)	2.087	2.698	0.900
H(38)	1.082	3.130	0.913
H(39)	2.349	2.430	1.937
H(40)	2.815	3.446	0.566
H(41)	1.043	-0.877	-1.854
H(42)	1.485	1.543	-1.088
H(43)	1.990	-2.696	-0.433

Transition State B-TS4 = F-TS5 (–662,7926137 Hartree)

C(1)	-2.300	-1.439	1.332
H(2)	-1.605	-2.190	1.732
H(3)	-3.296	-1.642	1.742
C(4)	-1.860	0.038	1.521
H(5)	-0.919	0.188	2.064
H(6)	-2.631	0.644	2.009
C(7)	-1.697	0.385	0.032
C(8)	-1.676	1.765	-0.576
C(9)	-2.295	2.831	0.342
H(10)	-2.297	3.801	-0.170
H(11)	-1.718	2.945	1.269
H(12)	-3.334	2.591	0.605
C(13)	-0.173	2.058	-0.958
H(14)	-0.074	3.152	-0.935
H(15)	-0.012	1.753	-2.002
C(16)	0.936	1.429	-0.078
H(17)	1.824	2.068	-0.162
H(18)	0.650	1.459	0.986
C(19)	1.408	0.006	-0.485
H(20)	1.512	0.011	-1.588
C(21)	2.852	-0.287	0.066
C(22)	2.935	-0.206	1.598
H(23)	2.667	0.794	1.959
H(24)	3.964	-0.410	1.917
H(25)	2.280	-0.947	2.077
C(26)	3.378	-1.649	-0.426
H(27)	4.440	-1.738	-0.167
H(28)	3.279	-1.745	-1.519
H(29)	2.851	-2.492	0.038
O(30)	3.726	0.758	-0.403
H(31)	3.897	0.600	-1.345
C(32)	0.404	-1.124	-0.138
H(33)	0.393	-1.292	0.948
H(34)	0.720	-2.070	-0.597
C(35)	-0.985	-0.765	-0.647
C(36)	-2.277	-1.441	-0.199
C(37)	-3.074	-2.359	-1.080
H(38)	-3.085	-2.028	-2.126
H(39)	-2.556	-3.333	-1.052
H(40)	-4.098	-2.499	-0.718
H(41)	-0.986	-0.628	-1.737
H(42)	-2.895	-0.238	-0.301
H(43)	-2.243	1.737	-1.522

Intermediate C = G3 (–662,8338407 Hartree)

C(1)	1.717	-1.851	0.052
C(2)	0.666	-0.750	-0.255
H(3)	0.116	-1.060	-1.151
C(4)	-0.333	-0.441	0.869
H(5)	0.206	0.020	1.714
H(6)	-0.697	-1.401	1.260
C(7)	-1.542	0.457	0.488
H(8)	-1.878	0.935	1.426
C(9)	-2.808	-0.311	-0.048
C(10)	-2.541	-1.158	-1.301
H(11)	-1.861	-1.990	-1.080
H(12)	-2.124	-0.559	-2.121
H(13)	-3.489	-1.581	-1.653
C(14)	-3.419	-1.189	1.061
H(15)	-3.639	-0.594	1.960
H(16)	-4.356	-1.630	0.699
H(17)	-2.756	-2.012	1.358
O(18)	-3.774	0.675	-0.463
H(19)	-4.221	1.006	0.332
C(20)	-1.126	1.601	-0.460
H(21)	-1.961	2.300	-0.570
H(22)	-0.927	1.219	-1.472
C(23)	0.095	2.352	0.075
H(24)	-0.071	2.757	1.090
H(25)	0.286	3.274	-0.523
C(26)	1.414	1.684	0.045
C(27)	1.608	0.498	-0.775
H(28)	1.137	0.749	-1.744
C(29)	3.024	-0.084	-0.977
H(30)	3.687	0.174	-0.141
H(31)	3.491	0.310	-1.890
C(32)	2.809	-1.610	-1.011
H(33)	2.444	-1.918	-2.003
H(34)	3.733	-2.165	-0.809
C(35)	2.514	2.288	0.831
H(36)	2.864	1.549	1.572
H(37)	3.389	2.470	0.187
H(38)	2.222	3.206	1.349
C(39)	2.272	-1.836	1.484
H(40)	3.034	-2.618	1.597
H(41)	2.752	-0.881	1.743
H(42)	1.492	-2.025	2.231
H(43)	1.219	-2.821	-0.116

Intermediate C1 = G2 (–662,8367535 Hartree)

C(1)	-1.471	-2.030	0.084
C(2)	-0.688	-0.671	0.071
H(3)	-0.207	-0.621	1.088
C(4)	0.414	-0.468	-0.990
H(5)	-0.069	-0.124	-1.915
H(6)	0.812	-1.464	-1.220
C(7)	1.569	0.487	-0.604
H(8)	1.988	0.846	-1.561
C(9)	2.758	-0.222	0.140
C(10)	2.324	-0.962	1.417
H(11)	1.667	-1.812	1.183
H(12)	1.825	-0.291	2.129
H(13)	3.212	-1.361	1.922
C(14)	3.502	-1.190	-0.800
H(15)	3.842	-0.669	-1.708
H(16)	4.380	-1.598	-0.285
H(17)	2.878	-2.036	-1.117
O(18)	3.673	0.790	0.602
H(19)	4.183	1.100	-0.163
C(20)	1.074	1.738	0.154
H(21)	1.885	2.475	0.154
H(22)	0.912	1.506	1.219
C(23)	-0.205	2.361	-0.434
H(24)	-0.239	2.239	-1.528
H(25)	-0.205	3.446	-0.258
C(26)	-1.519	1.836	0.183
C(27)	-1.693	0.374	0.300
C(28)	-2.958	-0.245	0.764
H(29)	-3.612	-0.186	-0.135
H(30)	-3.488	0.333	1.534
C(31)	-2.623	-1.721	1.068
H(32)	-2.279	-1.811	2.110
H(33)	-3.491	-2.378	0.940
C(34)	-2.778	2.537	-0.411
H(35)	-2.916	2.240	-1.458
H(36)	-3.694	2.314	0.147
H(37)	-2.617	3.620	-0.382
C(38)	-1.965	-2.442	-1.313
H(39)	-2.544	-3.371	-1.246
H(40)	-2.611	-1.678	-1.770
H(41)	-1.130	-2.621	-2.001
H(42)	-0.812	-2.817	0.476
H(43)	-1.511	2.113	1.267

Intermediate C2 = G1 (–662,8356142 Hartree)

C(1)	-1.393	-1.904	-0.166
C(2)	-0.751	-0.573	-0.265
H(3)	-0.560	0.295	1.462
C(4)	0.325	-0.237	-1.194
H(5)	-0.156	0.445	-1.923
H(6)	0.662	-1.118	-1.750
C(7)	1.551	0.577	-0.617
H(8)	2.045	0.952	-1.529
C(9)	2.599	-0.347	0.101
C(10)	2.041	-1.053	1.347
H(11)	1.219	-1.737	1.091
H(12)	1.697	-0.336	2.103
H(13)	2.834	-1.655	1.807
C(14)	3.207	-1.381	-0.865
H(15)	3.574	-0.899	-1.784
H(16)	4.051	-1.880	-0.374
H(17)	2.488	-2.159	-1.156
O(18)	3.631	0.521	0.598
H(19)	4.193	0.781	-0.150
C(20)	1.148	1.816	0.225
H(21)	1.982	2.525	0.169
H(22)	1.084	1.559	1.293
C(23)	-0.150	2.498	-0.236
H(24)	-0.185	2.522	-1.338
H(25)	-0.134	3.554	0.068
C(26)	-1.471	1.881	0.314
C(27)	-1.361	0.373	0.678
C(28)	-2.591	-0.361	1.277
H(29)	-3.490	-0.135	0.687
H(30)	-2.789	-0.048	2.308
C(31)	-2.208	-1.850	1.151
H(32)	-1.563	-2.153	1.990
H(33)	-3.073	-2.523	1.130
C(34)	-2.640	2.155	-0.647
H(35)	-2.522	1.604	-1.593
H(36)	-3.613	1.891	-0.215
H(37)	-2.671	3.224	-0.894
C(38)	-2.322	-1.996	-1.438
H(39)	-2.889	-2.931	-1.352
H(40)	-3.032	-1.163	-1.486
H(41)	-1.734	-2.018	-2.362
H(42)	-0.666	-2.727	-0.240
H(43)	-1.697	2.375	1.273

Intermediate C3 = G (–662,8271801 Hartree)

C(1)	-1.922	-1.479	-0.151
C(2)	-0.734	-0.680	0.122
H(3)	-0.461	0.806	1.684
C(4)	0.325	-0.542	-0.986
H(5)	-0.190	-0.139	-1.872
H(6)	0.674	-1.542	-1.276
C(7)	1.551	0.357	-0.677
H(8)	1.994	0.524	-1.676
C(9)	2.683	-0.338	0.160
C(10)	2.222	-0.832	1.541
H(11)	1.562	-1.708	1.452
H(12)	1.715	-0.046	2.114
H(13)	3.099	-1.147	2.118
C(14)	3.326	-1.502	-0.621
H(15)	3.690	-1.163	-1.603
H(16)	4.178	-1.896	-0.054
H(17)	2.628	-2.333	-0.794
O(18)	3.690	0.646	0.455
H(19)	4.163	0.846	-0.369
C(20)	1.188	1.767	-0.133
H(21)	1.996	2.450	-0.419
H(22)	1.206	1.774	0.966
C(23)	-0.147	2.320	-0.660
H(24)	-0.225	2.100	-1.737
H(25)	-0.131	3.417	-0.591
C(26)	-1.443	1.819	0.061
C(27)	-1.224	0.563	0.936
C(28)	-2.493	0.024	1.653
H(29)	-3.235	0.806	1.846
H(30)	-2.220	-0.417	2.622
C(31)	-3.040	-1.084	0.727
H(32)	-3.511	-1.958	1.210
H(33)	-3.825	-0.711	0.037
C(34)	-2.596	1.685	-0.946
H(35)	-2.428	0.855	-1.655
H(36)	-3.570	1.529	-0.460
H(37)	-2.679	2.596	-1.552
C(38)	-2.011	-2.532	-1.175
H(39)	-2.909	-3.150	-1.091
H(40)	-2.015	-2.028	-2.161
H(41)	-1.102	-3.151	-1.189
H(42)	-0.316	-1.364	0.919
H(43)	-1.733	2.598	0.787

Transition State C-TS1 = C-TS3 (–662,8284416 Hartree)

C(1)	-1.531	-2.025	0.143
C(2)	-0.668	-0.718	0.216
H(3)	-0.189	-0.731	1.209
C(4)	0.409	-0.569	-0.876
H(5)	-0.090	-0.288	-1.816
H(6)	0.828	-1.566	-1.063
C(7)	1.552	0.429	-0.573
H(8)	1.923	0.781	-1.553
C(9)	2.802	-0.207	0.137
C(10)	2.469	-0.898	1.469
H(11)	1.813	-1.765	1.315
H(12)	1.997	-0.208	2.181
H(13)	3.397	-1.256	1.929
C(14)	3.523	-1.194	-0.801
H(15)	3.787	-0.709	-1.753
H(16)	4.445	-1.545	-0.322
H(17)	2.913	-2.076	-1.035
O(18)	3.707	0.859	0.491
H(19)	4.188	1.117	-0.312
C(20)	1.051	1.680	0.172
H(21)	1.863	2.414	0.203
H(22)	0.839	1.449	1.230
C(23)	-0.189	2.337	-0.462
H(24)	-0.130	2.291	-1.565
H(25)	-0.223	3.408	-0.216
C(26)	-1.542	1.753	-0.100
C(27)	-1.729	0.389	0.253
H(28)	-1.446	1.293	1.159
C(29)	-3.091	-0.221	0.577
H(30)	-3.684	-0.209	-0.352
H(31)	-3.658	0.352	1.321
C(32)	-2.761	-1.664	1.003
H(33)	-2.504	-1.688	2.073
H(34)	-3.608	-2.343	0.846
C(35)	-2.719	2.690	-0.228
H(36)	-2.796	2.953	-1.295
H(37)	-3.674	2.260	0.083
H(38)	-2.539	3.627	0.313
C(39)	-1.921	-2.438	-1.286
H(40)	-2.576	-3.318	-1.254
H(41)	-2.461	-1.644	-1.824
H(42)	-1.044	-2.703	-1.890
H(43)	-0.957	-2.844	0.600

Transition State C-TS2 = C-TS2 (–662,8317666 Hartree)

C(1)	-1.328	-1.985	-0.050
C(2)	-0.758	-0.588	-0.260
H(3)	-0.483	-0.116	0.949
C(4)	0.393	-0.306	-1.189
H(5)	-0.039	0.196	-2.068
H(6)	0.787	-1.265	-1.542
C(7)	1.556	0.573	-0.627
H(8)	2.055	0.972	-1.527
C(9)	2.649	-0.254	0.143
C(10)	2.084	-1.040	1.337
H(11)	1.400	-1.834	1.009
H(12)	1.571	-0.384	2.055
H(13)	2.909	-1.519	1.878
C(14)	3.406	-1.206	-0.803
H(15)	3.824	-0.657	-1.660
H(16)	4.232	-1.679	-0.258
H(17)	2.768	-2.008	-1.199
O(18)	3.574	0.679	0.727
H(19)	4.149	1.014	0.019
C(20)	1.066	1.793	0.193
H(21)	1.882	2.522	0.218
H(22)	0.925	1.519	1.252
C(23)	-0.220	2.446	-0.345
H(24)	-0.260	2.382	-1.444
H(25)	-0.215	3.520	-0.113
C(26)	-1.524	1.878	0.263
C(27)	-1.588	0.373	0.377
C(28)	-2.722	-0.337	1.096
H(29)	-3.612	-0.228	0.455
H(30)	-2.966	0.120	2.063
C(31)	-2.263	-1.810	1.173
H(32)	-1.694	-1.972	2.101
H(33)	-3.100	-2.517	1.167
C(34)	-2.770	2.355	-0.530
H(35)	-2.794	1.907	-1.533
H(36)	-3.709	2.120	-0.015
H(37)	-2.713	3.444	-0.649
C(38)	-2.074	-2.393	-1.345
H(39)	-2.523	-3.383	-1.203
H(40)	-2.880	-1.686	-1.586
H(41)	-1.396	-2.447	-2.205
H(42)	-0.525	-2.713	0.135
H(43)	-1.622	2.264	1.293

Transition State C-TS3 = C-TS1 (–662,8243181 Hartree)

C(1)	-1.846	-1.537	-0.182
C(2)	-0.807	-0.565	-0.114
H(3)	-0.335	0.696	1.555
C(4)	0.334	-0.462	-1.088
H(5)	-0.136	-0.015	-1.982
H(6)	0.647	-1.463	-1.412
C(7)	1.567	0.395	-0.700
H(8)	2.072	0.553	-1.670
C(9)	2.621	-0.360	0.185
C(10)	2.070	-0.829	1.542
H(11)	1.278	-1.584	1.418
H(12)	1.686	0.007	2.138
H(13)	2.878	-1.298	2.117
C(14)	3.233	-1.557	-0.570
H(15)	3.630	-1.248	-1.548
H(16)	4.057	-1.977	0.021
H(17)	2.507	-2.365	-0.743
O(18)	3.660	0.571	0.530
H(19)	4.184	0.745	-0.269
C(20)	1.224	1.815	-0.156
H(21)	2.030	2.490	-0.468
H(22)	1.271	1.830	0.942
C(23)	-0.122	2.381	-0.646
H(24)	-0.236	2.176	-1.723
H(25)	-0.100	3.477	-0.565
C(26)	-1.393	1.884	0.112
C(27)	-1.171	0.535	0.866
C(28)	-2.400	-0.029	1.635
H(29)	-3.143	0.744	1.857
H(30)	-2.070	-0.451	2.595
C(31)	-2.974	-1.160	0.749
H(32)	-3.377	-2.019	1.301
H(33)	-3.784	-0.801	0.090
C(34)	-2.614	1.870	-0.822
H(35)	-2.502	1.127	-1.628
H(36)	-3.551	1.663	-0.290
H(37)	-2.725	2.848	-1.308
C(38)	-1.935	-2.680	-1.142
H(39)	-2.400	-3.557	-0.677
H(40)	-2.592	-2.360	-1.967
H(41)	-0.969	-2.958	-1.578
H(42)	-0.729	-1.643	0.612
H(43)	-1.599	2.598	0.927

Transition State C-TS4 = C-TS5 (–662,796156 Hartree)

C(1)	2.226	-1.460	-0.252
C(2)	0.924	-0.759	-0.618
H(3)	0.896	-0.566	-1.698
C(4)	-0.455	-1.146	-0.092
H(5)	-0.382	-1.748	0.823
H(6)	-0.922	-1.797	-0.843
C(7)	-1.346	0.108	0.177
H(8)	-1.187	0.394	1.235
C(9)	-2.877	-0.234	0.085
C(10)	-3.339	-0.508	-1.355
H(11)	-2.812	-1.364	-1.796
H(12)	-3.182	0.369	-1.994
H(13)	-4.412	-0.734	-1.355
C(14)	-3.238	-1.414	1.005
H(15)	-2.899	-1.230	2.037
H(16)	-4.327	-1.545	1.019
H(17)	-2.792	-2.359	0.666
O(18)	-3.619	0.933	0.489
H(19)	-3.558	1.003	1.455
C(20)	-0.996	1.359	-0.674
H(21)	-1.890	1.996	-0.652
H(22)	-0.834	1.093	-1.730
C(23)	0.171	2.228	-0.161
H(24)	0.076	2.381	0.926
H(25)	0.109	3.227	-0.618
C(26)	1.637	1.746	-0.471
C(27)	1.677	0.353	0.081
C(28)	1.875	-0.055	1.549
H(29)	0.938	0.048	2.111
H(30)	2.646	0.535	2.055
C(31)	2.323	-1.519	1.277
H(32)	3.342	-1.722	1.625
H(33)	1.661	-2.295	1.685
C(34)	2.695	2.700	0.093
H(35)	2.591	2.821	1.179
H(36)	3.712	2.342	-0.121
H(37)	2.587	3.692	-0.362
C(38)	2.985	-2.341	-1.202
H(39)	4.025	-2.490	-0.893
H(40)	2.472	-3.318	-1.184
H(41)	2.946	-1.973	-2.234
H(42)	2.849	-0.251	-0.328
H(43)	1.726	1.684	-1.569

Intermediate D (–662,8402381 Hartree)

C(1)	-3.450	-0.818	0.438
H(2)	-3.668	-0.201	1.323
H(3)	-4.308	-1.486	0.291
C(4)	-3.187	0.073	-0.792
H(5)	-3.832	0.960	-0.831
H(6)	-3.367	-0.483	-1.721
C(7)	-1.678	0.439	-0.748
C(8)	-1.290	1.743	-0.247
C(9)	0.036	2.309	-0.572
H(10)	0.078	3.371	-0.300
H(11)	0.251	2.193	-1.644
C(12)	1.166	1.538	0.212
H(13)	2.077	2.123	0.035
H(14)	0.954	1.566	1.290
C(15)	1.416	0.110	-0.298
H(16)	1.349	0.151	-1.402
C(17)	2.903	-0.319	-0.007
C(18)	3.199	-1.711	-0.590
H(19)	4.270	-1.925	-0.489
H(20)	2.934	-1.760	-1.658
H(21)	2.648	-2.504	-0.069
C(22)	3.255	-0.270	1.488
H(23)	4.293	-0.591	1.626
H(24)	3.166	0.749	1.884
H(25)	2.612	-0.934	2.081
O(26)	3.765	0.660	-0.620
H(27)	3.819	0.457	-1.568
C(28)	0.392	-0.955	0.185
H(29)	0.368	-1.760	-0.559
H(30)	0.747	-1.407	1.124
C(31)	-1.034	-0.484	0.462
C(32)	-2.132	-1.586	0.643
C(33)	-1.988	-2.772	-0.323
H(34)	-1.089	-3.365	-0.115
H(35)	-2.853	-3.439	-0.212
H(36)	-1.948	-2.457	-1.375
H(37)	-1.038	0.111	1.390
C(38)	-2.153	2.510	0.682
H(39)	-1.560	3.020	1.454
H(40)	-2.974	1.942	1.127
H(41)	-2.595	3.322	0.072
H(42)	-1.135	0.179	-1.665
H(43)	-2.051	-1.958	1.677

Intermediate D1 (–662,8385123 Hartree)

C(1)	-3.230	-1.200	0.951
H(2)	-3.152	-1.123	2.045
H(3)	-4.166	-1.719	0.717
C(4)	-3.176	0.216	0.333
H(5)	-3.657	1.039	0.885
H(6)	-3.673	0.243	-0.662
C(7)	-1.756	0.474	0.021
C(8)	-1.204	1.721	-0.508
C(9)	-0.037	2.260	0.433
H(10)	-0.508	2.720	1.314
H(11)	0.405	3.075	-0.156
C(12)	1.056	1.263	0.853
H(13)	1.954	1.845	1.097
H(14)	0.777	0.751	1.787
C(15)	1.438	0.242	-0.238
H(16)	1.460	0.786	-1.200
C(17)	2.907	-0.279	-0.048
C(18)	3.311	-1.216	-1.200
H(19)	4.372	-1.478	-1.105
H(20)	3.160	-0.729	-2.177
H(21)	2.735	-2.151	-1.201
C(22)	3.125	-0.957	1.314
H(23)	4.159	-1.313	1.381
H(24)	2.962	-0.249	2.135
H(25)	2.460	-1.819	1.454
O(26)	3.780	0.867	-0.031
H(27)	3.904	1.161	-0.947
C(28)	0.408	-0.926	-0.380
H(29)	0.233	-1.151	-1.442
H(30)	0.823	-1.846	0.054
C(31)	-0.952	-0.719	0.304
C(32)	-1.979	-1.905	0.365
C(33)	-2.226	-2.540	-1.011
H(34)	-1.317	-3.010	-1.407
H(35)	-2.995	-3.319	-0.932
H(36)	-2.572	-1.804	-1.754
H(37)	-0.759	-0.487	1.390
C(38)	-2.214	2.826	-0.836
H(39)	-2.714	3.196	0.069
H(40)	-2.982	2.483	-1.541
H(41)	-1.695	3.676	-1.295
H(42)	-1.602	-2.674	1.053
H(43)	-0.670	1.400	-1.428

Intermediate D2 (–662,8377183 Hartree)

C(1)	3.263	-1.423	-0.772
H(2)	3.287	-1.505	-1.869
H(3)	4.112	-1.990	-0.373
C(4)	3.254	0.053	-0.334
H(5)	3.907	0.690	-0.938
H(6)	3.587	0.146	0.710
C(7)	1.760	0.479	-0.448
C(8)	1.264	1.651	0.478
C(9)	-0.011	2.349	-0.041
H(10)	0.283	3.094	-0.799
H(11)	-0.426	2.921	0.803
C(12)	-1.120	1.465	-0.641
H(13)	-2.046	2.051	-0.660
H(14)	-0.885	1.223	-1.691
C(15)	-1.387	0.158	0.133
H(16)	-1.163	0.317	1.203
C(17)	-2.900	-0.277	0.078
C(18)	-3.177	-1.504	0.966
H(19)	-4.260	-1.663	1.032
H(20)	-2.785	-1.358	1.985
H(21)	-2.737	-2.426	0.561
C(22)	-3.387	-0.523	-1.358
H(23)	-4.453	-0.776	-1.340
H(24)	-3.262	0.370	-1.981
H(25)	-2.854	-1.363	-1.826
O(26)	-3.670	0.839	0.553
H(27)	-3.595	0.868	1.521
C(28)	-0.460	-0.970	-0.376
H(29)	-0.704	-1.961	0.035
H(30)	-0.643	-1.088	-1.471
C(31)	0.995	-0.788	-0.350
C(32)	1.907	-1.949	-0.247
C(33)	1.951	-2.287	1.295
H(34)	0.982	-2.654	1.652
H(35)	2.703	-3.076	1.423
H(36)	2.251	-1.425	1.901
H(37)	1.569	0.804	-1.498
C(38)	2.378	2.698	0.669
H(39)	2.699	3.112	-0.297
H(40)	3.258	2.297	1.185
H(41)	1.990	3.527	1.275
H(42)	1.509	-2.840	-0.757
H(43)	1.052	1.200	1.463

Intermediate D3 (–662,8322194 Hartree)

C(1)	3.034	-1.529	0.477
H(2)	3.846	-1.675	-0.273
H(3)	3.272	-2.243	1.281
C(4)	2.908	-0.037	0.845
H(5)	3.882	0.461	0.879
H(6)	2.451	0.065	1.842
C(7)	1.964	0.543	-0.241
C(8)	1.273	1.856	0.171
C(9)	0.143	2.287	-0.787
H(10)	0.577	2.565	-1.761
H(11)	-0.284	3.215	-0.375
C(12)	-1.006	1.271	-1.010
H(13)	-1.927	1.825	-1.224
H(14)	-0.819	0.658	-1.906
C(15)	-1.273	0.364	0.203
H(16)	-1.171	0.981	1.111
C(17)	-2.761	-0.179	0.238
C(18)	-3.018	-1.026	1.497
H(19)	-4.085	-1.271	1.561
H(20)	-2.731	-0.477	2.407
H(21)	-2.460	-1.972	1.487
C(22)	-3.143	-0.941	-1.039
H(23)	-4.176	-1.298	-0.957
H(24)	-3.079	-0.289	-1.918
H(25)	-2.496	-1.814	-1.201
O(26)	-3.612	0.974	0.257
H(27)	-3.630	1.325	1.163
C(28)	-0.264	-0.824	0.354
H(29)	0.045	-0.938	1.401
H(30)	-0.743	-1.764	0.057
C(31)	1.035	-0.660	-0.580
C(32)	1.810	-1.859	-0.282
C(33)	1.429	-3.234	-0.655
H(34)	0.617	-3.280	-1.388
H(35)	2.308	-3.791	-1.016
H(36)	1.116	-3.769	0.260
H(37)	2.559	0.738	-1.153
C(38)	2.309	2.986	0.317
H(39)	2.817	3.179	-0.639
H(40)	3.076	2.750	1.066
H(41)	1.820	3.918	0.628
H(42)	0.638	-0.677	-1.606
H(43)	0.828	1.689	1.169

Intermediate D4 (–662,8414057 Hartree)

C(1)	-3.331	-0.941	0.364
H(2)	-3.634	-0.253	1.167
H(3)	-4.114	-1.701	0.266
C(4)	-3.069	-0.168	-0.944
H(5)	-3.796	0.623	-1.149
H(6)	-3.084	-0.852	-1.806
C(7)	-1.625	0.359	-0.759
C(8)	-1.503	1.620	0.281
C(9)	-0.116	2.279	0.187
H(10)	-0.164	3.190	0.802
H(11)	0.049	2.621	-0.849
C(12)	1.087	1.448	0.659
H(13)	1.967	2.103	0.677
H(14)	0.918	1.110	1.696
C(15)	1.431	0.242	-0.236
H(16)	1.300	0.545	-1.292
C(17)	2.944	-0.183	-0.115
C(18)	3.274	-1.350	-1.064
H(19)	4.359	-1.510	-1.074
H(20)	2.947	-1.137	-2.094
H(21)	2.810	-2.294	-0.745
C(22)	3.359	-0.522	1.325
H(23)	4.427	-0.770	1.342
H(24)	3.202	0.331	1.995
H(25)	2.806	-1.387	1.715
O(26)	3.729	0.968	-0.464
H(27)	3.710	1.067	-1.430
C(28)	0.489	-0.954	0.032
H(29)	0.619	-1.710	-0.775
H(30)	0.749	-1.493	0.956
C(31)	-0.956	-0.702	-0.012
C(32)	-1.948	-1.569	0.690
C(33)	-1.807	-3.032	0.141
H(34)	-0.820	-3.455	0.358
H(35)	-2.568	-3.647	0.634
H(36)	-1.982	-3.066	-0.941
C(37)	-2.626	2.626	-0.028
H(38)	-2.471	3.510	0.606
H(39)	-3.624	2.236	0.196
H(40)	-2.597	2.948	-1.077
H(41)	-1.103	0.686	-1.669
H(42)	-1.710	-1.617	1.767
H(43)	-1.646	1.251	1.310

Intermediate D5 (–662,8364244 Hartree)

C(1)	3.088	-1.470	0.652
H(2)	3.988	-2.071	0.460
H(3)	2.776	-1.722	1.692
C(4)	3.187	0.066	0.504
H(5)	3.755	0.307	-0.408
H(6)	3.702	0.530	1.351
C(7)	1.712	0.516	0.360
C(8)	1.517	1.853	-0.386
C(9)	0.046	2.323	-0.414
H(10)	-0.003	3.201	-1.075
H(11)	-0.224	2.690	0.591
C(12)	-1.029	1.313	-0.858
H(13)	-1.957	1.876	-1.015
H(14)	-0.772	0.870	-1.836
C(15)	-1.325	0.208	0.181
H(16)	-1.118	0.626	1.183
C(17)	-2.863	-0.187	0.217
C(18)	-3.148	-1.240	1.303
H(19)	-4.233	-1.371	1.405
H(20)	-2.743	-0.929	2.278
H(21)	-2.716	-2.218	1.051
C(22)	-3.389	-0.651	-1.149
H(23)	-4.465	-0.849	-1.071
H(24)	-3.242	0.118	-1.916
H(25)	-2.898	-1.576	-1.477
O(26)	-3.588	1.015	0.509
H(27)	-3.497	1.202	1.458
C(28)	-0.450	-1.052	0.023
H(29)	-0.444	-1.628	0.958
H(30)	-0.854	-1.705	-0.761
C(31)	1.038	-0.687	-0.363
C(32)	1.925	-1.836	-0.180
C(33)	1.711	-3.180	-0.739
H(34)	0.774	-3.295	-1.289
H(35)	2.562	-3.411	-1.406
H(36)	1.782	-3.939	0.059
C(37)	2.403	2.962	0.206
H(38)	2.227	3.913	-0.313
H(39)	3.473	2.733	0.118
H(40)	2.176	3.118	1.271
H(41)	1.274	0.601	1.372
H(42)	1.008	-0.495	-1.459
H(43)	1.837	1.677	-1.433

Transition State D-TS1 (–662,8266305 Hartree)

C(1)	-3.325	-1.214	0.723
H(2)	-3.439	-0.964	1.789
H(3)	-4.181	-1.832	0.429
C(4)	-3.251	0.091	-0.103
H(5)	-3.917	0.886	0.255
H(6)	-3.516	-0.106	-1.154
C(7)	-1.783	0.486	0.007
C(8)	-1.329	1.819	-0.178
C(9)	0.050	2.332	0.189
H(10)	-0.161	3.136	0.918
H(11)	0.449	2.860	-0.694
C(12)	1.103	1.368	0.746
H(13)	2.022	1.953	0.882
H(14)	0.808	1.008	1.742
C(15)	1.415	0.207	-0.214
H(16)	1.344	0.613	-1.243
C(17)	2.909	-0.262	-0.084
C(18)	3.237	-1.358	-1.114
H(19)	4.309	-1.588	-1.074
H(20)	2.991	-1.027	-2.135
H(21)	2.687	-2.288	-0.921
C(22)	3.258	-0.725	1.339
H(23)	4.309	-1.035	1.371
H(24)	3.128	0.089	2.062
H(25)	2.643	-1.578	1.655
O(26)	3.750	0.886	-0.314
H(27)	3.798	1.030	-1.272
C(28)	0.413	-0.975	-0.111
H(29)	0.260	-1.399	-1.112
H(30)	0.849	-1.787	0.488
C(31)	-0.965	-0.699	0.524
C(32)	-1.966	-1.902	0.471
C(33)	-1.954	-2.685	-0.853
H(34)	-1.013	-3.229	-0.999
H(35)	-2.764	-3.425	-0.855
H(36)	-2.101	-2.035	-1.730
H(37)	-0.816	-0.456	1.593
C(38)	-2.314	2.890	-0.605
H(39)	-2.786	3.295	0.303
H(40)	-3.110	2.516	-1.256
H(41)	-1.797	3.716	-1.107
H(42)	-1.264	0.914	-1.146
H(43)	-1.725	-2.597	1.289

Transition State D-TS2 (–662,8319705 Hartree)

C(1)	-3.268	-1.135	0.899
H(2)	-3.219	-1.001	1.990
H(3)	-4.190	-1.683	0.673
C(4)	-3.209	0.245	0.209
H(5)	-3.692	1.054	0.769
H(6)	-3.678	0.226	-0.789
C(7)	-1.728	0.485	-0.025
C(8)	-1.126	1.785	-0.527
C(9)	0.032	2.299	0.380
H(10)	-0.405	2.817	1.248
H(11)	0.556	3.068	-0.205
C(12)	1.049	1.246	0.867
H(13)	1.965	1.775	1.156
H(14)	0.693	0.752	1.789
C(15)	1.413	0.185	-0.193
H(16)	1.354	0.657	-1.190
C(17)	2.905	-0.293	-0.062
C(18)	3.269	-1.293	-1.175
H(19)	4.345	-1.500	-1.141
H(20)	3.027	-0.887	-2.170
H(21)	2.743	-2.251	-1.063
C(22)	3.229	-0.881	1.320
H(23)	4.293	-1.143	1.356
H(24)	3.036	-0.155	2.119
H(25)	2.653	-1.793	1.525
O(26)	3.735	0.876	-0.166
H(27)	3.774	1.135	-1.101
C(28)	0.427	-1.014	-0.176
H(29)	0.440	-1.537	-1.148
H(30)	0.752	-1.769	0.556
C(31)	-1.026	-0.747	0.095
C(32)	-2.001	-1.880	0.403
C(33)	-2.239	-2.686	-0.899
H(34)	-1.327	-3.192	-1.237
H(35)	-3.000	-3.452	-0.708
H(36)	-2.601	-2.043	-1.713
H(37)	-1.162	0.131	1.111
C(38)	-2.173	2.891	-0.743
H(39)	-2.639	3.197	0.204
H(40)	-2.964	2.586	-1.439
H(41)	-1.681	3.775	-1.167
H(42)	-1.588	-2.557	1.164
H(43)	-0.690	1.528	-1.508

Transition State D-TS3 (–662,822887 Hartree)

C(1)	2.518	2.006	0.650
H(2)	2.378	2.331	1.695
H(3)	3.204	2.737	0.197
C(4)	2.990	0.538	0.521
H(5)	3.595	0.212	1.373
H(6)	3.607	0.432	-0.384
C(7)	1.689	-0.295	0.362
C(8)	1.808	-1.601	-0.459
C(9)	0.498	-2.466	-0.433
H(10)	0.720	-3.389	0.121
H(11)	0.264	-2.787	-1.460
C(12)	-0.762	-1.858	0.212
H(13)	-1.501	-2.655	0.351
H(14)	-0.537	-1.518	1.234
C(15)	-1.444	-0.725	-0.584
H(16)	-1.908	-1.189	-1.473
C(17)	-2.620	-0.049	0.213
C(18)	-3.545	0.753	-0.723
H(19)	-4.389	1.156	-0.151
H(20)	-3.943	0.110	-1.522
H(21)	-3.034	1.599	-1.205
C(22)	-2.132	0.851	1.364
H(23)	-2.988	1.133	1.987
H(24)	-1.411	0.338	2.014
H(25)	-1.696	1.789	0.983
O(26)	-3.380	-1.077	0.866
H(27)	-3.895	-1.545	0.188
C(28)	-0.432	0.301	-1.158
H(29)	0.080	-0.163	-2.019
H(30)	-0.943	1.183	-1.560
C(31)	0.711	0.709	-0.272
C(32)	1.196	2.033	-0.094
C(33)	0.602	3.300	-0.624
H(34)	-0.438	3.193	-0.949
H(35)	0.680	4.110	0.112
H(36)	1.203	3.599	-1.498
H(37)	1.310	-0.562	1.364
C(38)	3.004	-2.438	0.028
H(39)	2.884	-2.708	1.088
H(40)	3.961	-1.914	-0.087
H(41)	3.067	-3.369	-0.548
H(42)	0.268	1.426	0.704
H(43)	2.019	-1.302	-1.502

Transition State D-TS4 (–662,7831071 Hartree)

C(1)	2.308	-1.791	1.045
H(2)	3.307	-2.029	1.430
H(3)	1.693	-2.700	1.103
C(4)	1.680	-0.539	1.708
H(5)	2.345	-0.065	2.440
H(6)	0.709	-0.707	2.194
C(7)	1.503	0.312	0.444
C(8)	1.271	1.804	0.418
C(9)	0.268	2.186	-0.695
H(10)	0.805	2.124	-1.655
H(11)	0.048	3.255	-0.563
C(12)	-1.087	1.400	-0.792
H(13)	-1.896	2.064	-0.465
H(14)	-1.274	1.194	-1.856
C(15)	-1.292	0.087	0.014
H(16)	-1.001	0.295	1.062
C(17)	-2.820	-0.283	0.104
C(18)	-3.034	-1.541	0.967
H(19)	-4.107	-1.673	1.151
H(20)	-2.526	-1.449	1.941
H(21)	-2.665	-2.450	0.475
C(22)	-3.476	-0.464	-1.273
H(23)	-4.524	-0.755	-1.140
H(24)	-3.459	0.471	-1.845
H(25)	-2.976	-1.247	-1.858
O(26)	-3.513	0.830	0.702
H(27)	-3.347	0.805	1.658
C(28)	-0.411	-1.107	-0.461
H(29)	-0.435	-1.915	0.283
H(30)	-0.788	-1.529	-1.402
C(31)	0.999	-0.572	-0.670
C(32)	2.336	-1.254	-0.386
C(33)	3.260	-1.735	-1.467
H(34)	3.269	-1.067	-2.336
H(35)	4.280	-1.896	-1.102
H(36)	2.852	-2.704	-1.805
H(37)	1.070	-0.045	-1.630
C(38)	2.567	2.641	0.360
H(39)	3.074	2.496	-0.605
H(40)	3.260	2.367	1.166
H(41)	2.332	3.707	0.459
H(42)	2.802	-0.034	-0.026
H(43)	0.820	1.981	1.416

Transition State D-TS5 (–662,8272767 Hartree)

C(1)	-3.465	0.859	0.018
H(2)	-4.091	0.242	-0.639
H(3)	-4.073	1.732	0.284
C(4)	-3.020	0.075	1.279
H(5)	-3.698	-0.752	1.526
H(6)	-2.990	0.735	2.156
C(7)	-1.590	-0.407	1.003
C(8)	-1.351	-1.472	-0.060
C(9)	-0.056	-2.290	0.072
H(10)	-0.187	-3.222	-0.494
H(11)	0.049	-2.563	1.133
C(12)	1.217	-1.577	-0.414
H(13)	2.081	-2.221	-0.214
H(14)	1.158	-1.447	-1.508
C(15)	1.449	-0.218	0.265
H(16)	1.311	-0.352	1.354
C(17)	2.932	0.277	0.099
C(18)	3.162	1.605	0.842
H(19)	4.234	1.837	0.844
H(20)	2.819	1.544	1.887
H(21)	2.643	2.445	0.362
C(22)	3.365	0.402	-1.370
H(23)	4.408	0.736	-1.409
H(24)	3.306	-0.564	-1.886
H(25)	2.755	1.134	-1.914
O(26)	3.783	-0.745	0.649
H(27)	3.768	-0.660	1.616
C(28)	0.418	0.826	-0.213
H(29)	0.472	1.722	0.423
H(30)	0.646	1.151	-1.238
C(31)	-1.040	0.401	-0.150
C(32)	-2.164	1.302	-0.715
C(33)	-1.824	2.784	-0.436
H(34)	-0.917	3.112	-0.956
H(35)	-2.657	3.401	-0.794
H(36)	-1.704	2.974	0.639
H(37)	-1.043	-0.623	-1.004
C(38)	-2.523	-2.199	-0.702
H(39)	-2.216	-2.693	-1.631
H(40)	-3.394	-1.573	-0.898
H(41)	-2.824	-2.980	0.014
H(42)	-0.938	-0.524	1.872
H(43)	-2.249	1.177	-1.805

Transition State D-TS6 (–662,8295072 Hartree)

C(1)	-3.287	1.272	-0.062
H(2)	-3.725	1.027	-1.043
H(3)	-3.897	2.096	0.339
C(4)	-3.171	0.061	0.893
H(5)	-3.947	-0.688	0.710
H(6)	-3.290	0.406	1.930
C(7)	-1.727	-0.490	0.691
C(8)	-1.621	-1.646	-0.357
C(9)	-0.181	-2.155	-0.589
H(10)	-0.239	-2.901	-1.396
H(11)	0.139	-2.712	0.308
C(12)	0.916	-1.136	-0.942
H(13)	1.785	-1.697	-1.309
H(14)	0.593	-0.492	-1.782
C(15)	1.395	-0.281	0.250
H(16)	1.308	-0.903	1.160
C(17)	2.919	0.090	0.147
C(18)	3.389	0.869	1.389
H(19)	4.478	0.994	1.350
H(20)	3.137	0.331	2.316
H(21)	2.942	1.872	1.448
C(22)	3.260	0.862	-1.137
H(23)	4.342	1.037	-1.174
H(24)	2.982	0.291	-2.031
H(25)	2.762	1.841	-1.172
O(26)	3.658	-1.138	0.037
H(27)	3.652	-1.570	0.907
C(28)	0.517	0.972	0.466
H(29)	0.600	1.331	1.506
H(30)	0.869	1.801	-0.163
C(31)	-0.955	0.760	0.246
C(32)	-1.861	1.754	-0.224
C(33)	-1.529	3.124	-0.720
H(34)	-0.484	3.240	-1.026
H(35)	-2.194	3.419	-1.541
H(36)	-1.715	3.821	0.113
C(37)	-2.511	-2.839	0.039
H(38)	-2.371	-3.662	-0.674
H(39)	-3.579	-2.596	0.047
H(40)	-2.237	-3.213	1.036
H(41)	-1.291	-0.847	1.637
H(42)	-1.203	0.819	-1.015
H(43)	-1.998	-1.238	-1.319

Intermediate E (–662,8329384 Hartree)

C(1)	-3.487	0.979	-0.424
H(2)	-4.384	1.431	0.025
H(3)	-3.518	1.299	-1.493
C(4)	-2.224	1.561	0.066
C(5)	-1.197	0.530	0.223
H(6)	-1.303	0.327	1.325
C(7)	-1.269	-2.203	1.565
H(8)	-2.264	-1.998	1.984
H(9)	-0.548	-1.530	2.055
H(10)	-0.996	-3.222	1.869
C(11)	-1.228	-2.087	0.030
C(12)	-1.777	-0.739	-0.489
H(13)	-1.533	-0.643	-1.562
C(14)	-3.303	-0.549	-0.330
H(15)	-3.879	-1.089	-1.090
H(16)	-3.642	-0.896	0.657
C(17)	0.181	-2.427	-0.546
H(18)	0.056	-3.065	-1.434
H(19)	0.715	-3.044	0.193
C(20)	1.092	-1.254	-0.956
H(21)	0.718	-0.789	-1.883
H(22)	2.071	-1.684	-1.205
C(23)	1.310	-0.152	0.108
H(24)	1.211	-0.602	1.113
C(25)	2.785	0.416	0.060
C(26)	3.129	1.066	-1.288
H(27)	2.979	0.367	-2.119
H(28)	4.182	1.369	-1.287
H(29)	2.518	1.962	-1.464
C(30)	3.058	1.394	1.220
H(31)	2.769	0.956	2.189
H(32)	4.130	1.623	1.251
H(33)	2.522	2.345	1.103
O(34)	3.679	-0.703	0.172
H(35)	3.648	-1.021	1.090
C(36)	0.269	0.976	-0.016
H(37)	0.324	1.405	-1.029
H(38)	0.501	1.787	0.687
C(39)	-2.014	2.999	0.289
H(40)	-2.944	3.573	0.342
H(41)	-1.421	3.373	-0.569
H(42)	-1.377	3.187	1.167
H(43)	-1.925	-2.849	-0.358

Intermediate E1 (–662,838907 Hartree)

C(1)	-3.504	0.929	-0.455
H(2)	-4.434	1.280	0.005
H(3)	-3.487	1.286	-1.497
C(4)	-2.254	1.480	0.283
C(5)	-1.182	0.501	-0.011
C(6)	-1.175	-2.062	1.635
H(7)	-2.184	-1.894	2.037
H(8)	-0.518	-1.281	2.042
H(9)	-0.826	-3.024	2.030
C(10)	-1.167	-2.093	0.093
C(11)	-1.766	-0.773	-0.486
H(12)	-1.500	-0.734	-1.571
C(13)	-3.305	-0.592	-0.382
H(14)	-3.830	-1.145	-1.168
H(15)	-3.656	-0.979	0.587
C(16)	0.208	-2.465	-0.522
H(17)	0.040	-3.086	-1.415
H(18)	0.738	-3.106	0.199
C(19)	1.138	-1.307	-0.933
H(20)	0.777	-0.839	-1.865
H(21)	2.119	-1.735	-1.171
C(22)	1.344	-0.219	0.140
H(23)	1.304	-0.692	1.136
C(24)	2.771	0.442	0.049
C(25)	3.030	1.109	-1.311
H(26)	2.933	0.391	-2.134
H(27)	4.050	1.508	-1.329
H(28)	2.344	1.950	-1.490
C(29)	3.009	1.443	1.196
H(30)	2.779	0.992	2.175
H(31)	4.063	1.745	1.195
H(32)	2.408	2.357	1.092
O(33)	3.736	-0.619	0.131
H(34)	3.780	-0.916	1.055
C(35)	0.235	0.852	0.094
H(36)	0.351	1.446	-0.849
H(37)	0.353	1.631	0.864
C(38)	-1.963	2.980	0.181
H(39)	-2.860	3.544	0.460
H(40)	-1.702	3.249	-0.852
H(41)	-1.148	3.302	0.841
H(42)	-1.886	-2.869	-0.217
H(43)	-2.403	1.217	1.367

Intermediate E2 (–662,8352998 Hartree)

C(1)	-3.501	0.960	-0.349
H(2)	-4.441	1.305	0.095
H(3)	-3.515	1.231	-1.417
C(4)	-2.249	1.555	0.330
C(5)	-1.142	0.511	-0.039
C(6)	-1.012	-2.393	1.458
H(7)	-1.965	-2.379	2.001
H(8)	-0.320	-1.690	1.929
H(9)	-0.589	-3.403	1.529
C(10)	-1.215	-2.110	-0.084
C(11)	-1.834	-0.780	-0.123
H(12)	-1.013	0.629	-1.157
C(13)	-3.298	-0.562	-0.203
H(14)	-3.797	-1.224	-0.927
H(15)	-3.663	-0.906	0.791
C(16)	0.104	-2.291	-0.912
H(17)	-0.190	-2.672	-1.900
H(18)	0.660	-3.101	-0.418
C(19)	1.035	-1.083	-1.109
H(20)	0.631	-0.411	-1.883
H(21)	1.977	-1.470	-1.520
C(22)	1.370	-0.299	0.173
H(23)	1.508	-1.039	0.981
C(24)	2.762	0.423	0.057
C(25)	2.823	1.411	-1.119
H(26)	2.683	0.893	-2.075
H(27)	3.809	1.890	-1.140
H(28)	2.066	2.202	-1.032
C(29)	3.130	1.119	1.380
H(30)	3.091	0.410	2.222
H(31)	4.150	1.518	1.312
H(32)	2.459	1.955	1.617
O(33)	3.751	-0.575	-0.254
H(34)	3.950	-1.061	0.563
C(35)	0.240	0.681	0.609
H(36)	0.541	1.710	0.375
H(37)	0.100	0.660	1.700
C(38)	-1.923	2.992	-0.079
H(39)	-2.770	3.652	0.147
H(40)	-1.723	3.060	-1.159
H(41)	-1.050	3.382	0.458
H(42)	-1.951	-2.854	-0.426
H(43)	-2.379	1.509	1.427

Intermediate E3 (–662,8369146 Hartree)

C(1)	-2.961	1.587	-0.545
H(2)	-3.845	2.133	-0.195
H(3)	-2.705	1.974	-1.545
C(4)	-1.751	1.738	0.391
C(5)	-0.757	0.652	-0.090
C(6)	-2.501	-2.447	0.906
H(7)	-3.469	-2.522	0.389
H(8)	-2.699	-1.780	1.766
H(9)	-2.184	-3.424	1.282
C(10)	-1.491	-1.791	0.048
C(11)	-1.761	-0.528	-0.631
H(12)	-0.244	1.030	-0.984
C(13)	-3.186	0.067	-0.594
H(14)	-3.786	-0.266	-1.451
H(15)	-3.718	-0.240	0.317
C(16)	-0.186	-2.462	-0.126
H(17)	-0.438	-3.273	-0.851
H(18)	0.055	-3.022	0.795
C(19)	1.002	-1.639	-0.634
H(20)	0.741	-1.129	-1.573
H(21)	1.829	-2.312	-0.882
C(22)	1.468	-0.627	0.433
H(23)	1.832	-1.219	1.293
C(24)	2.723	0.193	-0.051
C(25)	2.422	1.181	-1.188
H(26)	1.981	0.681	-2.060
H(27)	3.361	1.643	-1.515
H(28)	1.750	1.981	-0.852
C(29)	3.376	0.934	1.131
H(30)	3.619	0.237	1.949
H(31)	4.304	1.410	0.793
H(32)	2.729	1.719	1.543
O(33)	3.668	-0.741	-0.610
H(34)	4.137	-1.165	0.127
C(35)	0.284	0.229	0.958
H(36)	0.670	1.139	1.435
H(37)	-0.231	-0.315	1.771
C(38)	-1.122	3.137	0.393
H(39)	-1.856	3.880	0.733
H(40)	-0.798	3.423	-0.618
H(41)	-0.253	3.200	1.061
H(42)	-1.414	-0.679	-1.671
H(43)	-2.068	1.481	1.419

Intermediate E4 (–662,8460821 Hartree)

C(1)	3.298	1.470	-0.214
H(2)	4.138	1.994	0.254
C(3)	3.308	-0.044	0.075
C(4)	1.892	-0.472	0.083
C(5)	1.439	-1.859	0.233
H(6)	1.031	-1.827	1.274
C(7)	2.541	-2.927	0.162
H(8)	2.124	-3.901	0.440
H(9)	3.375	-2.716	0.843
H(10)	2.939	-3.014	-0.859
C(11)	0.185	-2.201	-0.660
C(12)	-1.171	-1.691	-0.156
H(13)	-1.958	-2.209	-0.718
C(14)	-1.415	-0.177	-0.297
H(15)	-1.266	0.086	-1.361
C(16)	-0.419	0.656	0.535
H(17)	-0.755	1.698	0.592
H(18)	-0.376	0.288	1.573
C(19)	1.011	0.699	-0.033
C(20)	1.919	1.929	0.307
C(21)	1.427	3.252	-0.279
H(22)	0.444	3.537	0.117
H(23)	1.350	3.194	-1.375
H(24)	2.126	4.061	-0.033
H(25)	1.957	2.008	1.409
H(26)	0.932	0.713	-1.158
C(27)	-2.904	0.200	-0.000
C(28)	-3.324	-0.097	1.453
H(29)	-4.377	0.179	1.601
H(30)	-3.220	-1.162	1.690
H(31)	-2.735	0.486	2.175
C(32)	-3.189	1.675	-0.352
H(33)	-2.846	1.910	-1.368
H(34)	-4.272	1.859	-0.309
H(35)	-2.727	2.379	0.354
O(36)	-3.637	-0.642	-0.911
H(37)	-4.582	-0.470	-0.776
H(38)	-1.290	-1.994	0.897
H(39)	0.159	-3.299	-0.695
H(40)	0.380	-1.863	-1.689
H(41)	3.957	-0.685	-0.541
H(42)	3.641	-0.244	1.119
H(43)	3.362	1.639	-1.301

Intermediate E5 (–662,8423316 Hartree)

C(1)	-3.497	-1.048	-0.048
H(2)	-3.829	-1.398	-1.035
H(3)	-4.289	-1.289	0.672
C(4)	-2.152	-1.727	0.330
C(5)	-1.093	-0.691	-0.123
H(6)	-1.096	-0.700	-1.231
C(7)	-1.618	3.146	0.610
H(8)	-2.204	2.981	1.519
H(9)	-2.229	3.713	-0.117
H(10)	-0.754	3.796	0.815
C(11)	-1.189	1.898	-0.063
C(12)	-1.738	0.616	0.365
C(13)	-3.256	0.492	-0.108
H(14)	-3.917	1.075	0.543
H(15)	-3.363	0.875	-1.132
C(16)	-0.164	1.964	-1.100
C(17)	1.131	1.462	-0.301
H(18)	1.091	1.807	0.745
H(19)	1.975	1.993	-0.765
C(20)	1.391	-0.051	-0.374
H(21)	1.338	-0.334	-1.443
C(22)	2.875	-0.342	0.059
C(23)	3.166	0.034	1.520
H(24)	3.021	1.106	1.698
H(25)	4.212	-0.199	1.751
H(26)	2.528	-0.531	2.212
C(27)	3.250	-1.811	-0.199
H(28)	3.000	-2.115	-1.227
H(29)	4.330	-1.936	-0.052
H(30)	2.739	-2.496	0.491
O(31)	3.732	0.520	-0.727
H(32)	3.832	0.117	-1.604
C(33)	0.342	-0.905	0.373
H(34)	0.385	-0.702	1.456
H(35)	0.599	-1.964	0.244
C(36)	-1.979	-3.126	-0.267
H(37)	-2.790	-3.790	0.058
H(38)	-1.033	-3.587	0.044
H(39)	-1.997	-3.088	-1.367
H(40)	-1.787	0.619	1.471
H(41)	-2.094	-1.804	1.432
H(42)	-0.009	2.977	-1.484
H(43)	-0.335	1.259	-1.923

Transition State E-TS1 (–662,8284842 Hartree)

C(1)	-3.565	0.870	-0.352
H(2)	-4.389	1.174	0.305
H(3)	-3.789	1.286	-1.352
C(4)	-2.242	1.486	0.028
C(5)	-1.188	0.539	-0.126
H(6)	-1.724	0.715	1.049
C(7)	-1.217	-1.912	1.671
H(8)	-2.232	-1.727	2.051
H(9)	-0.569	-1.103	2.042
H(10)	-0.867	-2.838	2.143
C(11)	-1.174	-2.055	0.136
C(12)	-1.757	-0.806	-0.579
H(13)	-1.510	-0.860	-1.656
C(14)	-3.296	-0.648	-0.412
H(15)	-3.846	-1.138	-1.222
H(16)	-3.621	-1.115	0.528
C(17)	0.225	-2.468	-0.400
H(18)	0.088	-3.155	-1.248
H(19)	0.731	-3.053	0.384
C(20)	1.168	-1.343	-0.867
H(21)	0.841	-0.959	-1.848
H(22)	2.157	-1.789	-1.028
C(23)	1.333	-0.159	0.110
H(24)	1.215	-0.528	1.144
C(25)	2.788	0.444	0.058
C(26)	3.164	0.977	-1.333
H(27)	3.070	0.199	-2.099
H(28)	4.208	1.312	-1.320
H(29)	2.540	1.835	-1.618
C(30)	2.994	1.535	1.127
H(31)	2.683	1.184	2.123
H(32)	4.058	1.796	1.172
H(33)	2.441	2.458	0.902
O(34)	3.710	-0.633	0.299
H(35)	3.650	-0.874	1.238
C(36)	0.270	0.931	-0.136
H(37)	0.418	1.336	-1.155
H(38)	0.418	1.786	0.537
C(39)	-2.098	2.944	0.330
H(40)	-2.941	3.311	0.927
H(41)	-2.121	3.478	-0.634
H(42)	-1.154	3.199	0.824
H(43)	-1.868	-2.874	-0.119

Transition State E-TS2 (–662,8309668 Hartree)

C(1)	3.536	-0.926	-0.288
H(2)	4.468	-1.274	0.170
H(3)	3.574	-1.188	-1.358
C(4)	2.282	-1.571	0.351
C(5)	1.197	-0.505	0.176
C(6)	0.959	2.533	1.387
H(7)	1.911	2.534	1.935
H(8)	0.262	1.871	1.913
H(9)	0.556	3.553	1.416
C(10)	1.154	2.116	-0.099
C(11)	1.806	0.755	-0.085
H(12)	1.335	-0.062	-1.059
C(13)	3.312	0.590	-0.131
H(14)	3.783	1.214	-0.901
H(15)	3.677	0.954	0.846
C(16)	-0.140	2.248	-0.965
H(17)	0.149	2.630	-1.955
H(18)	-0.753	3.033	-0.501
C(19)	-1.011	0.996	-1.167
H(20)	-0.555	0.319	-1.914
H(21)	-1.957	1.329	-1.614
C(22)	-1.331	0.220	0.125
H(23)	-1.366	0.942	0.959
C(24)	-2.766	-0.421	0.088
C(25)	-2.966	-1.387	-1.091
H(26)	-2.783	-0.889	-2.051
H(27)	-4.003	-1.741	-1.090
H(28)	-2.312	-2.266	-1.018
C(29)	-3.099	-1.118	1.420
H(30)	-2.945	-0.439	2.274
H(31)	-4.149	-1.432	1.415
H(32)	-2.485	-2.014	1.586
O(33)	-3.702	0.645	-0.151
H(34)	-3.809	1.141	0.677
C(35)	-0.246	-0.833	0.444
H(36)	-0.460	-1.761	-0.108
H(37)	-0.274	-1.128	1.507
C(38)	1.948	-2.975	-0.161
H(39)	2.814	-3.635	-0.024
H(40)	1.708	-2.957	-1.234
H(41)	1.104	-3.423	0.378
H(42)	1.900	2.806	-0.521
H(43)	2.424	-1.610	1.450

Transition State E-TS3 (–662,8317437 Hartree)

C(1)	-2.822	1.861	-0.376
H(2)	-3.589	2.554	-0.012
H(3)	-2.699	2.037	-1.457
C(4)	-1.470	2.024	0.340
C(5)	-0.717	0.696	0.015
C(6)	-2.936	-2.631	0.102
H(7)	-3.882	-2.121	-0.094
H(8)	-2.963	-3.004	1.138
H(9)	-2.846	-3.508	-0.549
C(10)	-1.717	-1.744	-0.004
C(11)	-1.849	-0.331	-0.091
H(12)	-0.273	0.821	-0.988
C(13)	-3.192	0.391	-0.117
H(14)	-3.909	-0.034	-0.831
H(15)	-3.632	0.272	0.888
C(16)	-0.379	-2.450	0.119
H(17)	-0.489	-3.435	-0.357
H(18)	-0.263	-2.660	1.199
C(19)	0.867	-1.729	-0.423
H(20)	0.632	-1.288	-1.405
H(21)	1.643	-2.475	-0.628
C(22)	1.455	-0.672	0.530
H(23)	1.815	-1.221	1.420
C(24)	2.735	0.007	-0.080
C(25)	2.433	0.921	-1.278
H(26)	1.931	0.377	-2.090
H(27)	3.377	1.308	-1.680
H(28)	1.815	1.778	-0.981
C(29)	3.508	0.790	0.997
H(30)	3.745	0.147	1.858
H(31)	4.449	1.163	0.572
H(32)	2.947	1.656	1.374
O(33)	3.581	-1.033	-0.611
H(34)	4.046	-1.444	0.135
C(35)	0.377	0.320	1.033
H(36)	0.850	1.251	1.371
H(37)	-0.119	-0.090	1.928
C(38)	-0.689	3.280	-0.053
H(39)	-1.260	4.182	0.201
H(40)	-0.493	3.299	-1.135
H(41)	0.274	3.341	0.470
H(42)	-1.727	-1.083	-1.168
H(43)	-1.655	2.039	1.430

Transition State E-TS5 (–662,7875937 Hartree)

C(1)	3.326	-1.420	0.172
H(2)	3.557	-2.005	1.073
H(3)	3.917	-1.817	-0.662
C(4)	1.816	-1.432	-0.102
C(5)	1.170	-0.518	0.939
H(6)	1.650	-0.647	1.918
C(7)	1.264	2.661	0.820
H(8)	2.162	2.703	1.451
H(9)	0.428	2.299	1.429
H(10)	1.036	3.684	0.495
C(11)	1.487	1.788	-0.444
C(12)	1.972	0.445	0.064
H(13)	1.867	-0.391	-0.985
C(14)	3.454	0.122	0.326
H(15)	4.122	0.571	-0.419
H(16)	3.757	0.468	1.323
C(17)	0.270	1.750	-1.411
H(18)	0.653	1.748	-2.442
H(19)	-0.272	2.700	-1.290
C(20)	-0.719	0.583	-1.262
H(21)	-0.275	-0.340	-1.667
H(22)	-1.576	0.805	-1.911
C(23)	-1.262	0.366	0.164
H(24)	-1.351	1.367	0.624
C(25)	-2.738	-0.182	0.135
C(26)	-2.864	-1.527	-0.600
H(27)	-2.508	-1.451	-1.634
H(28)	-3.920	-1.822	-0.633
H(29)	-2.312	-2.325	-0.086
C(30)	-3.328	-0.282	1.553
H(31)	-3.234	0.676	2.088
H(32)	-4.393	-0.537	1.487
H(33)	-2.836	-1.055	2.158
O(34)	-3.529	0.731	-0.649
H(35)	-3.689	1.522	-0.110
C(36)	-0.362	-0.488	1.101
H(37)	-0.691	-1.537	1.060
H(38)	-0.554	-0.174	2.135
C(39)	1.104	-2.531	-0.834
H(40)	1.609	-2.798	-1.768
H(41)	0.046	-2.325	-1.018
H(42)	1.158	-3.402	-0.157
H(43)	2.337	2.220	-0.995

Transition State E-TS6 (–662,8351363 Hartree)

C(1)	-3.372	-1.333	0.055
H(2)	-3.668	-1.372	-1.005
H(3)	-4.121	-1.891	0.629
C(4)	-1.946	-1.888	0.218
C(5)	-1.036	-0.709	-0.233
H(6)	-0.967	-0.724	-1.341
C(7)	-2.365	3.036	0.097
H(8)	-3.249	2.790	0.692
H(9)	-2.700	3.387	-0.892
H(10)	-1.830	3.870	0.566
C(11)	-1.433	1.871	-0.136
C(12)	-1.859	0.535	0.086
C(13)	-3.267	0.137	0.515
H(14)	-3.353	0.196	1.612
H(15)	-4.032	0.797	0.090
C(16)	-0.090	2.188	-0.771
C(17)	1.170	1.621	-0.082
H(18)	1.108	1.832	0.999
H(19)	2.036	2.178	-0.458
C(20)	1.442	0.125	-0.320
H(21)	1.381	-0.051	-1.413
C(22)	2.921	-0.240	0.076
C(23)	3.210	-0.025	1.569
H(24)	3.079	1.026	1.855
H(25)	4.250	-0.299	1.781
H(26)	2.558	-0.649	2.195
C(27)	3.272	-1.683	-0.334
H(28)	3.014	-1.874	-1.388
H(29)	4.351	-1.837	-0.208
H(30)	2.759	-2.432	0.282
O(31)	3.799	0.675	-0.611
H(32)	3.874	0.377	-1.532
C(33)	0.393	-0.779	0.346
H(34)	0.344	-0.571	1.429
H(35)	0.703	-1.828	0.255
C(36)	-1.689	-3.192	-0.541
H(37)	-2.361	-3.984	-0.187
H(38)	-0.660	-3.550	-0.406
H(39)	-1.865	-3.059	-1.619
H(40)	-1.243	1.253	1.025
H(41)	-1.757	-2.054	1.295
H(42)	-0.007	3.281	-0.830
H(43)	-0.141	1.815	-1.809

Intermediate H (–662,8338254 Hartree)

C(1)	-3.160	-1.299	-0.776
H(2)	-4.119	-1.789	-0.559
H(3)	-2.798	-1.769	-1.721
C(4)	-3.137	0.239	-0.918
H(5)	-3.783	0.681	-0.146
H(6)	-3.509	0.574	-1.893
C(7)	-1.651	0.607	-0.686
C(8)	-1.394	2.054	-0.197
C(9)	0.107	2.419	-0.266
H(10)	0.226	3.431	0.150
H(11)	0.413	2.486	-1.323
C(12)	1.087	1.472	0.457
H(13)	2.053	1.987	0.516
H(14)	0.771	1.296	1.499
C(15)	1.325	0.128	-0.269
H(16)	1.190	0.301	-1.353
C(17)	2.829	-0.366	-0.127
C(18)	3.077	-1.662	-0.919
H(19)	4.153	-1.877	-0.933
H(20)	2.729	-1.569	-1.960
H(21)	2.571	-2.525	-0.466
C(22)	3.266	-0.526	1.336
H(23)	4.327	-0.802	1.365
H(24)	3.145	0.410	1.894
H(25)	2.697	-1.316	1.842
O(26)	3.655	0.682	-0.652
H(27)	3.608	0.649	-1.622
C(28)	0.353	-0.998	0.138
H(29)	0.381	-1.800	-0.613
H(30)	0.658	-1.439	1.096
C(31)	-1.139	-0.494	0.289
C(32)	-2.092	-1.600	0.197
C(33)	-2.019	-2.858	0.957
H(34)	-2.229	-3.725	0.311
H(35)	-1.084	-2.998	1.505
H(36)	-2.857	-2.846	1.681
H(37)	-1.215	-0.129	1.338
C(38)	-1.978	2.349	1.197
H(39)	-1.443	1.819	2.001
H(40)	-3.042	2.092	1.280
H(41)	-1.884	3.419	1.418
H(42)	-1.120	0.471	-1.645
H(43)	-1.917	2.712	-0.912

Intermediate H1 (–662,8424273 Hartree)

C(1)	3.322	1.263	-0.503
H(2)	4.243	1.713	-0.117
H(3)	3.174	1.628	-1.532
C(4)	3.307	-0.272	-0.457
H(5)	3.756	-0.641	0.478
H(6)	3.833	-0.747	-1.292
C(7)	1.791	-0.610	-0.468
C(8)	1.353	-2.072	-0.194
C(9)	-0.048	-2.401	-0.756
H(10)	-0.197	-3.486	-0.653
H(11)	-0.049	-2.195	-1.840
C(12)	-1.251	-1.694	-0.112
H(13)	-2.166	-2.092	-0.568
H(14)	-1.306	-1.943	0.959
C(15)	-1.288	-0.161	-0.284
H(16)	-1.012	0.084	-1.328
C(17)	-2.734	0.437	-0.075
C(18)	-2.792	1.935	-0.430
H(19)	-3.840	2.257	-0.444
H(20)	-2.354	2.128	-1.422
H(21)	-2.269	2.568	0.299
C(22)	-3.272	0.184	1.340
H(23)	-4.275	0.615	1.431
H(24)	-3.346	-0.889	1.547
H(25)	-2.633	0.651	2.102
O(26)	-3.606	-0.289	-0.955
H(27)	-3.501	0.069	-1.852
C(28)	-0.256	0.557	0.657
H(29)	-0.551	1.580	0.911
H(30)	-0.257	-0.012	1.609
C(31)	1.134	0.525	0.202
C(32)	2.086	1.655	0.350
C(33)	1.564	3.094	0.221
H(34)	1.148	3.261	-0.781
H(35)	0.796	3.333	0.966
H(36)	2.396	3.793	0.364
C(37)	1.527	-2.458	1.286
H(38)	0.860	-1.895	1.954
H(39)	2.557	-2.299	1.630
H(40)	1.293	-3.522	1.422
H(41)	1.445	-0.356	-1.511
H(42)	2.070	-2.678	-0.774
H(43)	2.380	1.484	1.420

Intermediate H2 (–662,8425933 Hartree)

C(1)	3.533	1.066	-0.187
H(2)	4.409	1.440	0.354
H(3)	3.661	1.321	-1.251
C(4)	3.353	-0.459	-0.038
H(5)	3.556	-0.748	1.019
H(6)	3.985	-1.122	-0.647
C(7)	1.899	-0.712	-0.177
C(8)	1.325	-2.056	-0.318
C(9)	-0.042	-2.171	-1.037
H(10)	-0.203	-3.251	-1.176
H(11)	0.063	-1.746	-2.049
C(12)	-1.274	-1.574	-0.341
H(13)	-2.169	-1.968	-0.838
H(14)	-1.325	-1.932	0.698
C(15)	-1.371	-0.038	-0.370
H(16)	-1.239	0.286	-1.422
C(17)	-2.812	0.451	0.036
C(18)	-2.978	1.966	-0.191
H(19)	-4.036	2.230	-0.071
H(20)	-2.661	2.256	-1.205
H(21)	-2.408	2.568	0.529
C(22)	-3.178	0.085	1.483
H(23)	-4.186	0.451	1.707
H(24)	-3.177	-1.002	1.630
H(25)	-2.482	0.542	2.199
O(26)	-3.766	-0.254	-0.781
H(27)	-3.752	0.146	-1.665
C(28)	-0.262	0.640	0.456
H(29)	-0.474	1.711	0.555
H(30)	-0.240	0.235	1.480
C(31)	1.163	0.560	-0.130
C(32)	2.198	1.635	0.336
C(33)	1.888	3.055	-0.138
H(34)	1.838	3.103	-1.235
H(35)	0.936	3.425	0.264
H(36)	2.673	3.747	0.193
C(37)	1.271	-2.611	1.166
H(38)	0.723	-1.945	1.841
H(39)	2.277	-2.781	1.562
H(40)	0.737	-3.568	1.119
H(41)	1.073	0.695	-1.248
H(42)	2.065	-2.690	-0.835
H(43)	2.207	1.611	1.442

Intermediate H3 (–662,8411722 Hartree)

C(1)	3.515	0.940	-0.340
H(2)	4.429	1.209	0.205
H(3)	3.570	1.416	-1.332
C(4)	3.341	-0.580	-0.478
H(5)	3.641	-1.076	0.455
H(6)	3.944	-1.021	-1.283
C(7)	1.833	-0.809	-0.769
C(8)	1.105	-1.818	-0.025
C(9)	-0.119	-2.410	-0.590
H(10)	-0.219	-3.457	-0.267
H(11)	-0.136	-2.349	-1.685
C(12)	-1.363	-1.630	-0.008
H(13)	-2.245	-2.114	-0.445
H(14)	-1.407	-1.785	1.079
C(15)	-1.387	-0.133	-0.340
H(16)	-1.238	-0.026	-1.432
C(17)	-2.815	0.461	-0.046
C(18)	-2.957	1.878	-0.631
H(19)	-3.996	2.212	-0.517
H(20)	-2.702	1.892	-1.702
H(21)	-2.317	2.612	-0.123
C(22)	-3.158	0.457	1.451
H(23)	-4.154	0.890	1.598
H(24)	-3.178	-0.564	1.853
H(25)	-2.437	1.051	2.028
O(26)	-3.792	-0.405	-0.662
H(27)	-3.834	-0.182	-1.606
C(28)	-0.246	0.633	0.375
H(29)	-0.521	1.691	0.475
H(30)	-0.130	0.261	1.406
C(31)	1.103	0.640	-0.353
C(32)	2.236	1.394	0.377
C(33)	2.016	2.914	0.379
H(34)	1.915	3.299	-0.647
H(35)	1.123	3.205	0.946
H(36)	2.877	3.414	0.841
C(37)	1.495	-2.196	1.353
H(38)	0.681	-2.668	1.912
H(39)	1.934	-1.364	1.917
H(40)	2.303	-2.948	1.252
H(41)	0.946	1.094	-1.345
H(42)	1.633	-0.929	-1.841
H(43)	2.270	1.040	1.425

Intermediate H4 (–662,8423239 Hartree)

C(1)	3.141	1.272	-0.680
H(2)	3.898	1.363	0.115
H(3)	3.490	1.854	-1.541
C(4)	2.921	-0.216	-1.018
H(5)	3.801	-0.876	-1.049
H(6)	2.456	-0.314	-2.026
C(7)	1.864	-0.691	-0.098
C(8)	1.471	-2.096	0.015
C(9)	0.151	-2.232	-0.886
H(10)	0.041	-3.323	-0.985
H(11)	0.369	-1.845	-1.894
C(12)	-1.138	-1.626	-0.339
H(13)	-1.950	-1.924	-1.018
H(14)	-1.375	-2.073	0.637
C(15)	-1.163	-0.084	-0.232
H(16)	-0.761	0.327	-1.181
C(17)	-2.642	0.449	-0.138
C(18)	-2.694	1.982	-0.276
H(19)	-3.740	2.302	-0.353
H(20)	-2.159	2.320	-1.178
H(21)	-2.255	2.497	0.589
C(22)	-3.353	-0.005	1.144
H(23)	-4.377	0.386	1.151
H(24)	-3.412	-1.098	1.200
H(25)	-2.837	0.369	2.038
O(26)	-3.391	-0.144	-1.216
H(27)	-3.160	0.326	-2.034
C(28)	-0.275	0.422	0.920
H(29)	-0.529	1.456	1.186
H(30)	-0.469	-0.172	1.823
C(31)	1.238	0.454	0.591
C(32)	1.764	1.725	-0.156
C(33)	1.796	2.989	0.704
H(34)	0.793	3.279	1.044
H(35)	2.432	2.848	1.590
H(36)	2.203	3.832	0.130
H(37)	1.796	0.415	1.565
C(38)	1.246	-2.620	1.450
H(39)	0.506	-2.040	2.009
H(40)	2.190	-2.611	2.009
H(41)	0.891	-3.656	1.394
H(42)	2.232	-2.717	-0.480
H(43)	1.091	1.891	-1.018

Intermediate H5 (–662,8374439 Hartree)

C(1)	-3.598	-0.603	-0.134
H(2)	-4.070	-0.794	0.840
H(3)	-4.356	-0.777	-0.909
C(4)	-3.101	0.878	-0.176
H(5)	-3.248	1.377	0.791
H(6)	-3.574	1.494	-0.949
C(7)	-1.537	0.715	-0.482
C(8)	-0.827	1.890	0.004
C(9)	-0.166	1.839	1.317
C(10)	1.149	0.940	1.196
H(11)	1.997	1.635	1.176
H(12)	1.210	0.343	2.114
C(13)	1.280	0.035	-0.052
H(14)	1.120	0.675	-0.946
C(15)	2.767	-0.455	-0.193
C(16)	2.957	-1.287	-1.472
H(17)	4.026	-1.479	-1.624
H(18)	2.569	-0.757	-2.357
H(19)	2.449	-2.258	-1.410
C(20)	3.257	-1.241	1.034
H(21)	4.283	-1.583	0.856
H(22)	3.270	-0.614	1.933
H(23)	2.630	-2.121	1.226
O(24)	3.606	0.720	-0.247
H(25)	3.640	1.017	-1.170
C(26)	0.235	-1.103	-0.125
H(27)	0.265	-1.555	-1.127
H(28)	0.500	-1.896	0.589
C(29)	-1.195	-0.635	0.173
C(30)	-2.362	-1.522	-0.317
C(31)	-2.473	-2.874	0.391
H(32)	-1.578	-3.488	0.221
H(33)	-2.596	-2.741	1.476
H(34)	-3.338	-3.441	0.023
H(35)	-1.319	-0.533	1.267
C(36)	-0.749	3.122	-0.807
H(37)	-1.239	3.938	-0.245
H(38)	-1.195	3.032	-1.802
H(39)	0.303	3.444	-0.884
H(40)	-1.456	0.661	-1.582
H(41)	-2.212	-1.701	-1.398
H(42)	-0.842	1.347	2.033
H(43)	0.092	2.834	1.694

Transition State H-TS1 (–662,8326182 Hartree)

C(1)	3.348	1.163	-0.626
H(2)	4.273	1.550	-0.181
H(3)	3.260	1.614	-1.632
C(4)	3.233	-0.374	-0.689
H(5)	3.733	-0.816	0.187
H(6)	3.701	-0.800	-1.583
C(7)	1.712	-0.664	-0.628
C(8)	1.316	-2.069	-0.113
C(9)	-0.122	-2.455	-0.528
H(10)	-0.274	-3.513	-0.268
H(11)	-0.193	-2.397	-1.627
C(12)	-1.271	-1.643	0.090
H(13)	-2.216	-2.076	-0.260
H(14)	-1.265	-1.760	1.186
C(15)	-1.302	-0.138	-0.261
H(16)	-1.036	-0.019	-1.329
C(17)	-2.757	0.461	-0.129
C(18)	-2.832	1.905	-0.664
H(19)	-3.885	2.210	-0.715
H(20)	-2.401	1.979	-1.674
H(21)	-2.315	2.629	-0.019
C(22)	-3.294	0.386	1.308
H(23)	-4.314	0.787	1.337
H(24)	-3.329	-0.651	1.663
H(25)	-2.677	0.977	1.998
O(26)	-3.641	-0.367	-0.903
H(27)	-3.482	-0.179	-1.842
C(28)	-0.287	0.676	0.588
H(29)	-0.547	1.741	0.595
H(30)	-0.369	0.328	1.629
C(31)	1.147	0.537	0.127
C(32)	2.102	1.598	0.109
C(33)	1.895	2.994	0.600
H(34)	1.690	3.622	-0.283
H(35)	1.049	3.093	1.288
H(36)	2.804	3.389	1.070
H(37)	1.946	0.682	1.134
C(38)	1.575	-2.247	1.394
H(39)	0.954	-1.580	2.010
H(40)	2.626	-2.068	1.660
H(41)	1.336	-3.273	1.700
H(42)	1.297	-0.541	-1.649
H(43)	1.990	-2.764	-0.644

Transition State H-TS2 (–662,8359648 Hartree)

C(1)	3.400	1.250	-0.348
H(2)	4.309	1.677	0.088
H(3)	3.348	1.583	-1.398
C(4)	3.362	-0.289	-0.289
H(5)	3.823	-0.668	0.642
H(6)	3.858	-0.800	-1.123
C(7)	1.891	-0.632	-0.175
C(8)	1.390	-2.053	-0.266
C(9)	0.016	-2.232	-0.956
H(10)	-0.134	-3.317	-1.051
H(11)	0.090	-1.846	-1.987
C(12)	-1.216	-1.628	-0.268
H(13)	-2.111	-2.016	-0.770
H(14)	-1.274	-1.978	0.775
C(15)	-1.309	-0.091	-0.305
H(16)	-1.077	0.244	-1.337
C(17)	-2.777	0.410	-0.025
C(18)	-2.915	1.928	-0.254
H(19)	-3.979	2.194	-0.232
H(20)	-2.504	2.225	-1.231
H(21)	-2.416	2.524	0.521
C(22)	-3.270	0.029	1.378
H(23)	-4.295	0.394	1.515
H(24)	-3.282	-1.059	1.513
H(25)	-2.643	0.478	2.160
O(26)	-3.650	-0.284	-0.933
H(27)	-3.563	0.129	-1.808
C(28)	-0.283	0.590	0.637
H(29)	-0.539	1.644	0.780
H(30)	-0.337	0.116	1.632
C(31)	1.161	0.524	0.223
C(32)	2.116	1.706	0.389
C(33)	1.584	3.076	-0.047
H(34)	1.284	3.062	-1.105
H(35)	0.728	3.406	0.553
H(36)	2.373	3.830	0.067
C(37)	1.436	-2.655	1.165
H(38)	0.770	-2.125	1.856
H(39)	2.451	-2.635	1.581
H(40)	1.111	-3.702	1.117
H(41)	1.270	0.177	-1.056
H(42)	2.129	-2.599	-0.873
H(43)	2.312	1.723	1.481

Transition State H-TS3 (–662,8348043 Hartree)

C(1)	-3.413	-1.122	0.363
H(2)	-4.062	-1.653	1.068
H(3)	-3.955	-1.044	-0.594
C(4)	-3.028	0.285	0.859
H(5)	-2.692	0.245	1.911
H(6)	-3.823	1.036	0.799
C(7)	-1.799	0.605	0.031
C(8)	-1.308	1.899	-0.288
C(9)	-0.016	2.053	-1.070
H(10)	0.103	3.114	-1.325
H(11)	-0.077	1.502	-2.021
C(12)	1.206	1.543	-0.266
H(13)	2.101	2.044	-0.655
H(14)	1.104	1.851	0.787
C(15)	1.439	0.023	-0.363
H(16)	1.439	-0.238	-1.441
C(17)	2.878	-0.352	0.152
C(18)	3.203	-1.833	-0.117
H(19)	4.266	-2.009	0.091
H(20)	3.005	-2.103	-1.166
H(21)	2.626	-2.512	0.524
C(22)	3.087	-0.022	1.638
H(23)	4.104	-0.303	1.932
H(24)	2.971	1.052	1.829
H(25)	2.382	-0.574	2.272
O(26)	3.825	0.479	-0.552
H(27)	3.942	0.106	-1.440
C(28)	0.323	-0.799	0.303
H(29)	0.580	-1.866	0.265
H(30)	0.246	-0.540	1.371
C(31)	-1.086	-0.693	-0.338
C(32)	-2.053	-1.821	0.155
C(33)	-2.094	-3.036	-0.774
H(34)	-2.471	-2.759	-1.770
H(35)	-1.095	-3.477	-0.901
H(36)	-2.752	-3.817	-0.371
C(37)	-1.929	3.152	0.269
H(38)	-1.209	3.551	1.002
H(39)	-2.882	2.996	0.782
H(40)	-2.041	3.917	-0.510
H(41)	-0.976	-0.755	-1.436
H(42)	-2.196	1.246	-1.046
H(43)	-1.688	-2.143	1.146

Transition State H-TS5 (–662,795824 Hartree)

C(1)	-3.213	-1.641	0.088
H(2)	-3.426	-2.235	0.987
H(3)	-3.747	-2.089	-0.758
C(4)	-3.461	-0.115	0.223
H(5)	-3.813	0.212	1.211
H(6)	-4.146	0.277	-0.539
C(7)	-2.003	0.325	-0.005
C(8)	-1.643	1.705	-0.514
C(9)	-0.304	1.848	-1.268
H(10)	-0.276	2.883	-1.640
H(11)	-0.337	1.206	-2.165
C(12)	0.984	1.564	-0.486
H(13)	1.830	1.897	-1.099
H(14)	1.011	2.169	0.434
C(15)	1.215	0.078	-0.150
H(16)	0.930	-0.502	-1.050
C(17)	2.748	-0.228	0.064
C(18)	3.022	-1.743	0.141
H(19)	4.106	-1.910	0.169
H(20)	2.623	-2.264	-0.745
H(21)	2.592	-2.213	1.035
C(22)	3.323	0.487	1.295
H(23)	4.397	0.281	1.366
H(24)	3.194	1.574	1.218
H(25)	2.846	0.138	2.220
O(26)	3.476	0.313	-1.054
H(27)	3.323	-0.266	-1.818
C(28)	0.365	-0.391	1.054
H(29)	0.736	-1.355	1.431
H(30)	0.513	0.324	1.876
C(31)	-1.149	-0.571	0.891
C(32)	-1.700	-1.551	-0.141
C(33)	-0.906	-2.622	-0.827
H(34)	0.136	-2.352	-1.011
H(35)	-0.905	-3.466	-0.116
H(36)	-1.385	-2.960	-1.753
H(37)	-1.619	-0.720	1.874
C(38)	-1.749	2.641	0.721
H(39)	-1.021	2.380	1.497
H(40)	-2.753	2.622	1.164
H(41)	-1.545	3.668	0.392
H(42)	-1.797	-0.510	-1.043
H(43)	-2.444	1.984	-1.217

Transition State H-TS6 (–662,8288225 Hartree)

C(1)	3.116	-1.400	0.481
H(2)	3.761	-1.600	-0.388
H(3)	3.542	-1.937	1.337
C(4)	3.031	0.123	0.730
H(5)	3.908	0.683	0.377
H(6)	2.927	0.348	1.803
C(7)	1.779	0.560	-0.025
C(8)	1.328	1.898	-0.142
C(9)	0.114	2.219	-0.995
C(10)	-1.227	1.715	-0.396
H(11)	-1.529	2.352	0.445
H(12)	-1.988	1.860	-1.173
C(13)	-1.215	0.235	0.055
H(14)	-0.727	0.177	1.053
C(15)	-2.673	-0.293	0.319
C(16)	-2.641	-1.613	1.113
H(17)	-3.667	-1.921	1.351
H(18)	-2.087	-1.493	2.057
H(19)	-2.171	-2.430	0.550
C(20)	-3.479	-0.469	-0.977
H(21)	-4.486	-0.822	-0.728
H(22)	-3.586	0.480	-1.517
H(23)	-3.011	-1.203	-1.644
O(24)	-3.378	0.708	1.077
H(25)	-3.146	0.593	2.012
C(26)	-0.406	-0.639	-0.926
H(27)	-0.731	-1.684	-0.856
H(28)	-0.632	-0.331	-1.956
C(29)	1.131	-0.645	-0.691
C(30)	1.673	-1.834	0.156
C(31)	1.578	-3.194	-0.536
H(32)	0.539	-3.486	-0.737
H(33)	2.119	-3.184	-1.494
H(34)	2.021	-3.977	0.092
H(35)	1.640	-0.696	-1.676
C(36)	2.085	3.044	0.478
H(37)	2.616	3.558	-0.340
H(38)	2.825	2.739	1.223
H(39)	1.393	3.774	0.916
H(40)	0.915	1.082	0.829
H(41)	1.088	-1.865	1.096
H(42)	0.274	1.774	-1.988
H(43)	0.061	3.306	-1.128

Computational Results (water mediated proton transfer, Figure 2 of main text)

Intermediate 2* (–814.857492 Hartree)

C(1)	1.844	1.488	-0.351
C(2)	2.918	1.173	0.661
C(3)	-1.144	-0.702	-1.174
C(4)	-0.194	-1.931	-1.353
C(5)	1.746	-1.913	1.991
C(6)	0.619	0.929	-0.164
C(7)	-3.064	-2.138	-0.326
C(8)	2.290	2.143	-1.632
C(9)	-2.260	-0.856	-0.100
C(10)	-1.865	-0.719	1.372
C(11)	3.622	-0.188	0.259
C(12)	0.561	-2.615	-0.183
C(13)	2.616	-1.188	-0.250
C(14)	1.707	-1.859	0.483
C(15)	-0.446	0.690	-1.206
H(16)	4.169	-0.549	1.140
H(17)	-3.941	-2.172	0.338
O(18)	-3.201	0.297	-0.384
H(19)	-4.114	-0.033	-0.353
H(20)	-1.154	-1.498	1.657
O(21)	-2.893	2.429	0.819
H(22)	-3.199	2.378	1.738
H(23)	0.954	-3.560	-0.591
H(24)	1.463	2.382	-2.310
H(25)	2.844	3.069	-1.424
H(26)	4.371	0.014	-0.517
H(27)	0.921	-1.337	2.442
H(28)	3.675	1.965	0.734
H(29)	2.681	-1.523	2.404
H(30)	-1.236	1.457	-1.181
H(31)	2.988	1.482	-2.170
H(32)	-0.155	-2.933	0.589
H(33)	0.001	0.785	-2.204
H(34)	1.629	-2.948	2.344
H(35)	2.510	-1.219	-1.337
H(36)	0.467	0.444	0.799
H(37)	2.469	1.041	1.654
H(38)	-0.510	4.147	0.287
H(39)	-1.402	0.249	1.586
O(40)	-0.621	3.349	0.828
H(41)	-2.753	-0.828	2.009
H(42)	-2.453	-3.016	-0.092
H(43)	-1.728	-0.698	-2.108

Intermediate D* (–814.869422 Hartree)

C(1)	-0.472	-2.436	0.262
C(2)	-0.259	0.324	-1.499
C(3)	1.633	-1.235	-0.495
C(4)	-1.258	-1.992	-0.994
C(5)	1.265	-1.772	1.971
C(6)	-3.990	-0.322	-0.551
C(7)	3.135	-0.949	-0.285
C(8)	-2.316	-0.046	1.296
C(9)	-1.548	-0.488	-1.210
C(10)	0.852	-1.826	0.564
C(11)	0.881	0.244	-0.497
C(12)	-2.570	0.158	-0.208
C(13)	3.219	0.539	0.105
C(14)	2.465	1.397	-2.188
C(15)	2.095	1.199	-0.712
H(16)	1.398	-1.680	-1.467
H(17)	4.205	0.971	-0.105
H(18)	0.110	0.006	-2.484
H(19)	0.508	0.459	0.507
H(20)	-0.531	1.381	-1.600
H(21)	-2.087	-0.455	-2.168
H(22)	-1.300	0.210	1.611
H(23)	0.981	-0.749	2.338
H(24)	3.652	-1.131	-1.234
H(25)	1.800	2.164	-0.277
H(26)	-0.547	3.762	1.008
H(27)	-4.726	0.133	0.127
H(28)	1.666	1.903	-2.742
H(29)	2.347	-1.844	2.113
H(30)	0.725	-2.497	2.590
O(31)	0.739	1.140	2.761
H(32)	3.016	0.669	1.176
H(33)	-1.220	2.456	0.459
O(34)	-2.502	1.598	-0.486
H(35)	2.681	0.450	-2.700
H(36)	-3.004	0.599	1.858
H(37)	0.359	1.259	3.643
H(38)	-1.091	-2.411	1.165
H(39)	3.364	2.022	-2.258
H(40)	-4.250	-0.056	-1.583
O(41)	-0.439	2.803	0.952
H(42)	3.594	-1.619	0.452
H(43)	-0.215	-3.510	0.144

Intermediate D4* (–814.875086 Hartree)

C(1)	-1.038	-0.118	-0.728
H(2)	-0.277	1.473	1.395
C(3)	-2.079	-1.075	-0.577
C(4)	0.269	-0.500	-1.313
H(5)	0.028	-0.454	-2.397
H(6)	0.445	-1.568	-1.148
C(7)	-3.393	-0.317	-0.343
H(8)	-3.877	-0.137	-1.315
H(9)	-4.098	-0.882	0.277
C(10)	-2.913	0.986	0.306
H(11)	-3.596	1.828	0.165
H(12)	-2.785	0.832	1.387
C(13)	-2.101	-2.418	-1.298
H(14)	-1.177	-2.988	-1.153
H(15)	-2.934	-3.021	-0.920
H(16)	-2.258	-2.265	-2.374
C(17)	-1.514	1.253	-0.320
H(18)	-1.661	1.767	-1.294
C(19)	-0.574	2.119	0.559
C(20)	-1.332	3.320	1.157
H(21)	-1.766	3.945	0.363
H(22)	-2.136	3.018	1.836
H(23)	-0.636	3.946	1.730
C(24)	0.693	2.648	-0.155
H(25)	1.445	2.854	0.620
H(26)	0.453	3.631	-0.587
C(27)	1.326	1.829	-1.293
H(28)	0.704	1.937	-2.195
H(29)	2.279	2.310	-1.552
C(30)	1.568	0.316	-1.120
H(31)	2.180	0.020	-1.984
C(32)	2.454	-0.140	0.079
C(33)	3.810	0.577	0.026
H(34)	3.697	1.648	0.229
H(35)	4.485	0.174	0.794
H(36)	4.282	0.455	-0.957
C(37)	1.846	-0.030	1.480
H(38)	0.852	-0.484	1.543
H(39)	2.493	-0.552	2.197
H(40)	1.777	1.012	1.807
O(41)	2.686	-1.575	-0.192
H(42)	1.489	-2.694	0.417
H(43)	3.542	-1.801	0.204

Intermediate 3* (–814.886921 Hartree)

C(1)	1.097	0.286	-0.723
H(2)	0.522	-1.347	1.416
C(3)	2.006	1.306	-0.699
C(4)	-0.243	0.427	-1.410
H(5)	-0.022	0.326	-2.487
H(6)	-0.603	1.459	-1.315
C(7)	3.370	0.818	-0.257
H(8)	4.014	0.706	-1.147
H(9)	3.891	1.519	0.412
C(10)	3.060	-0.539	0.393
H(11)	3.880	-1.259	0.314
H(12)	2.856	-0.386	1.464
C(13)	1.840	2.703	-1.235
H(14)	0.794	3.021	-1.316
H(15)	2.379	3.434	-0.616
H(16)	2.279	2.771	-2.243
C(17)	1.756	-1.033	-0.291
H(18)	2.021	-1.583	-1.215
C(19)	0.930	-1.977	0.612
C(20)	1.815	-3.047	1.282
H(21)	2.356	-3.631	0.524
H(22)	2.552	-2.614	1.968
H(23)	1.196	-3.745	1.860
C(24)	-0.234	-2.707	-0.102
H(25)	-0.962	-3.016	0.664
H(26)	0.160	-3.652	-0.507
C(27)	-0.960	-2.026	-1.275
H(28)	-0.306	-2.063	-2.159
H(29)	-1.825	-2.652	-1.538
C(30)	-1.418	-0.554	-1.169
H(31)	-2.057	-0.395	-2.052
C(32)	-2.393	-0.224	-0.006
C(33)	-3.673	-1.055	-0.120
H(34)	-3.453	-2.110	0.075
H(35)	-4.415	-0.738	0.627
H(36)	-4.113	-0.978	-1.122
C(37)	-1.860	-0.260	1.424
H(38)	-0.923	0.288	1.544
H(39)	-2.607	0.163	2.110
H(40)	-1.684	-1.294	1.734
O(41)	-2.799	1.219	-0.274
H(42)	-2.136	2.180	0.376
H(43)	-3.762	1.288	-0.170

Transition State D-TS0* (–814.848246 Hartree)

C(1)	3.050	0.395	0.563
C(2)	1.201	-2.239	1.978
C(3)	-3.755	-1.077	-0.175
C(4)	-1.469	-0.521	-1.117
C(5)	-2.530	-0.167	-0.027
C(6)	0.749	0.495	-0.219
C(7)	1.018	-2.184	0.484
C(8)	-0.317	-2.714	-0.018
C(9)	-0.317	0.505	-1.260
C(10)	1.984	-1.727	-0.358
C(11)	3.282	-1.079	0.051
C(12)	-2.071	-0.137	1.440
C(13)	-0.997	-2.000	-1.208
C(14)	2.529	1.467	-1.733
C(15)	2.027	1.047	-0.358
H(16)	4.000	0.944	0.586
H(17)	3.966	-1.057	-0.805
H(18)	2.732	0.589	-2.366
H(19)	2.222	-2.014	2.300
H(20)	-2.039	-0.375	-2.047
H(21)	1.549	2.221	0.285
H(22)	0.149	0.353	-2.244
H(23)	1.806	2.096	-2.266
H(24)	-1.982	2.494	0.339
H(25)	2.664	0.355	1.590
H(26)	0.459	0.225	0.794
H(27)	-0.755	1.515	-1.308
H(28)	3.467	2.028	-1.645
H(29)	3.788	-1.642	0.846
H(30)	-1.323	0.644	1.612
H(31)	-4.119	-1.089	-1.209
H(32)	-1.013	-2.769	0.829
H(33)	-1.663	-1.098	1.765
H(34)	0.104	3.268	0.806
H(35)	-3.865	1.316	-0.142
O(36)	-2.929	1.214	-0.373
H(37)	-2.932	0.088	2.084
H(38)	1.432	4.082	0.380
H(39)	0.936	-3.239	2.350
H(40)	-1.871	-2.609	-1.477
O(41)	1.132	3.293	0.860
H(42)	-1.872	3.463	1.572
H(43)	1.788	-1.776	-1.431

Transition State D-TS5* (–814.851256 Hartree)

C(1)	1.097	0.103	-0.509
H(2)	0.763	-0.186	0.806
C(3)	2.105	1.255	-0.289
C(4)	-0.269	0.407	-1.098
H(5)	-0.038	0.620	-2.151
H(6)	-0.605	1.356	-0.681
C(7)	3.498	0.575	-0.188
H(8)	4.262	1.175	-0.697
H(9)	3.817	0.500	0.857
C(10)	3.353	-0.823	-0.835
H(11)	3.654	-0.798	-1.890
H(12)	3.972	-1.585	-0.345
C(13)	2.031	2.232	-1.482
H(14)	1.063	2.739	-1.542
H(15)	2.802	2.999	-1.345
H(16)	2.229	1.725	-2.437
C(17)	1.862	-1.157	-0.777
H(18)	1.486	-1.822	-1.553
C(19)	1.197	-1.351	0.579
C(20)	2.052	-1.525	1.831
H(21)	2.605	-2.468	1.728
H(22)	2.765	-0.716	1.986
H(23)	1.407	-1.588	2.713
C(24)	-0.073	-2.204	0.567
H(25)	-0.630	-1.993	1.485
H(26)	0.275	-3.245	0.646
C(27)	-0.971	-2.074	-0.674
H(28)	-0.458	-2.518	-1.537
H(29)	-1.852	-2.708	-0.515
C(30)	-1.426	-0.647	-1.062
H(31)	-1.766	-0.719	-2.103
C(32)	-2.711	-0.168	-0.297
C(33)	-3.944	-0.880	-0.875
H(34)	-3.879	-1.967	-0.753
H(35)	-4.854	-0.555	-0.351
H(36)	-4.055	-0.658	-1.944
C(37)	-2.670	-0.332	1.227
H(38)	-1.762	0.098	1.660
H(39)	-3.539	0.172	1.670
H(40)	-2.734	-1.390	1.511
O(41)	-2.838	1.262	-0.607
H(42)	-1.935	2.383	0.505
H(43)	-3.784	1.473	-0.577

Transition State D-TS6* (–814.874693 Hartree)

C(1)	-1.040	-0.125	-0.737
H(2)	-0.291	1.465	1.395
C(3)	-2.069	-1.081	-0.596
C(4)	0.272	-0.491	-1.334
H(5)	0.039	-0.427	-2.418
H(6)	0.459	-1.559	-1.183
C(7)	-3.387	-0.347	-0.325
H(8)	-3.898	-0.177	-1.285
H(9)	-4.069	-0.925	0.310
C(10)	-2.915	0.964	0.314
H(11)	-3.611	1.796	0.176
H(12)	-2.778	0.816	1.395
C(13)	-2.083	-2.437	-1.284
H(14)	-1.146	-2.989	-1.155
H(15)	-2.896	-3.049	-0.877
H(16)	-2.268	-2.309	-2.359
C(17)	-1.524	1.247	-0.323
H(18)	-1.680	1.769	-1.290
C(19)	-0.590	2.112	0.561
C(20)	-1.353	3.309	1.163
H(21)	-1.790	3.933	0.372
H(22)	-2.156	3.001	1.842
H(23)	-0.660	3.937	1.737
C(24)	0.676	2.652	-0.148
H(25)	1.424	2.862	0.630
H(26)	0.430	3.634	-0.578
C(27)	1.319	1.842	-1.286
H(28)	0.700	1.951	-2.191
H(29)	2.270	2.329	-1.539
C(30)	1.566	0.328	-1.123
H(31)	2.184	0.042	-1.986
C(32)	2.452	-0.131	0.075
C(33)	3.812	0.576	0.018
H(34)	3.705	1.648	0.217
H(35)	4.486	0.172	0.788
H(36)	4.283	0.449	-0.964
C(37)	1.848	-0.014	1.478
H(38)	0.852	-0.461	1.544
H(39)	2.496	-0.538	2.194
H(40)	1.786	1.029	1.801
O(41)	2.673	-1.570	-0.191
H(42)	1.502	-2.668	0.443
H(43)	3.545	-1.793	0.172

References

[1] H. Tesso, W. A. König, K.-H. Kubeczka, M. Bartnik, K. Glowniak, *Phytochemistry* **2005**, *66*, 707-713.

[2] E. Piers, K. F. Cheng, *Can. J. Chem.* **1970**, *48*, 2234-2245.

[3] L. Tissandie, S. Viciana, H. Brevard, U. J. Meierhenrich, J.-J. Filippi, *Phytochemistry* **2018**, *149*, 64-81.

[4] A. F. Thomas, M. Ozainne, *Helv. Chim. Acta* **1978**, *61*, 2874-2880.

[5] F. Sorm, L. Dolejs, O. Knessl, J. Pliva, *Coll. Czech. Chem. Commun.* **1950**, *15*, 82-95.

[6] L. Dolejs, A. Mironov, F. Sorm, *Tetrahedron Lett.* **1960**, *1*, 18-21.

[7] N. D. Lackus, J. Morawetz, H. Xu, J. Gershenzon, J. S. Dickschat, T. G. Köllner, *Molecules* **2021**, *26*, 555.

[8] G. Blay, B. Garcia, E. Molina, J. R. Pedro, *Tetrahedron* **2007**, *63*, 9621-9626.

[9] H. Nii, K. Furukawa, M. Iwakiri, T. Kubota, *Nippon Nogeik. Kaishi* **1983**, *57*, 733-741.

[10] A. C. Huang, C. J. Sumby, E. R. T. Tieking, D. K. Taylor, *J. Nat. Prod.* **2014**, *77*, 2522-2536.

[11] S. A. C. Mendes, T. A. Mansoor, A. Rodrigues, J. Bruges Armas, M.-J. U. Ferreira, *Phytochemistry* **2013**, *95*, 308-314.

[12] G. Singh, R. K. Upadhyay, *Fitoterapia* **1994**, *65*, 186-187.

[13] A. C. Huang, M. A. Sefton, C. J. Sumby, E. R. T. Tieking, D. K. Taylor, *J. Nat. Prod.* **2015**, *78*, 131-145.

[14] N. Tsubaki, K. Nishimura, Y. Hirose, *Bull. Chem. Soc. Jpn.* **1967**, *40*, 597-600.

[15] A. Gandurin, *Chem. Ber.* **1909**, *41*, 4359-4363.

[16] M. Ayubova, Z. Osman Guelleh, M. Osman Guelleh, H. Brevard, M. Baldovini, *Phytochemistry* **2019**, *164*, 78-85.

[17] C. Ehret, G. Ourisson, *Tetrahedron* **1969**, *25*, 1785-1799.

[18] G. M. König, A. D. Wright, *J. Org. Chem.* **1997**, *62*, 3837-3840.

[19] G. Rücker, F. W. Hefendehl, *Phytochemistry* **1978**, *17*, 809-810.

[20] S. Grimme, S. Ehrlich, L. Goerigk, *J. Comp. Chem.* **2011**, *32*, 1456-1465.

[21] M. J. Frisch et al., Gaussian 16, Revision B.01, Gaussian Inc., Wallingford CT, **2016**.

[22] Y. J. Hong, D. J. Tantillo, *J. Org. Chem.* **2018**, *83*, 3780-3793.

[23] C. Adamo, V. Barone, *J. Chem. Phys.* **1998**, *108*, 664-675.

[24] S. P. T. Matsuda, W. K. Wilson, Q. Xiong, *Org. Biomol. Chem.* **2006**, *4*, 530-543.

[25] L. Lauterbach, B. Goldfuss, J. S. Dickschat, *Angew. Chem. Int. Ed.* **2020**, *59*, 11943-11947; *Angew. Chem.* **2020**, *132*, 12041-12045.

[26] S. Grimme, *Chem. Eur. J.* **2012**, *18*, 9955-9964.

[27] G. Luchini, J. V. Alegre-Requena, Y. Guan, I. Funes-Ardoiz, R. S. Paton, GoodVibes v3.0.1, **2019**.

[28] P. Rabe, L. Barra, J. Rinkel, R. Riclea, C. A. Citron, T. A. Klapschinski, A. Janusko, J. S. Dickschat, *Angew. Chem.* **2015**, *127*, 13649-13653; *Angew. Chem. Int. Ed*. **2015**, *54*, 13448-13451.

[29] J. Rinkel, P. Rabe, X. Chen, T. G. Köllner, F. Chen, J. S. Dickschat, *Chem. Eur. J.* **2017**, *23*, 10501-10505.

[30] P. Rabe, J. Rinkel, E. Dolja, T. Schmitz, B. Nubbemeyer, T. H. Luu, J. S. Dickschat, *Angew. Chem.* **2017**, *129*, 2820-2823; *Angew. Chem. Int. Ed*. **2017**, *56*, 2776-2779.

[31] J. Rinkel, J. S. Dickschat, *Org. Lett.* **2019**, *21*, 2426-2429.

[32] P. Rabe, J. Rinkel, B. Nubbemeyer, T. G. Köllner, F. Chen, J. S. Dickschat, *Angew. Chem.* **2016**, *128*, 15646-15649; *Angew. Chem. Int. Ed*. **2016**, *55*, 15420-15423.
